# An umbrella review of candidate predictors of response, remission, recovery, and relapse across mental disorders

**DOI:** 10.1038/s41380-023-02298-3

**Published:** 2023-11-13

**Authors:** Marco Solmi, Samuele Cortese, Giovanni Vita, Michele De Prisco, Joaquim Radua, Elena Dragioti, Ole Köhler-Forsberg, Nanna M. Madsen, Christopher Rohde, Luis Eudave, Claudia Aymerich, Borja Pedruzo, Victoria Rodriguez, Stella Rosson, Michel Sabé, Mikkel Hojlund, Ana Catalan, Beatrice de Luca, Michele Fornaro, Giovanni Ostuzzi, Corrado Barbui, Gonzalo Salazar-de-Pablo, Paolo Fusar-Poli, Christoph U. Correll

**Affiliations:** 1 Department of Child and Adolescent Psychiatry, Charité Universitätsmedizin Berlin, Berlin, Germany; 2 Department of Psychiatry, University of Ottawa, Ottawa, ON Canada; 3 On Track: The Champlain First Episode Psychosis Program, Department of Mental Health, The Ottawa Hospital, Ottawa, ON Canada; 4 Ottawa Hospital Research Institute (OHRI) Clinical Epidemiology Program University of Ottawa, Ottawa, ON Canada; 5 School of Epidemiology and Public Health, Faculty of Medicine, University of Ottawa, Ottawa, ON Canada; 6 Centre for Innovation in Mental Health, School of Psychology, Faculty of Environmental and Life Sciences, University of Southampton, Southampton, UK; 7 Clinical and Experimental Sciences (CNS and Psychiatry), Faculty of Medicine, University of Southampton, Southampton, UK; 8 Solent NHS Trust, Southampton, UK; 9 Hassenfeld Children’s Hospital at NYU Langone, New York University Child Study Center, New York, NY USA; 10 Division of Psychiatry and Applied Psychology, School of Medicine, University of Nottingham, Nottingham, UK; 11 DiMePRe-J-Department of Precision and Regenerative Medicine-Jonic Area, University of Bari “Aldo Moro”, Bari, Italy; 12 WHO Collaborating Centre for Research and Training in Mental Health and Service Evaluation, Department of Neuroscience, Biomedicine, and Movement Sciences, Section of Psychiatry, University of Verona, Verona, Italy; 13 Bipolar and Depressive Disorders Unit, Hospìtal Clinic de Barcelona, c. Villarroel, 170, 08036 Barcelona, Spain; 14 Institut d’Investigacions Biomèdiques August Pi i Sunyer (IDIBAPS), c. Villarroel, 170, 08036 Barcelona, Spain; 15 Centro de Investigación Biomédica en Red de Salud Mental (CIBERSAM), Instituto de Salud Carlos III, Madrid, Spain; 16 Institut d’Investigacions Biomèdiques August Pi i Sunyer (IDIBAPS), Imaging of Mood- and Anxiety-Related Disorders (IMARD), CIBERSAM, University of Barcelona, Barcelona, Spain; 17 University of Ioannina, Research Laboratory Psychology of Patients, Families & Health Professionals, Department of Nursing, School of Health Sciences, Ioannina, Greece; 18 Linköping University, Pain and Rehabilitation Centre and Department of Health, Medicine and Caring Sciences, Linköping, Sweden; 19 Psychosis Research Unit, Aarhus University Hospital Psychiatry, Aarhus, Denmark; 20 Department of Clinical Medicine, Aarhus University, Aarhus, Denmark; 21 Department of Affective Disorders, Aarhus University Hospital - Psychiatry, Aarhus, Denmark; 22 Faculty of Education and Psychology, University of Navarra, Pamplona, Spain; 23 Biobizkaia Health Research Institute, Basurto University Hospital, OSI Bilbao-Basurto. University of the Basque Country UPV/EHU. Centro de Investigación en Red de Salud Mental. (CIBERSAM), Instituto de Salud Carlos III. Plaza de Cruces 12, 48903 Barakaldo, Bizkaia Spain; 24 Psychiatry Department, Basurto University Hospital, Bilbao, Spain; 25 Department of Psychosis Studies, King’s College London, London, UK; 26 Mental Health Department, Local Health Unit ULSS3 Serenissima, Venice, Italy; 27 Division of Adult Psychiatry, Department of Psychiatry, University Hospitals of Geneva, 2, Chemin du Petit-Bel-Air, CH-1226 Thonex, Switzerland; 28 Department of Psychiatry Aabenraa, Mental Health Services Region of Southern Denmark, Aabenraa, Denmark; 29 Clinical Pharmacology, Pharmacy, and Environmental Medicine, Department of Public Health, University of Southern Denmark, Odense, Denmark; 30 Child and Adolescent Mental Health Centre, Mental Health Services Capital Region of Denmark, Copenhagen, Denmark; 31 Department of Psychiatry, Federico II of Naples, Naples, Italy; 32 Department of Child and Adolescent Psychiatry, Institute of Psychiatry, Psychology & Neuroscience, King’s College London, London, UK; 33 Child and Adolescent Mental Health Services, South London and Maudsley NHS Foundation Trust, London, UK; 34 Institute of Psychiatry and Mental Health. Department of Child and Adolescent Psychiatry, Hospital General Universitario Gregorio Marañón School of Medicine, Universidad Complutense, Instituto de Investigación Sanitaria Gregorio Marañón (IiSGM), CIBERSAM, Madrid, Spain; 35 Department of Brain and Behavioral Sciences, University of Pavia, Pavia, Italy; 36 Outreach and Support in South London (OASIS) service, NHS South London and Maudsley Foundation Trust, London, UK; 37 Department of Psychiatry and Psychotherapy, Ludwig-Maximilian-University Munich, Munich, Germany; 38 The Zucker Hillside Hospital, Northwell Health, New York, NY USA; 39 Donald and Barbara Zucker School of Medicine at Hofstra/Northwell, New York, NY USA; 40 The Feinstein Institute for Medical Research, Center for Psychiatric Neuroscience, Manhasset, NY USA

**Keywords:** Psychiatric disorders, Depression, Schizophrenia, Addiction

## Abstract

We aimed to identify diagnosis-specific/transdiagnostic/transoutcome multivariable candidate predictors (MCPs) of key outcomes in mental disorders. We conducted an umbrella review (protocol link), searching MEDLINE/Embase (19/07/2022), including systematic reviews of studies reporting on MCPs of response, remission, recovery, or relapse, in DSM/ICD-defined mental disorders. From published predictors, we filtered MCPs, validating MCP criteria. AMSTAR2/PROBAST measured quality/risk of bias of systematic reviews/individual studies. We included 117 systematic reviews, 403 studies, 299,888 individuals with mental disorders, testing 796 prediction models. Only 4.3%/1.2% of the systematic reviews/individual studies were at low risk of bias. The most frequently targeted outcome was remission (36.9%), the least frequent was recovery (2.5%). Studies mainly focused on depressive (39.4%), substance-use (17.9%), and schizophrenia-spectrum (11.9%) disorders. We identified numerous MCPs within disorders for response, remission and relapse, but none for recovery. Transdiagnostic MCPs of remission included lower disease-specific symptoms (disorders = 5), female sex/higher education (disorders = 3), and quality of life/functioning (disorders = 2). Transdiagnostic MCPs of relapse included higher disease-specific symptoms (disorders = 5), higher depressive symptoms (disorders = 3), and younger age/higher anxiety symptoms/global illness severity/ number of previous episodes/negative life events (disorders = 2). Finally, positive trans-outcome MCPs for depression included less negative life events/depressive symptoms (response, remission, less relapse), female sex (response, remission) and better functioning (response, less relapse); for schizophrenia, less positive symptoms/higher depressive symptoms (remission, less relapse); for substance use disorder, marital status/higher education (remission, less relapse). Male sex, younger age, more clinical symptoms and comorbid mental/physical symptoms/disorders were poor prognostic factors, while positive factors included social contacts and employment, absent negative life events, higher education, early access/intervention, lower disease-specific and comorbid mental and physical symptoms/conditions, across mental disorders. Current data limitations include high risk of bias of studies and extraction of single predictors from multivariable models. Identified MCPs can inform future development, validation or refinement of prediction models of key outcomes in mental disorders.

## Introduction

Mental and substance use disorders have their mean onset in adolescence (14.5 years) [1], are among the 30 leading causes of disability globally and are associated with increased mortality [2].

Social and clinical outcomes of mental disorders are associated with non-modifiable (e.g., age, sex) and modifiable (e.g., disease symptoms, education, employment status, treatment type, and dose) patient-, illness-, or treatment-related factors, and can be targeted by pharmacological and non-pharmacological interventions [3]. However, rates of response, remission, and recovery, and relapses in mental disorders remain suboptimal. For instance, for patients with multi-episode schizophrenia, 51%, 23%, and 13.5% of subjects have a ≥ 20%, ≥50% reduction of total symptoms [4] or reach recovery [5]. In depressive disorders, treatment response ranges from 51 to 54%, remission is around 43% [6], and throughout 26 weeks, relapse occurs in about 33–50% [7] of cases. In anxiety disorders, response to pharmacotherapy ranges from 52 to 56% [8]. In substance use disorders, over a follow-up of 17 years, remission ranges from 35 to 54% [9].

One major of the many reasons behind suboptimal outcomes is the lack of personalized care [10]. Personalized approaches to predict outcomes are available in some areas of medicine, especially cancer [11], but remain aspirational in psychiatry. Personalized approaches are possible when validated and implemented clinical prediction models forecasting disease course (prognostic) or treatment response (predictive) are available [12]. Reasons behind lacking implementation of published predictors include risk of bias, lack of replication and limited generalizability. Indeed, two systematic reviews have recently applied stringent methodological criteria, showing that only a few multivariable prediction models are available in psychiatry that have been internally and externally validated [10, 13], and these do not focus on clinical outcomes but disease onset. To advance the field of stratified/precision psychiatry, multivariable candidate predictors (MCPs) supported by at least preliminary evidence need to be identified, enabling methodologically sound studies to incorporate them to refine promising prediction models and validate or refute their prognostic/predictive accuracy before translating them into clinical practice [12].

Given the high clinical relevance of predictors of response, remission, recovery, and relapse, and given the lack of a comprehensive umbrella review across mental disorders pooling data from different systematic reviews in this area, we aimed to identify multivariable candidate prognostic/predictive factors of key clinical real-world outcomes in psychiatry (response, remission, recovery, and relapse) across mental disorders and age groups. We also sought to dissect potentially disease-specific, trans-outcome, and transdiagnostic prognostic/predictive factors, providing leads to the field for further in-depth investigation.

## Methods

### Search strategy, inclusion, and exclusion criteria

A more comprehensive version of the methods is available in eMethods. We conducted an umbrella review of systematic reviews, including observational or interventional studies testing predictors of response, remission, recovery, and/or relapse in people with mental disorders. We followed an a-priori protocol (https://osf.io/gpysa/). We searched MEDLINE/Embase until 19/07/2022, and used the PRIOR checklist [14]. Protocol amendments, PRIOR checklist, and search key are available in the supplementary material.

Inclusion criteria were: i) systematic reviews, including ii) longitudinal (randomized) controlled trials (RCTs), open-label studies, or cohort studies; iii) including individuals with DSM/ICD-defined mental disorders, iv) reporting on multivariable models; and v) that tested prognostic or predictive factors; vi) of study-defined response, remission, recovery and/or relapse.

Exclusion criteria were: i) nonsystematic reviews, ii) individual studies not included in systematic reviews, iii) not using DSM/ICD criteria to define mental disorders; iv) testing univariable models only; v) focusing on cross-sectional markers instead of prognostic/predictive factors; or vi) not reporting on the outcomes of interest.

We identified eligible systematic reviews. Then, we included eligible individual studies among those included in the systematic reviews (two independent authors, CA, AC, BDL, MDP, LE, OKF, MF, CGR, NMM, MH, BP, VR, SR, GSDP, MS, GV). A third author resolved any conflict (CUC, MS).

#### Prognostic and predictive factors

Prognostic factors were evaluated in studies that used “treatment as usual”, or generically a “pharmacological treatment”, or “psychological treatment”, or in studies that reported on associations between a (set of) factor(s) within one treatment group only. Predictive factors were those investigated in studies that specifically measured outcomes of a specific intervention, typically in (R)CT, but also in some longitudinal studies, provided that a control group was present (cohort studies) and accounted for in the analyses.

#### Operationalization of predictors

We anticipated that eligible studies operationalized the same predictors in many different formats, but we could not know a-priori all possible (combinations of) definitions used in the literature. We organized the extracted predictors into three levels: I (micro-level), II (meso-level), and III (macro-level), with decreasing granularity. Level I (micro-level) was the individual study authors’ definition of the predictor (e.g., age as a continuous variable, age groups). Level II was a broader category into which we lumped different level I (micro-level) predictors (e.g., the meso-level level II category “age” consisted of a continuous variable, or of two or more age groups), thereby grouping different age operationalizations into the same predictor (level II, meso-level). Before pooling level I (micro-level) predictors into level II (meso-level) categories, we performed additional steps to ensure coherent reporting. Some studies reported on the same predictor using different reference groups. Other studies operationalized the predictor in opposite directions, i.e., longer or shorter duration of untreated illness as a predictor of response. For studies using different reference groups or the opposite direction of the predictor, we harmonized the direction of the effect size. Level II (meso-level) predictors were then pooled into broader level III (macro-level) categories according to the results found at level II.

Specific pharmacologic or non-pharmacologic treatments were not included as predictive factors, only different ways of dosing them (e.g., dose/frequency reduction, low dose/frequency, dose above the package insert dosing range), reflecting different global treatment strategies, instead [15–18].

#### Outcomes

We a-priori included studies focusing on response, remission, recovery, and relapse. We assumed that response was broadly defined as an improvement in symptoms or change compared to baseline [19, 20]; remission as reaching a minimum level of symptoms, including syndromal remission of symptoms not meeting criteria for a given disorder [21]; recovery as the presence of symptomatic remission plus a minimum level of functioning or adequate level of functioning alone, which by inference would represent a non-interfering level of symptoms [5]; and relapse as a worsening of symptoms with or without need for hospitalization. We originally planned to accept the authors’ definitions of response, remission, recovery, and relapse. However, as, when extracting data, some outcomes were mislabeled, we amended them.

#### Data extraction, quality, and risk of bias assessment

Data were extracted by two authors independently (MDP, MH, MS, GV). A third author resolved conflicts (CUC, MS). We extracted bibliographic identifiers and descriptive characteristics of systematic reviews and individual studies. For individual studies, we also extracted study design, diagnosis, diagnostic criteria, setting, age, follow-up duration, sample size, exposure/intervention and control, outcome definition, predictors in each multivariable model, frequency of the outcome, the statistical approach employed (e.g., regression analysis, machine learning), each predictors’ coefficient and *p* value, and performance metrics of the multivariable model.

Data to assess the quality of eligible systematic reviews (AMSTAR 2) [22] and risk of bias of individual studies (PROBAST) [23] were extracted.

#### Definition of predictors

From the overall set of “published” predictors, we extracted MCPs based on the following criteria: i) tested in ≥2 studies, ii) in specific spectra of mental disorders, iii) ≥2 significant findings with ≤20% of the significant associations going in the opposite/unknown direction, and iv) reporting model performance. Additional types of predictors satisfying criteria i) and ii) only, were labeled “broadly-defined” predictors. We conducted simulation analyses to test whether the criteria defining MCPs were sensitive and specific. Predictors were also classified into modifiable or non-modifiable, and patient-, ilnness-, or treatment-related factors.

## Results

A more detailed results section is available in eResults.

### Search results and characteristics of systematic reviews and individual studies of published predictors

**Fig. 1:**
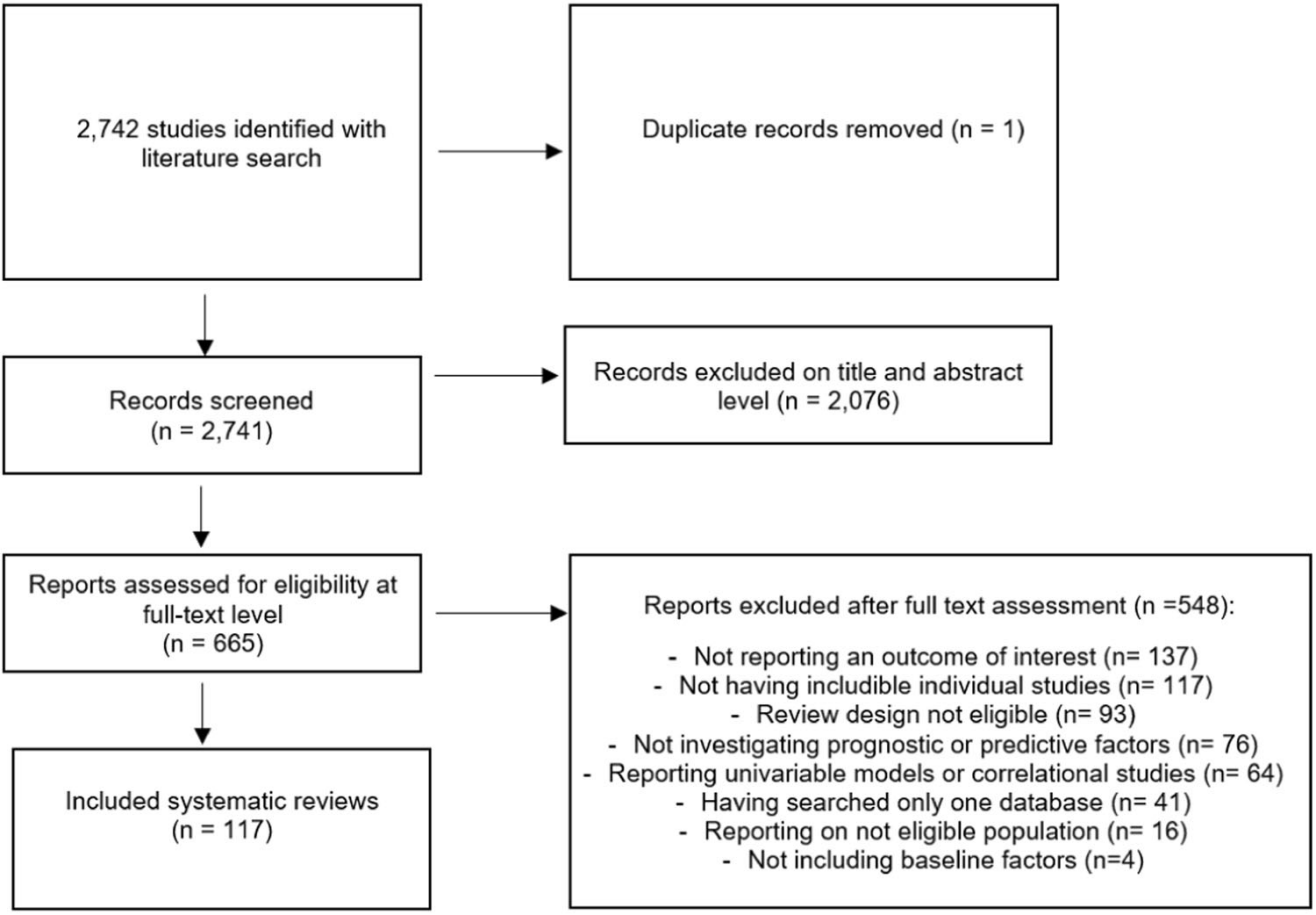
Study selection flow-chart. Screening procedure from title/abstract screening to final inclusion of eligible reviews.

From 2742 initial hits, we included 117 systematic reviews of 403 individual studies and 299,888 persons with mental disorders (Fig. 1, eTables 1–3, eFig. 1).

Across systematic reviews, the median number of included studies was 4.4 (range = 1–62), the median sample size was 316 (range=23-159,299) individuals, and predictors were reported across a median of 3 (range=1-9) level III (macro-level) domains. Most studies included adults (*N* = 311, 77.2%), with some focusing on elderlies (*N* = 40, 9.9%), children or adolescents (*N* = 36, 8.9%), or mixed age groups (*N* = 16, 3.9%).

At the basic published predictor level, most individual studies focused on depressive disorders (*N* = 159), followed by substance-use disorders (*N* = 72), schizophrenia-spectrum and other psychotic disorders (*N* = 48), and other disorders. Among eligible individual studies, 285 (70.8%) were cohort studies, 85 (21.1%) RCTs, 24 (5.9%) open-label studies, and 9 (2.2%) were non-randomized controlled trials. The vast majority of studies (372, 92.3%) tested prognostic factors, 19 (4.7%) predictive factors, and 12 (3%) tested both. Overall, 149 (36.9%) studies considered remission as an outcome, 142 (35.2%) relapse, 68 (16.8%) response, 10 (2.5%) recovery, and 34 (8.4%) of the studies considered multiple outcomes.

### Quality of included systematic reviews and individual studies

**Fig. 2:**
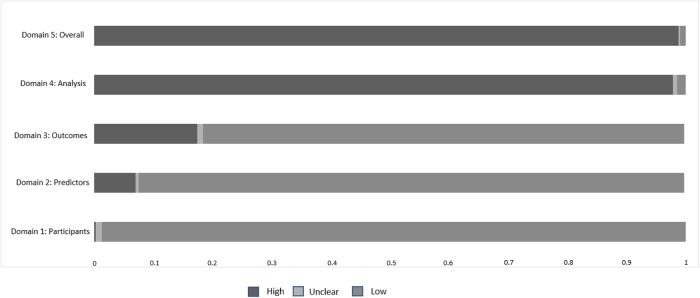
Risk of bias of published predictors models of response, remission, recovery, and relapse across mental disorders, according to PROBAST. The risk of bias of models included in this review is described across the four PROBAST domains. PROBAST Prediction model Risk Of Bias Assessment Tool.

Quality of the systematic reviews was high in 4.3%, moderate in 8.5%, low in 19.7%, and critically low in 67.5% (AMSTAR2). The risk of bias in individual studies was high in 98.8%. The “analysis” domain had the highest risk of bias, with 97.7% of studies being at high risk of bias (Fig. 2).

### Predictors of response, remission, recovery, and relapse across mental disorders, their model development or validation testing stage, and outcome operationalization

Overall, 2622 level I (micro-level) predictors were operationalized in 229 level II (meso-level, published predictors) categories across nine level III (macro-level) groups of predictors (i.e., biochemical, clinical, genetic, neuroimaging, neurophysiological, neuropsychological, pharmacological/treatment, psychological, and sociodemographic and environmental). Each predictor, at any level, could be used across multiple diagnostic groups, age groups, and outcomes, resulting in a higher number of predictors by diagnostic and age group, and outcome. The complete list of level III (macro-level) and level II (meso-level) published predictors, together with the level I predictors by the outcome, is available in eTable 4. The nine-level III (macro-level) predictor categories consisted of the following level I (micro**-**level) predictors: clinical: 50.6%, sociodemographic and environmental: 23.7%, genetic: 11.3%, pharmacological/treatment: 4.1%, psychological: 2.7%, neuropsychological: 2.6%, neuroimaging: 2.5%, biochemical: 1.7%, and neurophysiological: 0.8%.

Among the 229 level II (meso-level) published predictors, 96 level II (meso-level) predictors met criteria of broadly defined, and 36 for MCPs, which were used 1024, 339, and 72 times, respectively, across different diagnostic groups, age groups, and outcomes. The level II (meso-level) predictors by diagnostic group and outcomes are available in eFig. 2 and eTable 5.

For level II (meso-level) MCPs of response, remission and relapse, fulfilling the stringent criteria, the sample size, number of studies, number of models, the median and range of level II (meso-level) MCPs in each model, the median and range of the effect size of predictors significantly associated with outcomes, the validation testing stage (development, internal validation testing, external validation testing), and performance measures of models including each MCP, are reported in Tables 1 and 2. No MCPs of recovery emerged. Broadyl-defined predictors are reported in eTables 6–9, that do not meet the proposed “credibility” criteria for MCPs.

**Table 1:** Multivariable candidate predictors of response and remission across mental disorders.

Predictor^a^	*N*	Studies/models	Median/range of predictors in the models	Significant^b^ predictor coefficient median/range	Model D/I/E	Model accuracy measures median/range^c^
**Response**						
Non-modifiable Multivariable Candidate Predictors						
*Depressive Disorders (Major Depressive Disorder), adults*						
*Patient factors*						
Sex (females)	3328	13/16	8/2–15	OR: 1.53 (1); Beta: 5.28/5.12–5.44 (2)	16/0/0	Accuracy: 74.8% (1); *R*^2^: 64%/13–65% (3)
5-HT transporter polymorphisms (yes)	625	4/5	5/4–22	OR: 6.46/2.88–20.11 (8)	5/0/0	Accuracy: 74% (1); *R*^2^: 13% (1)
Modifiable Multivariable Candidate Predictors						
*Depressive Disorders (Major Depressive Disorder), adults*						
*Patient factors*						
Marital status (married)	2099	5/5	7/4–15	OR: 1.7/1.41–2.89 (3)	5/0/0	Accuracy: 70.9%/67–74.8% (2)
Negative life events (no)	495	3/5	7/5–13	OR: 2.56 (1); Beta: 6.88/6.06-6.92 (4)	5/0/0	*R*^2^: 64%/23–65% (3)
*Illness factors*						
Depressive symptomatology (lower)	2639	13/16	7/4–15	OR: 1.17/0.90–3.57 (5)	16/0/0	Accuracy: 74.8% (1); *R*^2^: 65%/64–65% (2)
Functioning (higher)	2013	3/3	10/4–13	OR: 1.9/1.86–2.3 (3); Wald: 4.902 (1)	3/0/0	*R*^2^: 64% (1)
*Treatment factors*						
Early treatment response (yes)	245	3/4	5/3-9	OR: 7.76/6.22–9.3 (2); Beta: 1.32 (1)	2/0/2	AUC: 91%/62–92% (4); *R*^2^: 74/74–74% (2)
*Depressive Disorders (Major Depressive Disorder, Minor Depression), older adults*						
*Illness factors*						
Depressive symptomatology (lower)	883	3/12	7/4–15	OR: 3.84 (1); Beta: 0.04 (1)	12/0/0	C-statistic: 0.75 (1)
**Remission**						
Non-modifiable Multivariable Candidate Predictors						
*Depressive Disorders (Major Depressive Disorder, Persistent Depressive Disorder), adults*						
*Patient Factors*						
Sex (females)	2810	9/17	5/4–48	OR: 1.42/1.21–2.17 (5)	14/3/0	Accuracy: 72.2%/65.5–85% (2); NPV: 81%/77–87% (7); PPV: 50%/43–78% (7); Sensitivity: 69%/64–75% (7);Specificity: 70%/62–93% (7)
*Depressive Disorders (Major Depressive Disorder, Minor Depression, Persistent Depressive Disorder), older adults*						
*Patient Factors*						
Sex (males)	2234	12/26	5/2–16	OR: 1.49 (1); HR: 1.71/1.58–1.85 (2)	23/1/2	Accuracy: 70%/67–89.47% (3); AUC: 71.5%/70–74% (4); Sensitivity: 64%/60–88.89% (5); Specificity: 72%/67–90% (5)
*Schizophrenia-Spectrum and Other Psychotic Disorders (Schizophrenia, Schizoaffective Disorder, Schizophrenia-Spectrum Disorders, FEP), adults*						
*Patient Factors*						
Sex (females)	2640	7/10	18/8–26	OR: 1.29/1.28–1.31 (3); HR: 2.56 (1)	9/1/0	NPV: 88%/85–92% (2); PPV: 90%/89–92% (2)
*Substance-Related and Addictive Disorders (Alcohol, Cannabis, Cocaine, Opioid, Tobacco, Substance Use Disorder), adults*						
*Patient Factors*						
Sex (females)	17,912	7/14	16/9–31	OR: 2/2–2 (2); HR: 1.38/1.23–1.56 (4)	14/0/0	Accuracy: 73%/31–77% (6)
Modifiable Multivariable Candidate Predictors						
*Anxiety Disorders (Generalized Anxiety Disorder, Panic Disorder, Separation Anxiety Disorder, Social Anxiety Disorder, Specific Phobia), children/adolescents*						
*Illness Factors*						
Anxious symptomatology (lower)	1062	5/6	10/8–23	OR: 1.05/1.05–1.07 (3)	5/1/0	AUC: 66% (1); *R*^2^: 24.8%/20.9–28.7% (2)
*Depressive Disorders (Major Depressive Disorder), children/adolescents*						
*Illness Factors*						
Depressive symptomatology (lower)	1059	5/5	8/5–15	OR: 1.07/1.06–1.08 (2); HR: 1.09 (1); RR: 2.63 (1)	5/0/0	AUC: 66% (1)
*Depressive Disorders (Major Depressive Disorder, Persistent Depressive Disorder), adults*						
*Patient Factors*						
Negative life events (no)	2960	6/25	3/3–25	OR: 2.32/2.22–2.85 (3)	25/0/0	AUC: 80% (1); *R*^2^: 28% (1); Sensitivity: 77% (1); Specificity: 66% (1)
General physical health (better)	1807	5/9	6/5–48	OR: 1.2/1.14–3.7 (7)	9/0/0	Accuracy: 85% (1); NPV: 87% (1); PPV: 78% (1); Sensitivity: 64% (1); Specificity: 93% (1)
Education (higher)	5146	4/6	22/5–48	OR: 1.30/1.03–1.47 (5)	4/1/1	Accuracy: 64.6%/59.6–85% (3); AUC: 68%/66–70% (2); NPV: 65.3%/65–87% (3); PPV: 65%/56–78% (4); Sensitivity: 62.8%/49.4–64% (3); Specificity: 70.8%/66.2–93% (3)
Emotional regulation/coping strategies (higher)	1026	4/9	5/2–5	OR: 2.21/2.13–2.3 (2)	6/3/0	NPV: 80.5%/77–88% (6); PPV: 49%/43–64% (6); *R*^2^: 23% (1); Sensitivity: 70%/67–75% (6); Specificity: 68.5%/62–73% (6)
Employment status (employed)	3841	4/6	15/3–48	OR: 1.28/1.03–1.75 (5)	4/1/1	Accuracy: 64.6%/59.6–85% (3); AUC: 71%/70–73% (2); NPV: 65.3%/65–87% (3); PPV: 64%/56–78% (3); Sensitivity: 62.8%/49.4–64% (3); Specificity: 70.8%/66.2–93% (3)
Quality of life (higher)	1003	3/4	6/2–7	OR: 1.07/1.04–2.5 (3)	3/0/0	*R*^2^: 48% (1)
*Illness Factors*						
Anxiety comorbidity (no)	3693	4/5	5/4–48	OR: 3.44/2.27–7.14 (2)	5/0/0	Accuracy: 85% (1); NPV: 87% (1); PPV: 78% (1); *R*^2^: 6.6%/6.3–48% (3); Sensitivity: 64% (1); Specificity: 93% (1)
OCD comorbidity (no)	1270	2/6	6/6–48	OR: 1.42/1.40–1.42 (3)	6/0/0	Accuracy: 85% (1); NPV: 87% (1); PPV: 78% (1); Sensitivity: 64% (1); Specificity: 93% (1)
PTSD comorbidity (no)	1270	2/5	6/6–48	OR: 1.47/1.42–1.58 (4)	5/0/0	Accuracy: 85% (1); NPV: 87% (1); PPV: 78% (1); Sensitivity: 64% (1); Specificity: 93% (1)
Personality disorder comorbidity (no)	429	3/3	4/2–5	OR: 6.25 (1)	3/0/0	Accuracy: 80% (1)
Global severity symptomatology (lower)	1337	3/6	6/3–48	OR: 1.12/1.08–1.13 (4)	6/0/0	Accuracy: 85% (1); NPV: 87% (1); PPV: 78% (1); Sensitivity: 64% (1); Specificity: 93% (1)
Episode/hospitalization duration (lower)	3053	7/7	5/4-19	OR: 1.69/1.04–4.34 (2)	7/0/0	Accuracy: 80% (1); AUC: 66% (1); PPV: 67% (1)
Depressive symptomatology (lower)	9774	22/28	8/3-125	OR: 1.17/1.04–2.7 (7); RR: 1.14 (1)	19/6/3	Accuracy: 68.75%/59.6–85% (6); AUC: 73.5%/66–83% (10); NPV: 65.3%/65–87% (3); PPV: 65.5%/56–78% (4); *R*^2^: 30%/14.9–48% (9); Sensitivity: 68%/49.4–78% (8); Specificity: 74%/65–93% (8)
*Depressive Disorders (Major Depressive Disorder, Minor Depression, Persistent Depressive Disorder), older adults*						
*Patient Factors*						
Social contacts (higher)	1416	6/8	7/2–13	OR: 1.76/1.19-4 (4); Wald: 4.47 (1)	7/0/1	AUC: 75%/70–77% (3)
General physical health (better)	1989	8/15	5/4–15	OR: 1.40/1.14-1.81 (2); HR: 1.44 (1)	14/0/1	Accuracy: 70.8%/70–71.6% (2); AUC: 72%/70–74% (2); Sensitivity: 66%/60–72% (2); Specificity: 70.5%/67-74% (2)
*Illness Factors*						
Anxiety comorbidity (no)	1539	4/5	5/4–7	OR: 1.58 (1)	4/0/1	Accuracy: 71.6% (1); AUC: 72.5%/70–75% (2)
Functioning (higher)	905	4/4	7/4–13	OR: 1.03/1.03-1.04 (2); HR: 1.69 (1)	4/0/0	AUC: 77% (1)
Episode/hospitalization duration (lower)	483	3/6	5/4–15	OR: 1.85 (1)	4/0/2	Accuracy: 68.5%/67–70% (2); AUC: 71.5%/70–74% (4); Sensitivity: 62.5%/60–72% (4); Specificity: 71.5%/67–74% (4)
*Feeding and Eating Disorders (Anorexia Nervosa, Bulimia Nervosa, Binge-eating Disorder, Eating Disorders not specified), adults*						
*Illness Factors*						
Weight (higher)	1016	9/12	5/2–14	OR: 1.72/1.06–2.18 (6); HR: 1.16 (1)	12/0/0	Accuracy: 78.2% (1); *R*^2^: 38.5%/33–44% (2)
Eating disorder symptomatology (lower)	1231	7/8	5/3–14	OR: 1.07/0.94–1.08 (6)	8/0/0	*R*^2^: 40% (1);
Duration of illness (lower)	561	4/5	6/4–10	OR: 2.56/1.28–2.77 (3)	5/0/0	*R*^2^: 45% (1)
*Schizophrenia-Spectrum and Other Psychotic Disorders (Schizophrenia, Schizoaffective Disorder, Schizophrenia-Spectrum Disorders, FEP), adults*						
*Patient Factors*						
Quality of life (higher)	891	2/2	5/5–5	OR: 1.03 (1)	2/0/0	AUC: 86.1 (1); PPV: 86% (1); *R*^2^: 63%/54–72% (2); Sensitivity: 89.9% (1); Specificity: 81.2% (1)
Education (higher)	2529	6/11	18/8–30	OR: 1.62/1.49–1.74 (2)	7/1/3	Accuracy: 64.75%/62.5-67 (2); AUC: 63%/62–73% (5); NPV: 75.9%/60.6–86% (4); PPV: 78.4%/64.5–92% (4); Sensitivity: 59.5%/58.4–60.6 % (2); Specificity: 69.8%/64.5–75.1% (2)
*Illness Factors*						
Duration of untreated illness (lower)	3784	10/15	13/3–30	OR: 1.20/1.01–2.32 (6)	12/1/2	Accuracy: 67% (1); AUC: 68%/62–70.3% (3); NPV: 85%/66.7–86% (3); PPV: 89%/67.9–92% (3); Sensitivity: 58.4% (1); Specificity: 75.1% (1)
Negative psychotic symptomatology (lower)	3208	9/13	18/9-30	OR: 1.07/1.03–1.29 (6)	9/1/3	Accuracy: 64.7%/62.5–67% (2); AUC: 65.5%/62–86.1% (6); NPV: 75.8%/60.6–86% (4); PPV: 86%/64.5–92% (5); *R*^2^: 63%/54–72% (2); Sensitivity: 60.6%/58.4–89.9% (3); Specificity: 75.1%/64.5–81.2% (3)
Positive psychotic symptomatology (lower)	3208	9/15	18/5–26	OR: 1.21/1.12–1.56 (3)	11/1/3	Accuracy: 64.7%/62.5–67% (2); AUC: 65.5%/62–86.1% (6); NPV: 75.8%/60.6–86% (4); PPV: 86%/64.5–92% (5); *R*^2^: 63%/54–72% (2); Sensitivity: 60.6%/58.4–89.9% (3); Specificity: 75.1%/64.5–81.2% (3)
Depressive symptomatology (higher)	166	3/5	18/13–26	HR: 1.18 (1)	3/1/1	Accuracy: 62.5% (1); AUC: 63%/63–63% (2); NPV: 85%/60.6–86% (3); PPV: 89%/64.5–92% (3); Sensitivity: 60.6% (1); Specificity: 64.5% (1)
Functioning (higher)	2765	4/7	18/9–30	OR: 4.35/1.02-7.69 (2)	5/0/2	Accuracy: 67% (1); AUC: 68%/62–70.3% (3); NPV: 66.7% (1); PPV: 67.9% (1); Sensitivity: 58.4% (1); Specificity: 75.1% (1)
*Substance-Related and Addictive Disorders (Alcohol, Cannabis, Cocaine, Opioid, Tobacco, Substance Use Disorder), adults*						
*Patient Factors*						
Marital status (married)	16,247	6/12	31/6–31	OR: 2.86/2.27–3.45 (2); HR: 1.29 (1)	12/0/0	Accuracy: 73%/31–77% (6)
Education (higher)	11,404	5/10	14/9–31	OR: 2.28/1.5–3.03 (2); HR: 1.1 (1)	10/0/0	Accuracy: 73%/31–77% (6)
*Illness Factors*						
SUD lifetime comorbidity, other (no)	5320	5/7	11/6–16	OR: 4/3.12–5.55 (3); HR: 2.56/1.56–9.09 (4)	7/0/0	Accuracy: 64% (1)
Depressive comorbidity (yes)	1403	4/4	6/5–9	OR: 2.15/1.82–2.49 (2)	4/0/0	Accuracy: 77.2% (1); *R*^2^: 6.4% (1)

*AUC* area under the curve, *D* development sample, *E* external validation sample, *HR* hazard ratio, *I* internal validation sample, *k* number of studies, *N* number of subjects, *NA* not available, *NPV* negative predictive value, *OR* odds ratio, *PPV* positive predictive value, *RR* relative risk, *SUD* substance use disorder.

^a^In brackets value of predictor associated with remission.

^b^Coefficient of predictors significantly associated with outcome in multivariable models.

^c^Studies might have reported more than one accuracy measure, so the number of studies can exceed *k.*

**Table 2:** Multivariable candidate predictors of relapse/hospitalization across mental disorders.

Predictor^a^	*N*	Studies/models	Median/range of predictors in the models	Significant^b^ predictor coefficient median/range	Model D/I/E	Model accuracy measures median/range^c^
Non-modifiable Multivariable Candidate Predictors						
*Bipolar and Related Disorders (Bipolar Disorder), adults*						
*Patient Factors*						
Sex (males)	8320	6/7	8/2––14	HR: 2.12/1.19–2.42 (3)	7/0/0	Accuracy: 98% (1); AUC: 88% (1); NPV: 98.2% (1); PPV: 66.7% (1); Specificity: 99.7% (1)
*Depressive Disorders (Major Depressive Disorder, Persistent Depressive Disorder, Depressive Disorder not specified), adults*						
*Patient Factors*						
Psychiatric family history (yes)	5325	6/7	15/6–81	OR: 1.87 (1); HR: 2.12 (1)	6/0/1	AUC: 74.6%/61–79% (3)
Age (younger)	8138	16/18	11/5–31	HR: 1.04 (1)	17/0/1	AUC: 73%/71–75% (2); *R*^2^: 52% (1)
*Substance-Related and Addictive Disorders (Alcohol, Cannabis, Cocaine, Opioid, Tobacco, Substance Use Disorder), adults*						
*Patient Factors*						
Age (younger)	15,933	20/22	8/2–23	OR: 1.20/1.14–1.25 (2); HR: 1.01 (1); Beta: 0.102 (1)	22/0/0	*R*^2^: 37.6%/33.4–55% (3)
*Illness Factors*						
Age at onset (younger)	5273	7/7	7/3–28	OR: 3.22 (1); HR: 1.02 (1)	7/0/0	*R*^2^: 13.3% (1)
Gray matter volume (lower)	340	4/5	5/3–6	HR: 1.79/1.78–1.81 (2); Beta: 0.64/36.13–0.40 (5)	5/0/0	Accuracy: 94% (1)
Modifiable Multivariable Candidate Predictors						
*Anxiety Disorders (Generalized Anxiety Disorder, Panic Disorder, Social Anxiety Disorder), adults*						
*Patient Factors*						
Negative life events (yes)	1589	3/4	11/11–11	RR: 3.2 (1); Beta: 0.55 (1)	3/0/1	AUC: 79% (1)
*Illness Factors*						
Anxious symptomatology (higher)	1584	3/5	11/11–11	OR: 1.07 (1); Beta: 0.49/0.44–0.54 (2)	4/0/1	AUC: 79% (1)
*Bipolar and Related Disorders (Bipolar Disorder), adults*						
*Patient Factors*						
Social contacts (lower)	2068	3/5	12/3–12	OR: 1.38/1.08–16.66 (3)	5/0/0	*R*^2^: 11%
*Illness Factors*						
Depressive symptomatology (higher)	1415	3/5	4/2–11	HR: 1.12/1.05–1.2 (4)	4/1/0	AUC: 82% (1); Sensitivity: 74% (1); Specificity: 78% (1)
*Depressive Disorders (Major Depressive Disorder, Persistent Depressive Disorder, Depressive Disorder not specified), adults*						
*Patient Factors*						
Negative life events (yes)	7618	11/14	12/2–81	OR: 3.77/1.58–8.95 (6); HR: 1.89/1.01–2.89 (4)	12/0/2	AUC: 74.6%/61–79% (5); *R*^2^: 52% (1)
*Illness Factors*						
Functioning (lower)	449	3/3	3/2–18	OR: 1.72/1.35–2.38 (2)	3/0/0	*R*^2^: 52% (1)
Depressive symptomatology (higher)	6567	20/23	14/2–70	OR: 1.81 (1); HR: 1.06/1.04–1.91 (3) R: 0.17 (1)	20/1/2	AUC: 72.8%/61–79% (6); NPV: 63%/57–81.57% (3); PPV: 59%/41.64–72% (3); Sensitivity: 34%/16–52% (2); Specificity: 82%/69–95% (2)
Number of episodes (higher)	5161	14/17	9/2–81	HR: 1.68/1.59–2.48 (3); R: 0.14 (1)	14/1/2	AUC: 72.8%/61–79% (6); NPV: 63%/57–81.57% (3); PPV: 59%/41.64–72% (3); Sensitivity: 34%/16–52% (2); Specificity: 82%/69–95% (2)
Anxious symptomatology (higher)	1652	5/7	11/3–81	OR: 1.04 (1); HR: 1.08 (1)	6/0/1	Accuracy: 90.7% (1); AUC: 67.8%/61–80% (4); *R*^2^: 79.7% (1); Sensitivity: 89% (1); Specificity: 92% (1)
*Schizophrenia-Spectrum and Other Psychotic Disorders (Schizophrenia, Schizophrenia-Spectrum Disorders, FEP), adults*						
*Illness Factors*						
Psychotic positive symptomatology (higher)	1003	6/6	8/3–31	OR: 1.69 (1)	5/1/0	AUC: 68.5%/64–73% (2); Sensitivity: 74%/71–77% (2); Specificity: 51.5%/45–58% (2)
Depressive symptomatology (lower)	443	2/2	22/13–31	OR: 1.49/1.47–1.49 (2)	2/0/0	*R*^2^: 14% (1)
Number of previous episodes (higher)	334	2/2	4/3–5	NA	1/1/0	Accuracy: 63.8% (1); Sensitivity: 71% (1); Specificity: 44.8% (1)
Global severity symptomatology (higher)	464	2/2	12/12–13	OR: 1.24/1.09–1.4 (2)	2/0/0	AUC: 73% (1); Sensitivity: 77% (1); Specificity: 58% (1)
*Substance-Related and Addictive Disorders (Alcohol, Cannabis, Cocaine, Opioid, Tobacco, Substance Use Disorder), adults*						
*Patient Factors*						
Marital status (unmarried)	15,475	15/16	9/3–28	OR: 2.38 (1); HR: 2.63/2.56–2.63 (2); Beta: 0.24 (1)	16/0/0	*R*^2^: 13.3% (1)
Education (lower)	13,352	12/14	9/5–23	HR: 2.5/2.43–2.56 (2)	14/0/0	*R*^2^: 33.4% (1)
“Self–efficacy” (lower)	404	3/3	11/3–23	OR: 1.25/1.20–1.28 (2)	3/0/0	*R*^2^: 13.3% (1)
*Illness Factors*						
Substance use symptoms (higher)	14,313	30/47	5/2–28	OR: 1.11/1.01–3.64 (10); HR: 2.63/1.06–3.7 (5); RR: 2.64 (1); Beta: 0.05/–0.15–0.06 (3)	47/0/0	AUC: 57% (1); *R*^2^: 30.4%/12.5–55% (5)
Depressive symptomatology (higher)	762	5/6	6/5–10	OR: 1.04/1.04–1.06 (3)	6/0/0	*R*^2^: 30.4%/12.5–37.6% (5)
Global severity symptomatology (higher)	647	5/5	7/2–10	OR: 1.77/1.04–2.5 (2)	5/0/0	*R*^2^: 24.3%/11–37.6% (2)
Suicidal behavior/self-harm (yes)	575	4/5	6/5–10	OR: 51/2.1–100 (2)	4/0/0	*R*^2^: 16.4%/12.5–37.6% (3)

*AUC* area under the curve, *D* development sample, *E* external validation sample, *HR* hazard ratio, *I* internal validation sample, *k* number of studies, *N* number of subjects, *NA* not available, *NPV* negative predictive value, *PPV* positive predictive value, *RR* relative risk.

^a^In brackets value of predictor associated with relapse.

^b^Coefficient of predictors significantly associated with outcome in multivariable models.

^c^Studies might have reported more than one accuracy measure, so the number of studies can exceed *k.*

The operationalizations of outcomes are reported in eTable 6.

### Non-modifiable and modifiable MCPs of treatment response

All MCPs of treatment response are related to depressive disorders only. Non-modifiable, patient-related MCPs included female sex and presence of specific polymorphisms in the 5-HT transporter gene (adults).

Modifiable, patient-related MCPs of treatment response in depressive disorders included being married and absent negative life events (adults). Modifiable, illness-related MCPs of treatment response in depressive disorders included lower depressive symptoms (both in elderlies and in adults) and higher functioning (adults). Finally, the only modifiable, intervention-related MCP in depressive disorders was early treatment response. Additional information is available in Table 1. Additional broadly-defined predictors of response are reported in eTable 7, that do not meet the proposed “credibility” criteria for MCPs.

### Non-modifiable and modifiable MCPs of remission

Non-modifiable, patient-related MCPs of remission included only sex (females in adults and males elderlies) in depressive disorders as well as in schizophrenia-spectrum (females) and substance use disorders (females).

Modifiable illness-related MCPs of remission included lower anxious symptomatology (children/adolescents) in anxiety disorders.

Modifiable patient-related MCPs of remission in depressive disorders included absent negative life events, higher education, higher use of emotion regulation and coping strategies, being employed, higher quality of life (all in adults), social contacts (elderlies), and better physical health (both in adults and elderlies). Modifiable illness-related MCPs of remission in depressive disorders were absence of anxiety, OCD, PTSD, and personality disorder comorbidities, lower global severity (all in adults), higher functioning (elderlies), lower depressive symptomatology (children/adolescents and adults), and a lower episode/hospitalization duration (adults and elderlies).

Modifiable illness-related MCPs of remission in eating disorders were higher weight, shorter illness duration, and lower eating disorder symptomatology (adults).

Modifiable patient-related MCPs of remission in schizophrenia-spectrum disorders, included higher quality of life and education (adults). Additionally, modifiable illness-related MCPs of remission in schizophrenia-spectrum disorders included shorter duration of untreated illness, lower negative and positive psychotic symptomatology, higher functioning and higher depressive symptomatology (adults).

Patient-related MCPs in substance use disorders included being married and higher education (adults). Finally, illness-related MCPs in substance use disorders included absence of lifetime comorbidity of other substance use disorders and presence of depressive comorbidities (adults). Additional information is available in Table 1. Additional broadly-defined predictors of remission are reported in eTable 8, that do not meet the proposed “credibility” criteria for MCPs.

### Non-modifiable and modifiable MCPs of relapse

Non-modifiable, patient-related MCPs of relapse in adults included male sex for bipolar disorders, a psychiatric family history and younger age for depressive disorders, and younger age for substance use disorders. Non-modifiable, illness-related MCPs of relapse in adults included only younger age at illness onset and lower gray matter volume.

Modifiable, patient-related MCPs of relapse in anxiety disorders in adults included presence of negative life events. Modifiable, illness-related MCPs of relapse in anxiety disorders in adults were only higher anxious symptomatology.

Modifiable, patient- and illness-related MCPs of relapse in adults with bipolar disorders included lower social contacts and a higher depressive symptomatology.

The only modifiable, patient-related MCP of relapse in adults with depressive disorders included present negative life events. Modifiable, illness-related MCPs of relapse in adults with depressive disorders included higher depressive and anxious symptomatology, lower functioning and higher number of episodes.

Modifiable, illness-related MCPs of relapse in adults with schizophrenia-spectrum disorder included higher global illness and psychotic positive symptom severity, lower depressive symptoms, and a higher number of previous episodes.

Modifiable, patient-related MCPs of relapse in substance use disorders included being unmarried, lower education, and lower “self-efficacy” (adults). Finally, modifiable, illness-related MCPs of relapse in adults with substance use disorders included higher global illness, depressive, and substance use symptom severity and present suicidal behavior/self-harm. Additional information is available in Table 2. Additional broadly-defined predictors of relapse are reported in eTable 10, that do not meet the proposed “credibility” criteria for MCPs.

### Transdiagnostic MCPs

Non-modifiable transdiagnostic MCPs included the following in the respective diagnoses: sex (depressive, schizophrenia-spectrum, substance use disorders—adults) and age (depressive, substance use disorders—adults). Modifiable transdiagnostic MCPs included the following in the respective diagnoses: education (depressive, schizophrenia-spectrum, substance use disorders—adults), quality of life (depressive, schizophrenia-spectrum disorders—adults), functioning (depressive disorders—elderly, schizophrenia-spectrum disorders—adults), illness-specific symptom level (anxiety, bipolar, depressive, schizophrenia-spectrum, substance use disorders—adults), number of episodes (depressive, schizophrenia-spectrum disorders—adults), negative life events (anxiety, depressive disorders—adults) and global severity of symptoms (schizophrenia-spectrum, substance use disorders—adults). Additional information is available in Table 3, and eTable 11 (transdiagnostic MCPs).

**Table 3:** Multivariable candidate predictors of response, remission, recovery, and relapse across mental disorders across specific mental disorders.

Disorder (age group)	Response (number of predictors)	Remission (number of predictors)	Relapse (number of predictors)	Recovery (number of predictors)
Anxiety disorders (youth)	—	Lower anxious symptoms (1)	—	—
Anxiety disorders (adults)	—	—	Higher anxious symptoms, Negative life events (2)	—
Bipolar and related disorders (adults)	—	—	Male sex, lower social contacts, higher depressive symptoms (3)	—
Depressive disorders (youth)	—	Lower depressive symptoms (1)	—	—
Depressive disorders (adults)	Female sex, married marital status, no negative life events, lower depressive symptoms, presence of early treatment response at index/previous episode, higher functioning, presence of serotonin transporter polymorphism (7)	Employed status, lower depressive symptoms, female sex, no negative life events, higher education, lower episode/hospitalization duration, no organic comorbidities/good general physical health, no anxiety comorbidity, no OCD comorbidity, no PTSD comorbidity, no personality disorder comorbidity, lower global severity symptoms, higher quality of life, higher emotional regulation/coping strategies (14)	Negative life events, higher depressive symptoms, higher number of previous episodes, presence of psychiatric family history, younger age, higher anxious symptoms, lower functioning (7)	—
Depressive disorders (elderly)	Lower depressive symptoms (1)	Higher social contacts, male sex, no organic comorbidities/good general physical health, no anxiety comorbidity, lower episode/hospitalization duration, higher functioning (6)	—	—
Feeding and eating disorders (adults)	—	Higher weight, lower eating disorder symptoms, shorter duration of illness (3)	—	—
Schizophrenia-spectrum disorders (adults)	—	Lower psychotic negative symptoms, lower psychotic positive symptoms, higher quality of life, female sex, higher education, shorter duration of untreated illness, higher depressive symptoms, higher functioning (8)	Higher psychotic positive symptoms, lower depressive symptoms, higher number of previous episodes, higher global severity symptoms (4)	—
Substance use disorders (adults)	—	Female sex, married status, higher education, no other substance use disorder lifetime comorbidities, presence of depressive comorbidity (5).	Younger age, lower education, unmarried status, higher substance use symptoms, younger age at onset, higher depressive symptoms, higher global severity symptoms, presence of suicidal behavior/self-harm, lower self-efficacy, lower gray matter volume (10)	—

### Trans-outcome MCPs within or across mental disorders

Non-modifiable MCPs included female sex in depressive disorders as the sole MCP for multiple outcomes (response and remission). Modifiable, trans-outcome MCPs in depressive disorders included depressive symptoms (response, remission, relapse), negative life events (response, remission, relapse) and functioning (higher = response, lower = relapse). Modifiable, trans-outcome MCPs in schizophrenia-spectrum disorders included more depressive symptoms and less positive psychotic symptoms for remission and the reverse for relapse. Finally, modifiable, trans-outcome MCPs in substance use disorders included marital status and education level were for remission and relapse. Additional information is available in Table 3, and eTable 12 (trans-outcome MCPs).

## Discussion

This umbrella review summarized evidence from 117 systematic reviews, including 403 individual studies and 299,888 persons with mental disorders, testing multivariable models to predict treatment response, or illness remission, recovery, or relapse. We showed that the field has relevant methodological limitations, with only 4.3% of systematic reviews having high quality and only 1.2% of models in individual studies having low risk of bias. The most frequently studied outcomes were remission (37%) and relapse (35%), with more limited evidence for response (17%) and with especially little evidence for clinically highly relevant recovery (3%). We filtered the most promising MCPs that should be considered as candidates to refine multivariable models in further studies. We here discuss main findings and provide additional discussion and literature context in the eDiscussion.

This umbrella review identified female sex and older age as both non-modifiable transdiagnostic MCPs of positive clinical outcomes. Female sex was a MCP of better clinical outcome across mood disorders, schizophrenia-spectrum disorders, and substance use disorders. Female sex was also a transoutcome MCP, being identified for both response and remission in depressive disorders. Biological, psychological, and social factors could underlie the protective role of female sex. For instance, XX vs XY chromosomes have been associated with better neuroplasticity [24] and less pro-inflammatory status [25–27], which are adverse characteristics associated with mental disorders [28, 29]. Women have shown higher levels of resilience [30], and might have better social cognition [31] and functioning, which could further contribute to the better outcomes.

Older age predicting better outcomes across diagnostic boundaries corroborates the importance of prevention and early and optimized intervention to improve outcomes in individuals with mental disorders [32–37], in particular at a young age. Importantly, the peak onset of mental disorders is even before age 18 [1]. Intervening early is important to reduce as much as possible the duration of the mental disorder episode and its biopsychosocial collateral damages, including that a depressive disorder or substance use disorder contributes to an accumulation of additional predictors of poor outcomes, including more severe mental disorders symptoms, poor functioning, poor quality of life, drop from education, unemployment, not being married, poor physical health and substance use [38].

Moreover, younger age at onset of substance use disorders predicted greater relapse risk, possibly via a negative impact of substance use in early age on functioning, mental and physical health [39], including decreased gray matter, ultimately decreasing chances of response to available treatments, and affecting education [40]. Indeed, education, a transdiagnostic modifiable MCP of good outcomes, can be jeopardized by mental disorders, which are frequently present among high school, and university [41] students.

The 5-HT transporter polymorphisms predicting better treatment response in depressive disorders supports the serotonin hypothesis of depression [42], which, however, requires to be updated involving more complex mechanisms and additional neurotransmitters.

Family history of mental disorders not only increases the risk of mental disorders, but also increases chances of relapse of depression [43], calling for close monitoring and potentially selective relapse prevention strategies.

Despite depression being more frequent in women than men [44, 45], female sex was a consistent positive prognostic factor across several mental disorders as mentioned above. However, male sex selectively predicted better outcome in elderlies with depression. A possible explanation for this finding could be related to menopausal hormonal changes in females [46], and other psychosocial factors, including support by females, whereas elderly females more often than males age without a partner due to their increased longevity relative to males [47].

Among MCPs, illness symptom severity was a replicated transdiagnostic and trans-outcome characteristic. Fewer symptoms of anxiety and depression predicted greater remission in each disorder, and more symptoms of depression predicted depression relapse, while more positive and negative symptoms predicted schizophrenia relapse. This finding relates to the potential of early detection and treatment before symptoms reach their peak and become enduring, as well as for the implementation of measurement-based effective and adequately dosed psychosocial and pharmacologic treatments, which could minimize (residual) symptoms and reduce the likelihood of the emergence of other related candidate predictors, i.e., more illness episodes and unemployment in depression, and lower quality of life in schizophrenia (where a better quality of life was related to a greater likelihood of remission). Shortened but validated versions of longer scales, such as PANSS-6 for schizophrenia [48], or self-report measures of symptoms, such as the Patient Health Questionnaire 9 items for depressive [49] or Generalized Anxiety Disorder 7 items for anxiety disorders [50], can facilitate measurement-based care. Moreover, self-report questionnaires can be easily implemented in electronic medical software, with the additional benefit of engaging and educating patients to self-monitor symptoms. The shift to virtual care in psychiatry that occurred during the COVID-19 pandemic might have made the integration of symptom measurements into clinical practice easier [51].

Depressive symptoms were modifiable, illness-related MCPs of relapse not only in depressive, but also in bipolar and substance use disorders. In bipolar disorder, depressive symptoms are commonly responsible for the largest proportion of non-euthymic mood states [52], and are also responsible for longer duration of untreated illness [52], and inter-episodic impairment in functioning [53]. A factor contributing to inappropriate treatment of bipolar disorder is a misdiagnosis leading to use of antidepressants [54], as opposed to using other effective treatments, including quetiapine or lurasidone among others [55, 56], or poor adherence to medications, which in turn is associated with depressive symptoms [57]. In substance use disorders, comorbid depressive disorder or depressive symptoms are highly prevalent [58, 59], and should be treated as in patients without substance use disorders [60–62].

Less anxiety symptoms and/or comorbidity were also MCPs of remission and less relapse in anxiety and depressive disorders, calling for careful diagnostic assessment excluding a diagnosis of bipolar depression (anxiety features are frequent in bipolar depression), and calling for effective treatments targeting depressive and anxiety symptoms.

More prior illness episodes were another modifiable illness-related MCP for both depressive and schizophrenia-spectrum disorders, calling for early multimodal interventions at effective and tolerable doses since early stages of illness [17, 33].

As we and others have shown in several other meta-research projects focusing on risk factors for mental disorders [63–67], one key transdiagnostic MCP in this umbrella review was negative life events, including childhood abuse [68]. According to findings from this and previous umbrella reviews and meta-umbrella reviews that we and other groups conducted on risk factors of all mental disorders [68–71], negative life events, including childhood abuse, not only is the most transdiagnostic risk factor increasing the risk of multiple mental disorders, but also impacting their long-term outcomes, resulting in major health inequity and individual as well as societal costs. Thus, prevention of negative life events and, especially, of abuse and neglect during childhood, plus increasing resilience factors are key, both globally but especially in populations with an increased risk for mental disorders, as not only the emergence of mental illness but also worse outcomes within mental disorders are more likely among those with early negative life experiences [72, 73].

Moreover, the fact that quality of life was a MCP of remission in schizophrenia highlights that subjective wellbeing and patient-reported outcomes and goals beyond symptoms, relapse, including functional level, need to be considered and targeted to improve outcomes.

Additional MCPs were identified within specific mental disorders. Beyond better functioning, MCPs increasing chances of response or remission, or lowering the risk of relapse in depressive disorders were being employed, and having less psychiatric and physical comorbidities, confirming the close interplay between physical and mental health [2], and calling for remediating disparities in quality of physical healthcare in those with mental disorders [74]. Poor emotional regulation predicting worse outcomes might indicate the need to rule out borderline personality disorder before treating depression. Preventing social isolation and loneliness, which are a common and increasing problem in the elderlies [75], could improve outcomes since social contacts emerged as a MCP of remission in depressive disorders. Lower duration of episodes should be achieved by offering biological and psychosocial first-line treatments to all patients with depression, including exercise with its pleiotropic beneficial effect, and by offering early switch to second-line antidepressants when first-line treatment is ineffective.

While lower depressive symptoms were a transdiagnostic predictor of good outcomes, conversely, lower depressive symptoms seemed to predict relapse in schizophrenia. From a phenomenological perspective, higher depressive symptoms might indicate lower severity of flat affect and negative symptoms [76], with more negative symptoms having been associated with poorer outcomes [77–79]. Nevertheless, depression is frequently comorbid in those with schizophrenia [80], and, if left untreated, depressive symptoms could also worsen prognosis, including suicide mortality. Hence, medications, other biological treatments, and psychosocial treatments that are not only effective and safe for disease-specific symptoms, i.e, positive and negative symptoms, but that can also improve mood should be offered [33, 81–85], and/or treatment of comorbid depressive symptoms or disorder with antidepressants should be offered to persons with schizophrenia.

In addition to the importance of treating depressive symptoms or disorders, self-injurious behaviors should be prevented and self-efficacy should be promoted [86] to improve outcomes in substance use disorders. Self-injurious behavior might be a marker of depressive symptoms, or borderline personality traits or disorders, which might complicate the response to standard treatments for substance use disorder. In case that comorbid borderline personality disorder underlies impaired self-efficacy that can increase relapse in substance use disorder, psychological treatment should be offered to optimize outcomes in people with substance use disorder [87].

Finally, in eating disorders, in addition to body weight and eating disorder-specific symptoms, illness duration seems to predict poor outcome. It is important that effective treatments for eating disorders are provided as early as possible [88], and that services account for the early age at onset of eating disorders, which occurs in almost 50% of patients before age 18 [1].

Unfortunately, there was a paucity of data in the area of predictors for recovery. Still, low symptom levels and symptomatic stability should be explored as one relevant predictor for achieving recovery, as has been demonstrated in patients with first-episode schizophrenia previously [89].

Results from this umbrella review provide several specific leads and recommendations for additional research on predicting outcomes across mental disorders. First, future studies should use a more homogeneous definition of predictors to facilitate evidence synthesis projects that can further inform future multivariable models on the prediction of outcomes. The preponderance of studies in individuals with depression may, at least in part, be driven by the fact that in depression, definitions of response and remission and the related scales used for these definitions had been proposed early and accepted and adopted widely. Second, whenever sample size allows, studies should focus on more outcomes that could be predicted, primarily, as response, remission, and recovery are related to one another clinically. Third, transdiagnostic predictors could also optimize scalability, as it is more feasible to collect the same set of predictors that can be clinically meaningful across multiple disorders, as opposed to collecting different predictors based on the primary diagnosis. Moreover, mental disorders are frequently comorbid with other mental disorders, and the prognosis of each disorder can contribute to the overall well-being and quality of life of persons with mental disorders. Fourth, identifying trans-outcome predictors might also increase efficiency in research and clinical care, provided that these predictors could be assessed at scale.

This study also has several limitations. First, when presenting results, we reduced the granularity of predictors for feasibility and knowledge translation considerations. However, this umbrella review should serve as a starting point to inform future studies testing literature-informed candidate predictors rather than being used as a clinical guide. Unfortunately, the high risk of bias in virtually all eligible studies precludes clinical implementations of any reported predictors at this stage of research knowledge. Second, since our search key focused on systematic reviews, as this is an umbrella review, we may have missed individual studies that could have been published since the most recent systematic review for each combination of populations, interventions, controls, and outcomes. However, this is the first umbrella review on MCPs of response, remission, recovery, and relapse, which can also identify where the first or an updated systematic review is needed. Third, methodological decisions on how to label predictors and outcomes were made after data extraction, given the large and heterogeneous body of included evidence, as detailed in the methods section, to translate data into information. Fourth, it was not always sufficiently clear whether the MCPs studied in at least two studies were significantly associated with the outcome of interest, and if they were, in which direction, as reporting was often poor, and mainly focused on model performance rather than on individual factors. Fifth, the criteria we applied to identify MCPs were arbitrary but validated via simulations. Sixth, many mental disorders had only limited published MCP evidence available, calling for more research and appropriate funding to conduct such research in sufficiently large samples with enough patients per tested variable in the multivariable analyses. Seventh, information regarding candidate prognostic and predictive factors that significantly affect treatment response and, especially, recovery was mainly missing, calling for more research attention to this area. Eighth, many predictors were only tested in a few studies with small samples, and some models might have suffered from overfitting. Ninth, the effect of treatments, comorbid disorders, and usual illness trajectory on the predictors and outcomes could not be measured. Future studies should attempt to delineate those factors more clearly. Tenth, the difference between “interventions” and “predictive factors” is sometimes debatable, i.e., some researchers may consider that antipsychotic dose reduction is a potential predictive factor. In contrast, others may consider that dose reduction is an intervention. Finally, we focused on individual predictors extracted from multivariable models. Still, their performance depended on other model variables, and future research may identify several significant predictors performing even better when combined. Therefore, validation studies testing these candidate predictors within and across different populations and models will be important.

In conclusion, despite the limitations of this work and the available literature, this umbrella review for the first time scrutinized the level of evidence for MCPs of response, remission, recovery, and relapse across mental disorders and outcomes, identifying MCPs across mental disorders and outcomes, and calling out numerous areas that need further investigation. Future studies should replicate broadly defined MCPs of major clinical outcomes identified by this study, and consider them to refine existing or build even superior multivariable models.

### Supplementary information

Supplementary material

## Data Availability

The whole dataset is available from authors upon request.
