# Supplementary material for: An umbrella review of candidate predictors of response, remission, recovery, and relapse across mental disorders
Source: Mol Psychiatry. 2023 Nov 13;28(9):3671–87. doi: 10.1038/s41380-023-02298-3 (PMC10730397; doi:10.1038/s41380-023-02298-3)
Supplement: Supplementary file 1 — Supplementary material [file 41380_2023_2298_MOESM1_ESM.docx]

**Supplementary material**

**An umbrella review of candidate predictors of Response, Remission, Recovery, and Relapse across Mental Disorders**

Marco Solmi*^1,2,3,4,5^, Samuele Cortese*^6,7,8,9,10,11^, Giovanni Vita*^12^, Michele De Prisco*^13,14,15^, Joaquim Radua^16^, Elena Dragioti^17,18^, Ole Köhler-Forsberg^19,20^, Nanna M. Madsen^19,20^, Christopher Rohde^20,21^, Luis Eudave^22^, Claudia Aymerich^23^, Borja Pedruzo^24^, Victoria Rodriguez^25^, Stella Rosson^26^, Michel Sabé^27^, Mikkel Hojlund^28,29,30^, Ana Catalan^23^, Beatrice de Luca^12^, Michele Fornaro^31^, Giovanni Ostuzzi^12^, Corrado Barbui^12^, Gonzalo Salazar-de-Pablo^32,33,34^, Paolo Fusar-Poli^25,35,36,37^, Christoph U Correll ^1,38,39,40^

1 Charité Universitätsmedizin Berlin, Department of Child and Adolescent Psychiatry, Berlin, Germany

2 Department of Psychiatry, University of Ottawa, Ontario, Canada.

3 On Track: The Champlain First Episode Psychosis Program, Department of Mental Health, The Ottawa Hospital, Ontario, Canada.

4 Ottawa Hospital Research Institute (OHRI) Clinical Epidemiology Program University of Ottawa Ottawa Ontario

5 School of Epidemiology and Public Health, Faculty of Medicine, University of Ottawa, Ottawa, Canada

6 Centre for Innovation in Mental Health, School of Psychology, Faculty of Environmental and Life Sciences, University of Southampton, Southampton, UK;

7 Clinical and Experimental Sciences (CNS and Psychiatry), Faculty of Medicine, University of Southampton, Southampton, UK

8 Solent NHS Trust, Southampton, UK;

9 Hassenfeld Children’s Hospital at NYU Langone, New York University Child Study Center, New York, NY, USA;

10 Division of Psychiatry and Applied Psychology, School of Medicine, University of Nottingham, Nottingham, UK;

11 DiMePRe-J-Department of Precision and Regenerative Medicine-Jonic Area, University of Bari “Aldo Moro”, Bari, Italy.

12 Department of Neuroscience, Biomedicine, and Movement Sciences, Section of Psychiatry, University of Verona, Verona, Italy

13 Bipolar and Depressive Disorders Unit, Hospìtal Clinic de Barcelona. c. Villarroel, 170, 08036 Barcelona, Spain;

14 Institut d’Investigacions Biomèdiques August Pi i Sunyer (IDIBAPS), c. Villarroel, 170, 08036 Barcelona, Spain;

15 Centro de Investigación Biomédica en Red de Salud Mental (CIBERSAM), Instituto de Salud Carlos III, Madrid, Spain

16 Institut d’Investigacions Biomèdiques August Pi i Sunyer (IDIBAPS), Imaging of Mood- and Anxiety-Related Disorders (IMARD), CIBERSAM, University of Barcelona, Barcelona, Spain

17 University of Ioannina, Research Laboratory Psychology of Patients, Families & Health Professionals, Department of Nursing, School of Health Sciences, Ioannina, Greece

18 Linköping University, Pain and Rehabilitation Centre and Department of Health, Medicine and Caring Sciences, Linköping, Sweden

19 Psychosis Research Unit, Aarhus University Hospital Psychiatry, Aarhus, Denmark

20 Department of Clinical Medicine, Aarhus University, Aarhus, Denmark

21 Department of Affective Disorders, Aarhus University Hospital - Psychiatry, Aarhus, Denmark

22 Faculty of Education and Psychology, University of Navarra, Pamplona, Spain

23 Biobizkaia Health Research Institute. Basurto University Hospital, OSI Bilbao-Basurto. University of the Basque Country UPV/EHU. Centro de Investigación en Red de Salud Mental. (CIBERSAM), Instituto de Salud Carlos III. Plaza de Cruces 12. 48903. Barakaldo. Bizkaia. Spain

24 Psychiatry Department, Basurto University Hospital, Bilbao, Spain

25 Department of Psychosis Studies, King's College London, UK

26 Mental Health Department, Local Health Unit ULSS3 Serenissima, Venice, Italy

27 Division of Adult Psychiatry, Department of Psychiatry, University Hospitals of Geneva, 2, Chemin du Petit-Bel-Air, CH-1226, Thonex, Switzerland;

28 Department of Psychiatry Aabenraa, Mental Health Services Region of Southern Denmark, Aabenraa, Denmark

29 Clinical Pharmacology, Pharmacy, and Environmental Medicine, Department of Public Health, University of Southern Denmark, Odense, Denmark

30 Child and Adolescent Mental Health Centre, Mental Health Services Capital Region of Denmark, Copenhagen, Denmark

31 Department of Psychiatry, Federico II of Naples, Italy

32 Department of Child and Adolescent Psychiatry, Institute of Psychiatry, Psychology & Neuroscience, King’s College London UK;

33 Child and Adolescent Mental Health Services, South London and Maudsley NHS Foundation Trust, London, UK

34 Institute of Psychiatry and Mental Health. Department of Child and Adolescent Psychiatry, Hospital General Universitario Gregorio Marañón School of Medicine, Universidad Complutense, Instituto de Investigación Sanitaria Gregorio Marañón (IiSGM), CIBERSAM, Madrid, Spain

35 Department of Brain and Behavioral Sciences, University of Pavia, Italy

36 Outreach and Support in South London (OASIS) service, NHS South London and Maudsley Foundation Trust, UK

37 Department of Psychiatry and Psychotherapy, Ludwig-Maximilian-University Munich, Munich, Germany.

38 The Zucker Hillside Hospital, Northwell Health, New York, USA

39 Donald and Barbara Zucker School of Medicine at Hofstra/Northwell, New York, USA

40 The Feinstein Institute for Medical Research, Center for Psychiatric Neuroscience, Manhasset, NY, USA

* M Solmi, S Cortese, G Vita, and M De Prisco contributed equally to this article.

Corresponding author: Christoph U Correll; Charité Universitätsmedizin - Berlin, Department of Child and Adolescent Psychiatry, Augustenburger Plarz 1, 13353 Berlin, Germany ; christoph.correll@charite.de

**Protocol amendments**

To filter the evidence on all published level II predictors, we applied the definition of broadly defined and multivariable candidate predictors (MCPs) as specified in the methods and specified below:

As we expected studies to report on a large number of MCPs, we had a three-tiered approach to the selection and evidence grading of published MCPs for this umbrella review. First, from the overall set of “published predictors” reported in the literature, we identified “broadly defined” level II (meso-level) predictors from multivariable models that i) were tested in at least two studies, and ii) in specific spectra of mental disorders (i.e., not in mixed categories of mental disorders). In the next step, to fulfill criteria for an MCP, we required iii) at least two significant findings without more than 20% of the significant associations going in the opposite or unknown direction (e.g., male and female sex both associated with relapse), and iv) reporting of model performance.

To test whether the criteria i)-iv) for CPs were sensitive and specific, we conducted simulation analyses to measure the sensitivity and specificity of the proposed set of criteria (for definitions, see below).

**eMETHODS**

*Search strategy, inclusion, and exclusion criteria*

A more comprehensive version of the methods is available in eMethods. We conducted an umbrella review of systematic reviews with or without meta-analysis, including observational or interventional studies testing predictors of response, remission, recovery, and/or relapse in people with mental disorders. We followed an a-priori protocol (<https://osf.io/5wbsv/>), and used PRIOR (supplementary material)(1). We searched MEDLINE and Embase up to July 19^th^, 2022. (search key in supplementary material).

Inclusion criteria were: i) systematic reviews defined as a review that a) searched ≥2 databases, b) made the search key available, c) specified inclusion and/or exclusion criteria of the individual studies, and d) reported details of included studies, which ii) were longitudinal (randomized) controlled trials (RCTs), open-label studies, or cohort studies; iii) included individuals with mental disorders defined according to Diagnostic and Statistical Manual(2), any version, or the International Classification of Diseases, any version(3); iv) reported on multivariable models (i.e., including at least two variables); and v) tested prognostic or predictive factors; vi) of study-defined response, remission, recovery, and/or relapse. Therefore, inclusion criteria were only applied in full to the individual included studies and not to the inclusion or exclusion criteria of the systematic review that may have been more inclusive.

Exclusion criteria were: i) pooled analyses that summarized a selected number of (R)CTs without conducting a systematic search, unless these pooled analyses were included in a systematic review; ii) individual studies that were not included in systematic reviews (i.e., we searched the literature for systematic reviews, not for individual studies), iii) that included people with mental disorders defined according to questionnaires, scales, or any other tool not allowing a diagnosis as per DSM or ICD criteria; iv) that tested univariable models or reported correlation analyses; v) that focused on cross-sectional markers instead of prognostic/predictive factors (e.g., brain imaging markers in responders versus non-responders, case-control studies); or vi) that did not report on the outcomes of interest.

Notably, we conducted the search at the systematic review level to identify individual studies matching inclusion criteria. Still, we ultimately included and extracted data from eligible individual studies included in the eligible systematic reviews. The literature screening was performed at two levels by two independent authors (CA, AC, BDL, MDP, LE, OKF, MF, CGR, NMM, MH, BP, VR, SR, GSDP, MS, GV). First, two authors independently assessed the eligibility of systematic reviews. Then each study included in the eligible systematic reviews was screened against eligibility criteria. Hence, we only included studies meeting eligibility criteria from the overall list of studies included in a systematic review. If, in a systematic review, no individual study was eligible, we excluded that systematic review. Inconsistencies were resolved by consensus or with a third author (CUC, MS).

**Prognostic and predictive factors**

While it is often difficult to disentangle whether a factor has been tested as a prognostic or a predictive factor(4), differences between the two exist. Prognostic factors are defined as factors forecasting a given outcome, independent of the effect of any or specific intervention(5–7). Conversely, predictive factors are defined as factors predicting a given outcome in the context of a specific intervention (of any type), compared with a control group. Prognostic factors are typically investigated in observational studies (cohort studies in this project)(5,6). Still, they can also be studied in interventional studies when a control group is not accounted for in the analyses. Predictive factors are investigated in studies that specifically measure outcomes of an intervention, typically in (R)CT, but also in some longitudinal studies, provided that a control group is present (cohort studies)(8). Also, predictive factors can be investigated if a specific class of intervention or a particular intervention is reported (e.g., CBT, antipsychotics, fluoxetine) in the experimental/exposed and control/non-exposed group, and if the non-exposed/control group is accounted for in the analyses. In this umbrella review, prognostic factors were evaluated in studies that used “treatment as usual”, or generically a “pharmacological treatment”, or “psychological treatment”, or in studies that reported on the association between a (set of) factor(s) within one treatment group only. This operationalization was chosen to account for studies where a specific intervention and/or a control group was missing and the outcome could be seen as being related to usual care that may or may not be received and that changes over time, with the outcome reflecting a “usual illness trajectory”.

**Data extraction**

We extracted data at two levels: first, at the systematic review level, and then, after having identified eligible individual studies within eligible systematic reviews, at the individual study level. Data from each systematic review and individual study were extracted by two authors independently, reciprocally checking the quality of the data extraction (MDP, MH, MS, GV), with two further authors resolving conflicts and performing further quality checks as needed (CUC, MS).

From each included systematic review, we extracted the digital object identifier (DOI) or PubMed identifier (PMID), first author’s last name, year of publication, number of studies originally included in the systematic review, mental disorders considered, and outcomes of interest.

From each study, we extracted the DOI or PMID, first author’s last name, publication year, country, study design, patient diagnosis, diagnostic criteria, setting (inpatient, outpatient, mixed), mean patient age and range, follow-up duration in weeks, sample size, type of exposure/intervention and control, authors’ outcome definition (i.e., criteria defining the outcome) and its operationalization (i.e., response, remission, recovery, relapse), all predictors in each multivariable model, the total number of predictors, frequency of the outcome event, the statistical approach employed (e.g., regression analysis, machine learning), each predictors’ coefficient and p-value, and performance metrics of the multivariable model.

**Quality of eligible systematic reviews**

The same authors conducting the literature screening assessed the quality of eligible systematic reviews, using A MeaSurement Tool to Assess systematic Reviews, version 2 (AMSTAR 2)(9), checked by a third author. AMSTAR 2, composed of 16 items, assesses the quality of systematic review via 16 items, categorizing the quality of systematic review based on weakness in critical domains into high, moderate, low, or critically low. AMSTAR 2 is not intended to generate an overall score.

**Risk of bias of individual studies**

The same authors conducting the data extraction of individual studies also assessed the risk of bias of individual studies using the PROBAST(10), which measures the risk of bias of systematic reviews or of individual studies developing, validating, or updating prediction models, with 20 questions across four domains, namely participants, predictors, outcome, and analysis.

**Definition of predictors**

From the overall set of “published” predictors reported in the literature, we identified multivariable MCPs fulfilling all of the following criteria: i) tested in at least two studies, ii) in specific spectra of mental disorders (i.e., not in mixed categories of mental disorders), iii) at least two significant findings without more than 20% of the significant associations going in the opposite or unknown direction (e.g., male and female sex both associated with relapse), and iv) reporting of model performance. Additional types of predictors which satisfied both criteria i) and ii) only, were labeled as "broadly-defined" predictors, and are reported in the supplementary material.

Predictors were also classified into modifiable or non-modifiable, and into patient-, ilnness-, or treatment-related factors.

**eMethods of simulation analyses to explore the validity of criteria used for the definition of multivariable candidate predictors**

We conducted the following exploratory simulation analyses to explore the validity of the definition of MCPs. In each simulation, we first simulated ten variables:
Variable 1 predicts the outcome and is not correlated with any other variable.
Variable 2 predicts the outcome and correlates with variable 3.
Variable 3 predicts the outcome and correlates with variable 2.
Variable 4 predicts the outcome and correlates with variable 5.
Variable 5 does not predict the outcome and correlates with variable 4.
Variable 6 does not predict the outcome and correlates with variable 7.
Variable 7 does not predict the outcome and correlates with variable 6.
Variables 8-10 do not predict the outcome and are not correlated with other variables.
Afterward, we created prediction models (multiple regressors) and derived how many times a variable predicted the outcome (and the same vs. opposite direction).

Finally, we applied the criteria and saved how many times the variables that should predict the outcome were detected as MCPs (sensitivity) and how many times the variables that should not predict were not detected as MCPs (specificity).

**Patient and public involvement**

Patient and public have not been included in this project.

**eRESULTS**

**Search results and characteristics of systematic reviews and individual studies of published predictors**

Out of the 2,742 studies identified in the literature search, we excluded 2,077 based on the title and abstract, whilst reading the full text of 665 reviews. Of these, we excluded 548 reviews because they did not report any outcome of interest (N=137), did not have includible individual studies (N=117), not eligible review design (N=93), did not investigate prognostic or predictive factors (N=76), reported univariable models or correlational studies (N=64), had searched only one database (N=41), reported on an ineligible patient population (N=16), or did not include baseline factors (N=4). We ultimately included 117 systematic reviews that provided 403 eligible unique individual studies, including overall 299,888 persons with mental disorders. The study selection flow-chart is reported in Figure 1. The list of excluded studies after full-text assessment is in the eTable 1.

Across eligible systematic reviews, the median of included studies was 4.4 (range: 1-62), pooling together a median sample size of 316 (range 23-159,299) individuals and predictors across a median of 3 (range: 1-9) level III (macro-level) domains. More details on the eligible systematic reviews are reported in eTable 2.

The list of individual studies included in eligible systematic reviews and meeting inclusion criteria of providing information at a minimum at the published predictor level are reported in eTable 3. Individual studies were conducted in the U.S. (N=157), Netherlands (N=36), Germany (N=32), U.K. (N=19), multiple Countries (N=17), China (N=15), Canada (N=14), France (N=12), Italy (N=9), Spain (N=9), Sweden (N=8), South Korea (N=7), Turkey (N=7), Australia (N=5), Denmark (N=5), Poland (N=5), Taiwan (N=5), Brazil (N=4), India (N=4), Japan (N=4), Switzerland (N=4), Austria (N=3), Czech Republic (N=3), Ireland (N=3), Norway (N=3), Mexico (N=2), Portugal (N=2), South Africa (N=2), Estonia (N=1), Israel (N=1), Malawi (N=1), Malaysia (N=1), New Zealand (N=1), Saudi Arabia (N=1), and Singapore (N=1). The distribution of individual studies across the globe is visualized in eFigure 1. Most of the studies included adults (N=311, 77.2%), with some focusing on elderlies (N=40, 9.9%), children or adolescents (N=36, 8.9%), or mixed age groups (N=16, 3.9%).

At the published predictor level, most individual studies focused on depressive disorders (N=159), followed by substance-related addictive disorders (N=72), schizophrenia-spectrum disorders and other psychotic disorders (N=48), mixed categories disorders (N=35), feeding and eating disorders (N=29), bipolar and related disorders (N=26), anxiety disorders (N=21), obsessive-compulsive disorders (N=7), neurodevelopmental disorders (N=4), or trauma and stress-related disorders (N=1), and personality disorders (N=1).

Among eligible individual studies, 285 (70.8%) were cohort studies, 85 (21.1%) RCTs, 24 (5.9%) were open-label studies, and 9 (2.2%) were non-randomized controlled trials.

The vast majority of studies (372, 92.3%) tested prognostic factors, 19 (4.7%) predictive factors, and 12 (3%) tested both.

Overall, 149 (36.9%) studies considered remission as an outcome, 142 (35.2%) relapse, 68 (16.8%) response, 10 (2.5%) recovery, and 34 (8.4%) studies considered multiple outcomes.

**Quality of included systematic reviews and individual studies**

According to AMSTAR2, only five (4.3%) systematic reviews were of high quality, 10 (8.5%) had moderate, 23 (19.7%) low, and 79 (67.5%) had critically low quality.

The risk of bias in individual studies was deemed high in 98.8% of the studies. The “participants” PROBAST domain had the lowest risk of bias (only 1.2% having high or unclear risk of bias). Conversely, the “analysis” domain had the highest risk of bias, with 97.7% of studies being at high risk of bias (Figure 2).

**eResults of simulation analyses to explore the validity of criteria used for the definition of multivariable candidate predictors**

The criteria we used for defining MCPs showed excellent sensitivity and specificity. Below is reported the expected output for n.subjects = 50 and n.models = 50:

#    variable sensitivity specificity
# 1         1           1          NA
# 2         2           1          NA
# 3         3           1          NA
# 4         4           1          NA
# 5         5          NA       0.838
# 6         6          NA       0.962
# 7         7          NA       0.965
# 8         8          NA       0.973
# 9         9          NA       0.977
# 10       10        NA       0.974

Note that numbers may differ slightly after each run, but sensitivity for the first variables should be close to 100%, and specificity for the last six variables should be >95% - except for variable 5 (specificity around 85

%.)

**eDISCUSSION**

In this umbrella review, we summarized the evidence from 117 systematic reviews, including 403 individual studies and 299,888 persons with mental disorders, testing multivariable models to predict treatment response or illness remission, recovery, or relapse. We showed that the field has relevant methodological limitations, with only 4.3% of systematic reviews having high quality and only 1.2% of models in individual studies having low risk of bias. The most frequently studied outcomes were remission (37%) and relapse (35%), with more limited evidence for response (17%) and with especially little evidence for clinically highly relevant recovery (3%) that combines symptomatic and functional attainment.

Facing methodological complexities of the published non-modifiable (risk factors) and modifiable (actionable factors) and non-modifiable multivariable predictors of clinically relevant outcomes across mental disorders(7,11), we filtered the most promising MCPs that should be used as candidates to refine multivariable models in further studies.

Female sex was a MCP of better clinical outcome across mood disorders, schizophrenia-spectrum disorders, and substance use disorders, and a trans-outcome MCP in depressive disorders (response, remission). Better neuroplasticity and less pro-inflammatory status(12), higher resilience(13), and better social cognition(14) may mediate this association.

Older age predicting better outcomes across diagnostic boundaries calls for early intervention at younger age when mental disorders frequently have onset(15), to minimize psychosocial impact and avoid a poor prognostic cascade effect with additional multiple negative prognostic factors. Moreover, younger age at onset of substance use disorders predicted greater relapse risk, possibly via a negative impact of substance use in early age on functioning, mental and physical health(16), including decreased gray matter, ultimately decreasing chances of response to available treatments, and affecting education(17). Indeed, education, a transdiagnostic modifiable MCP of good outcomes, can be jeopardized by mental disorders, which are frequently present among high school, and university(18) studentsAdditional non-modifiable patient- and illness-related MCPs of good outcomes within a mental disorder were 5-HT transporter polymorphisms and absent family history of mental disorders in adults with depressive disorders, male sex in older adults with depressive disorder, as well as younger age at illness onset and gray matter volume in substance use disorders.

The serotonin hypothesis of depression has been initially proposed in 1967 with the development of the first antidepressants(19). Since then, the theory has been expanded and updated, involving more complex mechanisms and additional neurotransmitters(20,21). Recently, an umbrella review with concerning methodological limitations claimed that there is no basis of any involvement of serotonin in depression, essentially confirming the opinion the authors had already stated in lay press outlets and books, ultimately recommending against the use of antidepressants for depression(22). The finding of our umbrella review of 5HT polimorpshism being a MCP of improved treatment response in individuals with depression supports the role of serotonin in depression and treatment response, which is already well supported by well conducted network meta-analyses clearly showing that antidepressants, including selective serotonin reuptake inhibitors, are effective in depression(23).

Regarding family history of mental disorders, this umbrella work shows that family history of mental disorders not only increases the risk of mental disorders, but it also increases the chances of relapse of depression(24,25), calling for close monitoring and potentially selective relapse prevention strategies or closer screening to ensure early targeted interventions in those with a history of mental disorders.

Despite depression being more frequent in women than men(26), female sex was a consistent positive prognostic factor across several mental disorders. However, male sex selectively predicted better outcome in elderlies with depression, possibly due to menopausal hormonal changes in females(27), and less spousal support for females(28).

Younger age at illness onset of substance use disorders predicted greater relapse risk. Substance use during school age can have dramatic consequences on education(17), functioning, mental and physical health(16,29), increasing the risk of additional poor prognostic factors(30,31). Ultimately, poor physical health and long-term substance use can result in reduced gray matter, which in turn further decreases the chances of good outcomes in individuals suffering from substance use disorders. Decreased gray matter might actually be a proxy of toxicity on the central nervous system of polysubstance use(32), in particular with use of substances at a younger age(33), which decreases chances of response to available treatments.

Education emerged as a transdiagnostic modifiable MCP of good outcomes, which should be promoted and to which universal access should ideally be warranted. Intervening early in people with mental disorders likely implies improving education attainments. For instance, the prevalence of depression or depressive symptoms ranges from 23% to 44% in high school students(34–36), and is around 27% in medical students(18). Anxiety disorders can range from 24% to 66.7% in highschool(34), and be as high as around 34%(37) in medical students. Hence, mental disorders start affecting functioning during school age, with possible impact on education achievements and completion(38,39), which, in turn, reduces chances of desired outcomes.

Importantly, modifiable transdiagnostic MCPs of outcomes across five mental disorders consisted of disease-specific symptoms, i.e., with more depressive and anxiety symptoms predicting more relapse in depressive and anxiety disorder, and with less depressive, positive, and negative symptom severity predicting more remission in depressive and schizophrenia-spectrum disorders, respectively. Moreover, lower depressive symptoms (bipolar, depressive and substance use disorders) and higher education (depressive, schizophrenia-spectrum and substance use disorders) predicted better outcomes in three disorders, each, while fewer previous illness episodes (depressive and schizophrenia-spectrum disorders), higher quality of life or functioning (depressive and schizophrenia-spectrum disorders), lower global illness severity (schizophrenia-spectrum and substance use disorders) or anxiety symptoms (anxiety and depressive disorders), and no history of negative life events (anxiety and depressive disorders) were transdiagnostic MCPs across two mental disorders.

Among MCPs, illness symptom severity was a replicated transdiagnostic and trans-outcome characteristic as we identified illness symptom levels as a crucial, evidence-based target for enhancing the chance of remission and reducing the risk of relapse. Among the identified MCPs, illness symptom severity,together with female sex, absent negative life events, higher education and better functioning fulfilled both transdiagnostic or trans-outcome characteristics. In particular, fewer symptoms of anxiety and depression predicted greater remission in each disorder, and more symptoms of depression and positive as well as negative symptoms of schizophrenia predicted more relapse of depression and of schizophrenia, respectively. This finding relates to the potential of early detection and treatment before symptoms reach their peak and become enduring, as well as for the implementation of effective and adequately dosed psychosocial and pharmacologic treatments, which could minimize (residual) symptoms and reduce the likelihood of the emergence of other related candidate predictors, i.e., more illness episodes and unemployment in depression, and lower quality of life in schizophrenia (where a better quality of life was related to a greater likelihood of remission). Data on the undesirable effect of residual symptoms and unstable disease on increased relapse risk have been reported in depression(40–42) and schizophrenia(43). Relatedly, insufficiently low antidepressant or antipsychotic doses have also been identified as a risk factor for less and later response and remission in depression(44) as well as for relapse in schizophrenia(45–47). Hence, targeting symptom levels and treating them as fully as possible appears to be a critical factor for achieving symptomatic remission and relapse prevention. Further, symptom stability and avoiding the biopsychosocial disruption caused by relapse also enhances the ability to benefit from psychosocial and rehabilitative interventions. Importantly, to be able to target symptoms appropriately, they should to be measured. Measurement-based care has the promise to improve outcomes(44,48). However, it is not feasible to administer time-consuming scales and interviews to measure symptoms in the real world, such as the 30-item Positive and Negative Syndrome Scale or a 21-item Hamilton Depressive Rating scale(49). Hence, shortened but validated versions of longer scales, such as PANSS-6 for schizophrenia(50,51), or self-report measures of symptoms, such as the Patient Health Questionnaire 9 items, or Generalized Anxiety Disorder 7 items for depressive and anxiety disorders, should be explored for both clinical and research purposes (52,53). Moreover, self-report questionnaires can be easily implemented in electronic medical software, with the additional benefit of engaging and educating patients to self-monitor symptoms. The shift to virtual care in the field of psychiatry that occurred during the COVID-19 pandemic might have made the integration of measurements of symptoms into clinical practice easier(54).

Among MCPs, illness symptom severity was a replicated transdiagnostic and trans-outcome characteristic. Fewer symptoms of anxiety and depression predicted greater remission in each disorder, and more symptoms of depression predicted depression relapse, while more positive and negative symptoms predicted schizophrenia relapse . This finding relates to the potential of early detection and treatment before symptoms reach their peak and become enduring, as well as for the implementation of measurement-based effective and adequately dosed psychosocial and pharmacologic treatments, which could minimize (residual) symptoms and reduce the likelihood of the emergence of other related candidate predictors, i.e., more illness episodes and unemployment in depression, and lower quality of life in schizophrenia (where a better quality of life was related to a greater likelihood of remission). Shortened but validated versions of longer scales, such as PANSS-6 for schizophrenia(55), or self-report measures of symptoms, such as the Patient Health Questionnaire 9 items for depressive(52) or Generalized Anxiety Disorder 7 items for anxiety disorders(53), can facilitate measurement-based care. Moreover, self-report questionnaires can be easily implemented in electronic medical software, with the additional benefit of engaging and educating patients to self-monitor symptoms. The shift to virtual care in psychiatry that occurred during the COVID-19 pandemic might have made the integration of symptom measurements into clinical practice easier(54).

Depressive symptoms were modifiable, illness-related MCPs of relapse in bipolar disorder, confirming previous evidence on their predominant proportion of non-euthymic mood states(56), and association with duration of untreated illness(56) and inter-episodic impairment in functioning(57), possibly related with misdiagnosis leading to use of antidepressants(58).

Less anxiety symptoms and/or comorbiditiy were also MCPs of remission and less relapse in anxiety and depressive disorders. Anxiety symptoms are frequently present in depressive disorder and can be markers of bipolar spectrum symptoms. Therefore, careful screening of previous manic or hypomanic episodes in people with depressive disorder with anxiety symptoms should be conducted(58–60). Also, adherence to antidepressants is frequently non-optimal, and simplifying the treatment regimen, including minimizing the number of different medications can favour compliance(48). Pharmacological and non-pharmacological interventions that target both depressive and anxiety symptoms or disorders in subjects affected by both disorders should be offered(23,61–64).

A greater number or prior illness episodes was another modifiable illness-related MCP for both depressive and schizophrenia-spectrum disorders. Effective relapse prevention should always be targeted, as the more episodes a patient has, the higher the risk of relapse is. In order to prevent relapse, in schizophrenia-spectrum disorders long-acting injectable antipsychotics should be offered(65–69), since the early stages of illness(70–72), ideally at a dose between 0.6 and 1 Daily Defined Doses (DDDs) (e.g. for risperidone 1 DDD is 5mg)(45,73,74), at the best trade-off between efficacy, tolerability, and safety(64,75–78), to be augmented with psychososcial interventions(79). In depression, a combination of psychotherapy and antidepressants might allow safer discontinuation of antidepressants(80–86), should a patient decide to discontinue pharmacological treatment, and exercise should always be proposed given its transdiagnostic beneficial effects on the body and the mind(87–89).

As we and others have shown in several other meta-research projects focusing on risk factors for mental disorders(90–94), one key transdiagnostic MCP in this umbrella review was negative life events, including childhood abuse(30). According to findings from this and previous umbrella reviews and meta-umbrella reviews that we and other groups conducted on risk factors of all mental disorders(30,95–97), negative life events, including childhood abuse, not only is the most transdiagnostic risk factor increasing the risk of multiple mental disorders, but also impacting their long-term outcomes, resulting in major health inequity and individual as well as societal costs. Thus, prevention of negative life events and, especially, of abuse and neglect during childhood, plus increasing resilience factors are key, both globally but especially in populations with an increased risk for mental disorders, as not only the emergence of mental illness but also worse outcomes within mental disorders are more likely among those with early negative life experiences(98,99).

Moreover, the fact that quality of life was a MCP of remission in schizophrenia highlights that subjective wellbeing and patient-reported outcomes and goals beyond symptoms, relapse, including functional level, need to be considered and targeted to improve outcomes(100–102). Patient-reported outcomes are increasingly measured across different branches of medicine, and promoting patient engagement and measuremenet-based care with the use of digital tools can be a scalable approach with relatively little costs(103).

In addition to transdiagnostic modifiable MCPs, other MCPs were identified within specific mental disorders, some of them also being replicated across multiple outcomes. In adults with depression, trans-outcome modifiable MCPs of positive outcomes included better functioning. Additional MCPs of increased chances of response or remission, or lower risk of relapse in depressive disorders were being employed, having less psychiatric and physical comorbidities, lower episode duration, better emotional regulation/coping strategies, more social contacts, and early treatment response. People with mental disorders have indeed poor physical health and also receive lower quality of screening and care for physical conditions(104,105). There is a bidirectional relationship between mental and physical health, with the former influencing outcomes of the latter, and vice versa(106–108). Quality care of those with mental disorders should also target physical health(88,109). Poor emotional regulation might indicate a possible comorbidity with borderline personality traits(110,111), which might complicate the response to standard pharmacological treatment or psychotherapies for depression(112). Preventing social isolation and loneliness, which are a common and increasing problem in the elderlies(113), could improve outcomes since social contacts emerged as a MCP of remission in depressive disorders(113–115). Early treatment response should be targeted. Early response could be enhanced by offering biological and psychosocial treatments first-line to all patients with depression, including exercise with its pleiotropic beneficial effect, and by offering early switch to second-line antidepressants when first-line treatment is not effective(80–89).

While lower depressive symptoms were a transdiagnostic predictor of good outcomes, conversey, lower depressive symptoms seemed to predict relapse in schizophrenia. From a phenomenological perspective, higher depressive symptoms might indicate lower severity of flat affect and negative symptoms(116), with more negative symptoms having been associated with poorere outcomes(117–119). Nevertheless, depression is frequently comorbid in those with schizophrenia(120), and, if left untreated, depressive symptoms could also worsen prognosis, including suicide mortality. Hence, medications, other biological treatments, and psychosocial treatments that are not only effective and safe for disease-specific symptoms, i.e, positive and negative symptoms, but that can also improve mood should be offered (69,79,121–124), and/or treatment of comorbid depressive symptoms or disorder with antidepressants should be offered to persons with schizophrenia(123,124).

In addition to the importance of treating depressive symptoms or disorders, self-injurious behaviors should be prevented and self-efficacy should be promoted(125–127) to improve outcomes in substance use disorders. Self-injurious behavior might be a marker of depressive symptoms, or borderline personality traits or disorders, which might complicate the response to standard treatments for substance use disorder. In case that comorbid borderline personality disorder underlies impaired self-efficacy that can increase relapse in substance use disorder, psychological treatment should be offered to optimize outcomes in people with substance use disorder(128).

Finally, in eating disorders, in addition to body weight and eating disorder-specific symptoms, illness duration seems to predict poor outcome. It is important that effective treatments for eating disorders are provided as early as possible(129), and that services account for the early age at onset of eating disorders, which occurs in almost 50% of patients before age 18(130).

Unfortunately, there was a paucity of data in the area of predictors for recovery. Still, low symptom levels and symptomatic stability should be explored as one relevant predictor for achieving recovery, as has been demonstrated in patients with first-episode schizophrenia previously(131).

Although the prediction of outcomes is a critical goal for all mental disorders, the vast majority of studies have focused on depressive disorders, substance use disorders, and schizophrenia. Moreover, although proximal treatment response and more distal achievement of remission are also crucial to improving outcomes, multivariable predictor studies were mainly dominated by a focus on symptomatic remission and relapse, with the least attention drawn to the most important outcome of recovery. The fact that only 21% of eligible individual studies were RCTs and that only 4.7% of studies focused specifically on predictive factors is surprising since a large meta-analysis of many RCTs exist across many of the included mental disorders, which include many well-characterized individuals with prospectively determined and clearly defined outcomes. This finding indicates a large but so far lost opportunity when only conducting study-level outcomes and, much less so, prediction analyses, opening the door for pooled individual patient level and pooling data from comparable RCTs and testing multivariable models of treatment response. Moreover, the fact that <10% of the studies each focused on the elderly, a largely growing population, and on children and adolescents whose outcomes determine long-term illness trajectories throughout life and for whom efficacy and safety information on interventions exist(64,77), calls for more multivariable prediction research in these critical age groups. Taken together, these results indicate that more high-quality studies are needed that focus on a broader range of mental disorders and across age groups, also focusing on predictors of treatment response and recovery, that use multivariable modeling to reduce the risk of spurious findings that is likely when focusing only on univariate and/or cross-sectional correlations.

Results from this umbrella review provide several specific leads and recommendations for additional research on predicting outcomes across mental disorders. First, future studies should use a more homogeneous definition of predictors to facilitate evidence synthesis projects that can further inform future multivariable models on the prediction of outcomes. The preponderance of studies in individuals with depression may, at least in part, be driven by the fact that in depression, definitions of response and remission and the related scales used for these definitions had been proposed early and accepted and adopted widely. Second, whenever sample size allows, studies should focus on more outcomes that could be predicted, primarily, as response, remission, and recovery are related to one another clinically. Third, transdiagnostic predictors could also optimize scalability, as it is more feasible to collect the same set of predictors that can be clinically meaningful across multiple disorders, as opposed to collecting different predictors based on the primary diagnosis. Moreover, mental disorders are frequently comorbid with other mental disorders, and the prognosis of each disorder can contribute to the overall well-being and quality of life of persons with mental disorders. Fourth, identifying trans-outcome predictors might also increase efficiency in research and clinical care, provided that these predictors could be assessed at scale.

This study also has several limitations. First, when presenting results, we reduced the granularity of predictors for feasibility and knowledge translation considerations. However, this umbrella review should serve as a starting point to inform future studies testing literature-informed candidate predictors rather than being used as a clinical guide. Unfortunately, the high risk of bias in virtually all eligible studies precludes clinical implementations of any reported predictors at this stage of research knowledge. Second, since our search key focused on systematic reviews, as this is an umbrella review, we may have missed individual studies that could have been published since the most recent systematic review for each combination of populations, interventions, controls, and outcomes. However, this is the first umbrella review on MCPs of response, remission, recovery, and relapse, which can also identify where the first or an updated systematic review is needed. Third, methodological decisions on how to label predictors and outcomes were made after data extraction, given the large and heterogeneous body of included evidence, as detailed in the methods section, to translate data into information. Fourth, it was not always sufficiently clear whether the MCPs studied in at least two studies were significantly associated with the outcome of interest, and if they were, in which direction, as reporting was often poor, and mainly focused on model performance rather than on individual factors. Fifth, the criteria we applied to identify MCPs were arbitrary but validated via simulations. Sixth, many mental disorders had only limited published MCP evidence available, calling for more research and appropriate funding to conduct such research in sufficiently large samples with enough patients per tested variable in the multivariable analyses. Seventh, information regarding candidate prognostic and predictive factors that significantly affect treatment response and, especially, recovery were mainly missing, calling for more research attention to this area. Eighth, many predictors were only tested in a few studies with small samples, and some models might have suffered from overfitting. Ninth, the effect of treatments, comorbid disorders, and usual illness trajectory on the predictors and outcomes could not be measured. Future studies should attempt to delineate those factors more clearly. Tenth, the difference between “interventions” and “predictive factors” is sometimes debatable, i.e., some researchers may consider that antipsychotic dose reduction is a potential predictive factor. In contrast, others may consider that dose reduction is an intervention. Finally, we focused on individual predictors extracted from multivariable models. Still, their performance depended on other model variables, and future research may identify several significant predictors performing even better when combined. Therefore, validation studies testing these candidate predictors within and across different populations and models will be important.

In conclusion, despite limitations of this work and the available literature, this umbrella review for the first time scrutinized the level of evidence for MCPs of response, remission, recovery, and relapse across mental disorders and outcomes, identifying MCPs across mental disorders and outcomes, and calling out numerous areas that need further investigation. Future studies should replicate broadly defined MCPs of major clinical outcomes identified by this study, and consider them to refine existing or build even superior multivariable models.

**Reporting checklist. Preferred Reporting Items for Overviews of Reviews (PRIOR)**(1)

| **Section**  Topic | **#** | **Item** | **Location reported** |
| --- | --- | --- | --- |
| **TITLE** | | |  |
| Title | 1 | Identify the report as an overview of reviews. | 1 |
| **ABSTRACT** | | |  |
| Abstract | 2 | Provide a comprehensive and accurate summary of the purpose, methods, and results of the overview of reviews. | 2 |
| **INTRODUCTION** | | |  |
| Rationale | 3 | Describe the rationale for conducting the overview of reviews in the context of existing knowledge. | 3 |
| Objectives | 4 | Provide an explicit statement of the objective(s) or question(s) addressed by the overview of reviews. | 3 |
| **METHODS** | | |  |
| Eligibility criteria | 5a | Specify the inclusion and exclusion criteria for the overview of reviews. If supplemental primary studies were included, this should be stated, with a rationale. | 4/E-methods |
|  | 5b | Specify the definition of ‘systematic review’ as used in the inclusion criteria for the overview of reviews. | 4/E-methods |
| Information sources | 6 | Specify all databases, registers, websites, organizations, reference lists, and other sources searched or consulted to identify systematic reviews and supplemental primary studies (if included).  Specify the date when each source was last searched or consulted. | 4/E-methods |
| Search strategy | 7 | Present the full search strategies for all databases, registers and websites, such that they could be reproduced. Describe any search filters and limits applied. | E-methods |
| Selection process | 8a | Describe the methods used to decide whether a systematic review or supplemental primary study (if included) met the inclusion criteria of the overview of reviews. | 4/E-methods |
|  | 8b | Describe how overlap in the populations, interventions, comparators, and/or outcomes of systematic reviews was identified and managed during study selection. | E-methods |
| Data collection process | 9a | Describe the methods used to collect data from reports. | 5/E-methods |
|  | 9b | If applicable, describe the methods used to identify and manage primary study overlap at the level  of the comparison and outcome during data collection. For each outcome, specify the method used to illustrate and/or quantify the degree of primary study overlap across systematic reviews. | NA |
|  | 9c | If applicable, specify the methods used to manage discrepant data across systematic reviews during data collection. | NA |
| Data items | 10 | List and define all variables and outcomes for which data were sought. Describe any assumptions made and/or measures taken to identify and clarify missing or unclear information. | 5-6/E-methods |
| Risk of bias assessment | 11a | Describe the methods used to *assess* risk of bias or methodological quality of the included systematic reviews. | 7/E-methods |
|  | 11b | Describe the methods used to *collect* data on (from the systematic reviews) and/or *assess* the risk of bias of the primary studies included in the systematic reviews. Provide a justification for instances where flawed, incomplete, or missing assessments are identified but not re-assessed. | 7/E-methods |
|  | 11c | Describe the methods used to *assess* the risk of bias of supplemental primary studies (if included). | 7/E-methods |
| Synthesis methods | 12a | Describe the methods used to summarize or synthesize results and provide a rationale for the choice(s). | 6-7/E-methods |
|  | 12b | Describe any methods used to explore possible causes of heterogeneity among results. | 6-7/E-methods |
|  | 12c | Describe any sensitivity analyses conducted to assess the robustness of the synthesized results. | NA |
| Reporting bias assessment | 13 | Describe the methods used to *collect* data on (from the systematic reviews) and/or *assess* the risk of bias due to missing results in a summary or synthesis (arising from reporting biases at the levels of the systematic reviews, primary studies, and supplemental primary studies, if included). | E-methods |
| Certainty assessment | 14 | Describe the methods used to *collect* data on (from the systematic reviews) and/or *assess* certainty (or confidence) in the body of evidence for an outcome. | E-methods |
| **RESULTS** | | |  |
| Systematic review and supplemental primary study selection | 15a | Describe the results of the search and selection process, including the number of records screened, assessed for eligibility, and included in the overview of reviews, ideally with a flow diagram. | 7-27 |
|  | 15b | Provide a list of studies that might appear to meet the inclusion criteria, but were excluded, with the main reason for exclusion. | E-table 1 |
| Characteristics of systematic reviews and  supplemental primary studies | 16 | Cite each included systematic review and supplemental primary study (if included) and present its characteristics. | E-table 2/E-table 3 |
| Primary study overlap | 17 | Describe the extent of primary study overlap across the included systematic reviews. | NA |
| Risk of bias in systematic reviews, primary studies, and  supplemental primary studies | 18a | Present assessments of risk of bias or methodological quality for each included systematic review. | 8/E-results |
|  | 18b | Present assessments (collected from systematic reviews or assessed anew) of the risk of bias of  the primary studies included in the systematic reviews. | 8/E-results |
|  | 18c | Present assessments of the risk of bias of supplemental primary studies (if included). | 8/E-results |
| Summary or synthesis of results | 19a | For all outcomes, summarize the evidence from the systematic reviews and supplemental primary studies (if included). If meta-analyses were done, present for each the summary estimate and its  precision and measures of statistical heterogeneity. If comparing groups, describe the direction of the effect. | E-results |
|  | 19b | If meta-analyses were done, present results of all investigations of possible causes of  heterogeneity. | NA |
|  | 19c | If meta-analyses were done, present results of all sensitivity analyses conducted to assess the  robustness of synthesized results. | NA |
| Reporting biases | 20 | Present assessments (collected from systematic reviews and/or assessed a new) of the risk of bias due to missing primary studies, analyses, or results in a summary or synthesis (arising from reporting biases at the levels of the systematic reviews, primary studies, and supplemental primary  studies, if included) for each summary or synthesis assessed. | NA |
| Certainty of  evidence | 21 | Present assessments (collected or assessed anew) of certainty (or confidence) in the body of  evidence for each outcome. | E-results |
| **DISCUSSION** | | |  |
| Discussion | 22a | Summarize the main findings, including any discrepancies in findings across the included systematic reviews and supplemental primary studies (if included). | 11-16 |
|  | 22b | Provide a general interpretation of the results in the context of other evidence. | 11-16 |
|  | 22c | Discuss any limitations of the evidence from systematic reviews, their primary studies, and supplemental primary studies (if included) included in the overview of reviews. Discuss any  limitations of the overview of reviews methods used. | 11-16 |
|  | 22d | Discuss implications for practice, policy, and future research (both systematic reviews and  primary research). Consider the relevance of the findings to the end users of the overview of reviews, e.g., healthcare providers, policymakers, patients, among others. | 11-16 |
| **OTHER INFORMATION** | | |  |
| Registration and protocol | 23a | Provide registration information for the overview of reviews, including register name and registration number, or state that the overview of reviews was not registered. | 5 |
|  | 23b | Indicate where the overview of reviews protocol can be accessed, or state that a protocol was not  prepared. | 5 |
|  | 23c | Describe and explain any amendments to information provided at registration or in the protocol.  Indicate the stage of the overview of reviews at which amendments were made. | Sup. Mat. |
| Support | 24 | Describe sources of financial or non-financial support for the overview of reviews, and the role of  the funders or sponsors in the overview of reviews. | 17 |
| Competing  interests | 25 | Declare any competing interests of the overview of reviews’ authors. | 17 |
| Author information | 26a | Provide contact information for the corresponding author. | 1 |
|  | 26b | Describe the contributions of individual authors and identify the guarantor of the overview of  reviews. | 16-17/E-methods |
| Availability of data and other materials | 27 | Report which of the following are available, where they can be found, and under which conditions they may be accessed: template data collection forms; data collected from included systematic  reviews and supplemental primary studies; analytic code; any other materials used in the overview of reviews. | E-methods |

**
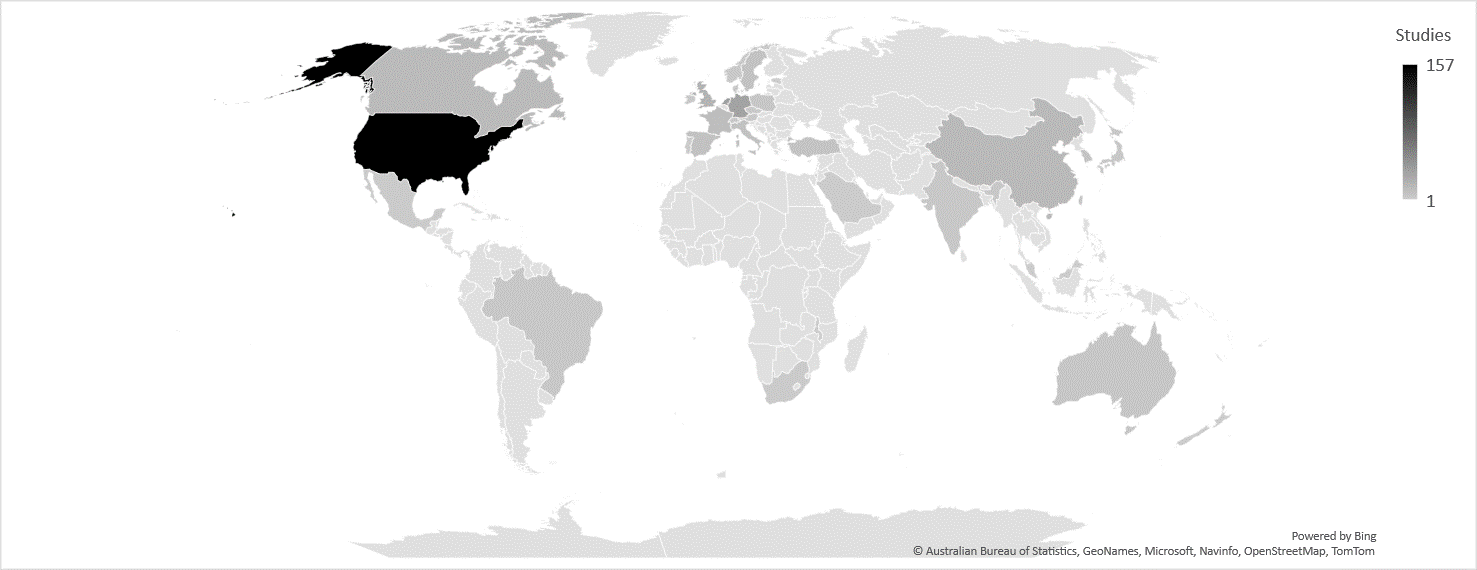
eFigure 1. Geographic distribution of individual studies reporting on published predictors of response, remission, recovery and relapse across mental disorders.**

**eFigure 2. Flow of filtering from all broadly defined, and multivariable candidate predictors by the diagnostic group across mental disorders**

**Trauma and stress-related disorders**

N=128

N=0

N=0

N=6

N=0

N=0

N=13

N=0

N=3

N=23

N=0

N=1

N=28

N=0

N=6

N=71

N=3

N=18

N=84

N=3

N=29

N=113

N=3

N=25

N=133

N=15

N=62

N=136

N=12

N=58

N=289

N=36

N=137

**Personality disorders**

**Neurodevelopmental disorder**

**Obsessive-compulsive disorders**

**Feeding and eating disorders**

**Bipolar disorders**

**Anxiety disorders**

**Mixed disorders**

**Substance use disorder**

**Schizophrenia spectrum disorders**

**Depressive disorders**

**All published**

**predictors**

**N=1,024**

**Broadly defined**

**predictors**

**N=339**

**Multivariable candidate predictors**

**N=72**

*Legend. The number of predictors examined in multivariable models is larger than the sum of the predictors across the mental disorder, as the same predictor could have been used within each disorder in up to three different age groups and for up to four different outcomes*

*.*

**eTable 1. List of systematic reviews excluded after full-text assessment**

| **Reference** | Reason |
| --- | --- |
| Abate, S. M., Checkole, Y. A., Mantedafro, B., Basu, B., & Aynalem, A. E. (2021). Global prevalence and predictors of postoperative delirium among non-cardiac surgical patients: A systematic review and meta-analysis. International Journal of Surgery Open, 32, 100334. https://doi.org/https://dx.doi.org/10.1016/j.ijso.2021.100334 | Not reporting outcomes of interest |
| Ahern, E., & Semkovska, M. (2017). Cognitive functioning in the first episode of major depressive disorder: A systematic review and meta-analysis. Neuropsychology, 31(1), 52-72. https://doi.org/https://dx.doi.org/10.1037/neu0000319 | Not reporting outcomes of interest |
| Ahmed, R., Kotapati, V. P., Khan, A. M., Hussain, N., Hussain, M., Dar, S., Kumar, J., Begum, G. A., Esang, M., Brainch, N., & Ahmed, S. (2018). Adding Psychotherapy to the Naltrexone Treatment of Alcohol Use Disorder: Meta-analytic Review. Cureus, 10(8), e3107. https://doi.org/https://dx.doi.org/10.7759/cureus.3107 | Univariable models or correlational studies |
| Aldi, G. A., Bertoli, G., Ferraro, F., Pezzuto, A., & Cosci, F. (2018). Effectiveness of pharmacological or psychological interventions for smoking cessation in smokers with major depression or depressive symptoms: A systematic literature review. Substance abuse, 39(3), 289-306. https://doi.org/https://dx.doi.org/10.1080/08897077.2018.1439802 | Not reporting outcomes of interest |
| Aleman, A., Enriquez-Geppert, S., Knegtering, H., & Dlabac-de Lange, J. J. (2018). Moderate effects of noninvasive brain stimulation of the frontal cortex for improving negative symptoms in schizophrenia: Meta-analysis of controlled trials. Neuroscience and biobehavioral reviews, 89, 111-118. https://doi.org/https://dx.doi.org/10.1016/j.neubiorev.2018.02.009 | Not reporting outcomes of interest |
| Allan, C. L., Herrmann, L. L., & Ebmeier, K. P. (2011). Transcranial magnetic stimulation in the management of mood disorders. Neuropsychobiology, 64(3), 163-169. https://doi.org/https://dx.doi.org/10.1159/000328951 | Univariable models or correlational studies |
| Alonso, P., Cuadras, D., Gabriels, L., Denys, D., Goodman, W., Greenberg, B. D., Jimenez-Ponce, F., Kuhn, J., Lenartz, D., Mallet, L., Nuttin, B., Real, E., Segalas, C., Schuurman, R., du Montcel, S. T., & Menchon, J. M. (2015). Deep Brain Stimulation for Obsessive-Compulsive Disorder: A Meta-Analysis of Treatment Outcome and Predictors of Response. PLoS ONE, 10(7), e0133591. https://doi.org/https://dx.doi.org/10.1371/journal.pone.0133591 | Univariable models or correlational studies |
| Altar, C. A., Hornberger, J., Shewade, A., Cruz, V., Garrison, J., & Mrazek, D. (2013). Clinical validity of cytochrome P450 metabolism and serotonin gene variants in psychiatric pharmacotherapy. International review of psychiatry (Abingdon, England), 25(5), 509-533. https://doi.org/https://dx.doi.org/10.3109/09540261.2013.825579 | Only searched one database |
| Alvarez-Jimenez, M., O'Donoghue, B., Thompson, A., Gleeson, J. F., Bendall, S., Gonzalez-Blanch, C., Killackey, E., Wunderink, L., & McGorry, P. D. (2016). Beyond Clinical Remission in First Episode Psychosis: Thoughts on Antipsychotic Maintenance vs. Guided Discontinuation in the Functional Recovery Era. CNS Drugs, 30(5), 357-368. https://doi.org/https://dx.doi.org/10.1007/s40263-016-0331-x | Only searched one database |
| Alzeer, A. H., Jones, J., & Bair, M. J. (2018). Review of factors, methods, and outcome definition in designing opioid abuse predictive models. Pain Medicine (United States), 19(5), 997-1009. https://doi.org/https://dx.doi.org/10.1093/pm/pnx149 | Only searched one database |
| Anagnostou, E. (2018). Clinical trials in autism spectrum disorder: Evidence, challenges, and future directions. Current Opinion in Neurology, 31(2), 119-125. https://doi.org/https://dx.doi.org/10.1097/WCO.0000000000000542 | Study design not eligible |
| Andersson, G., Carlbring, P., & Rozental, A. (2019). Response and Remission Rates in Internet-Based Cognitive Behavior Therapy: An Individual Patient Data Meta-Analysis. Frontiers in Psychiatry, 10, 749. https://doi.org/https://dx.doi.org/10.3389/fpsyt.2019.00749 | Univariable models or correlational studies |
| Andrade, C. (2016). Cannabis and neuropsychiatry, 2: The longitudinal risk of psychosis as an adverse outcome. Journal of Clinical Psychiatry, 77(6), e739-e742. https://doi.org/https://dx.doi.org/10.4088/JCP.16f10918 | Study design not eligible |
| Andraud, F., & Hardy, P. (2005). Prognostic factors of recovery. Encephale, 31(6 III), S18-S20. http://ovidsp.ovid.com/ovidweb.cgi?T=JS&PAGE=reference&D=emed9&NEWS=N&AN=43568999 | Study design not eligible |
| Angst, J. (1993). The severity of depression and benzodiazepine co-medication in relationship to efficacy of antidepressants in acute trials. A meta-analysis of moclobemide trials. Human Psychopharmacology, 8(6), 401-407. http://ovidsp.ovid.com/ovidweb.cgi?T=JS&PAGE=reference&D=emed5&NEWS=N&AN=24086958 | Study design not eligible |
| Abramovitch A., & Cooperman A. (2015). The cognitive neuropsychology of obsessive-compulsive disorder: A critical review. Journal of Obsessive-Compulsive and Related Disorders, 5, 24-36. https://doi.org/https://dx.doi.org/10.1016/j.jocrd.2015.01.002 | Study design not eligible |
| Antunes, P. B., Rosa, M. A., Belmonte-de-Abreu, P. S., Lobato, M. I. R., & Fleck, M. P. (2009). [Electroconvulsive therapy in major depression: current aspects]. Eletroconvulsoterapia na depressao maior: aspectos atuais., 31 Suppl 1, S26-33. http://ovidsp.ovid.com/ovidweb.cgi?T=JS&PAGE=reference&D=med7&NEWS=N&AN=19565148 | Study design not eligible |
| Ayerbe, L., Ayis, S., Wolfe, C. D. A., & Rudd, A. G. (2013). Natural history, predictors and outcomes of depression after stroke: systematic review and meta-analysis. The British journal of psychiatry : the journal of mental science, 202(1), 14-21. <https://doi.org/https://dx.doi.org/10.1192/bjp.bp.111.107664> | Patient population not eligible |
| Bacaltchuk, J., Trefiglio, R. P., de Oliveira, I. R., Lima, M. S., & Mari, J. J. (1999). Antidepressants versus psychotherapy for bulimia nervosa: a systematic review. Journal of clinical pharmacy and therapeutics, 24(1), 23-31. http://ovidsp.ovid.com/ovidweb.cgi?T=JS&PAGE=reference&D=med4&NEWS=N&AN=10319904 | Not investigating prognostic or predictive factors |
| Backhouse, E. V., McHutchison, C. A., Cvoro, V., Shenkin, S. D., & Wardlaw, J. M. (2018). Cognitive ability, education and socioeconomic status in childhood and risk of post-stroke depression in later life: A systematic review and meta-analysis. PLoS ONE, 13(7), e0200525. https://doi.org/https://dx.doi.org/10.1371/journal.pone.0200525 | Not reporting outcomes of interest |
| Baglioni, C., Battagliese, G., Feige, B., Spiegelhalder, K., Nissen, C., Voderholzer, U., Lombardo, C., & Riemann, D. (2011). Insomnia as a predictor of depression: a meta-analytic evaluation of longitudinal epidemiological studies. Journal of Affective Disorders, 135(1-3), 10-19. https://doi.org/https://dx.doi.org/10.1016/j.jad.2011.01.011 | Not reporting outcomes of interest |
| Bahadoor, R., Alexandre, J.-M., Fournet, L., Gelle, T., Serre, F., & Auriacombe, M. (2021). Inventory and Analysis of Controlled Trials of Mobile Phone Applications Targeting Substance Use Disorders: A Systematic Review. Frontiers in Psychiatry, 12, 622394. https://doi.org/https://dx.doi.org/10.3389/fpsyt.2021.622394 | Not reporting outcomes of interest |
| Bahji, A., Hawken, E. R., Sepehry, A. A., Cabrera, C. A., & Vazquez, G. (2019). ECT beyond unipolar major depression: systematic review and meta-analysis of electroconvulsive therapy in bipolar depression. *Acta psychiatrica Scandinavica*, *139*(3), 214–226. https://doi.org/10.1111/acps.12994 | No includible individual studies |
| Barlati, S., Deste, G., Galluzzo, A., Perin, A. P., Valsecchi, P., Turrina, C., & Vita, A. (2018). Factors Associated With Response and Resistance to Cognitive Remediation in Schizophrenia: A Critical Review. Frontiers in pharmacology, 9, 1542. https://doi.org/https://dx.doi.org/10.3389/fphar.2018.01542 | Not reporting outcomes of interest |
| Barnicot, K., Katsakou, C., Bhatti, N., Savill, M., Fearns, N., & Priebe, S. (2012). Factors predicting the outcome of psychotherapy for borderline personality disorder: a systematic review. Clinical psychology review, 32(5), 400–412. https://doi.org/10.1016/j.cpr.2012.04.004 | No includible individual studies |
| Bao, Y. P., Han, Y., Ma, J., Wang, R. J., Shi, L., Wang, T. Y., He, J., Yue, J. L., Shi, J., Tang, X. D., & Lu, L. (2017). Cooccurrence and bidirectional prediction of sleep disturbances and depression in older adults: Meta-analysis and systematic review. *Neuroscience and biobehavioral reviews*, *75*, 257–273. https://doi.org/10.1016/j.neubiorev.2017.01.032 | No includible individual studies |
| Barrio, C., Arias-Sanchez, S., & Martin-Monzon, I. (2022). The gut microbiota-brain axis, psychobiotics and its influence on brain and behavior: A systematic review. Psychoneuroendocrinology, 137, 105640. https://doi.org/https://dx.doi.org/10.1016/j.psyneuen.2021.105640 | Not reporting outcomes of interest |
| Barth, M., Kriston, L., Klostermann, S., Barbui, C., Cipriani, A., & Linde, K. (2016). Efficacy of selective serotonin reuptake inhibitors and adverse events: meta-regression and mediation analysis of placebo-controlled trials. The British journal of psychiatry: the journal of mental science, 208(2), 114-119. https://doi.org/https://dx.doi.org/10.1192/bjp.bp.114.150136 | Univariable models or correlational studies |
| Barton, S., Karner, C., Salih, F., Baldwin, D. S., & Edwards, S. J. (2014). Clinical effectiveness of interventions for treatment-resistant anxiety in older people: A systematic review. Health Technology Assessment, 18(50), 1-62. https://doi.org/https://dx.doi.org/10.3310/hta18500 | Not reporting outcomes of interest |
| Bauer, I. E., Soares, J. C., & Nielsen, D. A. (2015). The role of opioidergic genes in the treatment outcome of drug addiction pharmacotherapy: A systematic review. The American journal on addictions, 24(1), 15-23. https://doi.org/https://dx.doi.org/10.1111/ajad.12172 | Univariable models or correlational studies |
| Bauer, M., Adli, M., Bschor, T., Pilhatsch, M., Pfennig, A., Sasse, J., Schmid, R., & Lewitzka, U. (2010). Lithium's emerging role in the treatment of refractory major depressive episodes: augmentation of antidepressants. Neuropsychobiology, 62(1), 36-42. https://doi.org/https://dx.doi.org/10.1159/000314308 | Study design not eligible |
| Baumeister, H., Hutter, N., & Bengel, J. (2011). Psychological and pharmacological interventions for depression in patients with coronary artery disease. *The Cochrane database of systematic reviews*, *2011*(9), CD008012. https://doi.org/10.1002/14651858.CD008012.pub3 | No includible individual studies |
| Baumeister, H., Hutter, N., & Bengel, J. (2012). Psychological and pharmacological interventions for depression in patients with diabetes mellitus and depression. Cochrane database of systematic reviews (Online), 12, CD008381. http://ovidsp.ovid.com/ovidweb.cgi?T=JS&PAGE=reference&D=emed13&NEWS=N&AN=366385328 | Not investigating prognostic or predictive factors |
| Baumel, W. T., Lu, L., Huang, X., Drysdale, A. T., Sweeny, J. A., Gong, Q., Sylvester, C. M., & Strawn, J. R. (2022). Neurocircuitry of treatment in anxiety disorders. Biomarkers in Neuropsychiatry, 6, 100052. https://doi.org/https://dx.doi.org/10.1016/j.bionps.2022.100052 | Patient population not eligible |
| Bear, H. A., Edbrooke-Childs, J., Norton, S., Krause, K. R., & Wolpert, M. (2020). Systematic Review and Meta-analysis: Outcomes of Routine Specialist Mental Health Care for Young People With Depression and/or Anxiety. *Journal of the American Academy of Child and Adolescent Psychiatry*, *59*(7), 810–841. https://doi.org/10.1016/j.jaac.2019.12.002 | No includible individual studies |
| Beard, J. I. L., & Delgadillo, J. (2019). Early response to psychological therapy as a predictor of depression and anxiety treatment outcomes: A systematic review and meta-analysis. Depression and Anxiety, 36(9), 866-878. https://doi.org/https://dx.doi.org/10.1002/da.22931 | Not reporting outcomes of interest |
| Beaucage, C., Cardinal, L., Kavanagh, M., & Aube, D. (2009). [Major depression in primary care and clinical impacts of treatment strategies: a literature review]. La depression majeure en premiere ligne et les impacts cliniques des strategies d'intervention : une revue de la litterature., 34(1), 77-100. http://ovidsp.ovid.com/ovidweb.cgi?T=JS&PAGE=reference&D=med7&NEWS=N&AN=19475195 | No includible individual studies |
| Bell, L. (2002). Does concurrent psychopathology at presentation influence response to treatment for bulimia nervosa? Eating and weight disorders : EWD, 7(3), 168-181. http://ovidsp.ovid.com/ovidweb.cgi?T=JS&PAGE=reference&D=med4&NEWS=N&AN=12452248 | Study design not eligible |
| Bellani, M., Biagianti, B., Zovetti, N., Rossetti, M. G., Bressi, C., Perlini, C., & Brambilla, P. (2019). The effects of cognitive remediation on cognitive abilities and real-world functioning among people with bipolar disorder: A systematic review. Journal of Affective Disorders, 257, 691-697. https://doi.org/https://dx.doi.org/10.1016/j.jad.2019.07.059 | Not reporting outcomes of interest |
| Bentzley, B. S., Barth, K. S., Back, S. E., & Book, S. W. (2015). Discontinuation of buprenorphine maintenance therapy: perspectives and outcomes. Journal of substance abuse treatment, 52, 48-57. https://doi.org/https://dx.doi.org/10.1016/j.jsat.2014.12.011 | Study design not eligible |
| Berardelli, I., Serafini, G., Cortese, N., Fiasche, F., O'Connor R, C., & Pompili, M. (2020). The involvement of hypothalamus-pituitary-adrenal (Hpa) axis in suicide risk. Brain Sciences, 10(9), 1-12. https://doi.org/https://dx.doi.org/10.3390/brainsci10090653 | Not reporting outcomes of interest |
| Berlim, M. T., McGirr, A., Rodrigues Dos Santos, N., Tremblay, S., & Martins, R. (2017). Efficacy of theta burst stimulation (TBS) for major depression: An exploratory meta-analysis of randomized and sham-controlled trials. Journal of psychiatric research, 90, 102-109. https://doi.org/https://dx.doi.org/10.1016/j.jpsychires.2017.02.015 | Univariable models or correlational studies |
| Bernhardt, M., Klauke, S., & Schroder, A. (2019). Longitudinal course of cognitive function across treatment in patients with MDD: A meta-analysis. Journal of Affective Disorders, 249, 52-62. https://doi.org/https://dx.doi.org/10.1016/j.jad.2019.02.021 | Not reporting outcomes of interest |
| Bertschy, G., Velten, M., & Weibel, S. (2016). Major depression: Does gender influence the risk of recurrence? A systematic review. European Journal of Psychiatry, 30(1), 7-27. http://scielo.isciii.es/pdf/ejpen/v30n1/original01.pdfhttp://ovidsp.ovid.com/ovidweb.cgi?T=JS&PAGE=reference&D=emed17&NEWS=N&AN=609920856 | Univariable models or correlational studies |
| Berwian, I. M., Walter, H., Seifritz, E., & Huys, Q. J. M. (2017). Predicting relapse after antidepressant withdrawal - a systematic review. Psychological medicine, 47(3), 426-437. https://doi.org/https://dx.doi.org/10.1017/S0033291716002580 | Only searched one database |
| Biagianti, B., Conelea, C., Brambilla, P., & Bernstein, G. (2020). A systematic review of treatments targeting cognitive biases in socially anxious adolescents: Special Section on "Translational and Neuroscience Studies in Affective Disorders" Section Editor, Maria Nobile MD, PhD. Journal of Affective Disorders, 264, 543-551. https://doi.org/https://dx.doi.org/10.1016/j.jad.2019.12.002 | Not investigating prognostic or predictive factors |
| Biagianti, B., Bigoni, D., Maggioni, E., & Brambilla, P. (2022). Can neuroimaging-based biomarkers predict response to cognitive remediation in patients with psychosis? A state-of-the-art review. *Journal of affective disorders*, *305*, 196–205. | No includible individual studies |
| Biondi, M., & D'Orazio, M. (2010). Long-term course of treatment strategies in panic disorder: An update narrative review 1999-2010. Rivista di Psichiatria, 45(3), 123-144. http://www.rivistadipsichiatria.it/allegati/00497_2010_03/fulltext/01-Biondi%20(123-144).pdfhttp://ovidsp.ovid.com/ovidweb.cgi?T=JS&PAGE=reference&D=emed11&NEWS=N&AN=359232019 | Not reporting outcomes of interest |
| Blasco, B. V., Garcia-Jimenez, J., Bodoano, I., & Gutierrez-Rojas, L. (2020). Obesity and Depression: Its Prevalence and Influence as a Prognostic Factor: A Systematic Review. Psychiatry Investigation, 17(8), 715-724. https://doi.org/https://dx.doi.org/10.30773/pi.2020.0099 | Univariable models or correlational studies |
| Bleys, D., Luyten, P., Soenens, B., & Claes, S. (2018). Gene-environment interactions between stress and 5-HTTLPR in depression: A meta-analytic update. Journal of Affective Disorders, 226, 339-345. https://doi.org/https://dx.doi.org/10.1016/j.jad.2017.09.050 | Not reporting outcomes of interest |
| Bond, K., & Anderson, I. M. (2015). Psychoeducation for relapse prevention in bipolar disorder: a systematic review of efficacy in randomized controlled trials. Bipolar Disorders, 17(4), 349-362. https://doi.org/https://dx.doi.org/10.1111/bdi.12287 | Not reporting outcomes of interest |
| Bonvicini, C., Cortese, S., Maj, C., Baune, B. T., Faraone, S. V., & Scassellati, C. (2020). DRD4 48 bp multiallelic variants as age-population-specific biomarkers in attention-deficit/hyperactivity disorder. Translational psychiatry, 10(1), 70. https://doi.org/https://dx.doi.org/10.1038/s41398-020-0755-4 | Study design not eligible |
| Boonstra, N., Klaassen, R., Sytema, S., Marshall, M., De Haan, L., Wunderink, L., & Wiersma, D. (2012). Duration of untreated psychosis and negative symptoms--a systematic review and meta-analysis of individual patient data. Schizophrenia Research, 142(1-3), 12-19. https://doi.org/https://dx.doi.org/10.1016/j.schres.2012.08.017 | Univariable models or correlational studies |
| Borschmann, R., Henderson, C., Hogg, J., Phillips, R., & Moran, P. (2012). Crisis interventions for people with borderline personality disorder. The Cochrane database of systematic reviews(6), CD009353. https://doi.org/https://dx.doi.org/10.1002/14651858.CD009353.pub2 | Not reporting outcomes of interest |
| Bourke, M., Patten, R. K., Klamert, L., Klepac, B., Dash, S., & Pascoe, M. C. (2022). The acute affective response to physical activity in people with depression: A meta-analysis. Journal of Affective Disorders, 311, 353-363. https://doi.org/https://dx.doi.org/10.1016/j.jad.2022.05.089 | Not reporting outcomes of interest |
| Bowden, C. L. (2003). Valproate. Bipolar Disorders, 5(3), 189-202. http://ovidsp.ovid.com/ovidweb.cgi?T=JS&PAGE=reference&D=med5&NEWS=N&AN=12780873 | Study design not eligible |
| Bozzatello, P., Bellino, S., & Rocca, P. (2019). Predictive Factors of Treatment Resistance in First Episode of Psychosis: A Systematic Review. Frontiers in Psychiatry, 10, 67. https://doi.org/https://dx.doi.org/10.3389/fpsyt.2019.00067 | Only searched one database |
| Brakoulias, V., & Stockings, E. (2019). A systematic review of the use of risperidone, paliperidone and aripiprazole as augmenting agents for obsessive-compulsive disorder. Expert Opinion on Pharmacotherapy, 20(1), 47-53. https://doi.org/https://dx.doi.org/10.1080/14656566.2018.1540590 | Not investigating prognostic or predictive factors |
| Breilmann, J., Furukawa, T. A., Becker, T., & Koesters, M. (2018). Differences in the placebo response in duloxetine and venlafaxine trials. *Acta psychiatrica Scandinavica*, *137*(6), 472–480. https://doi.org/10.1111/acps.12881 | No includible individual studies |
| Brewer, D. D., Catalano, R. F., Haggerty, K., Gainey, R. R., & Fleming, C. B. (1998). A meta-analysis of predictors of continued drug use during and after treatment for opiate addiction. Addiction (Abingdon, England), 93(1), 73-92. http://ovidsp.ovid.com/ovidweb.cgi?T=JS&PAGE=reference&D=med4&NEWS=N&AN=9624713 | Only searched one database |
| Brown, J. C., Huedo-Medina, T. B., Pescatello, L. S., Ryan, S. M., Pescatello, S. M., Moker, E., LaCroix, J. M., Ferrer, R. A., & Johnson, B. T. (2012). The efficacy of exercise in reducing depressive symptoms among cancer survivors: A meta-analysis. PLoS ONE, 7(1), e30955. https://doi.org/https://dx.doi.org/10.1371/journal.pone.0030955 | Patient population not eligible |
| Brunoni, A. R., Moffa, A. H., Fregni, F., Palm, U., Padberg, F., Blumberger, D. M., Daskalakis, Z. J., Bennabi, D., Haffen, E., Alonzo, A., & Loo, C. K. (2016). Transcranial direct current stimulation for acute major depressive episodes: meta-analysis of individual patient data. The British journal of psychiatry : the journal of mental science, 208(6), 522–531. https://doi.org/10.1192/bjp.bp.115.164715 | No includible individual studies |
| Buchanan-Pascall, S., Gray, K. M., Gordon, M., & Melvin, G. A. (2018). Systematic Review and Meta-analysis of Parent Group Interventions for Primary School Children Aged 4-12 Years with Externalizing and/or Internalizing Problems. Child psychiatry and human development, 49(2), 244-267. https://doi.org/https://dx.doi.org/10.1007/s10578-017-0745-9 | Patient population not eligible |
| Buckley, P. F., & Shendarkar, N. (2005). Treatment-refractory schizophrenia. Current Opinion in Psychiatry, 18(2), 165-173. https://doi.org/http://dx.doi.org/10.1097/00001504-200503000-00010 | Study design not eligible |
| Buckman, J. E. J., Saunders, R., Stott, J., Arundell, L. L., O'Driscoll, C., Davies, M. R., Eley, T. C., Hollon, S. D., Kendrick, T., Ambler, G., Cohen, Z. D., Watkins, E., Gilbody, S., Wiles, N., Kessler, D., Richards, D., Brabyn, S., Littlewood, E., DeRubeis, R. J., Lewis, G., … Pilling, S. (2021). Role of age, gender and marital status in prognosis for adults with depression: An individual patient data meta-analysis. *Epidemiology and psychiatric sciences*, *30*, e42. https://doi.org/10.1017/S2045796021000342 | No includible individual studies |
| Burdick, K. E., Millett, C. E., Yocum, A. K., Altimus, C. M., Andreassen, O. A., Aubin, V., Belzeaux, R., Berk, M., Biernacka, J. M., Blumberg, H. P., Cleare, A. J., Diaz-Byrd, C., Dubertret, C., Etain, B., Eyler, L. T., Forester, B. P., Fullerton, J. M., Frye, M. A., Gard, S., Godin, O., Haffen, E., Klaus, F., Lagerberg, T. V., Leboyer, M., Martinez-Aran, A., McElroy, S., Mitchell, P. B., Olie, E., Olorunfemi, P., Passerieux, C., Peters, A. T., Pham, D. L., Polosan, M., Potter, J. R., Sajatovic, M., Samalin, L., Schwan, R., Shanahan, M., Sole, B., Strawbridge, R., Stuart, A. L., Torres, I., Ueland, T., Vieta, E., Williams, L. J., Wrobel, A. L., Yatham, L. N., Young, A. H., Nierenberg, A. A., & McInnis, M. G. (2022). Predictors of functional impairment in bipolar disorder: Results from 13 cohorts from seven countries by the global bipolar cohort collaborative. Bipolar Disorders. https://doi.org/https://dx.doi.org/10.1111/bdi.13208 | Not reporting outcomes of interest |
| Burton, A. W., Deer, T., Wallace, M. S., Rauck, R. L., & Grigsby, E. (2010). Considerations and methodology for trialing ziconotide. Pain Physician, 13(1), 23-33. http://www.painphysicianjournal.com/2010/january/2010;16;23-33.pdfhttp://ovidsp.ovid.com/ovidweb.cgi?T=JS&PAGE=reference&D=emed11&NEWS=N&AN=358251232 | Patient population not eligible |
| Butler, R., Berry, K., Varese, F., & Bucci, S. (2019). Are family warmth and positive remarks related to outcomes in psychosis? A systematic review. *Psychological medicine*, *49*(8), 1250–1265. https://doi.org/10.1017/S0033291718003768 | No includible individual studies |
| Butzlaff, R. L., & Hooley, J. M. (1998). Expressed emotion and psychiatric relapse: a meta-analysis. Archives of general psychiatry, 55(6), 547-552. <http://ovidsp.ovid.com/ovidweb.cgi?T=JS&PAGE=reference&D=med4&NEWS=N&AN=9633674> | Only searched one database |
| Cailhol, L., Bui, E., Rouillon, L., Bruno, N., Lemoalle, A., Faure, K., Klein, R., Lamy, P., Guelfi, J. D., & Schmitt, L. (2011). Differential indications for psychotherapies in borderline personality disorder. Encephale, 37(SUPPL. 1), S77-S82. https://doi.org/https://dx.doi.org/10.1016/j.encep.2010.04.002 | Study design not eligible |
| Cannon, J., O'Brien, A. M., Bungert, L., & Sinha, P. (2021). Prediction in Autism Spectrum Disorder: A Systematic Review of Empirical Evidence. Autism research : official journal of the International Society for Autism Research, 14(4), 604-630. https://doi.org/https://dx.doi.org/10.1002/aur.2482 | Not reporting outcomes of interest |
| Carbon, M., & Correll, C. U. (2014). Clinical predictors of therapeutic response to antipsychotics in schizophrenia. Dialogues in Clinical Neuroscience, 16(4), 505-524. http://www.ncbi.nlm.nih.gov/pmc/articles/PMC4336916/pdf/DialoguesClinNeurosci-16-505.pdfhttp://ovidsp.ovid.com/ovidweb.cgi?T=JS&PAGE=reference&D=emed15&NEWS=N&AN=603243506 | Study design not eligible |
| Carr, C. P., Martins, C. M. S., Stingel, A. M., Lemgruber, V. B., & Juruena, M. F. (2013). The role of early life stress in adult psychiatric disorders: a systematic review according to childhood trauma subtypes. The Journal of nervous and mental disease, 201(12), 1007-1020. https://doi.org/https://dx.doi.org/10.1097/NMD.0000000000000049 | Not reporting outcomes of interest |
| Castells, X., Saez, M., Barcheni, M., Cunill, R., Serrano, D., Lopez, B., & van Lissa, C. J. (2022). Placebo Response and Its Predictors in Attention Deficit Hyperactivity Disorder: A Meta-Analysis and Comparison of Meta-Regression and MetaForest. The international journal of neuropsychopharmacology, 25(1), 26-35. https://doi.org/https://dx.doi.org/10.1093/ijnp/pyab054 | Patient population not eligible |
| Caye, A., Spadini, A. V., Karam, R. G., Grevet, E. H., Rovaris, D. L., Bau, C. H., Rohde, L. A., & Kieling, C. (2016). Predictors of persistence of ADHD into adulthood: a systematic review of the literature and meta-analysis. *European child & adolescent psychiatry*, *25*(11), 1151–1159. https://doi.org/10.1007/s00787-016-0831-8 | No includible individual studies |
| Chang, P. G. R. Y., Delgadillo, J., & Waller, G. (2021). Early response to psychological treatment for eating disorders: A systematic review and meta-analysis. Clinical Psychology Review, 86, 102032. https://doi.org/https://dx.doi.org/10.1016/j.cpr.2021.102032 | Not including baseline factors |
| Chakrabarty, T., Ogrodniczuk, J., & Hadjipavlou, G. (2016). Predictive Neuroimaging Markers of Psychotherapy Response: A Systematic Review. *Harvard review of psychiatry*, *24*(6), 396–405. https://doi.org/10.1097/HRP.0000000000000132 | No includible individual studies |
| Chi, K. F., Korgaonkar, M., & Grieve, S. M. (2015). Imaging predictors of remission to anti-depressant medications in major depressive disorder. Journal of Affective Disorders, 186, 134-144. https://doi.org/https://dx.doi.org/10.1016/j.jad.2015.07.002 | Only searched one database |
| Chu, C. L., Liang, C. K., Lin, Y. T., Chow, P. C., Pan, C. C., Chou, M. Y., & Lu, T. (2011). Biomarkers of delirium: Well evidenced or not? Journal of Clinical Gerontology and Geriatrics, 2(4), 100-104. https://doi.org/https://dx.doi.org/10.1016/j.jcgg.2011.11.005 | Study design not eligible |
| Cipriani, A., Hawton, K., Stockton, S., & Geddes, J. R. (2013). Lithium in the prevention of suicide in mood disorders: updated systematic review and meta-analysis. BMJ (Clinical research ed.), 346, f3646. https://doi.org/https://dx.doi.org/10.1136/bmj.f3646 | Not reporting outcomes of interest |
| Citrome, L. (2009). Asenapine for schizophrenia and bipolar disorder: a review of the efficacy and safety profile for this newly approved sublingually absorbed second-generation antipsychotic. International journal of clinical practice, 63(12), 1762-1784. https://doi.org/https://dx.doi.org/10.1111/j.1742-1241.2009.02228.x | Not investigating prognostic or predictive factors |
| Clark, C. R., Galletly, C. A., Ash, D. J., Moores, K. A., Penrose, R. A., & McFarlane, A. C. (2009). Evidence-based medicine evaluation of electrophysiological studies of the anxiety disorders. Clinical EEG and Neuroscience, 40(2), 84-112. https://doi.org/http://dx.doi.org/10.1177/155005940904000208 | Not reporting outcomes of interest |
| Cole, M. G. (1990). The prognosis of depression in the elderly. CMAJ, 143(7), 633-639. http://ovidsp.ovid.com/ovidweb.cgi?T=JS&PAGE=reference&D=emed4&NEWS=N&AN=20374142 | Univariable models or correlational studies |
| Cole, M. G., & Bellavance, F. (1997). Depression in elderly medical inpatients: a meta-analysis of outcomes. CMAJ : Canadian Medical Association journal = journal de l'Association medicale canadienne, 157(8), 1055-1060. http://ovidsp.ovid.com/ovidweb.cgi?T=JS&PAGE=reference&D=med4&NEWS=N&AN=9347776 | Patient population not eligible |
| Cole, M. G., Bellavance, F., & Mansour, A. (1999). Prognosis of depression in elderly community and primary care populations: a systematic review and meta-analysis. The American journal of psychiatry, 156(8), 1182-1189. http://ovidsp.ovid.com/ovidweb.cgi?T=JS&PAGE=reference&D=med4&NEWS=N&AN=10450258 | Not reporting outcomes of interest |
| Colvonen, P. J., Glassman, L. H., Crocker, L. D., Buttner, M. M., Orff, H., Schiehser, D. M., Norman, S. B., & Afari, N. (2017). Pretreatment biomarkers predicting PTSD psychotherapy outcomes: A systematic review. *Neuroscience and biobehavioral reviews*, *75*, 140–156. https://doi.org/10.1016/j.neubiorev.2017.01.027 | No includible individual studies |
| Cornelius, L. R., van der Klink, J. J. L., Groothoff, J. W., & Brouwer, S. (2011). Prognostic factors of long term disability due to mental disorders: a systematic review. Journal of occupational rehabilitation, 21(2), 259-274. https://doi.org/https://dx.doi.org/10.1007/s10926-010-9261-5 | Not reporting outcomes of interest |
| Costain, G., & Bassett, A. S. (2014). Individualizing recurrence risks for severe mental illness: Epidemiologic and molecular genetic approaches. Schizophrenia Bulletin, 40(1), 21-23. https://doi.org/https://dx.doi.org/10.1093/schbul/sbt133 | Study design not eligible |
| Coyle, C. M., & Laws, K. R. (2015). The use of ketamine as an antidepressant: a systematic review and meta-analysis. Human Psychopharmacology, 30(3), 152-163. https://doi.org/https://dx.doi.org/10.1002/hup.2475 | Univariable models or correlational studies |
| Cristea, I. A., Karyotaki, E., Hollon, S. D., Cuijpers, P., & Gentili, C. (2019). Biological markers evaluated in randomized trials of psychological treatments for depression: a systematic review and meta-analysis. *Neuroscience and biobehavioral reviews*, *101*, 32–44. https://doi.org/10.1016/j.neubiorev.2019.03.022 | No includible individual studies |
| Cuijpers, P., Weitz, E., Twisk, J., Kuehner, C., Cristea, I., David, D., DeRubeis, R. J., Dimidjian, S., Dunlop, B. W., Faramarzi, M., Hegerl, U., Jarrett, R. B., Kennedy, S. H., Kheirkhah, F., Mergl, R., Miranda, J., Mohr, D. C., Segal, Z. V., Siddique, J., Simons, A. D., Vittengl, J. R., & Hollon, S. D. (2014). Gender as predictor and moderator of outcome in cognitive behavior therapy and pharmacotherapy for adult depression: an "individual patient data" meta-analysis. Depression and Anxiety, 31(11), 941-951. https://doi.org/https://dx.doi.org/10.1002/da.22328 | Not reporting outcomes of interest |
| Danborg, P. B., & Gotzsche, P. C. (2019). Benefits and harms of antipsychotic drugs in drug-naive patients with psychosis: A systematic review. The International journal of risk & safety in medicine, 30(4), 193-201. https://doi.org/https://dx.doi.org/10.3233/JRS-195063 | Not reporting outcomes of interest |
| Danckaerts, M., Sonuga-Barke, E. J. S., Banaschewski, T., Buitelaar, J., Dopfner, M., Hollis, C., Santosh, P., Rothenberger, A., Sergeant, J., Steinhausen, H. C., Taylor, E., Zuddas, A., & Coghill, D. (2010). The quality of life of children with attention deficit/hyperactivity disorder: A systematic review. European Child and Adolescent Psychiatry, 19(2), 83-105. https://doi.org/https://dx.doi.org/10.1007/s00787-009-0046-3 | Not reporting outcomes of interest |
| Davidson, B., Suresh, H., Goubran, M., Rabin, J. S., Meng, Y., Mithani, K., Pople, C. B., Giacobbe, P., Hamani, C., & Lipsman, N. (2020). Predicting response to psychiatric surgery: a systematic review of neuroimaging findings. Journal of psychiatry & neuroscience : JPN, 45(6), 387-394. https://doi.org/https://dx.doi.org/10.1503/jpn.190208 | Patient population not eligible |
| Davis, J. M., Janicak, P. G., & Hogan, D. M. (1999). Mood stabilizers in the prevention of recurrent affective disorders: a meta-analysis. Acta Psychiatrica Scandinavica, 100(6), 406-417. http://ovidsp.ovid.com/ovidweb.cgi?T=JS&PAGE=reference&D=med4&NEWS=N&AN=10626918 | Not investigating prognostic or predictive factors |
| de Aquino Ferreira, L. F., Queiroz Pereira, F. H., Neri Benevides, A. M. L., & Aguiar Melo, M. C. (2018). Borderline personality disorder and sexual abuse: A systematic review. Psychiatry Research, 262, 70-77. https://doi.org/https://dx.doi.org/10.1016/j.psychres.2018.01.043 | Not reporting outcomes of interest |
| De Crescenzo, F., Ciliberto, M., Menghini, D., Treglia, G., Ebmeier, K. P., & Janiri, L. (2017). Is ^18^F-FDG-PET suitable to predict clinical response to the treatment of geriatric depression? A systematic review of PET studies. *Aging & mental health*, *21*(9), 889–894. https://doi.org/10.1080/13607863.2016.1247413 | No includible individual studies |
| De Haan, L., Timmer, T., Linszen, D. H., Lenior, M. E., & Wouters, L. (2001). The 5-year outcome of schizophrenia after a first admission, in the western world. A meta-analysis. Tijdschrift voor Psychiatrie, 43(8), 559-565. http://ovidsp.ovid.com/ovidweb.cgi?T=JS&PAGE=reference&D=emed7&NEWS=N&AN=32800837 | Only searched one database |
| De Hert, M., Sermon, J., Geerts, P., Vansteelandt, K., Peuskens, J., & Detraux, J. (2015). The Use of Continuous Treatment Versus Placebo or Intermittent Treatment Strategies in Stabilized Patients with Schizophrenia: A Systematic Review and Meta-Analysis of Randomized Controlled Trials with First- and Second-Generation Antipsychotics. CNS Drugs, 29(8), 637-658. https://doi.org/https://dx.doi.org/10.1007/s40263-015-0269-4 | Only searched one database |
| De Santis, K. K., Azorina, V., & Reitz, S. K. (2014). More female patients and fewer stimuli per session are associated with the short-term antidepressant properties of repetitive transcranial magnetic stimulation (rTMS): A meta-analysis of 54 sham-controlled studies published between 1997-2013. Neuropsychiatric Disease and Treatment, 10, 727-756. https://doi.org/https://dx.doi.org/10.2147/NDT.S58405 | Univariable models or correlational studies |
| de Silva, V. A., & Hanwella, R. (2012). Efficacy and tolerability of venlafaxine versus specific serotonin reuptake inhibitors in treatment of major depressive disorder: a meta-analysis of published studies. International clinical psychopharmacology, 27(1), 8-16. https://doi.org/https://dx.doi.org/10.1097/YIC.0b013e32834ce13f | Not investigating prognostic or predictive factors |
| de Vries, Y. A., Roest, A. M., Bos, E. H., Burgerhof, J. G. M., van Loo, H. M., & de Jonge, P. (2019). Predicting antidepressant response by monitoring early improvement of individual symptoms of depression: individual patient data meta-analysis. The British journal of psychiatry : the journal of mental science, 214(1), 4-10. https://doi.org/https://dx.doi.org/10.1192/bjp.2018.122 | Only searched one database |
| de Zwart, P. L., Jeronimus, B. F., & de Jonge, P. (2019). Empirical evidence for definitions of episode, remission, recovery, relapse and recurrence in depression: a systematic review. Epidemiology and psychiatric sciences, 28(5), 544-562. https://doi.org/https://dx.doi.org/10.1017/S2045796018000227 | Not investigating prognostic or predictive factors |
| Dell'osso, B., Camuri, G., Castellano, F., Vecchi, V., Benedetti, M., Bortolussi, S., & Altamura, A. C. (2011). Meta-Review of Metanalytic Studies with Repetitive Transcranial Magnetic Stimulation (rTMS) for the Treatment of Major Depression. Clinical practice and epidemiology in mental health : CP & EMH, 7, 167-177. https://doi.org/https://dx.doi.org/10.2174/1745017901107010167 | Study design not eligible |
| Deng, W., Hu, D., Xu, S., Liu, X., Zhao, J., Chen, Q., Liu, J., Zhang, Z., Jiang, W., Ma, L., Hong, X., Cheng, S., Liu, B., & Li, X. (2019). The efficacy of virtual reality exposure therapy for PTSD symptoms: A systematic review and meta-analysis. Journal of Affective Disorders, 257, 698-709. https://doi.org/https://dx.doi.org/10.1016/j.jad.2019.07.086 | Not investigating prognostic or predictive factors |
| Dew, M. A., DiMartini, A. F., Steel, J., De Vito Dabbs, A., Myaskovsky, L., Unruh, M., & Greenhouse, J. (2008). Meta-analysis of risk for relapse to substance use after transplantation of the liver or other solid organs. Liver transplantation : official publication of the American Association for the Study of Liver Diseases and the International Liver Transplantation Society, 14(2), 159-172. https://doi.org/https://dx.doi.org/10.1002/lt.21278 | Patient population not eligible |
| Dewar, M., Paradis, A., & Fortin, C. A. (2020). Identifying Trajectories and Predictors of Response to Psychotherapy for Post-Traumatic Stress Disorder in Adults: A Systematic Review of Literature. *Canadian journal of psychiatry. Revue canadienne de psychiatrie*, *65*(2), 71–86. https://doi.org/10.1177/0706743719875602 | No includible individual studies |
| Di Sciascio, G., & Cal, S. (2010). Electroconvulsive therapy in the treatment of resistant major depression. Quaderni Italiani di Psichiatria, 29(4), 140-145. https://doi.org/https://dx.doi.org/10.1016/j.quip.2010.10.003 | Study design not eligible |
| Dichter, G. S., Gibbs, D., & Smoski, M. J. (2015). A systematic review of relations between resting-state functional-MRI and treatment response in major depressive disorder. Journal of Affective Disorders, 172, 8-17. https://doi.org/https://dx.doi.org/10.1016/j.jad.2014.09.028 | Only searched one database |
| DiGangi, J. A., Gomez, D., Mendoza, L., Jason, L. A., Keys, C. B., & Koenen, K. C. (2013). Pretrauma risk factors for posttraumatic stress disorder: a systematic review of the literature. Clinical Psychology Review, 33(6), 728-744. https://doi.org/https://dx.doi.org/10.1016/j.cpr.2013.05.002 | Not reporting outcomes of interest |
| Dodd, S., & Berk, M. (2004). Predictors of antidepressant response: A selective review. International Journal of Psychiatry in Clinical Practice, 8(2), 91-100. https://doi.org/https://dx.doi.org/10.1080/13651500410005423 | Study design not eligible |
| Dodd, S., Berk, M., Kelin, K., Zhang, Q., Eriksson, E., Deberdt, W., & Craig Nelson, J. (2014). Application of the Gradient Boosted method in randomised clinical trials: Participant variables that contribute to depression treatment efficacy of duloxetine, SSRIs or placebo. Journal of Affective Disorders, 168, 284-293. https://doi.org/https://dx.doi.org/10.1016/j.jad.2014.05.014 | Study design not eligible |
| Dom, G., Francque, S., & Michielsen, P. (2010). Risk for relapse of alcohol use after liver transplantation for alcoholic liver disease: a review and proposal of a set of risk assessment criteria. Acta gastro-enterologica Belgica, 73(2), 247-251. http://ovidsp.ovid.com/ovidweb.cgi?T=JS&PAGE=reference&D=med8&NEWS=N&AN=20690564 | Study design not eligible |
| Dominguez-Salas, S., Diaz-Batanero, C., Lozano-Rojas, O. M., & Verdejo-Garcia, A. (2016). Impact of general cognition and executive function deficits on addiction treatment outcomes: Systematic review and discussion of neurocognitive pathways. Neuroscience and biobehavioral reviews, 71, 772-801. https://doi.org/https://dx.doi.org/10.1016/j.neubiorev.2016.09.030 | Univariable models or correlational studies |
| Dubicka, B., Elvins, R., Roberts, C., Chick, G., Wilkinson, P., & Goodyer, I. M. (2010). Combined treatment with cognitive-behavioural therapy in adolescent depression: meta-analysis. The British journal of psychiatry : the journal of mental science, 197(6), 433-440. https://doi.org/https://dx.doi.org/10.1192/bjp.bp.109.075853 | Not investigating prognostic or predictive factors |
| Ducasse, D., Boyer, L., Michel, P., Loundou, A., Macgregor, A., Micoulaud-Franchi, J.-A., Courtet, P., Abbar, M., Leboyer, M., & Fond, G. (2014). D2 and D3 dopamine receptor affinity predicts effectiveness of antipsychotic drugs in obsessive-compulsive disorders: a metaregression analysis. Psychopharmacology, 231(18), 3765-3770. https://doi.org/https://dx.doi.org/10.1007/s00213-014-3516-3 | Study design not eligible |
| Dumas, R., Padovani, R., Richieri, R., & Lancon, C. (2012). [Repetitive transcranial magnetic stimulation in major depression: response factor]. Stimulation magnetique transcranienne repetee dans la prise en charge des episodes depressifs majeurs : facteurs predictifs de reponse therapeutique., 38(4), 360-368. https://doi.org/https://dx.doi.org/10.1016/j.encep.2011.08.004 | Study design not eligible |
| Dunlop, B. W., Thase, M. E., Wun, C.-C., Fayyad, R., Guico-Pabia, C. J., Musgnung, J., & Ninan, P. T. (2012). A meta-analysis of factors impacting detection of antidepressant efficacy in clinical trials: the importance of academic sites. Neuropsychopharmacology : official publication of the American College of Neuropsychopharmacology, 37(13), 2830-2836. https://doi.org/https://dx.doi.org/10.1038/npp.2012.153 | Only searched one database |
| Dunn, K. E., Sigmon, S. C., Strain, E. C., Heil, S. H., & Higgins, S. T. (2011). The association between outpatient buprenorphine detoxification duration and clinical treatment outcomes: a review. Drug and alcohol dependence, 119(1-2), 1-9. https://doi.org/https://dx.doi.org/10.1016/j.drugalcdep.2011.05.033 | Study design not eligible |
| Ehret, M. J., Baker, W., & O'Neill, H. (2013). BDNF Val66Met polymorphism and lithium response: a meta-analysis. Personalized medicine, 10(8), 777-784. https://doi.org/https://dx.doi.org/10.2217/pme.13.74 | Univariable models or correlational studies |
| Emmanuel, J., Simmonds, S., & Tyrer, P. (1998). Systematic review of the outcome of anxiety and depressive disorders. British Journal of Psychiatry, 173(JULY SUPPL. 34), 35-41. https://doi.org/https://dx.doi.org/10.1192/s0007125000293501 | Only searched one database |
| Emsley, R., Oosthulzen, P., Niehaus, D., Koen, L., & Chiliza, B. (2007). Changing the course of schizophrenia - Predictors of treatment outcome revisited. South African Journal of Psychiatry, 13(1), 4-9. https://doi.org/https://dx.doi.org/10.4102/sajpsychiatry.v13i1.4 | Study design not eligible |
| Enneking, V., Leehr, E. J., Dannlowski, U., & Redlich, R. (2020). Brain structural effects of treatments for depression and biomarkers of response: a systematic review of neuroimaging studies. Psychological medicine, 50(2), 187-209. https://doi.org/https://dx.doi.org/10.1017/S0033291719003660 | Only searched one database |
| Etain, B., & Bonnet-Perrin, E. (2001). Interest of fluoxetine in obsessive-compulsive disorders in adults: A review. Encephale, 27(3), 280-289. http://ovidsp.ovid.com/ovidweb.cgi?T=JS&PAGE=reference&D=emed7&NEWS=N&AN=32678138 | Study design not eligible |
| Etkin, A. (2015). Predicting treatment response in posttraumatic stress disorder. Journal of Clinical Psychiatry, 76(8), e1035-e1036. https://doi.org/https://dx.doi.org/10.4088/JCP.14com09752 | Study design not eligible |
| Evans, K., Dougherty, D., Pollack, M., & Rauch, S. (2006). Using neuroimaging to predict treatment response in mood and anxiety disorders. Annals of Clinical Psychiatry, 18(1), 33-42. https://doi.org/https://dx.doi.org/10.1177/0143034306062814 | Study design not eligible |
| Eyre, H. A., & Baune, B. T. (2014). Assessing for unique immunomodulatory and neuroplastic profiles of physical activity subtypes: a focus on psychiatric disorders. Brain, behavior, and immunity, 39, 42-55. https://doi.org/https://dx.doi.org/10.1016/j.bbi.2013.10.026 | Not investigating prognostic or predictive factors |
| Fabbri, C., Tansey, K. E., Perlis, R. H., Hauser, J., Henigsberg, N., Maier, W., Mors, O., Placentino, A., Rietschel, M., Souery, D., Breen, G., Curtis, C., Lee, S.-H., Newhouse, S., Patel, H., O'Donovan, M., Lewis, G., Jenkins, G., Weinshilboum, R. M., Farmer, A., Aitchison, K. J., Craig, I., McGuffin, P., Schruers, K., Biernacka, J. M., Uher, R., & Lewis, C. M. (2018). Effect of cytochrome CYP2C19 metabolizing activity on antidepressant response and side effects: Meta-analysis of data from genome-wide association studies. European neuropsychopharmacology : the journal of the European College of Neuropsychopharmacology, 28(8), 945-954. https://doi.org/https://dx.doi.org/10.1016/j.euroneuro.2018.05.009 | Study design not eligible |
| Fabbri, C., Porcelli, S., & Serretti, A. (2014). From pharmacogenetics to pharmacogenomics: the way toward the personalization of antidepressant treatment. *Canadian journal of psychiatry. Revue canadienne de psychiatrie*, *59*(2), 62–75. https://doi.org/10.1177/070674371405900202 | No includible individual studies |
| Fanelli, G., Domschke, K., Minelli, A., Gennarelli, M., Martini, P., Bortolomasi, M., Maron, E., Squassina, A., Kasper, S., Zohar, J., Souery, D., Montgomery, S., Albani, D., Forloni, G., Ferentinos, P., Rujescu, D., Mendlewicz, J., De Ronchi, D., Baune, B. T., European College of Neuropsychopharmacology, P., Transcriptomics Thematic Working, G., Serretti, A., & Fabbri, C. (2022). A meta-analysis of polygenic risk scores for mood disorders, neuroticism, and schizophrenia in antidepressant response. European neuropsychopharmacology : the journal of the European College of Neuropsychopharmacology, 55, 86-95. https://doi.org/https://dx.doi.org/10.1016/j.euroneuro.2021.11.005 | Univariable models or correlational studies |
| Fareed, A., Vayalapalli, S., Casarella, J., & Drexler, K. (2012). Effect of buprenorphine dose on treatment outcome. Journal of addictive diseases, 31(1), 8-18. https://doi.org/https://dx.doi.org/10.1080/10550887.2011.642758 | Not investigating prognostic or predictive factors |
| Fassino, S., Amianto, F., Sobrero, C., & Abbate Daga, G. (2013). Does it exist a personality core of mental illness? A systematic review on core psychobiological personality traits in mental disorders. *Panminerva medica*, *55*(4), 397–413 | No includible individual studies |
| Fava, M., & Rush, A. J. (2006). Current status of augmentation and combination treatments for major depressive disorder: A literature review and a proposal for a novel approach to improve practice. Psychotherapy and Psychosomatics, 75(3), 139-153. https://doi.org/https://dx.doi.org/10.1159/000091771 | Study design not eligible |
| Fawcett, J., & Barkin, R. L. (1998). A meta-analysis of eight randomized, double-blind, controlled clinical trials of mirtazapine for the treatment of patients with major depression and symptoms of anxiety. The Journal of clinical psychiatry, 59(3), 123-127. http://ovidsp.ovid.com/ovidweb.cgi?T=JS&PAGE=reference&D=med4&NEWS=N&AN=9541155 | Not investigating prognostic or predictive factors |
| Fernandez-Rodrigues, V., Sanchez-Carro, Y., Lagunas, L. N., Rico-Uribe, L. A., Pemau, A., Diaz-Carracedo, P., Diaz-Marsa, M., Hervas, G., & de la Torre-Luque, A. (2022). Risk factors for suicidal behaviour in late-life depression: A systematic review. World journal of psychiatry, 12(1), 187-203. https://doi.org/https://dx.doi.org/10.5498/wjp.v12.i1.187 | Not reporting outcomes of interest |
| Ferrando, Cloe & Selai, Caroline. (2021). A systematic review and meta-analysis on the effectiveness of exposure and response prevention therapy in the treatment of Obsessive-Compulsive Disorder. Journal of Obsessive-Compulsive and Related Disorders. 31. 100684. 10.1016/j.jocrd.2021.100684. | No includible individual studies |
| F Guerreiro Costa LN, Carneiro BA, Alves GS, Lins Silva DH, Faria Guimaraes D, Souza LS, Bandeira ID, Beanes G, Miranda Scippa A, Quarantini LC. Metabolomics of Major Depressive Disorder: A Systematic Review of Clinical Studies. Cureus. 2022 Mar 9;14(3):e23009. doi: 10.7759/cureus.23009. PMID: 35415046; PMCID: PMC8993993. | No includible individual studies |
| Fidalgo, T. M., Morales-Quezada, J. L., Muzy, G. S., Chiavetta, N. M., Mendonca, M. E., Santana, M. V., Goncalves, O. F., Brunoni, A. R., & Fregni, F. (2014). Biological markers in noninvasive brain stimulation trials in major depressive disorder: a systematic review. *The journal of ECT*, *30*(1), 47–61. https://doi.org/10.1097/YCT.0b013e31828b34d8 | No includible individual studies |
| Fineberg, N. A., Gale, T. M., & Sivakumaran, T. (2006). A review of antipsychotics in the treatment of obsessive compulsive disorder. *Journal of psychopharmacology (Oxford, England)*, *20*(1), 97–103. https://doi.org/10.1177/0269881105056640 | No includible individual studies |
| Fineberg, N. A., Brown, A., Reghunandanan, S., & Pampaloni, I. (2012). Evidence-based pharmacotherapy of obsessive-compulsive disorder. International Journal of Neuropsychopharmacology, 15(8), 1173-1191. https://doi.org/https://dx.doi.org/10.1017/S1461145711001829 | Study design not eligible |
| Firth, J., Solmi, M., Wootton, R. E., Vancampfort, D., Schuch, F. B., Hoare, E., Gilbody, S., Torous, J., Teasdale, S. B., Jackson, S. E., Smith, L., Eaton, M., Jacka, F. N., Veronese, N., Marx, W., Ashdown-Franks, G., Siskind, D., Sarris, J., Rosenbaum, S., Carvalho, A. F., & Stubbs, B. (2020). A meta-review of "lifestyle psychiatry": the role of exercise, smoking, diet and sleep in the prevention and treatment of mental disorders. World psychiatry : official journal of the World Psychiatric Association (WPA), 19(3), 360-380. https://doi.org/https://dx.doi.org/10.1002/wps.20773 | Not reporting outcomes of interest |
| Fischer, S., Strawbridge, R., Vives, A. H., & Cleare, A. J. (2017). Cortisol as a predictor of psychological therapy response in depressive disorders: systematic review and meta-analysis. *The British journal of psychiatry : the journal of mental science*, *210*(2), 105–109. https://doi.org/10.1192/bjp.bp.115.180653 | No includible individual studies |
| Fischer, S., & Cleare, A. J. (2017). Cortisol as a predictor of psychological therapy response in anxiety disorders-Systematic review and meta-analysis. Journal of Anxiety Disorders, 47, 60-68. https://doi.org/https://dx.doi.org/10.1016/j.janxdis.2017.02.007 | Univariable models or correlational studies |
| Fisher, C. A., Skocic, S., Rutherford, K. A., & Hetrick, S. E. (2018). Family therapy approaches for anorexia nervosa. The Cochrane database of systematic reviews, 10, CD004780. https://doi.org/https://dx.doi.org/10.1002/14651858.CD004780.pub3 | Univariable models or correlational studies |
| Fleeman, N., Dundar, Y., Dickson, R., Jorgensen, A., Pushpakom, S., McLeod, C., Pirmohamed, M., & Walley, T. (2011). Cytochrome P450 testing for prescribing antipsychotics in adults with schizophrenia: systematic review and meta-analyses. The pharmacogenomics journal, 11(1), 1-14. https://doi.org/https://dx.doi.org/10.1038/tpj.2010.73 | Not investigating prognostic or predictive factors |
| Fleury, M. J., Djouini, A., Huynh, C., Tremblay, J., Ferland, F., Menard, J. M., & Belleville, G. (2016). Remission from substance use disorders: A systematic review and meta-analysis. Drug and alcohol dependence, 168, 293-306. https://doi.org/https://dx.doi.org/10.1016/j.drugalcdep.2016.08.625 | Univariable models or correlational studies |
| Florence, L., Lassi, D. L. S., Kortas, G. T., Lima, D. R., de Azevedo-Marques Perico, C., Andrade, A. G., Torales, J., Ventriglio, A., De Berardis, D., De Aquino, J. P., & Castaldelli-Maia, J. M. (2022). Brain Correlates of the Alcohol Use Disorder Pharmacotherapy Response: A Systematic Review of Neuroimaging Studies. Brain Sciences, 12(3). https://doi.org/https://dx.doi.org/10.3390/brainsci12030386 | Not reporting outcomes of interest |
| Fornaro, M., De Berardis, D., Anastasia, A., Novello, S., Fusco, A., Cattaneo, C. I., Solmi, M., Monaco, F., Veronese, N., Kim, Y. K., & de Bartolomeis, A. (2018). The identification of biomarkers predicting acute and maintenance lithium treatment response in bipolar disorder: A plea for further research attention. *Psychiatry research*, *269*, 658–672. https://doi.org/10.1016/j.psychres.2018.08.034 | No includible individual studies |
| Forster, S. E., DePhilippis, D., & Forman, S. D. (2019). "I's" on the prize: A systematic review of individual differences in Contingency Management treatment response. *Journal of substance abuse treatment*, *100*, 64–83. https://doi.org/10.1016/j.jsat.2019.03.001 | No includible individual studies |
| Fountoulakis, K. N., Kontis, D., Gonda, X., & Yatham, L. N. (2013). A systematic review of the evidence on the treatment of rapid cycling bipolar disorder. *Bipolar disorders*, *15*(2), 115–137. https://doi.org/10.1111/bdi.12045 | No includible individual studies |
| Fountoulakis, K. N., Gonda, X., Koufaki, I., Hyphantis, T., & Cloninger, C. R. (2016). The Role of Temperament in the Etiopathogenesis of Bipolar Spectrum Illness. Harvard Review of Psychiatry, 24(1), 36-52. https://doi.org/https://dx.doi.org/10.1097/HRP.0000000000000077 | Not reporting outcomes of interest |
| Fraguas, D., Díaz-Caneja, C. M., Pina-Camacho, L., Umbricht, D., & Arango, C. (2019). Predictors of Placebo Response in Pharmacological Clinical Trials of Negative Symptoms in Schizophrenia: A Meta-regression Analysis. *Schizophrenia bulletin*, *45*(1), 57–68. https://doi.org/10.1093/schbul/sbx192 | No includible individual studies |
| Freire, R. C., Hallak, J. E., Crippa, J. A., & Nardi, A. E. (2011). New treatment options for panic disorder: clinical trials from 2000 to 2010. Expert Opinion on Pharmacotherapy, 12(9), 1419-1428. https://doi.org/https://dx.doi.org/10.1517/14656566.2011.562200 | Not investigating prognostic or predictive factors |
| Fridman, M., Hodgkins, P. S., Kahle, J. S., & Erder, M. H. (2015). Predicted effect size of lisdexamfetamine treatment of attention deficit/hyperactivity disorder (ADHD) in European adults: Estimates based on indirect analysis using a systematic review and meta-regression analysis. European psychiatry : the journal of the Association of European Psychiatrists, 30(4), 521-527. https://doi.org/https://dx.doi.org/10.1016/j.eurpsy.2015.01.001 | Not reporting outcomes of interest |
| Frigerio, S., Strawbridge, R., & Young, A. H. (2021). The impact of caffeine consumption on clinical symptoms in patients with bipolar disorder: A systematic review. Bipolar Disorders, 23(3), 241-251. https://doi.org/https://dx.doi.org/10.1111/bdi.12990 | Not reporting outcomes of interest |
| Frye, M. A., Prieto, M. L., Bobo, W. V., Kung, S., Veldic, M., Alarcon, R. D., Moore, K. M., Choi, D.-S., Biernacka, J. M., & Tye, S. J. (2014). Current landscape, unmet needs, and future directions for treatment of bipolar depression. Journal of Affective Disorders, 169 Suppl 1, S17-23. https://doi.org/https://dx.doi.org/10.1016/S0165-0327(14)70005-9 | Not reporting outcomes of interest |
| Frye, R. E., Vassall, S., Kaur, G., Lewis, C., Karim, M., & Rossignol, D. (2019). Emerging biomarkers in autism spectrum disorder: a systematic review. Annals of translational medicine, 7(23), 792. https://doi.org/https://dx.doi.org/10.21037/atm.2019.11.53 | Not including baseline factors |
| Fu, C. H., Steiner, H., & Costafreda, S. G. (2013). Predictive neural biomarkers of clinical response in depression: a meta-analysis of functional and structural neuroimaging studies of pharmacological and psychological therapies. *Neurobiology of disease*, *52*, 75–83. https://doi.org/10.1016/j.nbd.2012.05.008 | No includible individual studies |
| Fusar-Poli, P., & Berger, G. (2012). Eicosapentaenoic acid interventions in schizophrenia: meta-analysis of randomized, placebo-controlled studies. Journal of Clinical Psychopharmacology, 32(2), 179-185. https://doi.org/https://dx.doi.org/10.1097/JCP.0b013e318248b7bb | Not reporting outcomes of interest |
| Gaebel, W., Grossimlinghaus, I., Kerst, A., Cohen, Y., Hinsche-Bockenholt, A., Johnson, B., Mucic, D., Petrea, I., Rossler, W., Thornicroft, G., & Zielasek, J. (2016). European Psychiatric Association (EPA) guidance on the quality of eMental health interventions in the treatment of psychotic disorders. European Archives of Psychiatry and Clinical Neuroscience, 266(2), 125-137. https://doi.org/https://dx.doi.org/10.1007/s00406-016-0677-6 | Study design not eligible |
| Galvez, V., Ho, K. A., Alonzo, A., Martin, D., George, D., & Loo, C. K. (2015). Neuromodulation Therapies for Geriatric Depression. Current Psychiatry Reports, 17(7), 59. https://doi.org/https://dx.doi.org/10.1007/s11920-015-0592-y | Study design not eligible |
| Garfield, J. B. B., Lubman, D. I., & Yucel, M. (2014). Anhedonia in substance use disorders: a systematic review of its nature, course and clinical correlates. The Australian and New Zealand journal of psychiatry, 48(1), 36-51. https://doi.org/https://dx.doi.org/10.1177/0004867413508455 | Not reporting outcomes of interest |
| Gartlehner, G., Nussbaumer, B., Gaynes, B. N., Forneris, C. A., Morgan, L. C., Kaminski-Hartenthaler, A., Greenblatt, A., Wipplinger, J., Lux, L. J., Sonis, J. H., Hofmann, J., Van Noord, M. G., & Winkler, D. (2015). Second-generation antidepressants for preventing seasonal affective disorder in adults. The Cochrane database of systematic reviews(11), CD011268. https://doi.org/https://dx.doi.org/10.1002/14651858.CD011268.pub2 | Not investigating prognostic or predictive factors |
| Gasparini, A., Callegari, C., Lucca, G., Bellini, A., Caselli, I., & Ielmini, M. (2022). Inflammatory Biomarker and Response to Antidepressant in Major Depressive Disorder: a Systematic Review and Meta-Analysis. Psychopharmacology bulletin, 52(1), 36-52. http://ovidsp.ovid.com/ovidweb.cgi?T=JS&PAGE=reference&D=med20&NEWS=N&AN=35342200 | Study design not eligible |
| Gava, I., Barbui, C., Aguglia, E., Carlino, D., Churchill, R., De Vanna, M., & McGuire, H. F. (2007). Psychological treatments versus treatment as usual for obsessive compulsive disorder (OCD). The Cochrane database of systematic reviews(2), CD005333. http://ovidsp.ovid.com/ovidweb.cgi?T=JS&PAGE=reference&D=med6&NEWS=N&AN=17443583 | Not investigating prognostic or predictive factors |
| Genetics of Personality, C., de Moor, M. H. M., van den Berg, S. M., Verweij, K. J. H., Krueger, R. F., Luciano, M., Arias Vasquez, A., Matteson, L. K., Derringer, J., Esko, T., Amin, N., Gordon, S. D., Hansell, N. K., Hart, A. B., Seppala, I., Huffman, J. E., Konte, B., Lahti, J., Lee, M., Miller, M., Nutile, T., Tanaka, T., Teumer, A., Viktorin, A., Wedenoja, J., Abecasis, G. R., Adkins, D. E., Agrawal, A., Allik, J., Appel, K., Bigdeli, T. B., Busonero, F., Campbell, H., Costa, P. T., Davey Smith, G., Davies, G., de Wit, H., Ding, J., Engelhardt, B. E., Eriksson, J. G., Fedko, I. O., Ferrucci, L., Franke, B., Giegling, I., Grucza, R., Hartmann, A. M., Heath, A. C., Heinonen, K., Henders, A. K., Homuth, G., Hottenga, J.-J., Iacono, W. G., Janzing, J., Jokela, M., Karlsson, R., Kemp, J. P., Kirkpatrick, M. G., Latvala, A., Lehtimaki, T., Liewald, D. C., Madden, P. A. F., Magri, C., Magnusson, P. K. E., Marten, J., Maschio, A., Medland, S. E., Mihailov, E., Milaneschi, Y., Montgomery, G. W., Nauck, M., Ouwens, K. G., Palotie, A., Pettersson, E., Polasek, O., Qian, Y., Pulkki-Raback, L., Raitakari, O. T., Realo, A., Rose, R. J., Ruggiero, D., Schmidt, C. O., Slutske, W. S., Sorice, R., Starr, J. M., St Pourcain, B., Sutin, A. R., Timpson, N. J., Trochet, H., Vermeulen, S., Vuoksimaa, E., Widen, E., Wouda, J., Wright, M. J., Zgaga, L., Porteous, D., Minelli, A., Palmer, A. A., Rujescu, D., Ciullo, M., Hayward, C., Rudan, I., Metspalu, A., Kaprio, J., Deary, I. J., Raikkonen, K., Wilson, J. F., Keltikangas-Jarvinen, L., Bierut, L. J., Hettema, J. M., Grabe, H. J., van Duijn, C. M., Evans, D. M., Schlessinger, D., Pedersen, N. L., Terracciano, A., McGue, M., Penninx, B. W. J. H., Martin, N. G., & Boomsma, D. I. (2015). Meta-analysis of Genome-wide Association Studies for Neuroticism, and the Polygenic Association With Major Depressive Disorder. JAMA psychiatry, 72(7), 642-650. https://doi.org/https://dx.doi.org/10.1001/jamapsychiatry.2015.0554 | Not reporting outcomes of interest |
| Gillett, G., Tomlinson, A., Efthimiou, O., & Cipriani, A. (2020). Predicting treatment effects in unipolar depression: A meta-review. *Pharmacology & therapeutics*, *212*, 107557. https://doi.org/10.1016/j.pharmthera.2020.107557 | No includible individual studies |
| Giel, K. E., Behrens, S. C., Schag, K., Martus, P., Herpertz, S., Hofmann, T., Skoda, E.-M., Voderholzer, U., von Wietersheim, J., Wild, B., Zeeck, A., Schmidt, U., Zipfel, S., & Junne, F. (2021). Efficacy of post-inpatient aftercare treatments for anorexia nervosa: a systematic review of randomized controlled trials. Journal of eating disorders, 9(1), 129. https://doi.org/https://dx.doi.org/10.1186/s40337-021-00487-5 | Not investigating prognostic or predictive factors |
| Gilden, J., Kamperman, A. M., Munk-Olsen, T., Hoogendijk, W. J. G., Kushner, S. A., & Bergink, V. (2020). Long-Term Outcomes of Postpartum Psychosis: A Systematic Review and Meta-Analysis. The Journal of clinical psychiatry, 81(2). https://doi.org/https://dx.doi.org/10.4088/JCP.19r12906 | Not reporting outcomes of interest |
| Goncalves R, Lages AC, Rodrigues H, Pedrozo AL, Freire Coutinho ES, Neylan T, Figueira I, Ventura P.  Potential biomarkers of cognitive behavior-therapy for post-traumatic stress disorder: a systematic review. Revista de Psiquiatria Clinica 2011; 38(4): 155-160. Available from: http://www.scielo.br/scielo.php?pid=S0101-60832011000400008&script=sci_abstract | No includible individual studies |
| Goossens, J., Morrens, M., & Coppens, V. (2021). The Potential Use of Peripheral Blood Mononuclear Cells as Biomarkers for Treatment Response and Outcome Prediction in Psychiatry: A Systematic Review. *Molecular diagnosis & therapy*, *25*(3), 283–299. https://doi.org/10.1007/s40291-021-00516-8 | No includible individual studies |
| Goozée, R., Handley, R., Kempton, M. J., & Dazzan, P. (2014). A systematic review and meta-analysis of the effects of antipsychotic medications on regional cerebral blood flow (rCBF) in schizophrenia: association with response to treatment. *Neuroscience and biobehavioral reviews*, *43*, 118–136. https://doi.org/10.1016/j.neubiorev.2014.03.014 | No includible individual studies |
| Gorwood, P., Bayle, F., Vaiva, G., Courtet, P., Corruble, E., & Llorca, P. M. (2013). Is it worth assessing progress as early as week 2 to adapt antidepressive treatment strategy? Results from a study on agomelatine and a global meta-analysis. European psychiatry : the journal of the Association of European Psychiatrists, 28(6), 362-371. https://doi.org/https://dx.doi.org/10.1016/j.eurpsy.2012.11.004 | Study design not eligible |
| Goslar, M., Leibetseder, M., Muench, H. M., Hofmann, S. G., & Laireiter, A.-R. (2019). Pharmacological Treatments for Disordered Gambling: A Meta-analysis. Journal of gambling studies, 35(2), 415-445. https://doi.org/https://dx.doi.org/10.1007/s10899-018-09815-y | Not reporting outcomes of interest |
| Goslar, M., Leibetseder, M., Muench, H. M., Hofmann, S. G., & Laireiter, A.-R. (2020). Treatments for internet addiction, sex addiction and compulsive buying: A meta-analysis. Journal of behavioral addictions, 9(1), 14-43. https://doi.org/https://dx.doi.org/10.1556/2006.2020.00005 | Patient population not eligible |
| Goss, A. J., Kaser, M., Costafreda, S. G., Sahakian, B. J., & Fu, C. H. (2013). Modafinil augmentation therapy in unipolar and bipolar depression: a systematic review and meta-analysis of randomized controlled trials. *The Journal of clinical psychiatry*, *74*(11), 1101–1107. https://doi.org/10.4088/JCP.13r08560 | No includible individual studies |
| Gottlieb, J. F., Goel, N., Chen, S., & Young, M. A. (2021). Meta-analysis of sleep deprivation in the acute treatment of bipolar depression. Acta Psychiatrica Scandinavica, 143(4), 319-327. https://doi.org/https://dx.doi.org/10.1111/acps.13255 | Not reporting outcomes of interest |
| Gournellis, R., & Lykouras, L. (2006). Psychotic (delusional) major depression in the elderly: A review. Current Psychiatry Reviews, 2(2), 235-244. https://doi.org/https://dx.doi.org/10.2174/157340006776876003 | Not investigating prognostic or predictive factors |
| Green, R., Du, H., Grodin, E. N., Nieto, S. J., Bujarski, S., Roche, D. J. O., & Ray, L. A. (2021). A meta-regression of methodological features that predict the effects of medications on the subjective response to alcohol. Alcoholism, clinical and experimental research, 45(7), 1336–1347. https://doi.org/10.1111/acer.14643 | No includible individual studies |
| Gregertsen, E. C., Mandy, W., Kanakam, N., Armstrong, S., & Serpell, L. (2019). Pre-treatment patient characteristics as predictors of drop-out and treatment outcome in individual and family therapy for adolescents and adults with anorexia nervosa: A systematic review and meta-analysis. Psychiatry Research, 271, 484-501. https://doi.org/https://dx.doi.org/10.1016/j.psychres.2018.11.068 | Univariable models or correlational studies |
| Grigoriou, M., & Upthegrove, R. (2020). Blunted affect and suicide in schizophrenia: A systematic review. Psychiatry Research, 293, 113355. https://doi.org/https://dx.doi.org/10.1016/j.psychres.2020.113355 | Not reporting outcomes of interest |
| Gueorguieva, R., Chekroud, A. M., & Krystal, J. H. (2017). Trajectories of relapse in randomised, placebo-controlled trials of treatment discontinuation in major depressive disorder: an individual patient-level data meta-analysis. The lancet. Psychiatry, 4(3), 230-237. https://doi.org/https://dx.doi.org/10.1016/S2215-0366(17)30038-X | Study design not eligible |
| Guo, L., Kong, Z., & Zhang, Y. (2019). Qigong-Based Therapy for Treating Adults with Major Depressive Disorder: A Meta-Analysis of Randomized Controlled Trials. International journal of environmental research and public health, 16(5). https://doi.org/https://dx.doi.org/10.3390/ijerph16050826 | Not investigating prognostic or predictive factors |
| Guo, X., McCutcheon, R. A., Pillinger, T., Mizuno, Y., Natesan, S., Brown, K., & Howes, O. (2020). The magnitude and heterogeneity of antidepressant response in depression: A meta-analysis of over 45,000 patients. Journal of Affective Disorders, 276, 991-1000. https://doi.org/https://dx.doi.org/10.1016/j.jad.2020.07.102 | Not investigating prognostic or predictive factors |
| Hageman, S. B., van Rooijen, G., Bergfeld, I. O., Schirmbeck, F., de Koning, P., Schuurman, P. R., & Denys, D. (2021). Deep brain stimulation versus ablative surgery for treatment-refractory obsessive-compulsive disorder: A meta-analysis. Acta Psychiatrica Scandinavica, 143(4), 307-318. https://doi.org/https://dx.doi.org/10.1111/acps.13276 | Not reporting outcomes of interest |
| Hallab, A., Naveed, S., Altibi, A., Abdelkhalek, M., Ngo, H. T., Le, T. P., Hirayama, K., & Huy, N. T. (2018). Association of psychosis with antiphospholipid antibody syndrome: A systematic review of clinical studies. General Hospital Psychiatry, 50, 137-147. https://doi.org/https://dx.doi.org/10.1016/j.genhosppsych.2017.11.005 | Not reporting outcomes of interest |
| Handayani, F., Setyowati, Pudjonarko, D., & Sawitri, D. R. (2019). Psychosocial associated and predictors of post stroke Depression 3- 6 months after onset: A systematic review. Pakistan Journal of Medical and Health Sciences, 13(4), 1219-1223. http://www.pjmhsonline.com/2019/oct_dec/pdf/n/1219.pdfhttp://ovidsp.ovid.com/ovidweb.cgi?T=JS&PAGE=reference&D=emed20&NEWS=N&AN=2005069924 | Not reporting outcomes of interest |
| Hartley, S., Barrowclough, C., & Haddock, G. (2013). Anxiety and depression in psychosis: a systematic review of associations with positive psychotic symptoms. Acta Psychiatrica Scandinavica, 128(5), 327-346. https://doi.org/https://dx.doi.org/10.1111/acps.12080 | Not reporting outcomes of interest |
| Hartwell, E. E., Feinn, R., Morris, P. E., Gelernter, J., Krystal, J., Arias, A. J., Hoffman, M., Petrakis, I., Gueorguieva, R., Schacht, J. P., Oslin, D., Anton, R. F., & Kranzler, H. R. (2020). Systematic review and meta-analysis of the moderating effect of rs1799971 in OPRM1, the mu-opioid receptor gene, on response to naltrexone treatment of alcohol use disorder. Addiction (Abingdon, England), 115(8), 1426-1437. https://doi.org/https://dx.doi.org/10.1111/add.14975 | Only searched one database |
| Hautzinger, M., & Meyer, T. D. (2007). [Psychotherapy for bipolar disorder : a systematic review of controlled studies]. Psychotherapie bei bipolaren affektiven Storungen : ein systematischer Uberblick kontrollierter Interventionsstudien., 78(11), 1248-1260. http://ovidsp.ovid.com/ovidweb.cgi?T=JS&PAGE=reference&D=med6&NEWS=N&AN=17604972 | Not reporting outcomes of interest |
| Heal, D. J., & Smith, S. L. (2022). Prospects for new drugs to treat binge-eating disorder: Insights from psychopathology and neuropharmacology. Journal of Psychopharmacology, 36(6), 680-703. https://doi.org/https://dx.doi.org/10.1177/02698811211032475 | Not investigating prognostic or predictive factors |
| Herrmann, L. L., & Ebmeier, K. P. (2006). Factors modifying the efficacy of transcranial magnetic stimulation in the treatment of depression: a review. The Journal of clinical psychiatry, 67(12), 1870-1876. http://ovidsp.ovid.com/ovidweb.cgi?T=JS&PAGE=reference&D=med6&NEWS=N&AN=17194264 | Not reporting outcomes of interest |
| Hermens, M. L., van Hout, H. P., Terluin, B., van der Windt, D. A., Beekman, A. T., van Dyck, R., & de Haan, M. (2004). The prognosis of minor depression in the general population: a systematic review. *General hospital psychiatry*, *26*(6), 453–462. https://doi.org/10.1016/j.genhosppsych.2004.08.006 | No includible individual studies |
| Ho, C. S. H., Lim, L. J. H., Lim, A. Q., Chan, N. H. C., Tan, R. S., Lee, S. H., & Ho, R. C. M. (2020). Diagnostic and Predictive Applications of Functional Near-Infrared Spectroscopy for Major Depressive Disorder: A Systematic Review. *Frontiers in psychiatry*, *11*, 378. https://doi.org/10.3389/fpsyt.2020.00378 | No includible individual studies |
| Hollon, S. D., Jarrett, R. B., Nierenberg, A. A., Thase, M. E., Trivedi, M., & Rush, A. J. (2005). Psychotherapy and medication in the treatment of adult and geriatric depression: Which monotherapy or combined treatment? Journal of Clinical Psychiatry, 66(4), 455-468. https://doi.org/http://dx.doi.org/10.4088/JCP.v66n0408 | Not investigating prognostic or predictive factors |
| Houghton, B., Kouimtsidis, C., Duka, T., Paloyelis, Y., & Bailey, A. (2021). Can intranasal oxytocin reduce craving in automated addictive behaviours? A systematic review. British journal of pharmacology, 178(21), 4316-4334. https://doi.org/https://dx.doi.org/10.1111/bph.15617 | Not reporting outcomes of interest |
| Huedo-Medina, T. B., Kirsch, I., Middlemass, J., Klonizakis, M., & Siriwardena, A. N. (2012). Effectiveness of non-benzodiazepine hypnotics in treatment of adult insomnia: meta-analysis of data submitted to the Food and Drug Administration. BMJ (Clinical research ed.), 345, e8343. https://doi.org/https://dx.doi.org/10.1136/bmj.e8343 | Not reporting outcomes of interest |
| Hutsebaut, J., Willemsen, E. M. C., & Vani, H. L. (2018). Time for cluster C personality disorders: State of the art. Tijdschrift voor Psychiatrie, 60(5), 306-314. http://www.tijdschriftvoorpsychiatrie.nl/http://ovidsp.ovid.com/ovidweb.cgi?T=JS&PAGE=reference&D=emed19&NEWS=N&AN=622305950 | Not reporting outcomes of interest |
| Ilardi, S.S. and Edward Head, W.C. (1994), Personality pathology and response to somatic treatments for major depression: A critical review. Depression, 2: 200-217. https://doi.org/10.1002/depr.3050020404 | No includible individual studies |
| Imai, H., Noma, H., & Furukawa, T. A. (2021). Melancholic features (DSM-IV) predict but do not moderate response to antidepressants in major depression: an individual participant data meta-analysis of 1219 patients. European Archives of Psychiatry and Clinical Neuroscience, 271(3), 521-526. https://doi.org/https://dx.doi.org/10.1007/s00406-020-01173-4 | Study design not eligible |
| Iovieno, N., Papakostas, G. I., Feeney, A., Fava, M., Mathew, S. J., Iosifescu, D. I., Murrough, J. W., Macaluso, M., Hock, R. S., & Jha, M. K. (2021). Vortioxetine Versus Placebo for Major Depressive Disorder: A Comprehensive Analysis of the Clinical Trial Dataset. The Journal of clinical psychiatry, 82(4). https://doi.org/https://dx.doi.org/10.4088/JCP.20r13682 | Not investigating prognostic or predictive factors |
| Ipser, J. C., Wilson, D., Akindipe, T. O., Sager, C., & Stein, D. J. (2015). Pharmacotherapy for anxiety and comorbid alcohol use disorders. *The Cochrane database of systematic reviews*, *1*(1), CD007505. https://doi.org/10.1002/14651858.CD007505.pub2 | No includible individual studies |
| Ito, K., & Romero, K. (2020). Placebo effect in subjects with cognitive impairment. International Review of Neurobiology, 153, 213-230. https://doi.org/https://dx.doi.org/10.1016/bs.irn.2020.03.032 | Study design not eligible |
| Jääskeläinen, E., Juola, P., Hirvonen, N., McGrath, J. J., Saha, S., Isohanni, M., Veijola, J., & Miettunen, J. (2013). A systematic review and meta-analysis of recovery in schizophrenia. *Schizophrenia bulletin*, *39*(6), 1296–1306. https://doi.org/10.1093/schbul/sbs130 | No includible individual studies |
| Jambor, T., Juhasz, G., & Eszlari, N. (2022). Towards personalised antidepressive medicine based on "big data": an up-to-date review on robust factors affecting treatment response. *Neuropsychopharmacologia Hungarica : a Magyar Pszichofarmakologiai Egyesulet lapja = official journal of the Hungarian Association of Psychopharmacology*, *24*(1), 17–28. | No includible individual studies |
| Jani, B. D., McLean, G., Nicholl, B. I., Barry, S. J., Sattar, N., Mair, F. S., & Cavanagh, J. (2015). Risk assessment and predicting outcomes in patients with depressive symptoms: a review of potential role of peripheral blood based biomarkers. *Frontiers in human neuroscience*, *9*, 18. https://doi.org/10.3389/fnhum.2015.00018 | No includible individual studies |
| Janssen, D. G., Caniato, R. N., Verster, J. C., & Baune, B. T. (2010). A psychoneuroimmunological review on cytokines involved in antidepressant treatment response. *Human psychopharmacology*, *25*(3), 201–215. https://doi.org/10.1002/hup.1103 | No includible individual studies |
| Johnson, D., Wilke, M. A. P., Lyle, S. M., Kowalec, K., Jorgensen, A., Wright, G. E. B., & Drogemoller, B. I. (2022). A Systematic Review and Analysis of the Use of Polygenic Scores in Pharmacogenomics. Clinical pharmacology and therapeutics, 111(4), 919-930. https://doi.org/https://dx.doi.org/10.1002/cpt.2520 | Not investigating prognostic or predictive factors |
| Kampman, O., & Poutanen, O. (2011). Can onset and recovery in depression be predicted by temperament? A systematic review and meta-analysis. *Journal of affective disorders*, *135*(1-3), 20–27. https://doi.org/10.1016/j.jad.2010.12.021 | No includible individual studies |
| Kambeitz, J., Romanos, M., & Ettinger, U. (2014). Meta-analysis of the association between dopamine transporter genotype and response to methylphenidate treatment in ADHD. The pharmacogenomics journal, 14(1), 77-84. https://doi.org/https://dx.doi.org/10.1038/tpj.2013.9 | Univariable models or correlational studies |
| Kappelmann, N., Lewis, G., Dantzer, R., Jones, P. B., & Khandaker, G. M. (2018). Antidepressant activity of anti-cytokine treatment: a systematic review and meta-analysis of clinical trials of chronic inflammatory conditions. Molecular psychiatry, 23(2), 335-343. https://doi.org/https://dx.doi.org/10.1038/mp.2016.167 | Univariable models or correlational studies |
| Karson, C., Duffy, R. A., Eramo, A., Nylander, A.-G., & Offord, S. J. (2016). Long-term outcomes of antipsychotic treatment in patients with first-episode schizophrenia: a systematic review. Neuropsychiatric Disease and Treatment, 12, 57-67. https://doi.org/https://dx.doi.org/10.2147/NDT.S96392 | Univariable models or correlational studies |
| Karyotaki, E., Ebert, D. D., Donkin, L., Riper, H., Twisk, J., Burger, S., Rozental, A., Lange, A., Williams, A. D., Zarski, A. C., Geraedts, A., van Straten, A., Kleiboer, A., Meyer, B., Ünlü Ince, B. B., Buntrock, C., Lehr, D., Snoek, F. J., Andrews, G., Andersson, G., … Cuijpers, P. (2018). Do guided internet-based interventions result in clinically relevant changes for patients with depression? An individual participant data meta-analysis. *Clinical psychology review*, *63*, 80–92. https://doi.org/10.1016/j.cpr.2018.06.007 | No includible individual studies |
| Karyotaki, E., Riper, H., Twisk, J., Hoogendoorn, A., Kleiboer, A., Mira, A., Mackinnon, A., Meyer, B., Botella, C., Littlewood, E., Andersson, G., Christensen, H., Klein, J. P., Schröder, J., Bretón-López, J., Scheider, J., Griffiths, K., Farrer, L., Huibers, M. J., Phillips, R., … Cuijpers, P. (2017). Efficacy of Self-guided Internet-Based Cognitive Behavioral Therapy in the Treatment of Depressive Symptoms: A Meta-analysis of Individual Participant Data. *JAMA psychiatry*, *74*(4), 351–359. https://doi.org/10.1001/jamapsychiatry.2017.0044 | No includible individual studies |
| Kaymaz, N., Drukker, M., Lieb, R., Wittchen, H. U., Werbeloff, N., Weiser, M., Lataster, T., & van Os, J. (2012). Do subthreshold psychotic experiences predict clinical outcomes in unselected non-help-seeking population-based samples? A systematic review and meta-analysis, enriched with new results. Psychological medicine, 42(11), 2239-2253. https://doi.org/https://dx.doi.org/10.1017/S0033291711002911 | Not reporting outcomes of interest |
| Kedzior, K. K., & Reitz, S. K. (2014). Short-term efficacy of repetitive transcranial magnetic stimulation (rTMS) in depression- reanalysis of data from meta-analyses up to 2010. BMC psychology, 2(1), 39. https://doi.org/https://dx.doi.org/10.1186/s40359-014-0039-y | Univariable models or correlational studies |
| Keefe, J. R., McCarthy, K. S., Dinger, U., Zilcha-Mano, S., & Barber, J. P. (2014). A meta-analytic review of psychodynamic therapies for anxiety disorders. Clinical Psychology Review, 34(4), 309-323. https://doi.org/https://dx.doi.org/10.1016/j.cpr.2014.03.004 | Univariable models or correlational studies |
| Keith, S. J., & Kane, J. M. (2003). Partial Compliance and Patient Consequences in Schizophrenia: Our Patients Can Do Better. Journal of Clinical Psychiatry, 64(11), 1308-1315. https://doi.org/http://dx.doi.org/10.4088/JCP.v64n1105 | Study design not eligible |
| Kelly, J. F., Humphreys, K., & Ferri, M. (2020). Alcoholics Anonymous and other 12-step programs for alcohol use disorder. The Cochrane database of systematic reviews, 3, CD012880. https://doi.org/https://dx.doi.org/10.1002/14651858.CD012880.pub2 | Not investigating prognostic or predictive factors |
| Kemp, D. E., Calabrese, J. R., Eudicone, J. M., Ganocy, S., Tran, Q. V., McQuade, R. D., Marcus, R. N., Vester-Blokland, E., Owen, R., & Carlson, B. X. (2010). Predictive value of early improvement in bipolar depression trials: a post-hoc pooled analysis of two 8-week aripiprazole studies. Psychopharmacology bulletin, 43(2), 5-27. http://ovidsp.ovid.com/ovidweb.cgi?T=JS&PAGE=reference&D=med8&NEWS=N&AN=21052040 | Study design not eligible |
| Kemp, J., Barker, D., Benito, K., Herren, J., & Freeman, J. (2021). Moderators of Psychosocial Treatment for Pediatric Obsessive-Compulsive Disorder: Summary and Recommendations for Future Directions. Journal of clinical child and adolescent psychology : the official journal for the Society of Clinical Child and Adolescent Psychology, American Psychological Association, Division 53, 50(4), 478-485. https://doi.org/https://dx.doi.org/10.1080/15374416.2020.1790378 | Patient population not eligible |
| Kennedy, E., Kumar, A., & Datta, S. S. (2007). Antipsychotic medication for childhood-onset schizophrenia. Cochrane Database of Systematic Reviews(3), CD004027. https://doi.org/https://dx.doi.org/10.1002/14651858.CD004027.pub2 | Not investigating prognostic or predictive factors |
| Kessing, L. V., Willer, I., Andersen, P. K., & Bukh, J. D. (2017). Rate and predictors of conversion from unipolar to bipolar disorder: A systematic review and meta-analysis. *Bipolar disorders*, *19*(5), 324–335. https://doi.org/10.1111/bdi.12513 | No includible individual studies |
| Khan, A., Leventhal, R. M., Khan, S. R., & Brown, W. A. (2002). Severity of depression and response to antidepressants and placebo: An analysis of the food and drug administration database. Journal of Clinical Psychopharmacology, 22(1), 40-45. https://doi.org/https://dx.doi.org/10.1097/00004714-200202000-00007 | Study design not eligible |
| Kho, K. H., van Vreeswijk, M. F., Simpson, S., & Zwinderman, A. H. (2003). A meta-analysis of electroconvulsive therapy efficacy in depression. The journal of ECT, 19(3), 139-147. http://ovidsp.ovid.com/ovidweb.cgi?T=JS&PAGE=reference&D=med5&NEWS=N&AN=12972983 | Patient population not eligible |
| Kim, H. K., Blumberger, D. M., Downar, J., & Daskalakis, Z. J. (2021). Systematic review of biological markers of therapeutic repetitive transcranial magnetic stimulation in neurological and psychiatric disorders. Clinical neurophysiology : official journal of the International Federation of Clinical Neurophysiology, 132(2), 429-448. https://doi.org/https://dx.doi.org/10.1016/j.clinph.2020.11.025 | Not reporting outcomes of interest |
| Kim, H. S., Hodgins, D. C., Garcia, X., Ritchie, E. V., Musani, I., McGrath, D. S., & von Ranson, K. M. (2021). A systematic review of addiction substitution in recovery: Clinical lore or empirically-based? Clinical Psychology Review, 89, 102083. https://doi.org/https://dx.doi.org/10.1016/j.cpr.2021.102083 | Not reporting outcomes of interest |
| Kishi, T., Nakamura, H., Matsuura, A., & Iwata, N. (2022). Early improvement as a predictor of response to blonanserin transdermal patch in patients with schizophrenia. Schizophrenia Research, 240, 231-232. https://doi.org/https://dx.doi.org/10.1016/j.schres.2021.12.047 | Study design not eligible |
| Kleindienst, N., Engel, R. R., & Greil, W. (2005). Psychosocial and demographic factors associated with response to prophylactic lithium: a systematic review for bipolar disorders. *Psychological medicine*, *35*(12), 1685–1694. https://doi.org/10.1017/S0033291705004484 | No includible individual studies |
| Kline, A. C., Cooper, A. A., Rytwinski, N. K., & Feeny, N. C. (2021). The Effect of Concurrent Depression on PTSD Outcomes in Trauma-Focused Psychotherapy: A Meta-Analysis of Randomized Controlled Trials. *Behavior therapy*, *52*(1), 250–266. https://doi.org/10.1016/j.beth.2020.04.015 | No includible individual studies |
| Knopp, J., Knowles, S., Bee, P., Lovell, K., & Bower, P. (2013). A systematic review of predictors and moderators of response to psychological therapies in OCD: do we have enough empirical evidence to target treatment?. *Clinical psychology review*, *33*(8), 1067–1081. https://doi.org/10.1016/j.cpr.2013.08.008 | No includible individual studies |
| Koelen, J. A., Luyten, P., Eurelings-Bontekoe, L. H. M., Diguer, L., Vermote, R., Lowyck, B., & Buhring, M. E. F. (2012). The impact of level of personality organization on treatment response: a systematic review. Psychiatry, 75(4), 355-374. https://doi.org/https://dx.doi.org/10.1521/psyc.2012.75.4.355 | Not reporting outcomes of interest |
| Kohl, S., Schonherr, D. M., Luigjes, J., Denys, D., Mueller, U. J., Lenartz, D., Visser-Vandewalle, V., & Kuhn, J. (2014). Deep brain stimulation for treatment-refractory obsessive compulsive disorder: a systematic review. BMC psychiatry, 14, 214. https://doi.org/https://dx.doi.org/10.1186/s12888-014-0214-y | Not investigating prognostic or predictive factors |
| Kohlrausch, F. B. (2013). Pharmacogenetics in schizophrenia: A review of clozapine studies. Revista Brasileira de Psiquiatria, 35(3), 305-317. https://doi.org/https://dx.doi.org/10.1590/1516-4446-2012-0970 | Univariable models or correlational studies |
| Kool, S., Schoevers, R., de Maat, S., Van, R., Molenaar, P., Vink, A., & Dekker, J. (2005). Efficacy of pharmacotherapy in depressed patients with and without personality disorders: a systematic review and meta-analysis. Journal of Affective Disorders, 88(3), 269-278. http://ovidsp.ovid.com/ovidweb.cgi?T=JS&PAGE=reference&D=med6&NEWS=N&AN=16165217 | Not investigating prognostic or predictive factors |
| Kovacs, M., & Lopez-Duran, N. (2010). Prodromal symptoms and atypical affectivity as predictors of major depression in juveniles: implications for prevention. Journal of child psychology and psychiatry, and allied disciplines, 51(4), 472-496. https://doi.org/https://dx.doi.org/10.1111/j.1469-7610.2010.02230.x | Study design not eligible |
| Kraan, T., Velthorst, E., Koenders, L., Zwaart, K., Ising, H. K., van den Berg, D., de Haan, L., & van der Gaag, M. (2016). Cannabis use and transition to psychosis in individuals at ultra-high risk: review and meta-analysis. Psychological medicine, 46(4), 673-681. https://doi.org/https://dx.doi.org/10.1017/S0033291715002329 | Not reporting outcomes of interest |
| Krause, K. R., Courtney, D. B., Chan, B. W. C., Bonato, S., Aitken, M., Relihan, J., Prebeg, M., Darnay, K., Hawke, L. D., Watson, P., & Szatmari, P. (2021). Problem-solving training as an active ingredient of treatment for youth depression: a scoping review and exploratory meta-analysis. *BMC psychiatry*, *21*(1), 397. https://doi.org/10.1186/s12888-021-03260-9 | No includible individual studies |
| Krivoy, A., Gaughran, F., Weizman, A., Breen, G., & MacCabe, J. H. (2016). Gene polymorphisms potentially related to the pharmacokinetics of clozapine: a systematic review. International clinical psychopharmacology, 31(4), 179-184. https://doi.org/https://dx.doi.org/10.1097/YIC.0000000000000065 | Not reporting outcomes of interest |
| Kuang, H., Johnson, J. A., Mulqueen, J. M., & Bloch, M. H. (2017). The efficacy of benzodiazepines as acute anxiolytics in children: A meta-analysis. Depression and Anxiety, 34(10), 888-896. https://doi.org/https://dx.doi.org/10.1002/da.22643 | Univariable models or correlational studies |
| Kucharska-Pietura, K., & Mortimer, A. (2013). Can antipsychotics improve social cognition in patients with schizophrenia? CNS Drugs, 27(5), 335-343. https://doi.org/https://dx.doi.org/10.1007/s40263-013-0047-0 | Not reporting outcomes of interest |
| Kudlow, P. A., McIntyre, R. S., & Lam, R. W. (2014). Early switching strategies in antidepressant non-responders: Current evidence and future research directions. CNS Drugs, 28(7), 601-609. https://doi.org/https://dx.doi.org/10.1007/s40263-014-0171-5 | Study design not eligible |
| Kupka, R. W., Luckenbaugh, D. A., Post, R. M., Leverich, G. S., & Nolen, W. A. (2003). Rapid and non-rapid cycling bipolar disorder: a meta-analysis of clinical studies. The Journal of clinical psychiatry, 64(12), 1483-1494. http://ovidsp.ovid.com/ovidweb.cgi?T=JS&PAGE=reference&D=med5&NEWS=N&AN=14728111 | Only searched one database |
| Kurtz, M. M. (2011). Neurocognition as a predictor of response to evidence-based psychosocial interventions in schizophrenia: What is the state of the evidence? Clinical Psychology Review, 31(4), 663-672. https://doi.org/https://dx.doi.org/10.1016/j.cpr.2011.02.008 | Not reporting outcomes of interest |
| Kurtz, M. M. (2012). Cognitive remediation for schizophrenia: current status, biological correlates and predictors of response. Expert review of neurotherapeutics, 12(7), 813-821. https://doi.org/https://dx.doi.org/10.1586/ern.12.71 | Study design not eligible |
| Lam R. W. (2012). Onset, time course and trajectories of improvement with antidepressants. *European neuropsychopharmacology : the journal of the European College of Neuropsychopharmacology*, *22 Suppl 3*, S492–S498. https://doi.org/10.1016/j.euroneuro.2012.07.005 | No includible individual studies |
| Lascar, R., Letranchant, A., Hirot, F., & Godart, N. (2021). [What factors explain the length of hospitalization for anorexia nervosa: A systematic review]. Quels facteurs expliquent la duree d'hospitalisation pour anorexie mentale: revue systematique de la litterature., 47(4), 362-368. https://doi.org/https://dx.doi.org/10.1016/j.encep.2020.11.002 | Not reporting outcomes of interest |
| Lawrie, S. M., Olabi, B., Hall, J., & McIntosh, A. M. (2011). Do we have any solid evidence of clinical utility about the pathophysiology of schizophrenia? World Psychiatry, 10(1), 19-31. https://doi.org/http://dx.doi.org/10.1002/j.2051-5545.2011.tb00004.x | Study design not eligible |
| Lee, J. C., Blumberger, D. M., Fitzgerald, P. B., Daskalakis, Z. J., & Levinson, A. J. (2012). The role of transcranial magnetic stimulation in treatment-resistant depression: a review. Current pharmaceutical design, 18(36), 5846-5852. http://ovidsp.ovid.com/ovidweb.cgi?T=JS&PAGE=reference&D=med9&NEWS=N&AN=22681165 | Study design not eligible |
| Lee, P. C., Niew, W. I., Yang, H. J., Chen, V. C. H., & Lin, K. C. (2012). A meta-analysis of behavioral parent training for children with attention deficit hyperactivity disorder. Research in Developmental Disabilities, 33(6), 2040-2049. https://doi.org/https://dx.doi.org/10.1016/j.ridd.2012.05.011 | Not reporting outcomes of interest |
| Lejoyeux, M., & Lehert, P. (2011). Alcohol-use disorders and depression: results from individual patient data meta-analysis of the acamprosate-controlled studies. *Alcohol and alcoholism (Oxford, Oxfordshire)*, *46*(1), 61–67. https://doi.org/10.1093/alcalc/agq077 | No includible individual studies |
| Lett, T. A., Walter, H., & Brandl, E. J. (2016). Pharmacogenetics and Imaging-Pharmacogenetics of Antidepressant Response: Towards Translational Strategies. CNS Drugs, 30(12), 1169-1189. http://ovidsp.ovid.com/ovidweb.cgi?T=JS&PAGE=reference&D=med13&NEWS=N&AN=27752945 | Study design not eligible |
| Leucht, S., Corves, C., Arbter, D., Engel, R. R., Li, C., & Davis, J. M. (2009). Second-generation versus first-generation antipsychotic drugs for schizophrenia: a meta-analysis. Lancet (London, England), 373(9657), 31-41. https://doi.org/https://dx.doi.org/10.1016/S0140-6736(08)61764-X | Not investigating prognostic or predictive factors |
| Leucht, S., Busch, R., Kissling, W., & Kane, J. M. (2007). Early prediction of antipsychotic nonresponse among patients with schizophrenia. *The Journal of clinical psychiatry*, *68*(3), 352–360. https://doi.org/10.4088/jcp.v68n0301 | No includible individual studies |
| Leucht, S., Chaimani, A., Leucht, C., Huhn, M., Mavridis, D., Helfer, B., Samara, M., Cipriani, A., Geddes, J. R., Salanti, G., & Davis, J. M. (2018). 60 years of placebo-controlled antipsychotic drug trials in acute schizophrenia: Meta-regression of predictors of placebo response. *Schizophrenia research*, *201*, 315–323. https://doi.org/10.1016/j.schres.2018.05.009 | No includible individual studies |
| Leucht, S., Chaimani, A., Mavridis, D., Leucht, C., Huhn, M., Helfer, B., Samara, M., Cipriani, A., Geddes, J. R., & Davis, J. M. (2019). Disconnection of drug-response and placebo-response in acute-phase antipsychotic drug trials on schizophrenia? Meta-regression analysis. *Neuropsychopharmacology : official publication of the American College of Neuropsychopharmacology*, *44*(11), 1955–1966. https://doi.org/10.1038/s41386-019-0440-6 | No includible individual studies |
| Leucht, S., Leucht, C., Huhn, M., Chaimani, A., Mavridis, D., Helfer, B., Samara, M., Rabaioli, M., Bächer, S., Cipriani, A., Geddes, J. R., Salanti, G., & Davis, J. M. (2017). Sixty Years of Placebo-Controlled Antipsychotic Drug Trials in Acute Schizophrenia: Systematic Review, Bayesian Meta-Analysis, and Meta-Regression of Efficacy Predictors. *The American journal of psychiatry*, *174*(10), 927–942. https://doi.org/10.1176/appi.ajp.2017.16121358 | No includible individual studies |
| Levy, H. C., Stevens, K. T., & Tolin, D. F. (2022). Research Review: A meta-analysis of relapse rates in cognitive behavioral therapy for anxiety and related disorders in youth. Journal of child psychology and psychiatry, and allied disciplines, 63(3), 252-260. https://doi.org/https://dx.doi.org/10.1111/jcpp.13486 | Not investigating prognostic or predictive factors |
| Li, J.-M., Zhang, Y., Su, W.-J., Liu, L.-L., Gong, H., Peng, W., & Jiang, C.-L. (2018). Cognitive behavioral therapy for treatment-resistant depression: A systematic review and meta-analysis. Psychiatry Research, 268, 243-250. https://doi.org/https://dx.doi.org/10.1016/j.psychres.2018.07.020 | Not investigating prognostic or predictive factors |
| Li, J., Loebel, A., & Meltzer, H. Y. (2018). Identifying the genetic risk factors for treatment response to lurasidone by genome-wide association study: A meta-analysis of samples from three independent clinical trials. Schizophrenia Research, 199, 203-213. https://doi.org/https://dx.doi.org/10.1016/j.schres.2018.04.006 | Study design not eligible |
| Li, Q.-F., Lu, W.-T., Zhang, Q., Zhao, Y.-D., Wu, C.-Y., & Zhou, H.-F. (2022). Proprietary Medicines Containing Bupleurum chinense DC. (Chaihu) for Depression: Network Meta-Analysis and Network Pharmacology Prediction. Frontiers in pharmacology, 13, 773537. https://doi.org/https://dx.doi.org/10.3389/fphar.2022.773537 | Not reporting outcomes of interest |
| Li, Q., Wineinger, N. E., Fu, D.-J., Libiger, O., Alphs, L., Savitz, A., Gopal, S., Cohen, N., & Schork, N. J. (2017). Genome-wide association study of paliperidone efficacy. Pharmacogenetics and genomics, 27(1), 7-18. http://ovidsp.ovid.com/ovidweb.cgi?T=JS&PAGE=reference&D=med14&NEWS=N&AN=27846195 | Study design not eligible |
| Li, Y., Huang, J., He, Y., Yang, J., Lv, Y., Liu, H., Liang, L., Li, H., Zheng, Q., & Li, L. (2019). The Impact of Placebo Response Rates on Clinical Trial Outcome: A Systematic Review and Meta-Analysis of Antidepressants in Children and Adolescents with Major Depressive Disorder. Journal of child and adolescent psychopharmacology, 29(9), 712-720. https://doi.org/https://dx.doi.org/10.1089/cap.2019.0022 | Not reporting outcomes of interest |
| Lightfoot, K., Panagiotaki, G., & Nobes, G. (2020). Effectiveness of psychological interventions for smoking cessation in adults with mental health problems: A systematic review. British journal of health psychology, 25(3), 615-638. https://doi.org/https://dx.doi.org/10.1111/bjhp.12431 | Not reporting outcomes of interest |
| Lin, Y. Y., Chang, C. C., Huang, C. C. Y., Tzeng, N. S., Kao, Y. C., & Chang, H. A. (2021). Efficacy and neurophysiological predictors of treatment response of adjunct bifrontal transcranial direct current stimulation (tDCS) in treating unipolar and bipolar depression. Journal of Affective Disorders, 280, 295-304. https://doi.org/https://dx.doi.org/10.1016/j.jad.2020.11.030 | Study design not eligible |
| Linardon, J., Wade, T., de la Piedad Garcia, X., & Brennan, L. (2017). Psychotherapy for bulimia nervosa on symptoms of depression: A meta-analysis of randomized controlled trials. *The International journal of eating disorders*, *50*(10), 1124–1136. https://doi.org/10.1002/eat.22763 | No includible individual studies |
| Liu, J., Cui, Y., Yu, L., Wen, F., Wang, F., Yan, J., Yan, C., & Li, Y. (2021). Long-Term Outcome of Pediatric Obsessive-Compulsive Disorder: A Meta-Analysis. *Journal of child and adolescent psychopharmacology*, *31*(2), 95–101. https://doi.org/10.1089/cap.2020.0051 | No includible individual studies |
| Locher, C., Kossowsky, J., Gaab, J., Kirsch, I., Bain, P., & Krummenacher, P. (2015). Moderation of antidepressant and placebo outcomes by baseline severity in late-life depression: A systematic review and meta-analysis. Journal of Affective Disorders, 181, 50-60. https://doi.org/https://dx.doi.org/10.1016/j.jad.2015.03.062 | Not reporting outcomes of interest |
| Lombardo, G., Enache, D., Gianotti, L., Schatzberg, A. F., Young, A. H., Pariante, C. M., & Mondelli, V. (2019). Baseline cortisol and the efficacy of antiglucocorticoid treatment in mood disorders: A meta-analysis. Psychoneuroendocrinology, 110, 104420. https://doi.org/https://dx.doi.org/10.1016/j.psyneuen.2019.104420 | Univariable models or correlational studies |
| Long, Z., Du, L., Zhao, J., Wu, S., Zheng, Q., & Lei, X. (2020). Prediction on treatment improvement in depression with resting state connectivity: A coordinate-based meta-analysis. *Journal of affective disorders*, *276*, 62–68. https://doi.org/10.1016/j.jad.2020.06.072 | No includible individual studies |
| Lonergan, M. (2014). Cognitive behavioral therapy for PTSD: The role of complex PTSD on treatment outcome. Journal of Aggression, Maltreatment and Trauma, 23(5), 494-512. https://doi.org/https://dx.doi.org/10.1080/10926771.2014.904467 | Study design not eligible |
| Lowe, D. J. E., Müller, D. J., & George, T. P. (2020). Ketamine Treatment in Depression: A Systematic Review of Clinical Characteristics Predicting Symptom Improvement. Current topics in medicinal chemistry, 20(15), 1398–1414. https://doi.org/10.2174/1568026620666200423094423 | No includible individual studies |
| Luchini, F., Medda, P., Mariani, M. G., Mauri, M., Toni, C., & Perugi, G. (2015). Electroconvulsive therapy in catatonic patients: Efficacy and predictors of response. *World journal of psychiatry*, *5*(2), 182–192. https://doi.org/10.5498/wjp.v5.i2.182 | No includible individual studies |
| Lynch, D., Laws, K. R., & McKenna, P. J. (2010). Cognitive behavioural therapy for major psychiatric disorder: does it really work? A meta-analytical review of well-controlled trials. Psychological medicine, 40(1), 9-24. https://doi.org/https://dx.doi.org/10.1017/S003329170900590X | Not reporting outcomes of interest |
| Ma, C. F., Chan, S. K. W., Chung, Y. L., Ng, S. M., Hui, C. L. M., Suen, Y. N., & Chen, E. Y. H. (2021). The predictive power of expressed emotion and its components in relapse of schizophrenia: a meta-analysis and meta-regression. Psychological medicine, 51(3), 365-375. https://doi.org/https://dx.doi.org/10.1017/S0033291721000209 | Univariable models or correlational studies |
| Ma, J., Zhao, M., Zhou, W., Li, M., Huai, C., Shen, L., Wang, T., Wu, H., Zhang, N., Zhang, Z., He, L., & Qin, S. (2021). Association Between the COMT Val158Met Polymorphism and Antipsychotic Efficacy in Schizophrenia: An Updated Meta-Analysis. Current neuropharmacology, 19(10), 1780-1790. https://doi.org/https://dx.doi.org/10.2174/1570159X18666201023154049 | Univariable models or correlational studies |
| Magill, M., Tonigan, J. S., Kiluk, B., Ray, L., Walthers, J., & Carroll, K. (2020). The search for mechanisms of cognitive behavioral therapy for alcohol or other drug use disorders: A systematic review. Behaviour research and therapy, 131, 103648. https://doi.org/https://dx.doi.org/10.1016/j.brat.2020.103648 | Not reporting outcomes of interest |
| Magiati, Iliana & Tay, Xiang & Howlin, Patricia. (2012). Early comprehensive behaviorally based interventions for children with autism spectrum disorders: A summary of findings from recent reviews and meta-analyses. Neuropsychiatry. 2. 543-570. 10.2217/npy.12.59. | No includible individual studies |
| Mahe, V., & Balogh, A. (2000). Long-term pharmacological treatment of generalized anxiety disorder. International clinical psychopharmacology, 15(2), 99-105. http://ovidsp.ovid.com/ovidweb.cgi?T=JS&PAGE=reference&D=med4&NEWS=N&AN=10759341 | Not reporting outcomes of interest |
| Maina, G., Rosso, G., Aguglia, A., Chiodelli, D. F., & Bogetto, F. (2011). Anxiety and bipolar disorders: Epidemiological and clinical aspects. Italian Journal of Psychopathology, 17(4), 365-375. http://ovidsp.ovid.com/ovidweb.cgi?T=JS&PAGE=reference&D=emed12&NEWS=N&AN=364075658 | Not reporting outcomes of interest |
| Malivoire, B. L., Mutschler, C., & Monson, C. M. (2020). Interpersonal dysfunction and treatment outcome in GAD: A systematic review. Journal of Anxiety Disorders, 76, 102310. https://doi.org/https://dx.doi.org/10.1016/j.janxdis.2020.102310 | Not reporting outcomes of interest |
| Mancini, M., Perna, G., Rossi, A., & Petralia, A. (2010). Use of duloxetine in patients with an anxiety disorder, or with comorbid anxiety and major depressive disorder: A review of the literature. Expert Opinion on Pharmacotherapy, 11(7), 1167-1181. https://doi.org/https://dx.doi.org/10.1517/14656561003747441 | Not investigating prognostic or predictive factors |
| Marazziti, D., & Consoli, G. (2010). Treatment strategies for obsessive-compulsive disorder. *Expert opinion on pharmacotherapy*, *11*(3), 331–343. https://doi.org/10.1517/14656560903446948 | No includible individual studies |
| Mar-Barrutia, L., Real, E., Segalás, C., Bertolín, S., Menchón, J. M., & Alonso, P. (2021). Deep brain stimulation for obsessive-compulsive disorder: A systematic review of worldwide experience after 20 years. *World journal of psychiatry*, *11*(9), 659–680. https://doi.org/10.5498/wjp.v11.i9.659 | No includible individual studies |
| Marceau, E. M., Meuldijk, D., Townsend, M. L., Solowij, N., & Grenyer, B. F. S. (2018). Biomarker correlates of psychotherapy outcomes in borderline personality disorder: A systematic review. *Neuroscience and biobehavioral reviews*, *94*, 166–178. https://doi.org/10.1016/j.neubiorev.2018.09.001 | No includible individual studies |
| Maron, E., & Nutt, D. (2015). Biological predictors of pharmacological therapy in anxiety disorders. Dialogues in Clinical Neuroscience, 17(3), 305-317. http://www.ncbi.nlm.nih.gov/pmc/articles/PMC4610615/pdf/DialoguesClinNeurosci-17-305.pdfhttp://ovidsp.ovid.com/ovidweb.cgi?T=JS&PAGE=reference&D=emed16&NEWS=N&AN=606650374 | Study design not eligible |
| Martinho, F. P., Duarte, G. S., & Couto, F. S. d. (2020). Efficacy, Effect on Mood Symptoms, and Safety of Deep Brain Stimulation in Refractory Obsessive-Compulsive Disorder: A Systematic Review and Meta-Analysis. The Journal of clinical psychiatry, 81(3). https://doi.org/https://dx.doi.org/10.4088/JCP.19r12821 | Not reporting outcomes of interest |
| Martins, D., Paduraru, M., & Paloyelis, Y. (2022). Heterogeneity in response to repeated intranasal oxytocin in schizophrenia and autism spectrum disorders: A meta-analysis of variance. British journal of pharmacology, 179(8), 1525-1543. https://doi.org/https://dx.doi.org/10.1111/bph.15451 | Not reporting outcomes of interest |
| Marwood, L., Wise, T., Perkins, A. M., & Cleare, A. J. (2018). Meta-analyses of the neural mechanisms and predictors of response to psychotherapy in depression and anxiety. Neuroscience and biobehavioral reviews, 95, 61-72. https://doi.org/https://dx.doi.org/10.1016/j.neubiorev.2018.09.022 | Univariable models or correlational studies |
| Masi, A., Lampit, A., Glozier, N., Hickie, I. B., & Guastella, A. J. (2015). Predictors of placebo response in pharmacological and dietary supplement treatment trials in pediatric autism spectrum disorder: a meta-analysis. Translational psychiatry, 5, e640. https://doi.org/https://dx.doi.org/10.1038/tp.2015.143 | Not reporting outcomes of interest |
| Mason, B. J., & Ownby, R. L. (2000). Acamprosate for the treatment of alcohol dependence: A review of double- blind, placebo-controlled trials. CNS Spectrums, 5(2), 58-69. https://doi.org/http://dx.doi.org/10.1017/S1092852900012827 | Not investigating prognostic or predictive factors |
| Mastrogiannis, D., Giamouzis, G., Dardiotis, E., Karayannis, G., Chroub-Papavaiou, A., Kremeti, D., Spiliopoulos, K., Georgoulias, P., Koutsias, S., Bonotis, K., Mantzorou, M., Skoularigis, J., Hadjigeorgiou, G. M., Butler, J., & Triposkiadis, F. (2012). Depression in patients with cardiovascular disease. Cardiology Research and Practice, 1(1), 794762. http://ovidsp.ovid.com/ovidweb.cgi?T=JS&PAGE=reference&D=emed13&NEWS=N&AN=365672899 | Study design not eligible |
| Mataix-Cols, D., Fernandez de la Cruz, L., Monzani, B., Rosenfield, D., Andersson, E., Perez-Vigil, A., Frumento, P., de Kleine, R. A., Difede, J., Dunlop, B. W., Farrell, L. J., Geller, D., Gerardi, M., Guastella, A. J., Hofmann, S. G., Hendriks, G.-J., Kushner, M. G., Lee, F. S., Lenze, E. J., Levinson, C. A., McConnell, H., Otto, M. W., Plag, J., Pollack, M. H., Ressler, K. J., Rodebaugh, T. L., Rothbaum, B. O., Scheeringa, M. S., Siewert-Siegmund, A., Smits, J. A. J., Storch, E. A., Strohle, A., Tart, C. D., Tolin, D. F., van Minnen, A., Waters, A. M., Weems, C. F., Wilhelm, S., Wyka, K., Davis, M., Ruck, C., the, D. C. S. A. C., Altemus, M., Anderson, P., Cukor, J., Finck, C., Geffken, G. R., Golfels, F., Goodman, W. K., Gutner, C., Heyman, I., Jovanovic, T., Lewin, A. B., McNamara, J. P., Murphy, T. K., Norrholm, S., & Thuras, P. (2017). D-Cycloserine Augmentation of Exposure-Based Cognitive Behavior Therapy for Anxiety, Obsessive-Compulsive, and Posttraumatic Stress Disorders: A Systematic Review and Meta-analysis of Individual Participant Data. JAMA psychiatry, 74(5), 501-510. https://doi.org/https://dx.doi.org/10.1001/jamapsychiatry.2016.3955 | Not reporting outcomes of interest |
| Matsuda, Y., Makinodan, M., Morimoto, T., & Kishimoto, T. (2019). Neural changes following cognitive remediation therapy for schizophrenia. Psychiatry and clinical neurosciences, 73(11), 676-684. https://doi.org/https://dx.doi.org/10.1111/pcn.12912 | Study design not eligible |
| McCall, H. C., Hadjistavropoulos, H. D., & Sundstrom, C. R. F. (2021). Exploring the role of persuasive design in unguided internet-delivered cognitive behavioral therapy for depression and anxiety among adults: Systematic review, meta-analysis, and meta-regression. Journal of Medical Internet Research, 23(4), e26939. https://doi.org/https://dx.doi.org/10.2196/26939 | Not reporting outcomes of interest |
| McGrath, C. A., & Abbott, M. J. (2019). Family-Based Psychological Treatment for Obsessive Compulsive Disorder in Children and Adolescents: A Meta-analysis and Systematic Review. *Clinical child and family psychology review*, *22*(4), 478–501. https://doi.org/10.1007/s10567-019-00296-y | No includible individual studies |
| McIntyre, R. S., Gorwood, P., Thase, M. E., Liss, C., Desai, D., Chen, J., & Bauer, M. (2015). Early Symptom Improvement as a Predictor of Response to Extended Release Quetiapine in Major Depressive Disorder. Journal of Clinical Psychopharmacology, 35(6), 706-710. https://doi.org/https://dx.doi.org/10.1097/JCP.0000000000000416 | Study design not eligible |
| McLaughlin, S. P. B., Barkowski, S., Burlingame, G. M., Strauss, B., & Rosendahl, J. (2019). Group psychotherapy for borderline personality disorder: A meta-analysis of randomized-controlled trials. Psychotherapy, 56(2), 260-273. https://doi.org/https://dx.doi.org/10.1037/pst0000211 | Not including baseline factors |
| McLennan, S. N., & Mathias, J. L. (2010). The depression-executive dysfunction (DED) syndrome and response to antidepressants: a meta-analytic review. International journal of geriatric psychiatry, 25(10), 933-944. https://doi.org/https://dx.doi.org/10.1002/gps.2431 | Univariable models or correlational studies |
| McPheeters, M. L., Warren, Z., Sathe, N., Bruzek, J. L., Krishnaswami, S., Jerome, R. N., & Veenstra-Vanderweele, J. (2011). A systematic review of medical treatments for children with autism spectrum disorders. Pediatrics, 127(5), e1312-1321. https://doi.org/https://dx.doi.org/10.1542/peds.2011-0427 | Not reporting outcomes of interest |
| Mechelli, A., Prata, D., Kefford, C., & Kapur, S. (2015). Predicting clinical response in people at ultra-high risk of psychosis: a systematic and quantitative review. Drug discovery today, 20(8), 924-927. https://doi.org/https://dx.doi.org/10.1016/j.drudis.2015.03.003 | Only searched one database |
| Mehta, U. M., Ibrahim, F. A., Sharma, M. S., Venkatasubramanian, G., Thirthalli, J., Bharath, R. D., Bolo, N. R., Gangadhar, B. N., & Keshavan, M. S. (2021). Resting-state functional connectivity predictors of treatment response in schizophrenia - A systematic review and meta-analysis. *Schizophrenia research*, *237*, 153–165. https://doi.org/10.1016/j.schres.2021.09.004 | No includible individual studies |
| Melhuish Beaupre, L. M., Tiwari, A. K., Goncalves, V. F., Lisoway, A. J., Harripaul, R. S., Muller, D. J., Zai, C. C., & Kennedy, J. L. (2020). Antidepressant-Associated Mania in Bipolar Disorder: A Review and Meta-analysis of Potential Clinical and Genetic Risk Factors. Journal of Clinical Psychopharmacology, 40(2), 180-185. https://doi.org/https://dx.doi.org/10.1097/JCP.0000000000001186 | Not reporting outcomes of interest |
| Meshkat, S., Rodrigues, N. B., Di Vincenzo, J. D., Ceban, F., Jaberi, S., McIntyre, R. S., Lui, L. M. W., & Rosenblat, J. D. (2021). Pharmacogenomics of ketamine: A systematic review. *Journal of psychiatric research*, *145*, 27–34. Advance online publication. https://doi.org/10.1016/j.jpsychires.2021.11.036 | No includible individual studies |
| Meszaros, A., Czobor, P., Balint, S., Simon, V., & Bitter, I. (2007). [Pharmacotherapy of adult Attention Deficit/Hyperactivity Disorder (ADHD): a systematic review]. A felnottkori figyelemhianyos/hiperaktivitas zavar (ADHD) gyogyszeres kezelesenek hatekonysaga: irodalmi attekintes., 22(4), 259-270. http://ovidsp.ovid.com/ovidweb.cgi?T=JS&PAGE=reference&D=med6&NEWS=N&AN=18167421 | Not investigating prognostic or predictive factors |
| Miljevic, A., Bailey, N. W., Herring, S. E., & Fitzgerald, P. B. (2019). Potential predictors of depressive relapse following repetitive Transcranial Magnetic Stimulation: A systematic review. *Journal of affective disorders*, *256*, 317–323. https://doi.org/10.1016/j.jad.2019.06.006 | No includible individual studies |
| Miklowitz, D. J. (2008). Adjunctive psychotherapy for bipolar disorder: State of the evidence. American Journal of Psychiatry, 165(11), 1408-1419. https://doi.org/https://dx.doi.org/10.1176/appi.ajp.2008.08040488 | Study design not eligible |
| Miklowitz, D. J., & Scott, J. (2009). Psychosocial treatments for bipolar disorder: cost-effectiveness, mediating mechanisms, and future directions. Bipolar Disorders, 11 Suppl 2, 110-122. https://doi.org/https://dx.doi.org/10.1111/j.1399-5618.2009.00715.x | Univariable models or correlational studies |
| Miley, K., Hadidi, N., Kaas, M., & Yu, F. (2020). Cognitive Training and Remediation in First-Episode Psychosis: A Literature Review. Journal of the American Psychiatric Nurses Association, 26(6), 542-554. https://doi.org/https://dx.doi.org/10.1177/1078390319877952 | Univariable models or correlational studies |
| Miller, B. J., Bodenheimer, C., & Crittenden, K. (2011). Second-generation antipsychotic discontinuation in first episode psychosis: An updated review. Clinical Psychopharmacology and Neuroscience, 9(2), 45-53. https://doi.org/http://dx.doi.org/10.9758/cpn.2011.9.2.45 | Only searched one database |
| Mishra, A., Sarangi, S. C., Maiti, R., Sood, M., & Reeta, K. H. (2022). Efficacy and Safety of Adjunctive Serotonin-Dopamine Activity Modulators in Major Depression: A Meta-Analysis of Randomized Controlled Trials. Journal of clinical pharmacology, 62(6), 721-732. https://doi.org/https://dx.doi.org/10.1002/jcph.2022 | Univariable models or correlational studies |
| Miskowiak, K. W., Mariegaard, J., Jahn, F. S., & Kjaerstad, H. L. (2022). Associations between cognition and subsequent mood episodes in patients with bipolar disorder and their unaffected relatives: A systematic review. Journal of Affective Disorders, 297, 176-188. https://doi.org/https://dx.doi.org/10.1016/j.jad.2021.10.044 | Not reporting outcomes of interest |
| Mitchell, P. B., & Loo, C. K. (2006). Transcranial magnetic stimulation for depression. The Australian and New Zealand journal of psychiatry, 40(5), 406-413. http://ovidsp.ovid.com/ovidweb.cgi?T=JS&PAGE=reference&D=med6&NEWS=N&AN=16683966 | Study design not eligible |
| Moberg, L. T., Solvang, B., Sæle, R. G., & Myrvang, A. D. (2021). Effects of cognitive-behavioral and psychodynamic-interpersonal treatments for eating disorders: a meta-analytic inquiry into the role of patient characteristics and change in eating disorder-specific and general psychopathology in remission. *Journal of eating disorders*, *9*(1), 74. https://doi.org/10.1186/s40337-021-00430-8 | No includible individual studies |
| Mocking, R. J., Harmsen, I., Assies, J., Koeter, M. W., Ruhé, H. G., & Schene, A. H. (2016). Meta-analysis and meta-regression of omega-3 polyunsaturated fatty acid supplementation for major depressive disorder. *Translational psychiatry*, *6*(3), e756. https://doi.org/10.1038/tp.2016.29 | No includible individual studies |
| Moffa, A. H., Martin, D., Alonzo, A., Bennabi, D., Blumberger, D. M., Bensenor, I. M., Daskalakis, Z., Fregni, F., Haffen, E., Lisanby, S. H., Padberg, F., Palm, U., Razza, L. B., Sampaio-Jr, B., Loo, C., & Brunoni, A. R. (2020). Efficacy and acceptability of transcranial direct current stimulation (tDCS) for major depressive disorder: An individual patient data meta-analysis. Progress in neuro-psychopharmacology & biological psychiatry, 99, 109836. https://doi.org/https://dx.doi.org/10.1016/j.pnpbp.2019.109836 | Univariable models or correlational studies |
| Mohlman, J. (2005). Does executive dysfunction affect treatment outcome in late-life mood and anxiety disorders? Journal of Geriatric Psychiatry and Neurology, 18(2), 97-108. https://doi.org/https://dx.doi.org/10.1177/0891988705276061 | Study design not eligible |
| Molent, C., Olivo, D., Wolf, R. C., Balestrieri, M., & Sambataro, F. (2019). Functional neuroimaging in treatment resistant schizophrenia: A systematic review. Neuroscience and biobehavioral reviews, 104, 178-190. https://doi.org/https://dx.doi.org/10.1016/j.neubiorev.2019.07.001 | Univariable models or correlational studies |
| Molitor, S. J., & Langberg, J. M. (2017). Using task performance to inform treatment planning for youth with ADHD: A systematic review. Clinical Psychology Review, 58, 157-173. https://doi.org/https://dx.doi.org/10.1016/j.cpr.2017.10.007 | Not reporting outcomes of interest |
| Momtazmanesh, S., Zare-Shahabadi, A., & Rezaei, N. (2019). Cytokine Alterations in Schizophrenia: An Updated Review. Frontiers in Psychiatry, 10, 892. https://doi.org/https://dx.doi.org/10.3389/fpsyt.2019.00892 | Univariable models or correlational studies |
| Moon, S. Y., Kim, M., Lho, S. K., Oh, S., Kim, S. H., & Kwon, J. S. (2021). Systematic review of the neural effect of electroconvulsive therapy in patients with schizophrenia: Hippocampus and insula as the key regions of modulation. Psychiatry Investigation, 18(6), 486-499. https://doi.org/https://dx.doi.org/10.30773/pi.2020.0438 | Not reporting outcomes of interest |
| Morel, A., Lebard, P., Dereux, A., Azuar, J., Questel, F., Bellivier, F., Marie-Claire, C., Fatseas, M., Vorspan, F., & Bloch, V. (2021). Clinical Trials of Cannabidiol for Substance Use Disorders: Outcome Measures, Surrogate Endpoints, and Biomarkers. Frontiers in Psychiatry, 12, 565617. https://doi.org/https://dx.doi.org/10.3389/fpsyt.2021.565617 | Not investigating prognostic or predictive factors |
| Moriarty, A. S., Meader, N., Snell, K. I. E., Riley, R. D., Paton, L. W., Dawson, S., Hendon, J., Chew-Graham, C. A., Gilbody, S., Churchill, R., Phillips, R. S., Ali, S., & McMillan, D. (2022). Predicting relapse or recurrence of depression: systematic review of prognostic models. *The British journal of psychiatry : the journal of mental science*, *221*(2), 448–458. https://doi.org/10.1192/bjp.2021.218 | No includible individual studies |
| Morina, N., Wicherts, J. M., Lobbrecht, J., & Priebe, S. (2014). Remission from post-traumatic stress disorder in adults: a systematic review and meta-analysis of long term outcome studies. *Clinical psychology review*, *34*(3), 249–255. https://doi.org/10.1016/j.cpr.2014.03.002 | No includible individual studies |
| Mossman, S. A., Mills, J. A., Walkup, J. T., & Strawn, J. R. (2021). The Impact of Failed Antidepressant Trials on Outcomes in Children and Adolescents with Anxiety and Depression: A Systematic Review and Meta-Analysis. Journal of child and adolescent psychopharmacology, 31(4), 259-267. https://doi.org/https://dx.doi.org/10.1089/cap.2020.0195 | Univariable models or correlational studies |
| Motter, J. N., Pimontel, M. A., Rindskopf, D., Devanand, D. P., Doraiswamy, P. M., & Sneed, J. R. (2016). Computerized cognitive training and functional recovery in major depressive disorder: A meta-analysis. Journal of Affective Disorders, 189, 184-191. https://doi.org/https://dx.doi.org/10.1016/j.jad.2015.09.022 | Univariable models or correlational studies |
| Moulton, C. D., Hopkins, C. W. P., Ismail, K., & Stahl, D. (2018). Repositioning of diabetes treatments for depressive symptoms: A systematic review and meta-analysis of clinical trials. Psychoneuroendocrinology, 94, 91-103. https://doi.org/https://dx.doi.org/10.1016/j.psyneuen.2018.05.010 | Not reporting outcomes of interest |
| Mullins, N., Perroud, N., Uher, R., Butler, A. W., Cohen-Woods, S., Rivera, M., Malki, K., Euesden, J., Power, R. A., Tansey, K. E., Jones, L., Jones, I., Craddock, N., Owen, M. J., Korszun, A., Gill, M., Mors, O., Preisig, M., Maier, W., Rietschel, M., Rice, J. P., Muller-Myhsok, B., Binder, E. B., Lucae, S., Ising, M., Craig, I. W., Farmer, A. E., McGuffin, P., Breen, G., & Lewis, C. M. (2014). Genetic relationships between suicide attempts, suicidal ideation and major psychiatric disorders: a genome-wide association and polygenic scoring study. American journal of medical genetics. Part B, Neuropsychiatric genetics : the official publication of the International Society of Psychiatric Genetics, 165B(5), 428-437. https://doi.org/https://dx.doi.org/10.1002/ajmg.b.32247 | Not reporting outcomes of interest |
| Mungo, A., Hein, M., Hubain, P., Loas, G., & Fontaine, P. (2020). Impulsivity and its Therapeutic Management in Borderline Personality Disorder: a Systematic Review. The Psychiatric quarterly, 91(4), 1333-1362. https://doi.org/https://dx.doi.org/10.1007/s11126-020-09845-z | Not investigating prognostic or predictive factors |
| Murck, H., Laughren, T., Lamers, F., Picard, R., Walther, S., Goff, D., & Sainati, S. (2015). Taking personalized medicine seriously: Biomarker approaches in phase IIb/III studies in major depression and schizophrenia. Innovations in Clinical Neuroscience, 12(3-4 Supplement A), 26S-40S. http://www.ncbi.nlm.nih.gov/pmc/articles/PMC4423313/pdf/icns_12_SupplA_26.pdfhttp://ovidsp.ovid.com/ovidweb.cgi?T=JS&PAGE=reference&D=emed16&NEWS=N&AN=605439263 | Study design not eligible |
| Musliner, K. L., Munk-Olsen, T., Eaton, W. W., & Zandi, P. P. (2016). Heterogeneity in long-term trajectories of depressive symptoms: Patterns, predictors and outcomes. *Journal of affective disorders*, *192*, 199–211. https://doi.org/10.1016/j.jad.2015.12.030 | No includible individual studies |
| Nasir, M., Li, F., Courley, S., Olten, B., & Bloch, M. H. (2021). Meta-Analysis: Pediatric Placebo Response in Depression Trials Does Not Replicate in Anxiety and Obsessive-Compulsive Disorder Trials. Journal of child and adolescent psychopharmacology, 31(10), 670-684. https://doi.org/https://dx.doi.org/10.1089/cap.2021.0030 | Not investigating prognostic or predictive factors |
| Nelson, J. C. (2010). Anxiety does not predict response to duloxetine in major depression: results of a pooled analysis of individual patient data from 11 placebo-controlled trials. Depression and Anxiety, 27(1), 12-18. https://doi.org/https://dx.doi.org/10.1002/da.20632 | Study design not eligible |
| Nelson, J. C., Delucchi, K., & Schneider, L. S. (2009). Anxiety does not predict response to antidepressant treatment in late life depression: results of a meta-analysis. International journal of geriatric psychiatry, 24(5), 539-544. https://doi.org/https://dx.doi.org/10.1002/gps.2233 | Not reporting outcomes of interest |
| Nelson, J. C., Delucchi, K. L., & Schneider, L. S. (2013). Moderators of outcome in late-life depression: a patient-level meta-analysis. *The American journal of psychiatry*, *170*(6), 651–659. https://doi.org/10.1176/appi.ajp.2012.12070927 | No includible individual studies |
| Nery, F. G., Li, W., DelBello, M. P., & Welge, J. A. (2021). N-acetylcysteine as an adjunctive treatment for bipolar depression: A systematic review and meta-analysis of randomized controlled trials. *Bipolar disorders*, *23*(7), 707–714. https://doi.org/10.1111/bdi.13039 | No includible individual studies |
| Ng, Q. X., Ramamoorthy, K., Loke, W., Lee, M. W. L., Yeo, W. S., Lim, D. Y., & Sivalingam, V. (2019). Clinical Role of Aspirin in Mood Disorders: A Systematic Review. *Brain sciences*, *9*(11), 296. https://doi.org/10.3390/brainsci9110296 | No includible individual studies |
| Nierenberg, A. A. (2003). Predictors of response to antidepressants general principles and clinical implications. The Psychiatric clinics of North America, 26(2), 345-viii. http://ovidsp.ovid.com/ovidweb.cgi?T=JS&PAGE=reference&D=med5&NEWS=N&AN=12778837 | Study design not eligible |
| Nierenberg, A. A., Ostergaard, S. D., Iovieno, N., Walker, R. S. W., Fava, M., & Papakostas, G. I. (2015). Predictors of placebo response in bipolar depression. International clinical psychopharmacology, 30(2), 59-66. https://doi.org/https://dx.doi.org/10.1097/YIC.0000000000000058 | Only searched one database |
| Noma, H., Furukawa, T. A., Maruo, K., Imai, H., Shinohara, K., Tanaka, S., Ikeda, K., Yamawaki, S., & Cipriani, A. (2019). Exploratory analyses of effect modifiers in the antidepressant treatment of major depression: Individual-participant data meta-analysis of 2803 participants in seven placebo-controlled randomized trials. Journal of Affective Disorders, 250, 419-424. https://doi.org/https://dx.doi.org/10.1016/j.jad.2019.03.031 | Univariable models or correlational studies |
| Norton, P. J., & Price, E. C. (2007). A meta-analytic review of adult cognitive-behavioral treatment outcome across the anxiety disorders. Journal of Nervous and Mental Disease, 195(6), 521-531. https://doi.org/https://dx.doi.org/10.1097/01.nmd.0000253843.70149.9a | Not reporting outcomes of interest |
| Nothling, J., Malan-Muller, S., Abrahams, N., Hemmings, S. M. J., & Seedat, S. (2020). Epigenetic alterations associated with childhood trauma and adult mental health outcomes: A systematic review. World Journal of Biological Psychiatry, 21(7), 493-512. https://doi.org/https://dx.doi.org/10.1080/15622975.2019.1583369 | Not reporting outcomes of interest |
| Nunes, E. V., & Levin, F. R. (2004). Treatment of depression in patients with alcohol or other drug dependence: a meta-analysis. *JAMA*, *291*(15), 1887–1896. https://doi.org/10.1001/jama.291.15.1887 | No includible individual studies |
| Nussbaumer-Streit, B., Greenblatt, A., Kaminski-Hartenthaler, A., Van Noord, M. G., Forneris, C. A., Morgan, L. C., Gaynes, B. N., Wipplinger, J., Lux, L. J., Winkler, D., & Gartlehner, G. (2019). Melatonin and agomelatine for preventing seasonal affective disorder. The Cochrane database of systematic reviews, 6, CD011271. https://doi.org/https://dx.doi.org/10.1002/14651858.CD011271.pub3 | Univariable models or correlational studies |
| Nussbaumer, B., Kaminski-Hartenthaler, A., Forneris, C. A., Morgan, L. C., Sonis, J. H., Gaynes, B. N., Greenblatt, A., Wipplinger, J., Lux, L. J., Winkler, D., Van Noord, M. G., Hofmann, J., & Gartlehner, G. (2015). Light therapy for preventing seasonal affective disorder. The Cochrane database of systematic reviews(11), CD011269. https://doi.org/https://dx.doi.org/10.1002/14651858.CD011269.pub2 | Not investigating prognostic or predictive factors |
| Oeztuerk, O. F., Pigoni, A., Antonucci, L. A., & Koutsouleris, N. (2022). Association between formal thought disorders, neurocognition and functioning in the early stages of psychosis: a systematic review of the last half-century studies. *European archives of psychiatry and clinical neuroscience*, *272*(3), 381–393. https://doi.org/10.1007/s00406-021-01295-3 | No includible individual studies |
| Ogden, S. N., Dichter, M. E., & Bazzi, A. R. (2022). Intimate partner violence as a predictor of substance use outcomes among women: A systematic review. Addictive behaviors, 127, 107214. https://doi.org/https://dx.doi.org/10.1016/j.addbeh.2021.107214 | Not reporting outcomes of interest |
| Okhuijsen-Pfeifer, C., Sterk, A. Y., Horn, I. M., Terstappen, J., Kahn, R. S., & Luykx, J. J. (2020). Demographic and clinical features as predictors of clozapine response in patients with schizophrenia spectrum disorders: A systematic review and meta-analysis. *Neuroscience and biobehavioral reviews*, *111*, 246–252. https://doi.org/10.1016/j.neubiorev.2020.01.017 | No includible individual studies |
| Olagunju, A. T., Clark, S. R., & Baune, B. T. (2018). Clozapine and Psychosocial Function in Schizophrenia: A Systematic Review and Meta-Analysis. CNS Drugs, 32(11), 1011-1023. https://doi.org/https://dx.doi.org/10.1007/s40263-018-0565-x | Not reporting outcomes of interest |
| Olagunju, A. T., Clark, S. R., & Baune, B. T. (2019). Long-acting atypical antipsychotics in schizophrenia: A systematic review and meta-analyses of effects on functional outcome. The Australian and New Zealand journal of psychiatry, 53(6), 509-527. https://doi.org/https://dx.doi.org/10.1177/0004867419837358 | Not reporting outcomes of interest |
| Olatunji, B. O., Kauffman, B. Y., Meltzer, S., Davis, M. L., Smits, J. A., & Powers, M. B. (2014). Cognitive-behavioral therapy for hypochondriasis/health anxiety: a meta-analysis of treatment outcome and moderators. *Behaviour research and therapy*, *58*, 65–74. https://doi.org/10.1016/j.brat.2014.05.002 | No includible individual studies |
| olde Hartman, T. C., Borghuis, M. S., Lucassen, P. L., van de Laar, F. A., Speckens, A. E., & van Weel, C. (2009). Medically unexplained symptoms, somatisation disorder and hypochondriasis: course and prognosis. A systematic review. *Journal of psychosomatic research*, *66*(5), 363–377. https://doi.org/10.1016/j.jpsychores.2008.09.018 | No includible individual studies |
| Olgiati, P., Serretti, A., Souery, D., Dold, M., Kasper, S., Montgomery, S., Zohar, J., & Mendlewicz, J. (2018). Early improvement and response to antidepressant medications in adults with major depressive disorder. Meta-analysis and study of a sample with treatment-resistant depression. Journal of Affective Disorders, 227, 777-786. https://doi.org/https://dx.doi.org/10.1016/j.jad.2017.11.004 | Univariable models or correlational studies |
| Oliva, V., Fanelli, G., Kasper, S., Zohar, J., Souery, D., Montgomery, S., Albani, D., Forloni, G., Ferentinos, P., Rujescu, D., Mendlewicz, J., Kas, M. J., De Ronchi, D., Fabbri, C., & Serretti, A. (2022). Social withdrawal as a trans-diagnostic predictor of short-term remission: a meta-analysis of five clinical cohorts. International clinical psychopharmacology, 37(2), 38-45. https://doi.org/https://dx.doi.org/10.1097/YIC.0000000000000384 | Univariable models or correlational studies |
| Olivares, J. M., Sermon, J., Hemels, M., & Schreiner, A. (2013). Definitions and drivers of relapse in patients with schizophrenia: a systematic literature review. Annals of general psychiatry, 12(1), 32. https://doi.org/https://dx.doi.org/10.1186/1744-859X-12-32 | Only searched one database |
| Ori, R., Amos, T., Bergman, H., Soares-Weiser, K., Ipser, J. C., & Stein, D. J. (2015). Augmentation of cognitive and behavioural therapies (CBT) with d-cycloserine for anxiety and related disorders. The Cochrane database of systematic reviews(5), CD007803. https://doi.org/https://dx.doi.org/10.1002/14651858.CD007803.pub2 | Univariable models or correlational studies |
| Ozcan, M. E., Shivakumar, G., & Suppes, T. (2006). Treating rapid cycling bipolar disorder with novel medications. Current Psychiatry Reviews, 2(3), 361-369. https://doi.org/https://dx.doi.org/10.2174/157340006778018166 | Only searched one database |
| Pae, C. U., Seo, H. J., Lee, B. C., Seok, J. H., Jeon, H. J., Paik, J. W., Kwak, K. P., Ham, B. J., Han, C., & Lee, S. J. (2014). A meta-analysis comparing open-label versus placebo-controlled clinical trials for aripiprazole augmentation in the treatment of major depressive disorder: Lessons and promises. Psychiatry Investigation, 11(4), 371-379. https://doi.org/https://dx.doi.org/10.4306/pi.2014.11.4.371 | Not investigating prognostic or predictive factors |
| Palpacuer, C., Gallet, L., Drapier, D., Reymann, J. M., Falissard, B., & Naudet, F. (2017). Specific and non-specific effects of psychotherapeutic interventions for depression: Results from a meta-analysis of 84 studies. *Journal of psychiatric research*, *87*, 95–104. https://doi.org/10.1016/j.jpsychires.2016.12.015 | No includible individual studies |
| Papakostas, G. I., & Fava, M. (2009). Does the probability of receiving placebo influence clinical trial outcome? A meta-regression of double-blind, randomized clinical trials in MDD. European neuropsychopharmacology : the journal of the European College of Neuropsychopharmacology, 19(1), 34-40. https://doi.org/https://dx.doi.org/10.1016/j.euroneuro.2008.08.009 | Only searched one database |
| Peckmezian, T., & Paxton, S. J. (2020). A systematic review of outcomes following residential treatment for eating disorders. European eating disorders review : the journal of the Eating Disorders Association, 28(3), 246-259. https://doi.org/https://dx.doi.org/10.1002/erv.2733 | Not reporting outcomes of interest |
| Pedroso, V. S. P., Rachid, M. A., & Teixeira, A. L. (2016). Biomarkers in post-stroke depression. Current Neurovascular Research, 13(2), 163-173. https://doi.org/https://dx.doi.org/10.2174/1567202613666160219120114 | Not including baseline factors |
| Pekkala, E., & Merinder, L. (2002). Psychoeducation for schizophrenia. The Cochrane database of systematic reviews(2), CD002831. http://ovidsp.ovid.com/ovidweb.cgi?T=JS&PAGE=reference&D=med4&NEWS=N&AN=12076455 | Not investigating prognostic or predictive factors |
| Perkins, D. O., Gu, H., Boteva, K., & Lieberman, J. A. (2005). Relationship between duration of untreated psychosis and outcome in first-episode schizophrenia: a critical review and meta-analysis. The American journal of psychiatry, 162(10), 1785-1804. http://ovidsp.ovid.com/ovidweb.cgi?T=JS&PAGE=reference&D=med6&NEWS=N&AN=16199825 | Only searched one database |
| Perkins, K. A., & Scott, J. (2008). Sex differences in long-term smoking cessation rates due to nicotine patch. Nicotine and Tobacco Research, 10(7), 1245-1251. https://doi.org/https://dx.doi.org/10.1080/14622200802097506 | Not reporting outcomes of interest |
| Perlis, R. H., Iosifescu, D. V., & Renshaw, P. F. (2003). Biological predictors of treatment response in affective illness. Psychiatric Clinics of North America, 26(2), 323-344. https://doi.org/https://dx.doi.org/10.1016/S0193-953X%2802%2900112-0 | Only searched one database |
| Perlman, K., Benrimoh, D., Israel, S., Rollins, C., Brown, E., Tunteng, J.-F., You, R., You, E., Tanguay-Sela, M., Snook, E., Miresco, M., & Berlim, M. T. (2019). A systematic meta-review of predictors of antidepressant treatment outcome in major depressive disorder. Journal of Affective Disorders, 243, 503-515. https://doi.org/https://dx.doi.org/10.1016/j.jad.2018.09.067 | Univariable models or correlational studies |
| Perna, G., Alciati, A., Riva, A., Micieli, W., & Caldirola, D. (2016). Long-Term Pharmacological Treatments of Anxiety Disorders: An Updated Systematic Review. Current Psychiatry Reports, 18(3), 23. https://doi.org/https://dx.doi.org/10.1007/s11920-016-0668-3 | Only searched one database |
| Persico, A. M., Ricciardello, A., Lamberti, M., Turriziani, L., Cucinotta, F., Brogna, C., Vitiello, B., & Arango, C. (2021). The pediatric psychopharmacology of autism spectrum disorder: A systematic review - Part I: The past and the present. Progress in neuro-psychopharmacology & biological psychiatry, 110, 110326. https://doi.org/https://dx.doi.org/10.1016/j.pnpbp.2021.110326 | Not investigating prognostic or predictive factors |
| Petra, B., & Ede, F. (2012). Relapse prevention in schizophrenia - a brief review of the literature. Neuropsychopharmacologia Hungarica, 14(3), 197-200. https://doi.org/https://dx.doi.org/10.5706/nph201209005 | Study design not eligible |
| Pierce, M., Sutterland, A., Beraha, E. M., Morley, K., & van den Brink, W. (2018). Efficacy, tolerability, and safety of low-dose and high-dose baclofen in the treatment of alcohol dependence: A systematic review and meta-analysis. European neuropsychopharmacology : the journal of the European College of Neuropsychopharmacology, 28(7), 795-806. https://doi.org/https://dx.doi.org/10.1016/j.euroneuro.2018.03.017 | Not reporting outcomes of interest |
| Piet, J., & Hougaard, E. (2011). The effect of mindfulness-based cognitive therapy for prevention of relapse in recurrent major depressive disorder: A systematic review and meta-analysis. Clinical Psychology Review, 31(6), 1032-1040. https://doi.org/https://dx.doi.org/10.1016/j.cpr.2011.05.002 | Univariable models or correlational studies |
| Pigoni, A., Delvecchio, G., Madonna, D., Bressi, C., Soares, J., & Brambilla, P. (2019). Can Machine Learning help us in dealing with treatment resistant depression? A review. Journal of Affective Disorders, 259, 21-26. https://doi.org/https://dx.doi.org/10.1016/j.jad.2019.08.009 | Study design not eligible |
| Pilling, S., Bebbington, P., Kuipers, E., Garety, P., Geddes, J., Orbach, G., & Morgan, C. (2002). Psychological treatments in schizophrenia: I. Meta-analysis of family intervention and cognitive behaviour therapy. Psychological medicine, 32(5), 763-782. http://ovidsp.ovid.com/ovidweb.cgi?T=JS&PAGE=reference&D=med4&NEWS=N&AN=12171372 | Not investigating prognostic or predictive factors |
| Pimontel, M. A., Rindskopf, D., Rutherford, B. R., Brown, P. J., Roose, S. P., & Sneed, J. R. (2016). A Meta-Analysis of Executive Dysfunction and Antidepressant Treatment Response in Late-Life Depression. The American journal of geriatric psychiatry : official journal of the American Association for Geriatric Psychiatry, 24(1), 31-41. https://doi.org/https://dx.doi.org/10.1016/j.jagp.2015.05.010 | Study design not eligible |
| Pinna, F., Manchia, M., Paribello, P., & Carpiniello, B. (2020). The Impact of Alexithymia on Treatment Response in Psychiatric Disorders: A Systematic Review. *Frontiers in psychiatry*, *11*, 311. https://doi.org/10.3389/fpsyt.2020.00311 | No includible individual studies |
| Pitchot, W. (2014). Curative and preventive indications in bipolar disorder. Annales Medico-Psychologiques, 172(3), 173-176. https://doi.org/https://dx.doi.org/10.1016/j.amp.2014.02.008 | Study design not eligible |
| Pogarell, O., Juckel, G., Norra, C., Leicht, G., Karch, S., Schaaff, N., Folkerts, M., Ibrahim, A., Mulert, C., & Hegerl, U. (2007). Prediction of clinical response to antidepressants in patients with depression: Neurophysiology in clinical practice. Clinical EEG and Neuroscience, 38(2), 74-77. http://ovidsp.ovid.com/ovidweb.cgi?T=JS&PAGE=reference&D=emed10&NEWS=N&AN=46866505 | Study design not eligible |
| Polyakova, M., Stuke, K., Schuemberg, K., Mueller, K., Schoenknecht, P., & Schroeter, M. L. (2015). BDNF as a biomarker for successful treatment of mood disorders: a systematic & quantitative meta-analysis. Journal of Affective Disorders, 174, 432-440. https://doi.org/https://dx.doi.org/10.1016/j.jad.2014.11.044 | Not investigating prognostic or predictive factors |
| Porter, R. J., Douglas, K., Jordan, J., Bowie, C. R., Roiser, J., & Malhi, G. S. (2014). Psychological treatments for cognitive dysfunction in major depressive disorder: Current evidence and perspectives. CNS and Neurological Disorders - Drug Targets, 13(10), 1677-1692. https://doi.org/https://dx.doi.org/10.2174/1871527313666141130223248 | Not reporting outcomes of interest |
| Powers, M. B., & Emmelkamp, P. M. G. (2008). Virtual reality exposure therapy for anxiety disorders: A meta-analysis. Journal of Anxiety Disorders, 22(3), 561-569. http://ovidsp.ovid.com/ovidweb.cgi?T=JS&PAGE=reference&D=med7&NEWS=N&AN=17544252 | Not investigating prognostic or predictive factors |
| Poynter, B., Shuman, M., Diaz-Granados, N., Kapral, M., Grace, S. L., & Stewart, D. E. (2009). Sex differences in the prevalence of post-stroke depression: a systematic review. Psychosomatics, 50(6), 563-569. https://doi.org/https://dx.doi.org/10.1176/appi.psy.50.6.563 | Patient population not eligible |
| Prata, D., Mechelli, A., & Kapur, S. (2014). Clinically meaningful biomarkers for psychosis: a systematic and quantitative review. Neuroscience and biobehavioral reviews, 45, 134-141. https://doi.org/https://dx.doi.org/10.1016/j.neubiorev.2014.05.010 | Only searched one database |
| Prendergast, M. L., Podus, D., Chang, E., & Urada, D. (2002). The effectiveness of drug abuse treatment: a meta-analysis of comparison group studies. Drug and alcohol dependence, 67(1), 53-72. http://ovidsp.ovid.com/ovidweb.cgi?T=JS&PAGE=reference&D=med4&NEWS=N&AN=12062779 | Not reporting outcomes of interest |
| Price, R. B., Wallace, M., Kuckertz, J. M., Amir, N., Graur, S., Cummings, L., Popa, P., Carlbring, P., & Bar-Haim, Y. (2016). Pooled patient-level meta-analysis of children and adults completing a computer-based anxiety intervention targeting attentional bias. Clinical Psychology Review, 50, 37-49. https://doi.org/https://dx.doi.org/10.1016/j.cpr.2016.09.009 | Not reporting outcomes of interest |
| Provenzani, U., Salazar de Pablo, G., Arribas, M., Pillmann, F., & Fusar-Poli, P. (2021). Clinical outcomes in brief psychotic episodes: a systematic review and meta-analysis. Epidemiology and psychiatric sciences, 30, e71. https://doi.org/https://dx.doi.org/10.1017/S2045796021000548 | Only searched one database |
| Purgato, M., Gastaldon, C., Papola, D., Magni, L. R., Rossi, G., & Barbui, C. (2015). Drug dose as mediator of treatment effect in antidepressant drug trials: the case of fluoxetine. Acta Psychiatrica Scandinavica, 131(6), 408-416. <https://doi.org/https://dx.doi.org/10.1111/acps.12381> | Not reporting outcomes of interest |
| Raballo, A., Poletti, M., Preti, A., & McGorry, P. (2022). Clinical high risk for psychosis in children and adolescents: A meta-analysis of transition prevalences. Schizophrenia Research, 243, 254-261. https://doi.org/https://dx.doi.org/10.1016/j.schres.2020.03.063 | Not reporting outcomes of interest |
| Rajji, T. K., Mulsant, B. H., Lotrich, F. E., Lokker, C., & Reynolds, C. F., 3rd. (2008). Use of antidepressants in late-life depression. Drugs & aging, 25(10), 841-853. http://ovidsp.ovid.com/ovidweb.cgi?T=JS&PAGE=reference&D=med7&NEWS=N&AN=18808208 | Not investigating prognostic or predictive factors |
| Ranasinghe, I., Sin, J., Norman, I., & Lau-Walker, M. (2018). Predicting and preventing alcohol relapse in alcohol-related liver disease. British journal of nursing (Mark Allen Publishing), 27(4), 190-196. https://doi.org/https://dx.doi.org/10.12968/bjon.2018.27.4.190 | Study design not eligible |
| Rashidi, S., Jones, M., Murillo-Rodriguez, E., Machado, S., Hao, Y., & Yadollahpour, A. (2021). Transcranial direct current stimulation for auditory verbal hallucinations: a systematic review of clinical trials. Neural regeneration research, 16(4), 666-671. https://doi.org/https://dx.doi.org/10.4103/1673-5374.295315 | Not reporting outcomes of interest |
| Rayner, L., Price, A., Evans, A., Valsraj, K., Higginson, I. J., & Hotopf, M. (2010). Antidepressants for depression in physically ill people. Cochrane database of systematic reviews (Online), 3, CD007503. http://ovidsp.ovid.com/ovidweb.cgi?T=JS&PAGE=reference&D=emed11&NEWS=N&AN=358680224 | Not investigating prognostic or predictive factors |
| Razza, L. B., De Smet, S., Moffa, A., Sudbrack-Oliveira, P., Vanderhasselt, M.-A., & Brunoni, A. R. (2021). Follow-up effects of transcranial direct current stimulation (tDCS) for the major depressive episode: A systematic review and meta-analysis. Psychiatry Research, 302, 114024. https://doi.org/https://dx.doi.org/10.1016/j.psychres.2021.114024 | Not reporting outcomes of interest |
| Reas, D. L., Schoemaker, C., Zipfel, S., & Williamson, D. A. (2001). Prognostic value of duration of illness and early intervention in bulimia nervosa: A systematic review of the outcome literature. International Journal of Eating Disorders, 30(1), 1-10. https://doi.org/https://dx.doi.org/10.1002/eat.1049 | Univariable models or correlational studies |
| Reeve, S., Sheaves, B., & Freeman, D. (2015). The role of sleep dysfunction in the occurrence of delusions and hallucinations: A systematic review. *Clinical psychology review*, *42*, 96–115. https://doi.org/10.1016/j.cpr.2015.09.001 | No includible individual studies |
| Reid, J. E., Laws, K. R., Drummond, L., Vismara, M., Grancini, B., Mpavaenda, D., & Fineberg, N. A. (2021). Cognitive behavioural therapy with exposure and response prevention in the treatment of obsessive-compulsive disorder: A systematic review and meta-analysis of randomised controlled trials. Comprehensive psychiatry, 106, 152223. https://doi.org/https://dx.doi.org/10.1016/j.comppsych.2021.152223 | Not reporting outcomes of interest |
| Ren, F., Ma, Y., Zhu, X., Guo, R., Wang, J., & He, L. (2020). Pharmacogenetic association of bi- and triallelic polymorphisms of SLC6A4 with antidepressant response in major depressive disorder. Journal of Affective Disorders, 273, 254-264. https://doi.org/https://dx.doi.org/10.1016/j.jad.2020.04.058 | Study design not eligible |
| Reser, M. P., Slikboer, R., & Rossell, S. L. (2019). A systematic review of factors that influence the efficacy of cognitive remediation therapy in schizophrenia. *The Australian and New Zealand journal of psychiatry*, *53*(7), 624–641. https://doi.org/10.1177/0004867419853348 | No includible individual studies |
| Rethorst, C. D., Wipfli, B. M., & Landers, D. M. (2009). The antidepressive effects of exercise: A meta-analysis of randomized trials. Sports Medicine, 39(6), 491-511. https://doi.org/https://dx.doi.org/10.2165/00007256-200939060-00004 | Not reporting outcomes of interest |
| Reuter, L., Bengel, J., & Scheidt, C. E. (2014). [Non-response to therapy in acute and rehabilitative psychosomatic inpatient care - a systematic review]. Therapie-Non-Response in der psychosomatischen Krankenhausbehandlung und Rehabilitation - Eine systematische Ubersicht., 60(2), 121-145. https://doi.org/https://dx.doi.org/10.13109/zptm.2014.60.2.121 | Only searched one database |
| Rhebergen, D., & Sienaert, P. (2015). [Do psychomotor symptoms predict the treatment response of patients with depressive disorders?]. Voorspellen psychomotore symptomen de behandelrespons bij depressieve stoornissen?, 57(2), 83-88. http://ovidsp.ovid.com/ovidweb.cgi?T=JS&PAGE=reference&D=med12&NEWS=N&AN=25669943 | Only searched one database |
| Ribeiro, S. C., Tandon, R., Grunhaus, L., & Greden, J. F. (1993). The DST as a predictor of outcome in depression: a meta-analysis. *The American journal of psychiatry*, *150*(11), 1618–1629. https://doi.org/10.1176/ajp.150.11.1618 | No includible individual studies |
| Rice, S. M., Goodall, J., Hetrick, S. E., Parker, A. G., Gilbertson, T., Amminger, G. P., Davey, C. G., McGorry, P. D., Gleeson, J., & Alvarez-Jimenez, M. (2014). Online and social networking interventions for the treatment of depression in young people: a systematic review. Journal of Medical Internet Research, 16(9), e206. https://doi.org/https://dx.doi.org/10.2196/jmir.3304 | Not investigating prognostic or predictive factors |
| Robinaugh, D. J., Ward, M. J., Toner, E. R., Brown, M. L., Losiewicz, O. M., Bui, E., & Orr, S. P. (2019). Assessing vulnerability to panic: a systematic review of psychological and physiological responses to biological challenges as prospective predictors of panic attacks and panic disorder. General psychiatry, 32(6), e100140. https://doi.org/https://dx.doi.org/10.1136/gpsych-2019-100140 | Patient population not eligible |
| Romeo, B., Hermand, M., Pétillion, A., Karila, L., & Benyamina, A. (2021). Clinical and biological predictors of psychedelic response in the treatment of psychiatric and addictive disorders: A systematic review. *Journal of psychiatric research*, *137*, 273–282. https://doi.org/10.1016/j.jpsychires.2021.03.002 | No includible individual studies |
| Rommelse, N., van der Kruijs, M., Damhuis, J., Hoek, I., Smeets, S., Antshel, K. M., Hoogeveen, L., & Faraone, S. V. (2016). An evidenced-based perspective on the validity of attention-deficit/hyperactivity disorder in the context of high intelligence. Neuroscience and biobehavioral reviews, 71, 21-47. https://doi.org/https://dx.doi.org/10.1016/j.neubiorev.2016.08.032 | Not investigating prognostic or predictive factors |
| Rosa-Alcazar, A. I., Sanchez-Meca, J., Rosa-Alcazar, A., Iniesta-Sepulveda, M., Olivares-Rodriguez, J., & Parada-Navas, J. L. (2015). Psychological treatment of obsessive-compulsive disorder in children and adolescents: a meta-analysis. The Spanish journal of psychology, 18, E20. https://doi.org/https://dx.doi.org/10.1017/sjp.2015.22 | Patient population not eligible |
| Rosenblat, J. D., Lee, Y., & McIntyre, R. S. (2017). Does Pharmacogenomic Testing Improve Clinical Outcomes for Major Depressive Disorder? A Systematic Review of Clinical Trials and Cost-Effectiveness Studies. The Journal of clinical psychiatry, 78(6), 720-729. https://doi.org/https://dx.doi.org/10.4088/JCP.15r10583 | Not reporting outcomes of interest |
| Rosenfield, D., Smits, J. A. J., Hofmann, S. G., Mataix-Cols, D., de la Cruz, L. F., Andersson, E., Rück, C., Monzani, B., Pérez-Vigil, A., Frumento, P., Davis, M., de Kleine, R. A., Difede, J., Dunlop, B. W., Farrell, L. J., Geller, D., Gerardi, M., Guastella, A. J., Hendriks, G. J., Kushner, M. G., … Otto, M. W. (2019). Changes in Dosing and Dose Timing of D-Cycloserine Explain Its Apparent Declining Efficacy for Augmenting Exposure Therapy for Anxiety-related Disorders: An Individual Participant-data Meta-analysis. *Journal of anxiety disorders*, *68*, 102149. https://doi.org/10.1016/j.janxdis.2019.102149 | No includible individual studies |
| Rossignol, D. A., & Frye, R. E. (2021). The Effectiveness of Cobalamin (B12) Treatment for Autism Spectrum Disorder: A Systematic Review and Meta-Analysis. Journal of personalized medicine, 11(8). https://doi.org/https://dx.doi.org/10.3390/jpm11080784 | Not reporting outcomes of interest |
| Routhieaux, M., Keels, J., & Tillery, E. E. (2018). The use of pharmacogenetic testing in patients with schizophrenia or bipolar disorder: A systematic review. *The mental health clinician*, *8*(6), 294–302. https://doi.org/10.9740/mhc.2018.11.294 | No includible individual studies |
| Rozental, A., Andersson, G., & Carlbring, P. (2019). In the Absence of Effects: An Individual Patient Data Meta-Analysis of Non-response and Its Predictors in Internet-Based Cognitive Behavior Therapy. Frontiers in psychology, 10, 589. https://doi.org/https://dx.doi.org/10.3389/fpsyg.2019.00589 | Not reporting outcomes of interest |
| Rubio, J. M., Schoretsanitis, G., John, M., Tiihonen, J., Taipale, H., Guinart, D., Malhotra, A. K., Correll, C. U., & Kane, J. M. (2020). Psychosis relapse during treatment with long-acting injectable antipsychotics in individuals with schizophrenia-spectrum disorders: an individual participant data meta-analysis. *The lancet. Psychiatry*, *7*(9), 749–761. https://doi.org/10.1016/S2215-0366(20)30264-9 | No includible individual studies |
| Ruhrmann, S., Schultze-Lutter, F., Schmidt, S. J., Kaiser, N., & Klosterkotter, J. (2014). Prediction and prevention of psychosis: current progress and future tasks. European Archives of Psychiatry and Clinical Neuroscience, 264 Suppl 1, S9-16. https://doi.org/https://dx.doi.org/10.1007/s00406-014-0541-5 | Study design not eligible |
| Rybakowski, J. K. (2014). Factors associated with lithium efficacy in bipolar disorder. Harvard Review of Psychiatry, 22(6), 353-357. https://doi.org/https://dx.doi.org/10.1097/HRP.0000000000000006 | Study design not eligible |
| Sabe, M., Zhao, N., Crippa, A., Strauss, G. P., & Kaiser, S. (2021). Intranasal Oxytocin for Negative Symptoms of Schizophrenia: Systematic Review, Meta-Analysis, and Dose-Response Meta-Analysis of Randomized Controlled Trials. The international journal of neuropsychopharmacology, 24(8), 601-614. https://doi.org/https://dx.doi.org/10.1093/ijnp/pyab020 | Not reporting outcomes of interest |
| Sajjadian, M., Lam, R. W., Milev, R., Rotzinger, S., Frey, B. N., Soares, C. N., Parikh, S. V., Foster, J. A., Turecki, G., Muller, D. J., Strother, S. C., Farzan, F., Kennedy, S. H., & Uher, R. (2021). Machine learning in the prediction of depression treatment outcomes: a systematic review and meta-analysis. Psychological medicine, 51(16), 2742-2751. https://doi.org/https://dx.doi.org/10.1017/S0033291721003871 | Study design not eligible |
| Salahuddin, M., Manzar, M. D., Pandi-Perumal, S. R., & Bahammam, A. S. (2021). Emerging Challenges in COVID-19 With Substance Use Disorders. Addictive Disorders and their Treatment, 20(4), 444-453. https://doi.org/https://dx.doi.org/10.1097/ADT.0000000000000266 | Not reporting outcomes of interest |
| Salazar de Pablo, G., Pastor Jordá, C., Vaquerizo-Serrano, J., Moreno, C., Cabras, A., Arango, C., Hernández, P., Veenstra-VanderWeele, J., Simonoff, E., Fusar-Poli, P., Santosh, P., Cortese, S., & Parellada, M. (2022). Systematic Review and Meta-analysis: Efficacy of Pharmacological Interventions for Irritability and Emotional Dysregulation in Autism Spectrum Disorder and Predictors of Response. *Journal of the American Academy of Child and Adolescent Psychiatry*, S0890-8567(22)00198-8. Advance online publication. https://doi.org/10.1016/j.jaac.2022.03.033 | No includible individual studies |
| Samanaite, R., Gillespie, A., Sendt, K.-V., McQueen, G., MacCabe, J. H., & Egerton, A. (2018). Biological Predictors of Clozapine Response: A Systematic Review. Frontiers in Psychiatry, 9, 327. https://doi.org/https://dx.doi.org/10.3389/fpsyt.2018.00327 | Only searched one database |
| Samara, M. T., Leucht, C., Leeflang, M. M., Anghelescu, I.-G., Chung, Y.-C., Crespo-Facorro, B., Elkis, H., Hatta, K., Giegling, I., Kane, J. M., Kayo, M., Lambert, M., Lin, C.-H., Moller, H.-J., Pelayo-Teran, J. M., Riedel, M., Rujescu, D., Schimmelmann, B. G., Serretti, A., Correll, C. U., & Leucht, S. (2015). Early Improvement As a Predictor of Later Response to Antipsychotics in Schizophrenia: A Diagnostic Test Review. The American journal of psychiatry, 172(7), 617-629. http://ovidsp.ovid.com/ovidweb.cgi?T=JS&PAGE=reference&D=med12&NEWS=N&AN=26046338 | Not reporting outcomes of interest |
| Sampson, S. M., & Mrazek, D. A. (2001). Depression in adolescence. Current Opinion in Pediatrics, 13(6), 586-590. https://doi.org/https://dx.doi.org/10.1097/00008480-200112000-00017 | Study design not eligible |
| Sami, M. B., & Nilforooshan, R. (2015). The natural course of anxiety disorders in the elderly: a systematic review of longitudinal trials. *International psychogeriatrics*, *27*(7), 1061–1069. https://doi.org/10.1017/S1041610214001847 | No includible individual studies |
| Sanchez-Moreno, J., Martinez-Aran, A., & Vieta, E. (2017). Treatment of Functional Impairment in Patients with Bipolar Disorder. Current Psychiatry Reports, 19(1), 3. https://doi.org/https://dx.doi.org/10.1007/s11920-017-0752-3 | Not investigating prognostic or predictive factors |
| Sanchez Meca, J., Gomez Conesa, A., & Mendez Carrillo, F. X. (2003). Psychological treatment of obsessive-compulsive disorder in Europe: A meta-analytic study. Psicologia Conductual, 11(2), 213-237. http://ovidsp.ovid.com/ovidweb.cgi?T=JS&PAGE=reference&D=emed8&NEWS=N&AN=37409180 | Not reporting outcomes of interest |
| Sanger, N., Bhatt, M., Singhal, N., Panesar, B., D'Elia, A., Trottier, M., Shahid, H., Hillmer, A., Baptist-Mohseni, N., Roczyki, V., Soni, D., Brush, M., Lovell, E., Sanger, S., Samaan, M. C., de Souza, R. J., Thabane, L., & Samaan, Z. (2020). Treatment Outcomes in Patients With Opioid Use Disorder Who Were First Introduced to Opioids by Prescription: A Systematic Review and Meta-Analysis. Frontiers in Psychiatry, 11, 812. https://doi.org/https://dx.doi.org/10.3389/fpsyt.2020.00812 | Not reporting outcomes of interest |
| Santesteban-Echarri, O., Paino, M., Rice, S., Gonzalez-Blanch, C., McGorry, P., Gleeson, J., & Alvarez-Jimenez, M. (2017). Predictors of functional recovery in first-episode psychosis: A systematic review and meta-analysis of longitudinal studies. Clinical Psychology Review, 58, 59-75. https://doi.org/https://dx.doi.org/10.1016/j.cpr.2017.09.007 | Univariable models or correlational studies |
| Santiago, P. N., Ursano, R. J., Gray, C. L., Pynoos, R. S., Spiegel, D., Lewis-Fernandez, R., Friedman, M. J., & Fullerton, C. S. (2013). A Systematic Review of PTSD Prevalence and Trajectories in DSM-5 Defined Trauma Exposed Populations: Intentional and Non-Intentional Traumatic Events. PLoS ONE, 8(4), e59236. https://doi.org/https://dx.doi.org/10.1371/journal.pone.0059236 | Not reporting outcomes of interest |
| Santos, V. A., Carvalho, D. D., Van Ameringen, M., Nardi, A. E., & Freire, R. C. (2019). Neuroimaging findings as predictors of treatment outcome of psychotherapy in anxiety disorders. *Progress in neuro-psychopharmacology & biological psychiatry*, *91*, 60–71. https://doi.org/10.1016/j.pnpbp.2018.04.001 | No includible individual studies |
| Saunders, E. F. H., Ramsden, C. E., Sherazy, M. S., Gelenberg, A. J., Davis, J. M., & Rapoport, S. I. (2016). Omega-3 and Omega-6 Polyunsaturated Fatty Acids in Bipolar Disorder: A Review of Biomarker and Treatment Studies. The Journal of clinical psychiatry, 77(10), e1301-e1308. https://doi.org/https://dx.doi.org/10.4088/JCP.15r09925 | Study design not eligible |
| Sayyah, M., & Rahim, F. (2018). Pharmacotherapy for treatment-respondent vs. refractory obsessive-compulsive disorder in children and adults: Strategies, meta-analyses and clinical guidelines. Archives of Psychiatry and Psychotherapy, 20(3), 42-54. https://doi.org/https://dx.doi.org/10.12740/APP/92745 | Study design not eligible |
| Scaini, S., Belotti, R., Ogliari, A., & Battaglia, M. (2016). A comprehensive meta-analysis of cognitive-behavioral interventions for social anxiety disorder in children and adolescents. Journal of Anxiety Disorders, 42, 105-112. https://doi.org/https://dx.doi.org/10.1016/j.janxdis.2016.05.008 | Univariable models or correlational studies |
| Scherrer, B., Guiraud, J., Addolorato, G., Aubin, H. J., de Bejczy, A., Benyamina, A., van den Brink, W., Caputo, F., Dematteis, M., Goudriaan, A. E., Gual, A., Kiefer, F., Leggio, L., Lesch, O. M., Maremmani, I., Nutt, D. J., Paille, F., Perney, P., Poulnais, R., Raffaillac, Q., … Spanagel, R. (2021). Baseline severity and the prediction of placebo response in clinical trials for alcohol dependence: A meta-regression analysis to develop an enrichment strategy. *Alcoholism, clinical and experimental research*, *45*(9), 1722–1734. https://doi.org/10.1111/acer.14670 | No includible individual studies |
| Schiavone, S., & Trabace, L. (2017). Inflammation, stress response, and redox dysregulation biomarkers: Clinical outcomes and pharmacological implications for psychosis. Frontiers in Psychiatry, 8(OCT), 203. https://doi.org/https://dx.doi.org/10.3389/fpsyt.2017.00203 | Study design not eligible |
| Schiele, M. A., Reif, A., Lin, J., Alpers, G. W., Andersson, E., Andersson, G., Arolt, V., Bergstrom, J., Carlbring, P., Eley, T. C., Esquivel, G., Furmark, T., Gerlach, A. L., Hamm, A., Helbig-Lang, S., Hudson, J. L., Lang, T., Lester, K. J., Lindefors, N., Lonsdorf, T. B., Pauli, P., Richter, J., Rief, W., Roberts, S., Ruck, C., Schruers, K. R. J., Thiel, C., Wittchen, H.-U., Domschke, K., Weber, H., & Lueken, U. (2021). Therapygenetic effects of 5-HTTLPR on cognitive-behavioral therapy in anxiety disorders: A meta-analysis. European neuropsychopharmacology : the journal of the European College of Neuropsychopharmacology, 44, 105-120. https://doi.org/https://dx.doi.org/10.1016/j.euroneuro.2021.01.004 | Not reporting outcomes of interest |
| Schlaepfer, T. E., Agren, H., Monteleone, P., Gasto, C., Pitchot, W., Rouillon, F., Nutt, D. J., & Kasper, S. (2012). The hidden third: Improving outcome in treatment-resistant depression. Journal of Psychopharmacology, 26(5), 587-602. https://doi.org/https://dx.doi.org/10.1177/0269881111431748 | Not reporting outcomes of interest |
| Schoeler, T., Monk, A., Sami, M. B., Klamerus, E., Foglia, E., Brown, R., Camuri, G., Altamura, A. C., Murray, R., & Bhattacharyya, S. (2016). Continued versus discontinued cannabis use in patients with psychosis: a systematic review and meta-analysis. The lancet. Psychiatry, 3(3), 215-225. https://doi.org/https://dx.doi.org/10.1016/S2215-0366(15)00363-6 | Only searched one database |
| Schoemaker, J. H., Kilian, S., Emsley, R., & Vingerhoets, A. J. J. M. (2018). Factors associated with placebo response in depression trials: A systematic review of published meta-analyses (1990-2017). Neurology Psychiatry and Brain Research, 30, 12-21. https://doi.org/https://dx.doi.org/10.1016/j.npbr.2018.04.002 | Not reporting outcomes of interest |
| Schrank, B., Stanghellini, G., & Slade, M. (2008). Hope in psychiatry: A review of the literature. Acta Psychiatrica Scandinavica, 118(6), 421-433. https://doi.org/https://dx.doi.org/10.1111/j.1600-0447.2008.01271.x | Not reporting outcomes of interest |
| Schubert, K. O., Thalamuthu, A., Amare, A. T., Frank, J., Streit, F., Adl, M., Akula, N., Akiyama, K., Ardau, R., Arias, B., Aubry, J.-M., Backlund, L., Bhattacharjee, A. K., Bellivier, F., Benabarre, A., Bengesser, S., Biernacka, J. M., Birner, A., Marie-Claire, C., Cearns, M., Cervantes, P., Chen, H.-C., Chillotti, C., Cichon, S., Clark, S. R., Cruceanu, C., Czerski, P. M., Dalkner, N., Dayer, A., Degenhardt, F., Del Zompo, M., DePaulo, J. R., Etain, B., Falkai, P., Forstner, A. J., Frisen, L., Frye, M. A., Fullerton, J. M., Gard, S., Garnham, J. S., Goes, F. S., Grigoroiu-Serbanescu, M., Grof, P., Hashimoto, R., Hauser, J., Heilbronner, U., Herms, S., Hoffmann, P., Hou, L., Hsu, Y.-H., Jamain, S., Jimenez, E., Kahn, J.-P., Kassem, L., Kuo, P.-H., Kato, T., Kelsoe, J., Kittel-Schneider, S., Ferensztajn-Rochowiak, E., Konig, B., Kusumi, I., Laje, G., Landen, M., Lavebratt, C., Leboyer, M., Leckband, S. G., Maj, M., Major Depressive Disorder Working Group of the Psychiatric Genomics, C., Manchia, M., Martinsson, L., McCarthy, M. J., McElroy, S., Colom, F., Mitjans, M., Mondimore, F. M., Monteleone, P., Nievergelt, C. M., Nothen, M. M., Novak, T., O'Donovan, C., Ozaki, N., Osby, U., Papiol, S., Pfennig, A., Pisanu, C., Potash, J. B., Reif, A., Reininghaus, E., Rouleau, G. A., Rybakowski, J. K., Schalling, M., Schofield, P. R., Schweizer, B. W., Severino, G., Shekhtman, T., Shilling, P. D., Shimoda, K., Simhandl, C., Slaney, C. M., Squassina, A., Stamm, T., Stopkova, P., Tekola-Ayele, F., Tortorella, A., Turecki, G., Veeh, J., Vieta, E., Witt, S. H., Roberts, G., Zandi, P. P., Alda, M., Bauer, M., McMahon, F. J., Mitchell, P. B., Schulze, T. G., Rietschel, M., & Baune, B. T. (2021). Combining schizophrenia and depression polygenic risk scores improves the genetic prediction of lithium response in bipolar disorder patients. Translational psychiatry, 11(1), 606. https://doi.org/https://dx.doi.org/10.1038/s41398-021-01702-2 | Study design not eligible |
| Schuch, F. B., Dunn, A. L., Kanitz, A. C., Delevatti, R. S., & Fleck, M. P. (2016). Moderators of response in exercise treatment for depression: A systematic review. *Journal of affective disorders*, *195*, 40–49. https://doi.org/10.1016/j.jad.2016.01.014 | No includible individual studies |
| Schumacher, S., Niemeyer, H., Engel, S., Cwik, J. C., & Knaevelsrud, C. (2018). Psychotherapeutic treatment and HPA axis regulation in posttraumatic stress disorder: A systematic review and meta-analysis. *Psychoneuroendocrinology*, *98*, 186–201. https://doi.org/10.1016/j.psyneuen.2018.08.006 | No includible individual studies |
| Scott, J. C., Lynch, K. G., Cenkner, D. P., Kehle-Forbes, S. M., Polusny, M. A., Gur, R. C., Chen, S., Foa, E. B., & Oslin, D. W. (2021). Neurocognitive Predictors of Treatment Outcomes in Psychotherapy for Comorbid PTSD and Substance Use Disorders. Journal of Consulting and Clinical Psychology, 89(11), 937-946. https://doi.org/https://dx.doi.org/10.1037/ccp0000693 | Study design not eligible |
| Seccomandi, B., Tsapekos, D., Newbery, K., Wykes, T., & Cella, M. (2019). A systematic review of moderators of cognitive remediation response for people with schizophrenia. *Schizophrenia research. Cognition*, *19*, 100160. https://doi.org/10.1016/j.scog.2019.100160 | No includible individual studies |
| Serre, F., Fatseas, M., Swendsen, J., & Auriacombe, M. (2015). Ecological momentary assessment in the investigation of craving and substance use in daily life: a systematic review. Drug and alcohol dependence, 148, 1-20. https://doi.org/https://dx.doi.org/10.1016/j.drugalcdep.2014.12.024 | Not investigating prognostic or predictive factors |
| Serretti, A., Gibiino, S., & Drago, A. (2011). Specificity profile of paroxetine in major depressive disorder: meta-regression of double-blind, randomized clinical trials. Journal of Affective Disorders, 132(1-2), 14-25. https://doi.org/https://dx.doi.org/10.1016/j.jad.2010.08.018 | Not reporting outcomes of interest |
| Shah, A. S., Young, J., & Vieira, K. (2014). Long-term suboxone treatment and its benefit on long-term remission for opiate dependence. African Journal of Psychiatry (South Africa), 17(6), 1000174. https://doi.org/https://dx.doi.org/10.4172/Psychiatry.1000174 | Not investigating prognostic or predictive factors |
| Sharma, E., Thennarasu, K., & Reddy, Y. C. J. (2014). Long-term outcome of obsessive-compulsive disorder in adults: a meta-analysis. The Journal of clinical psychiatry, 75(9), 1019-1027. https://doi.org/https://dx.doi.org/10.4088/JCP.13r08849 | Only searched one database |
| Sharma, V., Khan, M., Baczynski, C., & Boate, I. (2020). Predictors of response to antidepressants in women with postpartum depression: a systematic review. *Archives of women's mental health*, *23*(5), 613–623. https://doi.org/10.1007/s00737-020-01044-w | No includible individual studies |
| Sheehan, D. V., Nakagome, K., Asami, Y., Pappadopulos, E. A., & Boucher, M. (2017). Restoring function in major depressive disorder: A systematic review. Journal of Affective Disorders, 215, 299-313. https://doi.org/https://dx.doi.org/10.1016/j.jad.2017.02.029 | Only searched one database |
| Sheehan, R., & Hassiotis, A. (2017). Reduction or discontinuation of antipsychotics for challenging behaviour in adults with intellectual disability: a systematic review. The lancet. Psychiatry, 4(3), 238-256. https://doi.org/https://dx.doi.org/10.1016/S2215-0366(16)30191-2 | Not reporting outcomes of interest |
| Shinohara, K., Tanaka, S., Imai, H., Noma, H., Maruo, K., Cipriani, A., Yamawaki, S., & Furukawa, T. A. (2019). Development and validation of a prediction model for the probability of responding to placebo in antidepressant trials: a pooled analysis of individual patient data. Evidence-based mental health, 22(1), 10-16. https://doi.org/https://dx.doi.org/10.1136/ebmental-2018-300073 | Not reporting outcomes of interest |
| Shiozawa, P., Fregni, F., Bensenor, I. M., Lotufo, P. A., Berlim, M. T., Daskalakis, J. Z., Cordeiro, Q., & Brunoni, A. R. (2014). Transcranial direct current stimulation for major depression: an updated systematic review and meta-analysis. The international journal of neuropsychopharmacology, 17(9), 1443-1452. https://doi.org/https://dx.doi.org/10.1017/S1461145714000418 | Not investigating prognostic or predictive factors |
| Sievers, S. B., Trembath, D., & Westerveld, M. (2018). A systematic review of predictors, moderators, and mediators of augmentative and alternative communication (AAC) outcomes for children with autism spectrum disorder. Augmentative and alternative communication (Baltimore, Md. : 1985), 34(3), 219-229. https://doi.org/https://dx.doi.org/10.1080/07434618.2018.1462849 | Not reporting outcomes of interest |
| Silverman, W. K., Pina, A. A., & Viswesvaran, C. (2008). Evidence-based psychosocial treatments for phobic and anxiety disorders in children and adolescents. Journal of clinical child and adolescent psychology : the official journal for the Society of Clinical Child and Adolescent Psychology, American Psychological Association, Division 53, 37(1), 105-130. https://doi.org/https://dx.doi.org/10.1080/15374410701817907 | Only searched one database |
| Sim, K., Lau, W. K., Sim, J., Sum, M. Y., & Baldessarini, R. J. (2015). Prevention of Relapse and Recurrence in Adults with Major Depressive Disorder: Systematic Review and Meta-Analyses of Controlled Trials. The international journal of neuropsychopharmacology, 19(2). https://doi.org/https://dx.doi.org/10.1093/ijnp/pyv076 | Univariable models or correlational studies |
| Simon, A. E., Velthorst, E., Nieman, D. H., Linszen, D., Umbricht, D., & de Haan, L. (2011). Ultra high-risk state for psychosis and non-transition: A systematic review. Schizophrenia Research, 132(1), 8-17. https://doi.org/https://dx.doi.org/10.1016/j.schres.2011.07.002 | Not reporting outcomes of interest |
| Simon, L., Blay, M., Galvao, F., & Brunelin, J. (2021). Using EEG to Predict Clinical Response to Electroconvulsive Therapy in Patients With Major Depression: A Comprehensive Review. *Frontiers in psychiatry*, *12*, 643710. https://doi.org/10.3389/fpsyt.2021.643710 | No includible individual studies |
| Sinclair, L. I., Christmas, D. M., Hood, S. D., Potokar, J. P., Robertson, A., Isaac, A., Srivastava, S., Nutt, D. J., & Davies, S. J. C. (2009). Antidepressant-induced jitteriness/anxiety syndrome: systematic review. The British journal of psychiatry : the journal of mental science, 194(6), 483-490. https://doi.org/https://dx.doi.org/10.1192/bjp.bp.107.048371 | Not reporting outcomes of interest |
| Siragusa, M. A., Remenieras, J. P., Bouakaz, A., Escoffre, J. M., Patat, F., Dujardin, P. A., Brizard, B., Belzung, C., Camus, V., El-Hage, W., & Desmidt, T. (2020). A systematic review of ultrasound imaging and therapy in mental disorders. Progress in Neuro-Psychopharmacology and Biological Psychiatry, 101, 109919. https://doi.org/https://dx.doi.org/10.1016/j.pnpbp.2020.109919 | Not investigating prognostic or predictive factors |
| Siskind, D., McCartney, L., Goldschlager, R., & Kisely, S. (2016). Clozapine v. first- and second-generation antipsychotics in treatment-refractory schizophrenia: systematic review and meta-analysis. The British journal of psychiatry : the journal of mental science, 209(5), 385-392. http://ovidsp.ovid.com/ovidweb.cgi?T=JS&PAGE=reference&D=med13&NEWS=N&AN=27388573 | Univariable models or correlational studies |
| Skorobogatov, K., De Picker, L., Verkerk, R., Coppens, V., Leboyer, M., Muller, N., & Morrens, M. (2021). Brain Versus Blood: A Systematic Review on the Concordance Between Peripheral and Central Kynurenine Pathway Measures in Psychiatric Disorders. Frontiers in immunology, 12, 716980. https://doi.org/https://dx.doi.org/10.3389/fimmu.2021.716980 | No includible individual studies |
| Sliedrecht, W., Roozen, H. G., Witkiewitz, K., De Waart, R., & Dom, G. (2021). The Association between Impulsivity and Relapse in Patients with Alcohol Use Disorder: A Literature Review. Alcohol and Alcoholism, 56(6), 637-650. https://doi.org/https://dx.doi.org/10.1093/alcalc/agaa132 | Univariable models or correlational studies |
| Smart, S. E., Kępińska, A. P., Murray, R. M., & MacCabe, J. H. (2021). Predictors of treatment resistant schizophrenia: a systematic review of prospective observational studies. *Psychological medicine*, *51*(1), 44–53. https://doi.org/10.1017/S0033291719002083 | No includible individual studies |
| So, S. H., Garety, P. A., Peters, E. R., & Kapur, S. (2010). Do antipsychotics improve reasoning biases? A review. Psychosomatic medicine, 72(7), 681-693. https://doi.org/https://dx.doi.org/10.1097/PSY.0b013e3181e7cca6 | Not investigating prognostic or predictive factors |
| Soares, E., & Pereira, F. C. (2019). Pharmacotherapeutic strategies for methamphetamine use disorder: mind the subgroups. Expert Opinion on Pharmacotherapy, 20(18), 2273-2293. https://doi.org/https://dx.doi.org/10.1080/14656566.2019.1681970 | Only searched one database |
| Solmi, M., Fornaro, M., Toyoshima, K., Carvalho, A. F., Kohler, C. A., Veronese, N., Stubbs, B., de Bartolomeis, A., & Correll, C. U. (2019). Systematic review and exploratory meta-analysis of the efficacy, safety, and biological effects of psychostimulants and atomoxetine in patients with schizophrenia or schizoaffective disorder. CNS Spectrums, 24(5), 479-495. https://doi.org/https://dx.doi.org/10.1017/S1092852918001050 | Univariable models or correlational studies |
| Solomon, H. V., Cates, K. W., & Li, K. J. (2019). Does obtaining CYP2D6 and CYP2C19 pharmacogenetic testing predict antidepressant response or adverse drug reactions? Psychiatry Research, 271, 604-613. https://doi.org/https://dx.doi.org/10.1016/j.psychres.2018.12.053 | Only searched one database |
| Sparshatt, A., Taylor, D., Patel, M. X., & Kapur, S. (2009). Amisulpride - Dose, plasma concentration, occupancy and response: Implications for therapeutic drug monitoring. Acta Psychiatrica Scandinavica, 120(6), 416-428. https://doi.org/https://dx.doi.org/10.1111/j.1600-0447.2009.01429.x | Study design not eligible |
| Sparshatt, A., Taylor, D., Patel, M. X., & Kapur, S. (2010). A systematic review of aripiprazole--dose, plasma concentration, receptor occupancy, and response: implications for therapeutic drug monitoring. The Journal of clinical psychiatry, 71(11), 1447-1456. https://doi.org/https://dx.doi.org/10.4088/JCP.09r05060gre | Not reporting outcomes of interest |
| Spielmans, G. I., Berman, M. I., & Usitalo, A. N. (2011). Psychotherapy versus second-generation antidepressants in the treatment of depression: a meta-analysis. The Journal of nervous and mental disease, 199(3), 142-149. https://doi.org/https://dx.doi.org/10.1097/NMD.0b013e31820caefb | Univariable models or correlational studies |
| Sriretnakumar, V., Huang, E., & Müller, D. J. (2015). Pharmacogenetics of clozapine treatment response and side-effects in schizophrenia: an update. *Expert opinion on drug metabolism & toxicology*, *11*(11), 1709–1731. https://doi.org/10.1517/17425255.2015.1075003 | No includible individual studies |
| Starkstein, S. E., & Hayhow, B. D. (2019). Treatment of Post-Stroke Depression. Current Treatment Options in Neurology, 21(7), 31. https://doi.org/https://dx.doi.org/10.1007/s11940-019-0570-5 | Study design not eligible |
| Stassen, H. H., Angst, J., & Delini-Stula, A. (1994). Severity at baseline and onset of improvement in depression. Meta-analysis of imipramine and moclobemide versus placebo. European Psychiatry, 9(3), 129-136. http://ovidsp.ovid.com/ovidweb.cgi?T=JS&PAGE=reference&D=emed5&NEWS=N&AN=24158209 | Study design not eligible |
| Stein, D. J., Seedat, S., van der Linden, G. J., & Zungu-Dirwayi, N. (2000). Selective serotonin reuptake inhibitors in the treatment of post-traumatic stress disorder: a meta-analysis of randomized controlled trials. International clinical psychopharmacology, 15 Suppl 2, S31-39. http://ovidsp.ovid.com/ovidweb.cgi?T=JS&PAGE=reference&D=med4&NEWS=N&AN=11110017 | Not investigating prognostic or predictive factors |
| Stein, D. J., Zungu-Dirwayi, N., van Der Linden, G. J., & Seedat, S. (2000). Pharmacotherapy for posttraumatic stress disorder. The Cochrane database of systematic reviews(4), CD002795. http://ovidsp.ovid.com/ovidweb.cgi?T=JS&PAGE=reference&D=med4&NEWS=N&AN=11034765 | Not investigating prognostic or predictive factors |
| Steinert, C., Hofmann, M., Kruse, J., & Leichsenring, F. (2014). The prospective long-term course of adult depression in general practice and the community. A systematic literature review. Journal of Affective Disorders, 152-154, 65-75. https://doi.org/https://dx.doi.org/10.1016/j.jad.2013.10.017 | Study design not eligible |
| Steinert, C., Hofmann, M., Leichsenring, F., & Kruse, J. (2015). The course of PTSD in naturalistic long-term studies: high variability of outcomes. A systematic review. Nordic journal of psychiatry, 69(7), 483-496. https://doi.org/https://dx.doi.org/10.3109/08039488.2015.1005023 | Not reporting outcomes of interest |
| Steinert, C., Hofmann, M., Leichsenring, F., & Kruse, J. (2013). What do we know today about the prospective long-term course of social anxiety disorder? A systematic literature review. *Journal of anxiety disorders*, *27*(7), 692–702. https://doi.org/10.1016/j.janxdis.2013.08.002 | No includible individual studies |
| Steiro, A., Dalsbo, T. K., Smedslund, G., Hammerstrom, K. T., & Samdal, K. (2009). Long Term Institutional or Residential Treatment of Patients with Substance Abuse Compared to Short-Term Outpatient Treatment. http://ovidsp.ovid.com/ovidweb.cgi?T=JS&PAGE=reference&D=medp&NEWS=N&AN=29320091 | Not investigating prognostic or predictive factors |
| Stewart, S. E., Geller, D. A., Jenike, M., Pauls, D., Shaw, D., Mullin, B., & Faraone, S. V. (2004). Long-term outcome of pediatric obsessive-compulsive disorder: a meta-analysis and qualitative review of the literature. Acta Psychiatrica Scandinavica, 110(1), 4-13. http://ovidsp.ovid.com/ovidweb.cgi?T=JS&PAGE=reference&D=med5&NEWS=N&AN=15180774 | Not investigating prognostic or predictive factors |
| Stimpfl, J. N., Mills, J. A., & Strawn, J. R. (2021). Pharmacologic predictors of benzodiazepine response trajectory in anxiety disorders: a Bayesian hierarchical modeling meta-analysis. CNS Spectrums, 1-8. https://doi.org/https://dx.doi.org/10.1017/S1092852921000870 | No includible individual studies |
| Storosum, J. G., Wohlfarth, T., Schene, A., Elferink, A., van Zwieten, B. J., & van den Brink, W. (2007). Magnitude of effect of lithium in short-term efficacy studies of moderate to severe manic episode. Bipolar Disorders, 9(8), 793-798. https://doi.org/https://dx.doi.org/10.1111/j.1399-5618.2007.00445.x | Not investigating prognostic or predictive factors |
| Straud, C. L., Siev, J., Messer, S., & Zalta, A. K. (2019). Examining military population and trauma type as moderators of treatment outcome for first-line psychotherapies for PTSD: A meta-analysis. Journal of Anxiety Disorders, 67, 102133. https://doi.org/https://dx.doi.org/10.1016/j.janxdis.2019.102133 | Patient population not eligible |
| Strauss, C., Cavanagh, K., Oliver, A., & Pettman, D. (2014). Mindfulness-based interventions for people diagnosed with a current episode of an anxiety or depressive disorder: a meta-analysis of randomised controlled trials. PLoS ONE, 9(4), e96110. https://doi.org/https://dx.doi.org/10.1371/journal.pone.0096110 | Univariable models or correlational studies |
| Stromme, R., Borstad, K. H., Ro, A. E., Erevik, E. K., Sagoe, D., Chegeni, R., Aune Mentzoni, R., Kaur, P., & Pallesen, S. (2021). The Relationship Between Gambling Problems and the Five-Factor Model of Personality: A Systematic Review and Meta-Analysis. Frontiers in Psychiatry, 12, 740235. https://doi.org/https://dx.doi.org/10.3389/fpsyt.2021.740235 | Not reporting outcomes of interest |
| Stroud, C. B., Davila, J., & Moyer, A. (2008). The relationship between stress and depression in first onsets versus recurrences: a meta-analytic review. Journal of abnormal psychology, 117(1), 206-213. https://doi.org/https://dx.doi.org/10.1037/0021-843X.117.1.206 | Not reporting outcomes of interest |
| Szegedi, A., Jansen, W. T., van Willigenburg, A. P. P., van der Meulen, E., Stassen, H. H., & Thase, M. E. (2009). Early improvement in the first 2 weeks as a predictor of treatment outcome in patients with major depressive disorder: a meta-analysis including 6562 patients. The Journal of clinical psychiatry, 70(3), 344-353. http://ovidsp.ovid.com/ovidweb.cgi?T=JS&PAGE=reference&D=med7&NEWS=N&AN=19254516 | Study design not eligible |
| Tani, H., Suzuki, T., Wolfgang Fleischhacker, W., Tomita, M., Mimura, M., & Uchida, H. (2018). Clinical Characteristics of Patients With Schizophrenia Who Successfully Discontinued Antipsychotics: A Literature Review. Journal of Clinical Psychopharmacology, 38(6), 582-589. https://doi.org/https://dx.doi.org/10.1097/JCP.0000000000000959 | Only searched one database |
| Tani, H., Takasu, S., Uchida, H., Suzuki, T., Mimura, M., & Takeuchi, H. (2020). Factors associated with successful antipsychotic dose reduction in schizophrenia: a systematic review of prospective clinical trials and meta-analysis of randomized controlled trials. Neuropsychopharmacology : official publication of the American College of Neuropsychopharmacology, 45(5), 887-901. https://doi.org/https://dx.doi.org/10.1038/s41386-019-0573-7 | Not reporting outcomes of interest |
| Tansey, K. E., Guipponi, M., Perroud, N., Bondolfi, G., Domenici, E., Evans, D., Hall, S. K., Hauser, J., Henigsberg, N., Hu, X., Jerman, B., Maier, W., Mors, O., O'Donovan, M., Peters, T. J., Placentino, A., Rietschel, M., Souery, D., Aitchison, K. J., Craig, I., Farmer, A., Wendland, J. R., Malafosse, A., Holmans, P., Lewis, G., Lewis, C. M., Stensbol, T. B., Kapur, S., McGuffin, P., & Uher, R. (2012). Genetic predictors of response to serotonergic and noradrenergic antidepressants in major depressive disorder: a genome-wide analysis of individual-level data and a meta-analysis. PLoS medicine, 9(10), e1001326. https://doi.org/https://dx.doi.org/10.1371/journal.pmed.1001326 | Study design not eligible |
| Taylor, M. J. (2007). Rapid onset of true antidepressant action. Current Psychiatry Reports, 9(6), 475-479. https://doi.org/https://dx.doi.org/10.1007/s11920-007-0064-0 | Study design not eligible |
| Taylor, M. J., Sen, S., & Bhagwagar, Z. (2010). Antidepressant response and the serotonin transporter gene-linked polymorphic region. Biological psychiatry, 68(6), 536-543. https://doi.org/https://dx.doi.org/10.1016/j.biopsych.2010.04.034 | Univariable models or correlational studies |
| Thase, M., Asami, Y., Wajsbrot, D., Dorries, K., Boucher, M., & Pappadopulos, E. (2017). A meta-analysis of the efficacy of venlafaxine extended release 75-225 mg/day for the treatment of major depressive disorder. Current medical research and opinion, 33(2), 317-326. https://doi.org/https://dx.doi.org/10.1080/03007995.2016.1255185 | Not investigating prognostic or predictive factors |
| Thomas, E. C., Despeaux, K. E., Drapalski, A. L., & Bennett, M. (2018). Person-Oriented Recovery of Individuals With Serious Mental Illnesses: A Review and Meta-Analysis of Longitudinal Findings. *Psychiatric services (Washington, D.C.)*, *69*(3), 259–267. https://doi.org/10.1176/appi.ps.201700058 | No includible individual studies |
| Thompson-Brenner, H., Franko, D. L., Thompson, D. R., Grilo, C. M., Boisseau, C. L., Roehrig, J. P., Richards, L. K., Bryson, S. W., Bulik, C. M., Crow, S. J., Devlin, M. J., Gorin, A. A., Kristeller, J. L., Masheb, R., Mitchell, J. E., Peterson, C. B., Safer, D. L., Striegel, R. H., Wilfley, D. E., & Wilson, G. T. (2013). Race/ethnicity, education, and treatment parameters as moderators and predictors of outcome in binge eating disorder. Journal of Consulting and Clinical Psychology, 81(4), 710-721. https://doi.org/https://dx.doi.org/10.1037/a0032946 | Study design not eligible |
| Thompson-Hollands, J., Edson, A., Tompson, M. C., & Comer, J. S. (2014). Family involvement in the psychological treatment of obsessive-compulsive disorder: a meta-analysis. Journal of family psychology : JFP : journal of the Division of Family Psychology of the American Psychological Association (Division 43), 28(3), 287-298. https://doi.org/https://dx.doi.org/10.1037/a0036709 | Not investigating prognostic or predictive factors |
| Tighe, S. K., Mahon, P. B., & Potash, J. B. (2011). Predictors of lithium response in bipolar disorder. Therapeutic Advances in Chronic Disease, 2(3), 209-226. https://doi.org/https://dx.doi.org/10.1177/2040622311399173 | Study design not eligible |
| Tolin, D. F., Frost, R. O., Steketee, G., & Muroff, J. (2015). Cognitive behavioral therapy for hoarding disorder: a meta-analysis. Depression and Anxiety, 32(3), 158-166. https://doi.org/https://dx.doi.org/10.1002/da.22327 | Univariable models or correlational studies |
| Tsang, H. W. H., Leung, A. Y., Chung, R. C. K., Bell, M., & Cheung, W.-M. (2010). Review on vocational predictors: a systematic review of predictors of vocational outcomes among individuals with schizophrenia: an update since 1998. The Australian and New Zealand journal of psychiatry, 44(6), 495-504. https://doi.org/https://dx.doi.org/10.3109/00048671003785716 | Not reporting outcomes of interest |
| Tse, S., Chan, S., Ng, K. L., & Yatham, L. N. (2014). Meta-analysis of predictors of favorable employment outcomes among individuals with bipolar disorder. Bipolar Disorders, 16(3), 217-229. https://doi.org/https://dx.doi.org/10.1111/bdi.12148 | Not reporting outcomes of interest |
| Tursi, M. F. d. S., Baes, C. v. W., Camacho, F. R. d. B., Tofoli, S. M. d. C., & Juruena, M. F. (2013). Effectiveness of psychoeducation for depression: a systematic review. The Australian and New Zealand journal of psychiatry, 47(11), 1019-1031. https://doi.org/https://dx.doi.org/10.1177/0004867413491154 | Not investigating prognostic or predictive factors |
| Twamley, E. W., Jeste, D. V., & Lehman, A. F. (2003). Vocational rehabilitation in schizophrenia and other psychotic disorders: a literature review and meta-analysis of randomized controlled trials. The Journal of nervous and mental disease, 191(8), 515-523. http://ovidsp.ovid.com/ovidweb.cgi?T=JS&PAGE=reference&D=med5&NEWS=N&AN=12972854 | Not reporting outcomes of interest |
| Uljarević, M., Billingham, W., Cooper, M. N., Condron, P., & Hardan, A. Y. (2022). Examining Effectiveness and Predictors of Treatment Response of Pivotal Response Treatment in Autism: An Umbrella Review and a Meta-Analysis. *Frontiers in psychiatry*, *12*, 766150. https://doi.org/10.3389/fpsyt.2021.766150 | No includible individual studies |
| Vafaie, N., & Kober, H. (2022). Association of Drug Cues and Craving With Drug Use and Relapse: A Systematic Review and Meta-analysis. JAMA psychiatry, 79(7), 641-650. https://doi.org/https://dx.doi.org/10.1001/jamapsychiatry.2022.1240 | Univariable models or correlational studies |
| Valiente, C., Espinosa, R., Trucharte, A., Nieto, J., & Martinez-Prado, L. (2019). The challenge of well-being and quality of life: A meta-analysis of psychological interventions in schizophrenia. Schizophrenia Research, 208, 16-24. https://doi.org/https://dx.doi.org/10.1016/j.schres.2019.01.040 | Not investigating prognostic or predictive factors |
| Vall, E., & Wade, T. D. (2015). Predictors of treatment outcome in individuals with eating disorders: A systematic review and meta-analysis. The International journal of eating disorders, 48(7), 946-971. https://doi.org/https://dx.doi.org/10.1002/eat.22411 | Univariable models or correlational studies |
| van Amsterdam, J., & van den Brink, W. (2022). Smoking As an Outcome Moderator In the Treatment of Alcohol Use Disorders. Alcohol and alcoholism (Oxford, Oxfordshire). https://doi.org/https://dx.doi.org/10.1093/alcalc/agac027 | Only searched one database |
| van Weel-Baumgarten, E. M., Schers, H. J., van den Bosch, W. J., van den Hoogen, H. J., & Zitman, F. G. (2000). Long-term follow-up of depression among patients in the community and in family practice settings. A systematic review. The Journal of family practice, 49(12), 1113-1120. http://ovidsp.ovid.com/ovidweb.cgi?T=JS&PAGE=reference&D=med4&NEWS=N&AN=11132061 | Not investigating prognostic or predictive factors |
| Vandevelde, A., Metivier, L., & Dollfus, S. (2021). Impact cerebral structurel et fonctionnel de la Clozapine chez les patients souffrant de schizophrenie : revue systematique des etudes longitudinales en neuroimagerie. Canadian Journal of Psychiatry, 66(8), 683-700. https://doi.org/https://dx.doi.org/10.1177/0706743720966459 | Not reporting outcomes of interest |
| Vanderplasschen, W., Colpaert, K., Autrique, M., Rapp, R. C., Pearce, S., Broekaert, E., & Vandevelde, S. (2013). Therapeutic communities for addictions: a review of their effectiveness from a recovery-oriented perspective. *TheScientificWorldJournal*, *2013*, 427817. https://doi.org/10.1155/2013/427817 | No includible individual studies |
| Vanderplasschen, W., Rapp, R. C., De Maeyer, J., & Van Den Noortgate, W. (2019). A Meta-Analysis of the Efficacy of Case Management for Substance Use Disorders: A Recovery Perspective. *Frontiers in psychiatry*, *10*, 186. https://doi.org/10.3389/fpsyt.2019.00186 | No includible individual studies |
| Varigonda, A. L., Jakubovski, E., Taylor, M. J., Freemantle, N., Coughlin, C., & Bloch, M. H. (2015). Systematic Review and Meta-Analysis: Early Treatment Responses of Selective Serotonin Reuptake Inhibitors in Pediatric Major Depressive Disorder. Journal of the American Academy of Child and Adolescent Psychiatry, 54(7), 557-564. https://doi.org/https://dx.doi.org/10.1016/j.jaac.2015.05.004 | Not investigating prognostic or predictive factors |
| Veale, D., Naismith, I., Miles, S., Gledhill, L. J., Stewart, G., & Hodsoll, J. (2016). Outcomes for residential or inpatient intensive treatment of obsessive-compulsive disorder: A systematic review and meta-analysis. Journal of Obsessive-Compulsive and Related Disorders, 8, 38-49. https://doi.org/https://dx.doi.org/10.1016/j.jocrd.2015.11.005 | Not reporting outcomes of interest |
| Victorri-Vigneau, C., Spiers, A., Caillet, P., Bruneau, M., Ignace, C., Challet-Bouju, G., & Grall-Bronnec, M. (2018). Opioid Antagonists for Pharmacological Treatment of Gambling Disorder: Are they Relevant? Current neuropharmacology, 16(10), 1418-1432. https://doi.org/https://dx.doi.org/10.2174/1570159X15666170718144058 | Not investigating prognostic or predictive factors |
| Vieta, E., Pacchiarotti, I., Scott, J., Sanchez-Moreno, J., Di Marzo, S., & Colom, F. (2005). Evidence-based research on the efficacy of psychologic interventions in bipolar disorders: a critical review. Current Psychiatry Reports, 7(6), 449-455. http://ovidsp.ovid.com/ovidweb.cgi?T=JS&PAGE=reference&D=med6&NEWS=N&AN=16318823 | Not investigating prognostic or predictive factors |
| Visser, E., Gosens, T., Den Oudsten, B. L., & De Vries, J. (2017). The course, prediction, and treatment of acute and posttraumatic stress in trauma patients: A systematic review. Journal of Trauma and Acute Care Surgery, 82(6), 1158-1183. https://doi.org/https://dx.doi.org/10.1097/TA.0000000000001447 | Univariable models or correlational studies |
| Vittengl, J. R., Jarrett, R. B., Weitz, E., Hollon, S. D., Twisk, J., Cristea, I., David, D., DeRubeis, R. J., Dimidjian, S., Dunlop, B. W., Faramarzi, M., Hegerl, U., Kennedy, S. H., Kheirkhah, F., Mergl, R., Miranda, J., Mohr, D. C., Rush, A. J., Segal, Z. V., Siddique, J., Simons, A. D., & Cuijpers, P. (2016). Divergent Outcomes in Cognitive-Behavioral Therapy and Pharmacotherapy for Adult Depression. The American journal of psychiatry, 173(5), 481-490. https://doi.org/https://dx.doi.org/10.1176/appi.ajp.2015.15040492 | Study design not eligible |
| Voegeli, G., Clery-Melin, M. L., Ramoz, N., & Gorwood, P. (2017). Progress in Elucidating Biomarkers of Antidepressant Pharmacological Treatment Response: A Systematic Review and Meta-analysis of the Last 15 Years. Drugs, 77(18), 1967-1986. https://doi.org/https://dx.doi.org/10.1007/s40265-017-0819-9 | Only searched one database |
| Wang, J., Luo, H., Schülke, R., Geng, X., Sahakian, B. J., & Wang, S. (2021). Is transcranial direct current stimulation, alone or in combination with antidepressant medications or psychotherapies, effective in treating major depressive disorder? A systematic review and meta-analysis. *BMC medicine*, *19*(1), 319. https://doi.org/10.1186/s12916-021-02181-4 | No includible individual studies |
| Walther, A., Breidenstein, J., & Miller, R. (2019). Association of Testosterone Treatment With Alleviation of Depressive Symptoms in Men: A Systematic Review and Meta-analysis. JAMA psychiatry, 76(1), 31-40. https://doi.org/https://dx.doi.org/10.1001/jamapsychiatry.2018.2734 | Univariable models or correlational studies |
| Watanabe, N., Maruo, K., Imai, H., Ikeda, K., Yamawaki, S., & Furukawa, T. A. (2020). Predicting antidepressant response through early improvement of individual symptoms of depression incorporating baseline characteristics of patients: An individual patient data meta-analysis. Journal of psychiatric research, 125, 85-90. https://doi.org/https://dx.doi.org/10.1016/j.jpsychires.2020.03.009 | Study design not eligible |
| Wee, Z. Y., Yong, S. W. L., Chew, Q. H., Guan, C., Lee, T. S., & Sim, K. (2019). Actigraphy studies and clinical and biobehavioural correlates in schizophrenia: a systematic review. Journal of neural transmission (Vienna, Austria : 1996), 126(5), 531-558. https://doi.org/https://dx.doi.org/10.1007/s00702-019-01993-2 | Not investigating prognostic or predictive factors |
| Weimer, K., Colloca, L., & Enck, P. (2015). Age and sex as moderators of the placebo response - An evaluation of systematic reviews and meta-analyses across medicine. Gerontology, 61(2), 97-108. https://doi.org/https://dx.doi.org/10.1159/000365248 | Study design not eligible |
| Weitz, E. S., Hollon, S. D., Twisk, J., van Straten, A., Huibers, M. J., David, D., DeRubeis, R. J., Dimidjian, S., Dunlop, B. W., Cristea, I. A., Faramarzi, M., Hegerl, U., Jarrett, R. B., Kheirkhah, F., Kennedy, S. H., Mergl, R., Miranda, J., Mohr, D. C., Rush, A. J., Segal, Z. V., … Cuijpers, P. (2015). Baseline Depression Severity as Moderator of Depression Outcomes Between Cognitive Behavioral Therapy vs Pharmacotherapy: An Individual Patient Data Meta-analysis. *JAMA psychiatry*, *72*(11), 1102–1109. https://doi.org/10.1001/jamapsychiatry.2015.1516 | No includible individual studies |
| Welge, J. A., & Keck Jr, P. E. (2003). Moderators of placebo response to antipsychotic treatment in patients with schizophrenia: A meta-regression. Psychopharmacology, 166(1), 1-10. https://doi.org/http://dx.doi.org/10.1007/s00213-002-1299-4 | Not reporting outcomes of interest |
| Welten, C. C. M., Koeter, M. W. J., Wohlfarth, T., Storosum, J. G., van den Brink, W., Gispen-de Wied, C. C., Leufkens, H. G. M., & Denys, D. A. J. P. (2015). Placebo response in antipsychotic trials of patients with acute mania: Results of an individual patient data meta-analysis. European neuropsychopharmacology : the journal of the European College of Neuropsychopharmacology, 25(7), 1018-1026. https://doi.org/https://dx.doi.org/10.1016/j.euroneuro.2015.03.010 | Not investigating prognostic or predictive factors |
| Welten, C. C. M., Koeter, M. W. J., Wohlfarth, T. D., Storosum, J. G., van den Brink, W., Gispen-de Wied, C. C., Leufkens, H. G. M., & Denys, D. A. J. P. (2016). Early Nonresponse in the Antipsychotic Treatment of Acute Mania: A Criterion for Reconsidering Treatment? Results From an Individual Patient Data Meta-Analysis. The Journal of clinical psychiatry, 77(9), e1117-e1123. https://doi.org/https://dx.doi.org/10.4088/JCP.15r10051 | Study design not eligible |
| West, O., Hajek, P., & McRobbie, H. (2011). Systematic review of the relationship between the 3-hydroxycotinine/cotinine ratio and cigarette dependence. Psychopharmacology, 218(2), 313-322. https://doi.org/https://dx.doi.org/10.1007/s00213-011-2341-1 | Not reporting outcomes of interest |
| Whiston, A., Lennon, A., Brown, C., Looney, C., Larkin, E., O'Sullivan, L., Sik, N., & Semkovska, M. (2022). A Systematic Review and Individual Patient Data Network Analysis of the Residual Symptom Structure Following Cognitive-Behavioral Therapy and Escitalopram, Mirtazapine and Venlafaxine for Depression. Frontiers in Psychiatry, 13, 746678. https://doi.org/https://dx.doi.org/10.3389/fpsyt.2022.746678 | Not reporting outcomes of interest |
| Williams, T., Hattingh, C. J., Kariuki, C. M., Tromp, S. A., van Balkom, A. J., Ipser, J. C., & Stein, D. J. (2017). Pharmacotherapy for social anxiety disorder (SAnD). *The Cochrane database of systematic reviews*, *10*(10), CD001206. https://doi.org/10.1002/14651858.CD001206.pub3 | No includible individual studies |
| Woodbridge J, Townsend M, Reis S, Singh S, Grenyer BF. Non-response to psychotherapy for borderline personality disorder: A systematic review. Aust N Z J Psychiatry. 2022 Jul;56(7):771-787. doi: 10.1177/00048674211046893. Epub 2021 Sep 15. PMID: 34525867; PMCID: PMC9218414. | No includible individual studies |
| Woolf, C., Lampit, A., Shahnawaz, Z., Sabates, J., Norrie, L. M., Burke, D., Naismith, S. L., & Mowszowski, L. (2022). A Systematic Review and Meta-Analysis of Cognitive Training in Adults with Major Depressive Disorder. Neuropsychology review, 32(2), 419-437. https://doi.org/https://dx.doi.org/10.1007/s11065-021-09487-3 | Not investigating prognostic or predictive factors |
| Woo, Y. S., Seo, H. J., McIntyre, R. S., & Bahk, W. M. (2016). Obesity and Its Potential Effects on Antidepressant Treatment Outcomes in Patients with Depressive Disorders: A Literature Review. *International journal of molecular sciences*, *17*(1), 80. https://doi.org/10.3390/ijms17010080 | No includible individual studies |
| Wray, J. M., Gass, J. C., & Tiffany, S. T. (2013). A systematic review of the relationships between craving and smoking cessation. Nicotine & tobacco research : official journal of the Society for Research on Nicotine and Tobacco, 15(7), 1167-1182. https://doi.org/https://dx.doi.org/10.1093/ntr/nts268 | Univariable models or correlational studies |
| Wu, M. S., McGuire, J. F., Martino, C., Phares, V., Selles, R. R., & Storch, E. A. (2016). A meta-analysis of family accommodation and OCD symptom severity. Clinical Psychology Review, 45, 34-44. https://doi.org/https://dx.doi.org/10.1016/j.cpr.2016.03.003 | Not investigating prognostic or predictive factors |
| Xia, J., Merinder, L. B., & Belgamwar, M. R. (2011). Psychoeducation for schizophrenia. The Cochrane database of systematic reviews(6), CD002831. https://doi.org/https://dx.doi.org/10.1002/14651858.CD002831.pub2 | Not investigating prognostic or predictive factors |
| Yang, C., Wardenaar, K. J., Bosker, F. J., Li, J., & Schoevers, R. A. (2019). Inflammatory markers and treatment outcome in treatment resistant depression: A systematic review. Journal of Affective Disorders, 257, 640-649. https://doi.org/https://dx.doi.org/10.1016/j.jad.2019.07.045 | Only searched one database |
| Yoon, S., Kim, Y., & Lee, S.-H. (2021). Does the Loudness Dependence of Auditory Evoked Potential Predict Response to Selective Serotonin Reuptake Inhibitors?: A Meta-analysis. Clinical psychopharmacology and neuroscience : the official scientific journal of the Korean College of Neuropsychopharmacology, 19(2), 254-261. https://doi.org/https://dx.doi.org/10.9758/cpn.2021.19.2.254 | Univariable models or correlational studies |
| Yosaee, S., Clark, C. C. T., Keshtkaran, Z., Ashourpour, M., Keshani, P., & Soltani, S. (2022). Zinc in depression: From development to treatment: A comparative/ dose response meta-analysis of observational studies and randomized controlled trials. General Hospital Psychiatry, 74, 110-117. https://doi.org/https://dx.doi.org/10.1016/j.genhosppsych.2020.08.001 | Study design not eligible |
| Yoshikawa, A., Li, J., & Meltzer, H. Y. (2020). A functional HTR1A polymorphism, rs6295, predicts short-term response to lurasidone: confirmation with meta-analysis of other antipsychotic drugs. The pharmacogenomics journal, 20(2), 260-270. https://doi.org/https://dx.doi.org/10.1038/s41397-019-0101-5 | Study design not eligible |
| Yu, B. C. L., Chio, F. H. N., Mak, W. W. S., Corrigan, P. W., & Chan, K. K. Y. (2021). Internalization process of stigma of people with mental illness across cultures: A meta-analytic structural equation modeling approach. Clinical Psychology Review, 87, 102029. https://doi.org/https://dx.doi.org/10.1016/j.cpr.2021.102029 | Not reporting outcomes of interest |
| Zantvoord, J. B., Diehle, J., & Lindauer, R. J. (2013). Using neurobiological measures to predict and assess treatment outcome of psychotherapy in posttraumatic stress disorder: systematic review. *Psychotherapy and psychosomatics*, *82*(3), 142–151. https://doi.org/10.1159/000343258 | No includible individual studies |
| Zhang, M.-M., Ma, Y., Du, L.-T., Wang, K., Li, Z., Zhu, W., Sun, Y.-H., Lu, L., Bao, Y.-P., & Li, S.-X. (2022). Sleep disorders and non-sleep circadian disorders predict depression: A systematic review and meta-analysis of longitudinal studies. Neuroscience and biobehavioral reviews, 134, 104532. https://doi.org/https://dx.doi.org/10.1016/j.neubiorev.2022.104532 | Not reporting outcomes of interest |
| Zhang, Y., Zhou, B., Qiu, J., Zhang, L., & Zou, Z. (2020). Heart rate variability changes in patients with panic disorder. Journal of Affective Disorders, 267, 297-306. https://doi.org/https://dx.doi.org/10.1016/j.jad.2020.01.132 | Not investigating prognostic or predictive factors |
| Zhao, S., Sampson, S., Xia, J., & Jayaram, M. B. (2015). Psychoeducation (brief) for people with serious mental illness. *The Cochrane database of systematic reviews*, (4), CD010823. https://doi.org/10.1002/14651858.CD010823.pub2 | No includible individual studies |
| Zhou, D. D., Zhou, X. X., Li, Y., Zhang, K. F., Lv, Z., Chen, X. R., Wan, L. Y., Wang, W., Wang, G. M., Li, D. Q., Ai, M., & Kuang, L. (2019). Augmentation agents to serotonin reuptake inhibitors for treatment-resistant obsessive-compulsive disorder: A network meta-analysis. Progress in Neuro-Psychopharmacology and Biological Psychiatry, 90, 277-287. https://doi.org/https://dx.doi.org/10.1016/j.pnpbp.2018.12.009 | Not investigating prognostic or predictive factors |
| Ziser, K., Molbert, S. C., Stuber, F., Giel, K. E., Zipfel, S., & Junne, F. (2018). Effectiveness of body image directed interventions in patients with anorexia nervosa: A systematic review. The International journal of eating disorders, 51(10), 1121-1127. https://doi.org/https://dx.doi.org/10.1002/eat.22946 | Not investigating prognostic or predictive factors |

**eTable 2. Systematic reviews reporting on multivariable models testing any predictors of response, remission, recovery, relapse across mental disorders**

| **Author, year** | **Mental disorders** | **Age group** | **Included/**  **overall studies** | **Included sample size** | **Predictors** | **Level III predictors** | **Outcomes** | **Q** |
| --- | --- | --- | --- | --- | --- | --- | --- | --- |
| Aardoom,2013(132) | Feeding and eating disorders | Mixed | 2/21 | 217 | 5 | Clinical (5) | Remission | CL |
| AlAqeel, 2012(133) | Schizophrenia-spectrum and other psychotic disorders | Mixed | 12/27 | 3894 | 121 | Clinical (90) ; pharmacology/treatment (8) ; sociodemographics/environmental (23) ; | Relapse; Remission | CL |
| Alvarez-Jimenez, 2012(134) | Schizophrenia-spectrum and other psychotic disorders | Mixed | 10/29 | 1191 | 84 | Biochemical (3); clinical (49); neuroimaging (6); neuropsychological (11); pharmacology/treatment (1) sociodemographics/environmental (14) | Relapse; Remission | CL |
| Amati, 2018(135) | Mixed disorders | Adults | 1/19 | 19395 | 14 | Clinical (13); pharmacological/treatment (1) | Remission | CL |
| Archie, 2009(136) | Schizophrenia-spectrum and other psychotic disorders | Mixed | 3/12 | 254 | 15 | Clinical (11); sociodemographics/environmental (4) | Relapse | CL |
| Arias,2020(137) | Bipolar and related disorders | Mixed | 2/5 | 2066 | 16 | Clinical (11); pharmacological/treatment (1); sociodemographics/environmental (4) | Relapse; Remission | CL |
| Arteaga-Henriquez, 2019(138) | Depressive disorders | Mixed | 4/24 | 243 | 16 | Biochemical (1); clinical (2); genetics (9); sociodemographic/environmental (4); | Response | CL |
| Barbosa, 2019(139) | Anxiety disorders | Mixed | 1/13 | 23 | 10 | Clinical (4); neuroimaging (2); sociodemographics/environmental (4) | Remission | CL |
| Berends, 2018(140) | Feeding and eating disorders | Mixed | 6/16 | 1359 | 45 | Clinical (42); sociodemographics/environmental (3) | Relapse; Remission | CL |
| Bonvicini, 2016(141) | Neurodevelopmental disorders | Adults | 1/23 | 171 | 6 | Clinical (4); sociodemographics/environmental (2) | Response | CL |
| Bowtell, 2018(142) | Schizophrenia-spectrum and other psychotic disorders | Mixed | 2/11 | 197 | 7 | Clinical (7) | Relapse | CL |
| Boylan, 2020(143) | Depressive disorders | Children/Adolescents | 6/21 | 1767 | 73 | Biochemical (1); clinical (35); genetics (19); pharmacology/treatment (4); sociodemographics/environmental (14) | Remission; Response | CL |
| Breitenstein, 2015(144) | Depressive disorders | Mixed | 1/15 | 71 | 8 | Clinical (2); genetics (2); sociodemographics/environmental (4) | Response | CL |
| Brunoni 2011(145) | Depressive disorders | Mixed | 4/12 | 714 | 45 | Clinical (15) ; neuroimaging (18) ; neuropsychological (5) ; sociodemographics/environmental (7) | Remission; Response | CL |
| Buckman, 2018(146) | Depressive disorders | Adults | 7/12 | 2340 | 109 | Biochemical (4); clinical (41); genetics (8); neuropsychological (3); pharmacology (4); psychological (10); sociodemographics/environmental (39) | Relapse | CL |
| Caldirola, 2020(147) | Anxiety disorders | Mixed | 1/7 | 51 | 11 | Clinical (10); pharmacology (1) | Relapse | CL |
| Carter, 2012(148) | Depressive disorders | Adults | 11/57 | 5529 | 122 | Clinical (48); neuropsychological (4); pharmacology/treatment (30); psychological (11); sociodemographics/environmental (29) | Recovery; Remission;  Response | CL |
| Catalan, 2021(149) | Schizophrenia-spectrum disorders and mood disorders with psychotic features | Mixed | 23/99 | 5714 | 497 | Clinical (339); neuropsychological (4); pharmacology/treatment (6); psychological (1); sociodemographics/environmental (147) | Recovery;  Relapse;  Remission | CL |
| Cohen, 2021(150) | Depressive disorders | Mixed | 2/22 | 69 | 5 | neuroimaging (2); sociodemographics/environmental (3) | Remission;  Response | L |
| Colle, 2018(151) | Depressive disorders | Mixed | 2/6 | 102 | 18 | neuroimaging (15); sociodemographics/environmental (3) | Remission | CL |
| Courtney 2022(152) | Depressive disorders | Children/Adolescents | 1/81 | 107 | 18 | Clinical (13) ; pharmacology/treatment (1) ; psychological (1) ; sociodemographics/environmental (3) | Relapse ;  Remission | M |
| De Carlo, 2016(153) | Depressive disorders | Adults | 3/51 | 430 | 11 | Clinical (5); psychological (2); sociodemographics/environmental (4); | Remission; Response | CL |
| Dewa 2021(154) | Schizophrenia-spectrum disorders and mood disorders with psychotic features | Mixed | 1/5 | 97 | 6 | Clinical (4); psychological (1); sociodemographics/environmental (1) | Relapse | L |
| Diaz-Caneja 2015(155) | Schizophrenia-spectrum disorders and mood disorders with psychotic features | Children/Adolescents | 3/75 | 151 | 18 | Clinical (12); neuropsychological (2); sociodemographic/environmental (4) | Relapse; Remission | CL |
| Du, 2020(156) | Depressive disorders | Mixed | 2/16 | 320 | 9 | Clinical (3) ; genetics (3) ; sociodemographic/environmental (2) ; psychological (1) | Response | CL |
| Eskild-Jensen 2020(157) | Feeding and eating disorders | Mixed | 6/15 | 1112 | 42 | Clinical (33); sociodemographic/environmental (8); psychological (1) | Relapse; Recovery; Remission | CL |
| Estrada-Prat, 2019(158) | Bipolar and related disorders | Mixed | 7/48 | 1636 | 57 | Clinical (42); pharmacological/treatment (2); sociodemographic/environmental (13) | Relapse; Remission | CL |
| Farooq 2009(159) | Schizophrenia-spectrum disorders and mood disorders with psychotic features | Adults | 2/11 | 239 | 8 | Clinical (5) ; pharmacological/treatment (1) ; sociodemographic/environmental (2) | Relapse; Remission | CL |
| Fekadu 2009(160) | Depressive disorders | Adults | 1/9 | 96 | 8 | Clinical (6); sociodemographic/environmental (2) | Relapse | CL |
| Fisher 2017(161) | Depressive disorders | Adults | 3/39 | 307 | 39 | Biochemical (22); clinical (6); genetics (2); sociodemographic/environmental (9) | Remission;  Response | L |
| Ferreira-Garcia 2017(162) | Anxiety disorders | Adults | 1/24 | 268 | 3 | Clinical (2); psychological (1) | Remission | CL |
| Forster 2018(163) | Substance-related and addictive disorders | Adults | 4/24 | 184 | 38 | Clinical (20); neuroimaging (18) | Relapse | CL |
| Foulds 2017(164) | Substance-related and addictive disorders | Adults | 4/18 | 572 | 23 | Clinical (8); pharmacological/treatment (1); psychological (12); sociodemographic (4) | Relapse | L |
| Fournier, 2022(165) | Depressive disorders | Adults | 1/4 | 84 | 7 | Clinical (5); sociodemographic/environmental (2) | Relapse | M |
| Frostad, 2022(166) | Feeding and eating disorders | Mixed | 1/19 | 41 | 3 | Biochemical (2); clinical (1) | Remission | M |
| Fusar-Poli, 2016(167) | Schizophrenia-spectrum and other psychotic disorders | Mixed | 3/84 | 1708 | 28 | Clinical (24); sociodemographic/environmental (4) | Relapse; Recovery | L |
| Garbutt, 2014(168) | Substance-related and addictive disorders | Adults | 1/23 | 63 | 7 | Clinical (1); genetics (1); sociodemographic/environmental (2) | Relapse | L |
| Ghabrash, 2020(169) | Substance-related and addictive disorders | Mixed | 2/18 | 225 | 18 | Clinical (9) ; neuropsychological (1) ; pharmacological/treatment (1) ; psychological (6) ; sociodemographic/environmental (1) | Remission | CL |
| Giblino, 2014(170) | Depressive disorders | Adults | 3/109 | 597 | 13 | Biochemical (1); clinical (5); pharmacological/treatment (2); sociodemographic/environmental (5) | Relapse; Remission | CL |
| Gimenez-Palomo, 2022(171) | Bipolar and related disorders | Mixed | 1/36 | 118 | 36 | Clinical (32); sociodemographic/environmental (4) | Remission | CL |
| Glashouwer 2019(172) | Feeding and eating disorders | Mixed | 6/44 | 803 | 36 | Clinical (31); psychological (1); sociodemographic/environmental (4) | Recovery; Relapse;  Remission Response | L |
| Grillault-Laroche, 2020(173) | Bipolar and related disorders | Adults | 6/34 | 6055 | 75 | Biochemical (14); clinical (36); neuroimaging (5); pharmacological/treatment (4); sociodemographic/environmental (16) | Relapse;  Remission;  Response | M |
| Groves, 2018(174) | Depressive disorders | Adults | 8/41 | 1120 | 84 | Clinical (8) ; genetics (3) ; neuropsychological (53) ; sociodemographic/environmental (20) | Remission; Response | CL |
| Hamadi, 2020(175) | Feeding and eating disorders | Children/Adolescents | 2/28 | 201 | 12 | Clinical (11); pharmacological/treatment (1) | Remission | L |
| Haq, 2015(176) | Depressive disorders | Adults | 2/32 | 542 | 8 | Clinical (6); sociodemographic/environmental (2) | Remission; Response | L |
| Hardevereld, 2010(177) | Depressive disorders | Adults | 1/26 | 176 | 7 | Clinical (5); sociodemographic/environmental (2) | Relapse | CL |
| Hui, 2019(178) | Bipolar and related disorders | Adults | 5/71 | 6179 | 74 | Biochemical (14); clinical (36); pharmacological/treatment (10); sociodemographic/environmental 14) | Relapse;  Remission | CL |
| Huijbregts, 2010(179) | Depressive disorders | Mixed | 1/9 | 40 | 4 | Clinical (4) | Response | CL |
| Ipser, 2012(180) | Trauma and stress-related disorders | Adults | 1/41 | 65 | 6 | Clinical (3); sociodemographic/environmental (3) | Relapse | M |
| Johnson, 2022(181) | Bipolar and related disorders | Mixed | 4/30 | 316 | 29 | Clinical (19); psychological (6); sociodemographic/environmental (4) | Relapse; Remission | CL |
| Kappelmann, 2020(182) | Depressive disorders | Adults | 1/20 | 94 | 8 | Clinical (4); sociodemographic/environmental (4) | Relapse | H |
| Kastner, 2019(183) | Feeding and eating disorders | Mixed | 3/68 | 372 | 18 | Clinical (11); pharmacological/treatment (4); psychological (3) | Relapse; Remission | CL |
| Kawaguchi, 2014(184) | Mixed disorders | Mixed | 2/5 | 581 | 33 | Clinical (4); genetics (18);pharmacological/treatment (3); sociodemographic/environmental (8) | Remission; Response | CL |
| Kennis, 2020(185) | Depressive disorders | Adults | 8/75 | 1050 | 59 | Biochemical (17); clinical (26); neuropsychological (2); pharmacological/treatment (4); sociodemographic/environmental (10) | Relapse | L |
| Khalsa, 2017(186) | Feeding and eating disorders | Mixed | 5/20 | 657 | 42 | Clinical (38); psychological (1); sociodemographic/environmental (3) | Recovery;  Relapse; Remission | CL |
| Kleindienst, 2005(187) | Bipolar and related disorders | Mixed | 3/45 | 202 | 42 | Biochemical (14) ; clinical (16) ; neuroimaging (5) ; sociodemographic/environmental (7) | Remission; Response | CL |
| Kunas, 2021(188) | Anxiety disorders | Mixed | 7/23 | 1313 | 90 | Clinical (55); pharmacology/treatment (10); sociodemographic/environmental (25) | Remission; Response | L |
| Lee, 2018(189) | Depressive disorders | Adults | 7/26 | 5747 | 95 | Clinical (51); neuroimaging (2); neurophysiological (22); pharmacological/treatment (3); psychological (1); sociodemographic/environmental (16) | Remission; Response | CL |
| Lee, 2022(190) | Schizophrenia-spectrum and other psychotic disorders | Mixed | 4/13 | 2704 | 79 | Clinical (49); sociodemographic/environmental (20) | Relapse; Remission | L |
| Lewy, 2019(191) | Major depressive disorder and bipolar disorder | Adults | 4/18 | 197 | 28 | Clinical (3); neuroimaging (8); neuropsychological (5); sociodemographic/environmental (10) | Relapse;  Remission;  Response | CL |
| Linardon, 2016(192) | Feeding and eating disorders | Mixed | 2/23 | 122 | 15 | Clinical (14); psychological (1) | Remission | L |
| Liu, 2020(193) | Depressive disorders | Adults | 1/44 | 107 | 4 | Biochemical (1); clinical (1); sociodemographic/environmental (2); | Response | L |
| Lorimer, 2021(194) | Anxiety disorders | Adults | 1/9 | 56 | 3 | sociodemographic/environmental (3) | Relapse | H |
| Leuken, 2016(195) | Anxiety disorders | Mixed | 5/60 | 508 | 30 | Clinical (18); genetics (3); neuroimaging (2); neuropsychological (1); sociodemographic/environmental (6) | Remission; Response | H |
| Lundkvist-  Hondoumadi,  2014(196) | Anxiety disorders | Children/Adolescents | 1/27 | 124 | 2 | Clinical (2) | Remission | CL |
| Marsche, 2020(197) | Depressive disorders | Elderly | 6/29 | 1061 | 85 | Biochemical (1); clinical (28); genetics (27); neuroimaging (4); pharmacological/treatment (6); sociodemographic/environmental (19) | Remission; Response | CL |
| Marshall, 2005(198) | Schizophrenia-spectrum and other psychotic disorders | Mixed | 2/26 | 267 | 11 | Clinical (6); sociodemographic/environmental (4) | Recovery | M |
| Martland, 2020(199) | Schizophrenia-spectrum and other psychotic disorders | Adults | 2/23 | 162 | 10 | Clinical (5); neuropsychological (2); sociodemographic/environmental (3) | Relapse | CL |
| Masse 2018(200) | Depressive disorders | Elderly | 7/13 | 548 | 80 | Clinical (15); neuroimaging (25); neuropsychological (17); pharmacology/treatment (1); sociodemographic/environmental (22) | Remission | CL |
| Masse-Sibille, 2018(201) | Depressive disorders | Elderly | 14/59 | 1409 | 71 | Clinical (26); genetics (3); neuroimaging (6); neuropsychological (19); pharmacology/treatment (3); sociodemographic/environmental (12) | Relapse; Remission | CL |
| Meehan, 2022(7) | Mixed disorders | Mixed | 36/228 | 159299 | 476 | Biochemical (4); clinical (324); genetics (23); neuroimaging (1); neurophysiological (1); neuropsychological (6); pharmacological/treatment (9); psychological (11); sociodemographic/environmental (96) | Recovery; Relapse; Remission; Response | M |
| Meerman, 2022(202) | Depressive disorders | Mixed | 1/11 | 335 | 36 | Clinical (26); genetics (2); sociodemographic/environmental (8) | Remission | CL |
| Mitchell, 2005(203) | Depressive disorders | Mixed | 1/31 | 338 | 5 | Clinical (1); psychological (1); sociodemographic/environmental (3) | Remission | CL |
| Moriarty, 2021(204) | Depressive disorders | Adults | 5/11 | 2643 | 80 | Biochemical (1); clinical (44); neuropsychological (3); pharmacological/treatment (2); psychological (1); sociodemographic/environmental (29) | Relapse | H |
| Mulder, 2002(205) | Depressive disorders | Mixed | 1/53 | 623 | 5 | Clinical (1); psychological (2); sociodemographic/environmental (2) | Response | CL |
| Mululo, 2012(206) | Anxiety disorders | Mixed | 1/9 | 30 | 9 | Clinical (9) | Response | CL |
| Najt, 2011(207) | Mixed disorders | Mixed | 5/26 | 869 | 42 | Clinical (24); sociodemographic/environmental (18) | Relapse; Remission; Response | CL |
| Nanni, 2012(208) | Depressive disorders | Mixed | 7/26 | 4012 | 58 | Clinical (20); pharmacological/treatment (2); psychological (2); sociodemographic/environmental (34) | Recovery; Relapse; Remission; Response | L |
| Nazar, 2017(209) | Feeding and eating disorders | Mixed | 4/24 | 323 | 22 | Clinical (17) ; pharmacological/treatment (1) ; sociodemographic/environmental (4) | Remission | M |
| Ng, 2016(210) | Personality disorders | Mixed | 1/15 | 275 | 8 | Clinical (2); psychological (3); sociodemographic/environmental (3) | Remission | M |
| Nilsen, 2013(211) | Mixed disorders | Children/Adolescents | 2/45 | 231 | 2 | Clinical (2) | Remission | L |
| Norman, 2005(212) | Schizophrenia-spectrum and other psychotic disorders | Mixed | 2/13 | 136 | 11 | Clinical (5); sociodemographic/environmental (6) | Recovery; Remission | M |
| Park, 2018(213) | Depressive disorders | Mixed | 1/7 | 30 | 2 | Clinical (1); neuropsychological (1) | Remission | CL |
| Penttila, 2014(214) | Schizophrenia-spectrum and other psychotic disorders | Mixed | 6/39 | 1964 | 81 | Clinical (50); neuropsychological (1); sociodemographic/environmental (30) | Recovery; Remission; Relapse | L |
| Pisanu, 2022(215) | Depressive disorders | Mixed | 3/123 | 136 | 12 | Clinical (1); genetics (9); sociodemographic/environmental (2) | Response | L |
| Raffin, 2008(216) | Obsessive-compulsive and related disorders | Mixed | 2/21 | 160 | 8 | Clinical (7); sociodemographic/environmental (1) | Remission;  Response | CL |
| Rao, 2019(217) | Mixed disorders | Mixed | 1/56 | 222 | 7 | Clinical (3); genetics (2); sociodemographic/environmental (2) | Response | H |
| Reich, 2018(218) | Mixed disorders | Mixed | 6/27 | 1312 | 136 | Clinical (94) ; pharmacological/treatment (2) ; psychological (12) ; sociodemographic/environmental (28) | Relapse; Remission; Recovery | L |
| Santesteban-Echarri, 2017(219) | Schizophrenia-spectrum and other psychotic disorders | Mixed | 3/50 | 1451 | 21 | Clinical (14); neuropsychological (1); sociodemographic/environmental (6) | Recovery; Remission | CL |
| Salazar 2021(11) | Mixed disorders | Mixed | 12/89 | 8990 | 474 | Clinical (86); genetics (325); neuroimaging (8); neurophysiological (7); neuropsychological (3); pharmacological/treatment (4); sociodemographic/environmental (41) | Relapse; Remission; Response | CL |
| Sappala, 2021(220) | Schizophrenia-spectrum and other psychotic disorders | Adults | 1/77 | 30 | 2 | Clinical (2) | Response | CL |
| Seeberg, 2018(221) | Depressive disorders | Mixed | 7/60 | 1242 | 81 | Clinical (5); genetics (3); neuroimaging (8); neuropsychological (38); pharmacological/treatment (6); psychological (6); | Remission; Response | CL |
| Senior 2021(222) | Bipolar and related disorders | Adults | 1/100 | 363 | 11 | Clinical (10); sociodemographic/environmental (1) | Relapse | CL |
| Silverstein, 2015(223) | Mixed disorders | Mixed | 1/41 | 33 | 4 | Neuroimaging (4) | Response | CL |
| Siskind , 2021(224) | Schizophrenia-spectrum and other psychotic disorders | Mixed | 1/20 | 61 | 2 | Clinical (2) | Response | L |
| Sliederecht, 2019(225) | Alcohol dependence | Mixed | 62/320 | 37410 | 474 | Biochemical (6); clinical (239); genetics (4); neuroimaging (16); neurophysiological (7); pharmacological/treatment ( 7); psychological (16); sociodemographic/environmental (179) | Recovery; Relapse; Remission | CL |
| Strawbridge, 2015(226) | Depressive disorders | Adults | 1/35 | 107 | 4 | Biochemical (1); clinical (1); sociodemographic/environmental (2) | Response | CL |
| Sullivan 2017(227) | Schizophrenia-spectrum and other psychotic disorders | Adults | 1/2 | 339 | 13 | Clinical (11); pharmacological/treatment (2) | Relapse | L |
| Taylor, 2019(228) | Depressive disorders | Mixed | 4/23 | 407 | 28 | Biochemical (4); clinical (21); pharmacological/treatment (1); sociodemographic/environmental (2) | Remission; Response | CL |
| Ter Hark, 2022(229) | Depressive disorders | Mixed | 4/37 | 1355 | 59 | Clinical (6); genetics (53) | Relapse; Remission; Response | CL |
| Thiel, 2013(230) | Obsessive-compulsive and related disorders | Adults | 1/23 | 100 | 3 | Clinical (2); sociodemographic/environmental (1) | Remission | CL |
| Tunvirachaisakul, 2018(231) | Depressive disorders | Elderly | 11/67 | 2643 | 186 | Biochemical (1); clinical (78); neuroimaging (7); neuropsychological (17); pharmacological/treatment (51); sociodemographic/environmental (32) | Remission; Response | CL |
| Turner 2018(232) | Obsessive-compulsive and related disorders | Children/Adolescents | 1/34 | 78 | 5 | Clinical (4); sociodemographic/environmental (1) | Remission | L |
| Valiengo,2022(233) | Depressive disorders | Elderly | 1/37 | 92 | 7 | Neuroimaging (7) | Response | CL |
| Van, 2008(234) | Depressive disorders | Mixed | 6/22 | 3558 | 30 | Clinical (19); pharmacology-treatment (3); sociodemographic/environmental (8) | Recovery; Remission; Response | CL |
| Van der Burg, 2021(235) | Depressive disorders | Mixed | 1/35 | 108 | 5 | Clinical (1) ; pharmacological/treatment (1) ; sociodemographic/environmental (3) | Remission | CL |
| Van der Velden, 2015(236) | Depressive disorders | Adults | 2/23 | 284 | 7 | Clinical (4); pharmacology-treatment (3) | Relapse | CL |
| Van Diermen, 2018(237) | Mixed disorders | Adults | 7/34 | 1239 | 74 | Clinical (50); pharmacological/treatment (7); sociodemographic/environmental (17) | Remission; Response | CL |
| Wagner, 2017(238) | Depressive disorders | Mixed | 1/17 | 120 | 9 | Clinical (6); pharmacology-treatment (1); sociodemographic/environmental (2) | Response | CL |
| Wang, 2018(239) | Mixed disorders | Adults | 8/34 | 1498 | 97 | Clinical (33) ; neuropsychological (1) ; pharmacological/treatment (3) ; sociodemographic/environmental (50) | Relapse;  Remission;  Response | L |
| Webb, 2020(240) | Depressive disorders | Mixed | 1/24 | 85 | 16 | Clinical (4); genetics (8); sociodemographic/environmental (4) | Remission | CL |
| Werz, 2022(241) | Feeding and eating disorders | Mixed | 4/26 | 310 | 14 | Clinical (11); sociodemographic/environmental (3) | Recovery; Remission | CL |
| Whiteford, 2013(242) | Depressive disorders | Mixed | 1/21 | 609 | 8 | Clinical (3); psychological (1); sociodemographic/environmental (4) | Relapse | CL |
| Widge, 2018(243) | Mixed disorders | Adults | 6/73 | 255 | 35 | Clinical (5); neurophysiological (27); sociodemographic/environmental (3) | Response | CL |
| Wojnarowski, 2019(244) | Depressive disorders | Adults | 5/13 | 549 | 32 | Clinical (15); psychological (9); sociodemographic/environmental (8) | Relapse; Remission | CL |
| Yeo, 2022(245) | Schizophrenia-spectrum and other psychotic disorders | Adults | 1/37 | 80 | 9 | Clinical (6); sociodemographic/environmental (3) | Recovery; Remission | CL |
| Zhou, 2021(246) | Mixed disorders | Mixed | 3/37 | 360 | 58 | Clinical (10); genetics (38); sociodemographic/environmental (10) | Remission; Response | CL |

*Legend. CL= Critically Low; H= High; K, number of included studies; L= Low; M= Moderate; Q, quality of included systematic reviews measured with Assessment of multiple systematic reviews (AMSTAR) 2; * all factors in the model are reported*

**eTable 3. List of individual studies included in eligible systematic reviews, reporting on multivariable models of response, remission, recovery, relapse in people with mental disorders defined according to Diagnostic and Statistical Manual or International Classification of Diseases criteria, any version.**

| **Author, Year** | **Reference** |
| --- | --- |
| **Abdullah-Koolmees, 2018** | Abdullah-Koolmees, H., Gardarsdottir, H., Minnema, L. A., Elmi, K., Stoker, L. J., Vuyk, J., Goedhard, L. E., Egberts, T. C. G., & Heerdink, E. R. (2018). Predicting rehospitalization in patients treated with antipsychotics: a prospective observational study. *Therapeutic advances in psychopharmacology*, *8*(8), 213–229. |
| **Aberg-Wistedt, 2000** | Aberg-Wistedt, A., Agren, H., Ekselius, L., Bengtsson, F., & Akerblad, A. C. (2000). Sertraline versus paroxetine in major depression: clinical outcome after six months of continuous therapy. *Journal of clinical psychopharmacology*, *20*(6), 645–652. |
| **Adamczyk, 2015** | Adamczyk, M., Gazea, M., Wollweber, B., Holsboer, F., Dresler, M., Steiger, A., & Pawlowski, M. (2015). Cordance derived from REM sleep EEG as a biomarker for treatment response in depression--a naturalistic study after antidepressant medication. *Journal of psychiatric research*, *63*, 97–104. |
| **Addington, 2008** | Addington, J., & Addington, D. (2008). Symptom remission in first episode patients. *Schizophrenia research*, *106*(2-3), 281–285. |
| **Adinoff, 2015** | Adinoff, B., Gu, H., Merrick, C., McHugh, M., Jeon-Slaughter, H., Lu, H., Yang, Y., & Stein, E. A. (2015). Basal Hippocampal Activity and Its Functional Connectivity Predicts Cocaine Relapse. *Biological psychiatry*, *78*(7), 496–504. |
| **Aguiar, 2012** | Aguiar, P., Neto, D., Lambaz, R., Chick, J., & Ferrinho, P. (2012). Prognostic factors during outpatient treatment for alcohol dependence: cohort study with 6 months of treatment follow-up. *Alcohol and alcoholism (Oxford, Oxfordshire)*, *47*(6), 702–710. |
| **Ajnarkina, 2018** | Ajnakina, O., Trotta, A., Forti, M. D., Stilo, S. A., Kolliakou, A., Gardner-Sood, P., Lopez-Morinigo, J., Gaughran, F., David, A. S., Dazzan, P., Pariante, C., Mondelli, V., Murray, R. M., & Fisher, H. L. (2018). Different types of childhood adversity and 5-year outcomes in a longitudinal cohort of first-episode psychosis patients. *Psychiatry research*, *269*, 199–206. |
| **Alexopolous, 2005** | Alexopoulos, G. S., Katz, I. R., Bruce, M. L., Heo, M., Ten Have, T., Raue, P., Bogner, H. R., Schulberg, H. C., Mulsant, B. H., Reynolds, C. F., 3rd, & PROSPECT Group (2005). Remission in depressed geriatric primary care patients: a report from the PROSPECT study. *The American journal of psychiatry*, *162*(4), 718–724. |
| **Alexopoulos, 2004** | Alexopoulos, G. S., Kiosses, D. N., Murphy, C., & Heo, M. (2004). Executive dysfunction, heart disease burden, and remission of geriatric depression. *Neuropsychopharmacology : official publication of the American College of Neuropsychopharmacology*, *29*(12), 2278–2284. |
| **Alexopoulos, 2010** | Alexopoulos, G. S., Glatt, C. E., Hoptman, M. J., Kanellopoulos, D., Murphy, C. F., Kelly, R. E., Jr, Morimoto, S. S., Lim, K. O., & Gunning, F. M. (2010). BDNF val66met polymorphism, white matter abnormalities and remission of geriatric depression. *Journal of affective disorders*, *125*(1-3), 262–268. |
| **Altamura, 2001** | Altamura, A. C., Bassetti, R., Sassella, F., Salvadori, D., & Mundo, E. (2001). Duration of untreated psychosis as a predictor of outcome in first-episode schizophrenia: a retrospective study. *Schizophrenia research*, *52*(1-2), 29–36 |
| **Alvarez, 1997** | Alvarez, E., Pérez-Solá, V., Pérez-Blanco, J., Queraltó, J. M., Torrubia, R., & Noguera, R. (1997). Predicting outcome of lithium added to antidepressants in resistant depression. *Journal of affective disorders*, *42*(2-3), 179–186. |
| **Ansell, 2011b** | Ansell, E. B., Pinto, A., Edelen, M. O., Markowitz, J. C., Sanislow, C. A., Yen, S., Zanarini, M., Skodol, A. E., Shea, M. T., Morey, L. C., Gunderson, J. G., McGlashan, T. H., & Grilo, C. M. (2011). The association of personality disorders with the prospective 7-year course of anxiety disorders. *Psychological medicine*, *41*(5), 1019–1028. |
| **Appelhof,2006** | Appelhof, B. C., Huyser, J., Verweij, M., Brouwer, J. P., van Dyck, R., Fliers, E., Hoogendijk, W. J., Tijssen, J. G., Wiersinga, W. M., & Schene, A. H. (2006). Glucocorticoids and relapse of major depression (dexamethasone/corticotropin-releasing hormone test in relation to relapse of major depression). *Biological psychiatry*, *59*(8), 696–701. |
| **Asarnow, 2009** | Asarnow, J. R., Emslie, G., Clarke, G., Wagner, K. D., Spirito, A., Vitiello, B., Iyengar, S., Shamseddeen, W., Ritz, L., Birmaher, B., Ryan, N., Kennard, B., Mayes, T., DeBar, L., McCracken, J., Strober, M., Suddath, R., Leonard, H., Porta, G., Keller, M., … Brent, D. (2009). Treatment of selective serotonin reuptake inhibitor-resistant depression in adolescents: predictors and moderators of treatment response. *Journal of the American Academy of Child and Adolescent Psychiatry*, *48*(3), 330–339. |
| **Askland, 2015** | Askland, K. D., Garnaat, S., Sibrava, N. J., Boisseau, C. L., Strong, D., Mancebo, M., Greenberg, B., Rasmussen, S., & Eisen, J. (2015). Prediction of remission in obsessive compulsive disorder using a novel machine learning strategy. *International journal of methods in psychiatric research*, *24*(2), 156–169. |
| **Azar, 2011** | Azar, A. R., Chopra, M. P., Cho, L. Y., Coakley, E., & Rudolph, J. L. (2011). Remission in major depression: results from a geriatric primary care population. *International journal of geriatric psychiatry*, *26*(1), 48–55. |
| **Bach, 2015** | Bach, P., Vollsta Dt-Klein, S., Kirsch, M., Hoffmann, S., Jorde, A., Frank, J., Charlet, K., Beck, A., Heinz, A., Walter, H., Sommer, W. H., Spanagel, R., Rietschel, M., & Kiefer, F. (2015). Increased mesolimbic cue-reactivity in carriers of the mu-opioid-receptor gene OPRM1 A118G polymorphism predicts drinking outcome: a functional imaging study in alcohol dependent subjects. *European neuropsychopharmacology : the journal of the European College of Neuropsychopharmacology*, *25*(8), 1128–1135. |
| **Bach, 2019** | Bach, P., Bumb, J. M., Schuster, R., Vollstädt-Klein, S., Reinhard, I., Rietschel, M., Witt, S. H., Wiedemann, K., Kiefer, F., & Koopmann, A. (2019). Effects of leptin and ghrelin on neural cue-reactivity in alcohol addiction: Two streams merge to one river?. *Psychoneuroendocrinology*, *100*, 1–9. |
| **Bagby, 1999** | Bagby, R. M., Rector, N. A., Segal, Z. V., Joffe, R. T., Levitt, A. J., Kennedy, S. H., & Levitan, R. D. (1999). Rumination and distraction in major depression: assessing response to pharmacological treatment. *Journal of affective disorders*, *55*(2-3), 225–229. |
| **Bares, 2012** | Bares, M., Novak, T., Brunovsky, M., Kopecek, M., Stopkova, P., Krajca, V., & Höschl, C. (2012). The change of QEEG prefrontal cordance as a response predictor to antidepressive intervention in bipolar depression. A pilot study. *Journal of psychiatric research*, *46*(2), 219–225. |
| **Bares, 2015** | Bares, M., Novak, T., Kopecek, M., Brunovsky, M., Stopkova, P., & Höschl, C. (2015). The effectiveness of prefrontal theta cordance and early reduction of depressive symptoms in the prediction of antidepressant treatment outcome in patients with resistant depression: analysis of naturalistic data. *European archives of psychiatry and clinical neuroscience*, *265*(1), 73–82. |
| **Bares, 2017** | Bares, M., Novak, T., Brunovsky, M., Kopecek, M., & Höschl, C. (2017). The Comparison of Effectiveness of Various Potential Predictors of Response to Treatment With SSRIs in Patients With Depressive Disorder. *The Journal of nervous and mental disease*, *205*(8), 618–626. |
| **Belzeaux, 2012** | Belzeaux, R., Bergon, A., Jeanjean, V., Loriod, B., Formisano-Tréziny, C., Verrier, L., Loundou, A., Baumstarck-Barrau, K., Boyer, L., Gall, V., Gabert, J., Nguyen, C., Azorin, J. M., Naudin, J., & Ibrahim, E. C. (2012). Responder and nonresponder patients exhibit different peripheral transcriptional signatures during major depressive episode. *Translational psychiatry*, *2*(11), e185. |
| **Berends, 2016** | Berends, T., van Meijel, B., Nugteren, W., Deen, M., Danner, U. N., Hoek, H. W., & van Elburg, A. A. (2016). Rate, timing and predictors of relapse in patients with anorexia nervosa following a relapse prevention program: a cohort study. *BMC psychiatry*, *16*(1), 316. |
| **Berger, 2004** | Berger, P., Sachs, G., Amering, M., Holzinger, A., Bankier, B., & Katschnig, H. (2004). Personality disorder and social anxiety predict delayed response in drug and behavioral treatment of panic disorder. *Journal of affective disorders*, *80*(1), 75–78. |
| **Berking, 2011** | Berking, M., Margraf, M., Ebert, D., Wupperman, P., Hofmann, S. G., & Junghanns, K. (2011). Deficits in emotion-regulation skills predict alcohol use during and after cognitive-behavioral therapy for alcohol dependence. *Journal of consulting and clinical psychology*, *79*(3), 307–318. |
| **Berlin, 1998** | Berlin, I., & Lavergne, F. (1998). Early predictors of two month response with mianserin and selective serotonin reuptake inhibitors and influence of definition of outcome on prediction. *European psychiatry : the journal of the Association of European Psychiatrists*, *13*(3), 138–142. |
| **Bhattacharyya, 2021** | Bhattacharyya, S., Schoeler, T., Patel, R., di Forti, M., Murray, R. M., & McGuire, P. (2021). Individualized prediction of 2-year risk of relapse as indexed by psychiatric hospitalization following psychosis onset: Model development in two first episode samples. *Schizophrenia research*, *228*, 483–492. |
| **Birkenhager, 2010** | Birkenhäger, T. K., Pluijms, E. M., Ju, M. R., Mulder, P. G., & den Broek, W. W. (2010). Influence of age on the efficacy of electroconvulsive therapy in major depression: a retrospective study. *Journal of affective disorders*, *126*(1-2), 257–261. |
| **Birmaher,2009** | Birmaher, B., Axelson, D., Goldstein, B., Strober, M., Gill, M. K., Hunt, J., Houck, P., Ha, W., Iyengar, S., Kim, E., Yen, S., Hower, H., Esposito-Smythers, C., Goldstein, T., Ryan, N., & Keller, M. (2009). Four-year longitudinal course of children and adolescents with bipolar spectrum disorders: the Course and Outcome of Bipolar Youth (COBY) study. *The American journal of psychiatry*, *166*(7), 795–804. |
| **Birmaher, 2020** | Birmaher, B., Merranko, J. A., Gill, M. K., Hafeman, D., Goldstein, T., Goldstein, B., Hower, H., Strober, M., Axelson, D., Ryan, N., Yen, S., Diler, R., Iyengar, S., Kattan, M. W., Weinstock, L., & Keller, M. (2020). Predicting Personalized Risk of Mood Recurrences in Youths and Young Adults With Bipolar Spectrum Disorder. *Journal of the American Academy of Child and Adolescent Psychiatry*, *59*(10), 1156–1164. |
| **Birmaher, 2000** | Birmaher, B., Brent, D. A., Kolko, D., Baugher, M., Bridge, J., Holder, D., Iyengar, S., & Ulloa, R. E. (2000). Clinical outcome after short-term psychotherapy for adolescents with major depressive disorder. *Archives of general psychiatry*, *57*(1), 29–36. |
| **Bischof, 2001** | Bischof, G., Rumpf, H. J., Hapke, U., Meyer, C., & John, U. (2001). Factors influencing remission from alcohol dependence without formal help in a representative population sample. *Addiction (Abingdon, England)*, *96*(9), 1327–1336. |
| **Black, 1994** | Black, D. W., Wesner, R. B., Gabel, J., Bowers, W., & Monahan, P. (1994). Predictors of short-term treatment response in 66 patients with panic disorder. *Journal of affective disorders*, *30*(4), 233–241. |
| **Blom, 2007** | Blom, M. B., Spinhoven, P., Hoffman, T., Jonker, K., Hoencamp, E., Haffmans, P. M., & van Dyck, R. (2007). Severity and duration of depression, not personality factors, predict short term outcome in the treatment of major depression. *Journal of affective disorders*, *104*(1-3), 119–126. |
| **Bock, 2009** | Bock, C., Bukh, J. D., Vinberg, M., Gether, U., & Kessing, L. V. (2009). Do stressful life events predict medical treatment outcome in first episode of depression?. *Social psychiatry and psychiatric epidemiology*, *44*(9), 752–760. |
| **Bockting, 2012** | Bockting, C. L., Lok, A., Visser, I., Assies, J., Koeter, M. W., Schene, A. H., & DELTA study group (2012). Lower cortisol levels predict recurrence in remitted patients with recurrent depression: a 5.5 year prospective study. *Psychiatry research*, *200*(2-3), 281–287. |
| **Bokma, 2020** | Bokma, W. A., Zhutovsky, P., Giltay, E. J., Schoevers, R. A., Penninx, B. W. J. H., van Balkom, A. L. J. M., Batelaan, N. M., & van Wingen, G. A. (2022). Predicting the naturalistic course in anxiety disorders using clinical and biological markers: a machine learning approach. *Psychological medicine*, *52*(1), 57–67. |
| **Boschloo, 2012** | Boschloo, L., Vogelzangs, N., van den Brink, W., Smit, J. H., Beekman, A. T., & Penninx, B. W. (2012). Predictors of the 2-year recurrence and persistence of alcohol dependence. *Addiction (Abingdon, England)*, *107*(9), 1639–1640. |
| **Bosworth, 2002** | Hayden B. Bosworth, Douglas R. McQuoid, Linda K. George, David C. Steffens,  Time-to-Remission From Geriatric Depression: Psychosocial and Clinical Factors, The American Journal of Geriatric Psychiatry, Volume 10, Issue 5,  2002, Pages 551-559 |
| **Boter, 2009** | Boter, H., Peuskens, J., Libiger, J., Fleischhacker, W. W., Davidson, M., Galderisi, S., Kahn, R. S., & EUFEST study group (2009). Effectiveness of antipsychotics in first-episode schizophrenia and schizophreniform disorder on response and remission: an open randomized clinical trial (EUFEST). *Schizophrenia research*, *115*(2-3), 97–103. |
| **Bottender, 2002** | Bottlender, R., Sato, T., Jäger, M., Groll, C., Strauss, A., & Möller, H. J. (2002). The impact of duration of untreated psychosis and premorbid functioning on outcome of first inpatient treatment in schizophrenic and schizoaffective patients. *European archives of psychiatry and clinical neuroscience*, *252*(5), 226–231. |
| **Bottlender, 2005** | Bottlender, M., & Soyka, M. (2005). Outpatient alcoholism treatment: predictors of outcome after 3 years. *Drug and alcohol dependence*, *80*(1), 83–89. |
| **Bouhouys, 2006** | Bouhuys, A. L., Bos, E. H., Geerts, E., van Os, T. W., & Ormel, J. (2006). The association between levels of cortisol secretion and fear perception in patients with remitted depression predicts recurrence. *The Journal of nervous and mental disease*, *194*(7), 478–484. |
| **Bouriion-Bedes, 2013** | Bourion-Bedes, S., Baumann, C., Kermarrec, S., Ligier, F., Feillet, F., Bonnemains, C., Guillemin, F., & Kabuth, B. (2013). Prognostic value of early therapeutic alliance in weight recovery: a prospective cohort of 108 adolescents with anorexia nervosa. *The Journal of adolescent health : official publication of the Society for Adolescent Medicine*, *52*(3), 344–350. |
| **Brent, 1998** | Brent, D. A., Kolko, D. J., Birmaher, B., Baugher, M., Bridge, J., Roth, C., & Holder, D. (1998). Predictors of treatment efficacy in a clinical trial of three psychosocial treatments for adolescent depression. *Journal of the American Academy of Child and Adolescent Psychiatry*, *37*(9), 906–914 |
| **Brent, 2009** | Brent, D., Melhem, N., Ferrell, R., Emslie, G., Wagner, K. D., Ryan, N., Vitiello, B., Birmaher, B., Mayes, T., Zelazny, J., Onorato, M., Devlin, B., Clarke, G., DeBar, L., & Keller, M. (2010). Association of FKBP5 polymorphisms with suicidal events in the Treatment of Resistant Depression in Adolescents (TORDIA) study. *The American journal of psychiatry*, *167*(2), 190–197 |
| **Bromet, 2005** | Bromet, E. J., Finch, S. J., Carlson, G. A., Fochtmann, L., Mojtabai, R., Craig, T. J., Kang, S., & Ye, Q. (2005). Time to remission and relapse after the first hospital admission in severe bipolar disorder. *Social psychiatry and psychiatric epidemiology*, *40*(2), 106–113. |
| **Brown, 2000** | Brown, C., Schulberg, H. C., & Prigerson, H. G. (2000). Factors associated with symptomatic improvement and recovery from major depression in primary care patients. *General hospital psychiatry*, *22*(4), 242–250. |
| **Bschor, 2003a** | Bschor, T., Baethge, C., Adli, M., Eichmann, U., Ising, M., Uhr, M., Modell, S., Künzel, H., Müller-Oerlinghausen, B., & Bauer, M. (2003). Association between response to lithium augmentation and the combined DEX/CRH test in major depressive disorder. *Journal of psychiatric research*, *37*(2), 135–143. |
| **Budzyński, 2016** | Budzyński, J., Ziółkowski, M., Kłopocka, M., & Czarnecki, D. (2016). Blood glucose and lipid concentrations after overload are not associated with the risk of alcohol relapse. *Drug and alcohol dependence*, *161*, 356–362. |
| **Camchong, 2013** | Camchong, J., Stenger, A., & Fein, G. (2013). Resting-state synchrony during early alcohol abstinence can predict subsequent relapse. *Cerebral cortex (New York, N.Y. : 1991)*, *23*(9), 2086–2099. |
| **Carter, 2012** | Carter, J. C., Mercer-Lynn, K. B., Norwood, S. J., Bewell-Weiss, C. V., Crosby, R. D., Woodside, D. B., & Olmsted, M. P. (2012). A prospective study of predictors of relapse in anorexia nervosa: implications for relapse prevention. *Psychiatry research*, *200*(2-3), 518–523. |
| **Caseiro, 2012** | Caseiro, O., Pérez-Iglesias, R., Mata, I., Martínez-Garcia, O., Pelayo-Terán, J. M., Tabares-Seisdedos, R., Ortiz-García de la Foz, V., Vázquez-Barquero, J. L., & Crespo-Facorro, B. (2012). Predicting relapse after a first episode of non-affective psychosis: a three-year follow-up study. *Journal of psychiatric research*, *46*(8), 1099–1105. |
| **Castellini, 2011** | Castellini, G., Lo Sauro, C., Mannucci, E., Ravaldi, C., Rotella, C. M., Faravelli, C., & Ricca, V. (2011). Diagnostic crossover and outcome predictors in eating disorders according to DSM-IV and DSM-V proposed criteria: a 6-year follow-up study. *Psychosomatic medicine*, *73*(3), 270–279. |
| **Cattaneo, 2016** | Cattaneo, A., Ferrari, C., Uher, R., Bocchio-Chiavetto, L., Riva, M. A., MRC ImmunoPsychiatry Consortium, & Pariante, C. M. (2016). Absolute Measurements of Macrophage Migration Inhibitory Factor and Interleukin-1-β mRNA Levels Accurately Predict Treatment Response in Depressed Patients. *The international journal of neuropsychopharmacology*, *19*(10), pyw045. |
| **Cearns, 2019** | Cearns, M., Opel, N., Clark, S., Kaehler, C., Thalamuthu, A., Heindel, W., Winter, T., Teismann, H., Minnerup, H., Dannlowski, U., Berger, K., & Baune, B. T. (2019). Predicting rehospitalization within 2 years of initial patient admission for a major depressive episode: a multimodal machine learning approach. *Translational psychiatry*, *9*(1), 285. |
| **Chang, 2006** | Chang, Y. C., Lane, H. Y., Yang, K. H., & Huang, C. L. (2006). Optimizing early prediction for antipsychotic response in schizophrenia. *Journal of clinical psychopharmacology*, *26*(6), 554–559 |
| **Chang, 2012a** | Chang, H. H., Lee, I. H., Gean, P. W., Lee, S. Y., Chi, M. H., Yang, Y. K., Lu, R. B., & Chen, P. S. (2012). Treatment response and cognitive impairment in major depression: association with C-reactive protein. *Brain, behavior, and immunity*, *26*(1), 90–95. |
| **Chang, 2012b** | Chang, W. C., Tang, J. Y., Hui, C. L., Lam, M. M., Chan, S. K., Wong, G. H., Chiu, C. P., & Chen, E. Y. (2012). Prediction of remission and recovery in young people presenting with first-episode psychosis in Hong Kong: a 3-year follow-up study. *The Australian and New Zealand journal of psychiatry*, *46*(2), 100–108 |
| **Chekraud, 2016** | Chekroud, A. M., Zotti, R. J., Shehzad, Z., Gueorguieva, R., Johnson, M. K., Trivedi, M. H., Cannon, T. D., Krystal, J. H., & Corlett, P. R. (2016). Cross-trial prediction of treatment outcome in depression: a machine learning approach. *The lancet. Psychiatry*, *3*(3), 243–250 |
| **Cheng, 2007** | Cheng, I. C., Liao, S. C., Lee, M. B., & Tseng, M. M. (2007). Predictors of treatment response and length of stay for inpatients with major depression. *Journal of the Formosan Medical Association = Taiwan yi zhi*, *106*(11), 903–910 |
| **Chiappetta, 2014** | Chiappetta, V., García-Rodríguez, O., Jin, C. J., Secades-Villa, R., & Blanco, C. (2014). Predictors of quit attempts and successful quit attempts among individuals with alcohol use disorders in a nationally representative sample. *Drug and alcohol dependence*, *141*, 138–144 |
| **Cho, 2019** | Cho, C. H., Lee, T., Kim, M. G., In, H. P., Kim, L., & Lee, H. J. (2019). Addendum to the Acknowledgements: Mood Prediction of Patients With Mood Disorders by Machine Learning Using Passive Digital Phenotypes Based on the Circadian Rhythm: Prospective Observational Cohort Study. *Journal of medical Internet research*, *21*(10), e15966 |
| **Ciudad, 2009** | Ciudad, A., Alvarez, E., Bobes, J., San, L., Polavieja, P., & Gilaberte, I. (2009). Remission in schizophrenia: results from a 1-year follow-up observational study. *Schizophrenia research*, *108*(1-3), 214–222 |
| **Clausen, 2008** | Clausen L. (2008). Time to remission for eating disorder patients: a 2(1/2)-year follow-up study of outcome and predictors. *Nordic journal of psychiatry*, *62*(2), 151–159 |
| **Clery-Melin, 2016** | Cléry-Melin, M. L., & Gorwood, P. (2017). A simple attention test in the acute phase of a major depressive episode is predictive of later functional remission. *Depression and anxiety*, *34*(2), 159–170 |
| **Cohen, 2004** | Cohen, A. N., Hammen, C., Henry, R. M., & Daley, S. E. (2004). Effects of stress and social support on recurrence in bipolar disorder. *Journal of affective disorders*, *82*(1), 143–147 |
| **Connor, 2007** | Connor, J. P., Symons, M., Feeney, G. F., Young, R. M., & Wiles, J. (2007). The application of machine learning techniques as an adjunct to clinical decision making in alcohol dependence treatment. *Substance use & misuse*, *42*(14), 2193–2206 |
| **Contini, 2010** | Contini, V., Victor, M. M., Marques, F. Z., Bertuzzi, G. P., Salgado, C. A., Silva, K. L., Sousa, N. O., Grevet, E. H., Belmonte-de-Abreu, P., & Bau, C. H. (2010). Response to methylphenidate is not influenced by DAT1 polymorphisms in a sample of Brazilian adult patients with ADHD. *Journal of neural transmission (Vienna, Austria : 1996)*, *117*(2), 269–276 |
| **Conus, 2017** | Conus, P., Cotton, S. M., Francey, S. M., O'Donoghue, B., Schimmelmann, B. G., McGorry, P. D., & Lambert, M. (2017). Predictors of favourable outcome in young people with a first episode psychosis without antipsychotic medication. *Schizophrenia research*, *185*, 130–136 |
| **Cooney, 2007** | Cooney, N. L., Litt, M. D., Cooney, J. L., Pilkey, D. T., Steinberg, H. R., & Oncken, C. A. (2007). Alcohol and tobacco cessation in alcohol-dependent smokers: analysis of real-time reports. *Psychology of addictive behaviors : journal of the Society of Psychologists in Addictive Behaviors*, *21*(3), 277–286 |
| **Cottraux, 1993** | Cottraux, J., Messy, P., Marks, I. M., Mollard, E., & Bouvard, M. (1993). Predictive factors in the treatment of obsessive-compulsive disorders with fluvoxamine and/or behaviour therapy. Behavioural Psychotherapy, 21(1), 45–50. |
| **Cougnard, 2006** | Cougnard, A., Parrot, M., Grolleau, S., Kalmi, E., Desage, A., Misdrahi, D., Brun-Rousseau, H., & Verdoux, H. (2006). Pattern of health service utilization and predictors of readmission after a first admission for psychosis: a 2-year follow-up study. *Acta psychiatrica Scandinavica*, *113*(4), 340–349 |
| **Curran, 2000** | Curran, G. M., Flynn, H. A., Kirchner, J., & Booth, B. M. (2000). Depression after alcohol treatment as a risk factor for relapse among male veterans. *Journal of substance abuse treatment*, *19*(3), 259–265 |
| **Damian, 2017** | Damian, A. J., & Mendelson, T. (2017). Association of Physical Activity with Alcohol Abuse and Dependence in a Nationally-Representative U.S. Sample. *Substance use & misuse*, *52*(13), 1744–1750 |
| **Das, 1997** | Das, M. K., Kulhara, P. L., & Verma, S. K. (1997). Life events preceding relapse of schizophrenia. *The International journal of social psychiatry*, *43*(1), 56–63 |
| **Davidson, 2001** | Davidson, J., Pearlstein, T., Londborg, P., Brady, K. T., Rothbaum, B., Bell, J., Maddock, R., Hegel, M. T., & Farfel, G. (2001). Efficacy of sertraline in preventing relapse of posttraumatic stress disorder: results of a 28-week double-blind, placebo-controlled study. *The American journal of psychiatry*, *158*(12), 1974–1981 |
| **Dawson, 2007** | Dawson, D. A., Goldstein, R. B., & Grant, B. F. (2007). Rates and correlates of relapse among individuals in remission from DSM-IV alcohol dependence: a 3-year follow-up. *Alcoholism, clinical and experimental research*, *31*(12), 2036–2045 |
| **Dawson, 2012** | Dawson, D. A., Goldstein, R. B., Ruan, W. J., & Grant, B. F. (2012). Correlates of recovery from alcohol dependence: a prospective study over a 3-year follow-up interval. *Alcoholism, clinical and experimental research*, *36*(7), 1268–1277 |
| **Dazzan, 2019** | Dazzan, P., Lappin, J. M., Heslin, M., Donoghue, K., Lomas, B., Reininghaus, U., Onyejiaka, A., Croudace, T., Jones, P. B., Murray, R. M., Fearon, P., Doody, G. A., & Morgan, C. (2020). Symptom remission at 12-weeks strongly predicts long-term recovery from the first episode of psychosis. *Psychological medicine*, *50*(9), 1452–1462 |
| **De vreede, 2005** | de Vreede, I. M., Burger, H., & van Vliet, I. M. (2005). Prediction of response to ECT with routinely collected data in major depression. *Journal of affective disorders*, *86*(2-3), 323–327 |
| **Degenhardt, 2011** | Degenhardt, E. K., Gatz, J. L., Jacob, J., & Tohen, M. (2012). Predictors of relapse or recurrence in bipolar I disorder. *Journal of affective disorders*, *136*(3), 733–739 |
| **Del bello, 2007** | DelBello, M. P., Hanseman, D., Adler, C. M., Fleck, D. E., & Strakowski, S. M. (2007). Twelve-month outcome of adolescents with bipolar disorder following first hospitalization for a manic or mixed episode. *The American journal of psychiatry*, *164*(4), 582–590 |
| **Denys, 2003** | Denys, D., Burger, H., van Megen, H., de Geus, F., & Westenberg, H. (2003). A score for predicting response to pharmacotherapy in obsessive-compulsive disorder. *International clinical psychopharmacology*, *18*(6), 315–322 |
| **Dijkstra-Kersten et al., 2017** | Dijkstra-Kersten, S. M. A., Sitnikova, K., Terluin, B., Penninx, B. W. J. H., Twisk, J. W. R., van Marwijk, H. W. J., van der Horst, H. E., & van der Wouden, J. C. (2017). Longitudinal associations of multiple physical symptoms with recurrence of depressive and anxiety disorders. *Journal of psychosomatic research*, *97*, 96–101. |
| **Dreifuss, 2013** | Dreifuss, J. A., Griffin, M. L., Frost, K., Fitzmaurice, G. M., Potter, J. S., Fiellin, D. A., Selzer, J., Hatch-Maillette, M., Sonne, S. C., & Weiss, R. D. (2013). Patient characteristics associated with buprenorphine/naloxone treatment outcome for prescription opioid dependence: Results from a multisite study. *Drug and alcohol dependence*, *131*(1-2), 112–118. |
| **Driscoll, 2005** | Driscoll, H. C., Basinski, J., Mulsant, B. H., Butters, M. A., Dew, M. A., Houck, P. R., Mazumdar, S., Miller, M. D., Pollock, B. G., Stack, J. A., Schlernitzauer, M. A., & Reynolds, C. F., 3rd (2005). Late-onset major depression: clinical and treatment-response variability. *International journal of geriatric psychiatry*, *20*(7), 661–667 |
| **Durazzo, 2017 a** | Durazzo, T. C., & Meyerhoff, D. J. (2017). Psychiatric, Demographic, and Brain Morphological Predictors of Relapse After Treatment for an Alcohol Use Disorder. *Alcoholism, clinical and experimental research*, *41*(1), 107–116 |
| **Eberhard, 2009** | Eberhard, J., Levander, S., & Lindström, E. (2009). Remission in schizophrenia: analysis in a naturalistic setting. *Comprehensive psychiatry*, *50*(3), 200–208 |
| **Edgecomb, 2019** | Edgcomb, J., Shaddox, T., Hellemann, G., & Brooks, J. O., 3rd (2019). High-Risk Phenotypes of Early Psychiatric Readmission in Bipolar Disorder With Comorbid Medical Illness. *Psychosomatics*, *60*(6), 563–573 |
| **Emsley, 2006** | Emsley, R., Oosthuizen, P. P., Kidd, M., Koen, L., Niehaus, D. J., & Turner, H. J. (2006). Remission in first-episode psychosis: predictor variables and symptom improvement patterns. *The Journal of clinical psychiatry*, *67*(11), 1707–1712 |
| **Emsley, 2007** | Emsley, R., Rabinowitz, J., Medori, R., & Early Psychosis Global Working Group (2007). Remission in early psychosis: Rates, predictors, and clinical and functional outcome correlates. *Schizophrenia research*, *89*(1-3), 129–139. |
| **Emsley, 2008** | Emsley, R., Oosthuizen, P., Koen, L., Niehaus, D. J., Medori, R., & Rabinowitz, J. (2008). Remission in patients with first-episode schizophrenia receiving assured antipsychotic medication: a study with risperidone long-acting injection. *International clinical psychopharmacology*, *23*(6), 325–331 |
| **Engel, 2016** | Engel, K., Schaefer, M., Stickel, A., Binder, H., Heinz, A., & Richter, C. (2016). The Role of Psychological Distress in Relapse Prevention of Alcohol Addiction. Can High Scores on the SCL-90-R Predict Alcohol Relapse?. *Alcohol and alcoholism (Oxford, Oxfordshire)*, *51*(1), 27–31 |
| **Enns, 2005** | Enns, M. W., & Cox, B. J. (2005). Psychosocial and clinical predictors of symptom persistence vs remission in major depressive disorder. *Canadian journal of psychiatry. Revue canadienne de psychiatrie*, *50*(12), 769–777 |
| **Erguzel, 2015** | Erguzel, T. T., Ozekes, S., Gultekin, S., Tarhan, N., Hizli Sayar, G., & Bayram, A. (2015). Neural Network Based Response Prediction of rTMS in Major Depressive Disorder Using QEEG Cordance. *Psychiatry investigation*, *12*(1), 61–65 |
| **Evren, 2013** | Evren, Cuneyt & Yigiter, Sera & Bozkurt, Müge & Cagil, Dilara & Ozcetinkaya, Serap & Can, Yesim & Mutlu, Elif. (2013). Personality dimensions and defense styles that are related with relapse during 12 month follow-up in male alcohol dependents. Dusunen Adam: The Journal of Psychiatry and Neurological Sciences. 26. 248-257. |
| **Ezquiaga, 2004** | Ezquiaga, E., García-López, A., de Dios, C., Leiva, A., Bravo, M., & Montejo, J. (2004). Clinical and psychosocial factors associated with the outcome of unipolar major depression: a one year prospective study. *Journal of affective disorders*, *79*(1-3), 63–70 |
| **Farren, 2010** | Farren, C. K., & McElroy, S. (2010). Predictive factors for relapse after an integrated inpatient treatment programme for unipolar depressed and bipolar alcoholics. *Alcohol and alcoholism (Oxford, Oxfordshire)*, *45*(6), 527–533 |
| **Fava, 2008** | Fava, M., Rush, A. J., Alpert, J. E., Balasubramani, G. K., Wisniewski, S. R., Carmin, C. N., Biggs, M. M., Zisook, S., Leuchter, A., Howland, R., Warden, D., & Trivedi, M. H. (2008). Difference in treatment outcome in outpatients with anxious versus nonanxious depression: a STAR*D report. *The American journal of psychiatry*, *165*(3), 342–351 |
| **Fernandez-Aranda, 2009** | Fernández-Aranda, F., Núñez, A., Martínez, C., Krug, I., Cappozzo, M., Carrard, I., Rouget, P., Jiménez-Murcia, S., Granero, R., Penelo, E., Santamaría, J., & Lam, T. (2009). Internet-based cognitive-behavioral therapy for bulimia nervosa: a controlled study. *Cyberpsychology & behavior : the impact of the Internet, multimedia and virtual reality on behavior and society*, *12*(1), 37–41 |
| **Fichter, 2006** | Fichter, M. M., Quadflieg, N., & Hedlund, S. (2006). Twelve-year course and outcome predictors of anorexia nervosa. *The International journal of eating disorders*, *39*(2), 87–100 |
| **Fichter, 2017** | Fichter, M. M., Quadflieg, N., Crosby, R. D., & Koch, S. (2017). Long-term outcome of anorexia nervosa: Results from a large clinical longitudinal study. *The International journal of eating disorders*, *50*(9), 1018–1030 |
| **Fink, 2007** | Fink, M., Rush, A. J., Knapp, R., Rasmussen, K., Mueller, M., Rummans, T. A., O'Connor, K., Husain, M., Biggs, M., Bailine, S., Kellner, C. H., & Consortium for Research in ECT (CORE) Study Group (2007). DSM melancholic features are unreliable predictors of ECT response: a CORE publication. *The journal of ECT*, *23*(3), 139–146 |
| **Finn, 2012** | Finn, Michael & Robinson, Elizabeth. (2012). Personality and Drinking Behavior in Alcohol Dependence: A Survival Analysis. Alcoholism Treatment Quarterly. 30. 146-162. |
| **Fisher 2017** | Fischer, L. K., Schreyer, C. C., Coughlin, J. W., Redgrave, G. W., & Guarda, A. S. (2017). Neuroticism and clinical course of weight restoration in a meal-based, rapid-weight gain, inpatient-partial hospitalization program for eating disorders. *Eating disorders*, *25*(1), 52–64 |
| **Flickt, 2006** | Flyckt, L., Mattsson, M., Edman, G., Carlsson, R., & Cullberg, J. (2006). Predicting 5-year outcome in first-episode psychosis: construction of a prognostic rating scale. *The Journal of clinical psychiatry*, *67*(6), 916–924 |
| **Flygare, 2020** | Flygare, O., Enander, J., Andersson, E., Ljótsson, B., Ivanov, V. Z., Mataix-Cols, D., & Rück, C. (2020). Predictors of remission from body dysmorphic disorder after internet-delivered cognitive behavior therapy: a machine learning approach. *BMC psychiatry*, *20*(1), 247 |
| **Fond, 2019** | Fond, G., Bulzacka, E., Boucekine, M., Schürhoff, F., Berna, F., Godin, O., Aouizerate, B., Capdevielle, D., Chereau, I., D'Amato, T., Dubertret, C., Dubreucq, J., Faget, C., Leignier, S., Lançon, C., Mallet, J., Misdrahi, D., Passerieux, C., Rey, R., Schandrin, A., … Llorca, P. M. (2019). Machine learning for predicting psychotic relapse at 2 years in schizophrenia in the national FACE-SZ cohort. *Progress in neuro-psychopharmacology & biological psychiatry*, *92*, 8–18 |
| **Forand, 2013** | Forand, N. R., & Derubeis, R. J. (2013). Pretreatment anxiety predicts patterns of change in cognitive behavioral therapy and medications for depression. *Journal of consulting and clinical psychology*, *81*(5), 774–782 |
| **Forsberg, 2003** | Forsberg, S., LoTempio, E., Bryson, S., Fitzpatrick, K. K., Le Grange, D., & Lock, J. (2013). Therapeutic alliance in two treatments for adolescent anorexia nervosa. *The International journal of eating disorders*, *46*(1), 34–38 |
| **Forsberg, 2014** | Forsberg, S., LoTempio, E., Bryson, S., Fitzpatrick, K. K., Le Grange, D., & Lock, J. (2014). Parent-therapist alliance in Family-Based Treatment for adolescents with anorexia nervosa. *European eating disorders review : the journal of the Eating Disorders Association*, *22*(1), 53–58 |
| **Fraguas, 2014** | Fraguas, D., Del Rey-Mejías, A., Moreno, C., Castro-Fornieles, J., Graell, M., Otero, S., Gonzalez-Pinto, A., Moreno, D., Baeza, I., Martínez-Cengotitabengoa, M., Arango, C., & Parellada, M. (2014). Duration of untreated psychosis predicts functional and clinical outcome in children and adolescents with first-episode psychosis: a 2-year longitudinal study. *Schizophrenia research*, *152*(1), 130–138 |
| **Furukawa, 2019** | Furukawa, T. A., Kato, T., Shinagawa, Y., Miki, K., Fujita, H., Tsujino, N., Kondo, M., Inagaki, M., & Yamada, M. (2019). Prediction of remission in pharmacotherapy of untreated major depression: development and validation of multivariable prediction models. *Psychological medicine*, *49*(14), 2405–2413 |
| **Gaebel, 2007** | Gaebel, W., & Riesbeck, M. (2007). Revisiting the relapse predictive validity of prodromal symptoms in schizophrenia. *Schizophrenia research*, *95*(1-3), 19–29 |
| **Gaebel, 2014** | Gaebel, W., Riesbeck, M., Wölwer, W., Klimke, A., Eickhoff, M., von Wilmsdorff, M., Heuser, I., Maier, W., Klosterkötter, J., Falkai, P., Schlösser, R., Schmitt, A., Riedel, M., Klingberg, S., Köpcke, W., Ohmann, C., & Möller, H. J. (2014). Rates and predictors of remission in first-episode schizophrenia within 1 year of antipsychotic maintenance treatment. Results of a randomized controlled trial within the German Research Network on Schizophrenia. *Schizophrenia research*, *152*(2-3), 478–486 |
| **Gaebel, 2016** | Gaebel, W., Riesbeck, M., Wölwer, W., Klimke, A., Eickhoff, M., von Wilmsdorff, M., de Millas, W., Maier, W., Ruhrmann, S., Falkai, P., Sauer, H., Schmitt, A., Riedel, M., Klingberg, S., & Möller, H. J. (2016). Predictors for symptom re-exacerbation after targeted stepwise drug discontinuation in first-episode schizophrenia: Results of the first-episode study within the German research network on schizophrenia. *Schizophrenia research*, *170*(1), 168–176 |
| **Garbusow, 2016** | Garbusow, M., Schad, D. J., Sebold, M., Friedel, E., Bernhardt, N., Koch, S. P., Steinacher, B., Kathmann, N., Geurts, D. E., Sommer, C., Müller, D. K., Nebe, S., Paul, S., Wittchen, H. U., Zimmermann, U. S., Walter, H., Smolka, M. N., Sterzer, P., Rapp, M. A., Huys, Q. J., … Heinz, A. (2016). Pavlovian-to-instrumental transfer effects in the nucleus accumbens relate to relapse in alcohol dependence. *Addiction biology*, *21*(3), 719–731 |
| **Garland, 2012** | Garland, E. L., Franken, I. H., & Howard, M. O. (2012). Cue-elicited heart rate variability and attentional bias predict alcohol relapse following treatment. *Psychopharmacology*, *222*(1), 17–26 |
| **Gebara, 2018** | Gebara, M. A., DiNapoli, E. A., Kasckow, J., Karp, J. F., Blumberger, D. M., Lenze, E. J., Mulsant, B. H., & Reynolds, C. F., 3rd (2018). Specific depressive symptoms predict remission to aripiprazole augmentation in late-life treatment resistant depression. *International journal of geriatric psychiatry*, *33*(2), e330–e335 |
| **Geller, 2002** | Geller, B., Craney, J. L., Bolhofner, K., Nickelsburg, M. J., Williams, M., & Zimerman, B. (2002). Two-year prospective follow-up of children with a prepubertal and early adolescent bipolar disorder phenotype. *The American journal of psychiatry*, *159*(6), 927–933 |
| **Geller, 2004** | Geller, B., Tillman, R., Craney, J. L., & Bolhofner, K. (2004). Four-year prospective outcome and natural history of mania in children with a prepubertal and early adolescent bipolar disorder phenotype. *Archives of general psychiatry*, *61*(5), 459–467 |
| **George, 1989** | George, L. K., Blazer, D. G., Hughes, D. C., & Fowler, N. (1989). Social support and the outcome of major depression. *The British journal of psychiatry : the journal of mental science*, *154*, 478–485 |
| **Gex-Fabry, 2008** | Gex-Fabry, M., Eap, C. B., Oneda, B., Gervasoni, N., Aubry, J. M., Bondolfi, G., & Bertschy, G. (2008). CYP2D6 and ABCB1 genetic variability: influence on paroxetine plasma level and therapeutic response. *Therapeutic drug monitoring*, *30*(4), 474–482 |
| **Ghesquiere, 2014** | Ghesquiere, A. R., Park, M., Bogner, H. R., Greenberg, R. L., & Bruce, M. L. (2014). The effect of recent bereavement on outcomes in a primary care depression intervention study. *The American journal of geriatric psychiatry : official journal of the American Association for Geriatric Psychiatry*, *22*(12), 1555–1564 |
| **Gilder, 2008** | Gilder, D. A., Lau, P., Corey, L., & Ehlers, C. L. (2008). Factors associated with remission from alcohol dependence in an American Indian community group. *The American journal of psychiatry*, *165*(9), 1172–1178 |
| **Ginsburg, 2014** | Ginsburg, G. S., Becker, E. M., Keeton, C. P., Sakolsky, D., Piacentini, J., Albano, A. M., Compton, S. N., Iyengar, S., Sullivan, K., Caporino, N., Peris, T., Birmaher, B., Rynn, M., March, J., & Kendall, P. C. (2014). Naturalistic follow-up of youths treated for pediatric anxiety disorders. *JAMA psychiatry*, *71*(3), 310–318 |
| **Gish, 2001** | Gish, R. G., Lee, A., Brooks, L., Leung, J., Lau, J. Y., & Moore, D. H., 2nd (2001). Long-term follow-up of patients diagnosed with alcohol dependence or alcohol abuse who were evaluated for liver transplantation. *Liver transplantation : official publication of the American Association for the Study of Liver Diseases and the International Liver Transplantation Society*, *7*(7), 581–587 |
| **Gollan, 2006** | Gollan JK, Hoxha D, Hunnicutt-Ferguson K, Norris CJ, Rosebrock L, Sankin L, Cacioppo J. The negativity bias predicts response rate to Behavioral Activation for depression. J Behav Ther Exp Psychiatry. 2016 Sep;52:171-178 |
| **Gordon, 2015** | Gordon, E., Rush, A. J., Palmer, D. M., Braund, T. A., & Rekshan, W. (2015). Toward an online cognitive and emotional battery to predict treatment remission in depression. *Neuropsychiatric disease and treatment*, *11*, 517–531 |
| **Greenfield. 2000** | Greenfield, S. F., Hufford, M. R., Vagge, L. M., Muenz, L. R., Costello, M. E., & Weiss, R. D. (2000). The relationship of self-efficacy expectancies to relapse among alcohol dependent men and women: a prospective study. *Journal of studies on alcohol*, *61*(2), 345–351 |
| **Greenfield, 2002** | Greenfield, S. F., Kolodziej, M. E., Sugarman, D. E., Muenz, L. R., Vagge, L. M., He, D. Y., & Weiss, R. D. (2002). History of abuse and drinking outcomes following inpatient alcohol treatment: a prospective study. *Drug and alcohol dependence*, *67*(3), 227–234 |
| **Greenfield, 2003** | Greenfield, S. F., Sugarman, D. E., Muenz, L. R., Patterson, M. D., He, D. Y., & Weiss, R. D. (2003). The relationship between educational attainment and relapse among alcohol-dependent men and women: a prospective study. *Alcoholism, clinical and experimental research*, *27*(8), 1278–1285 |
| **Greenfield, 1998** | Greenfield, S. F., Weiss, R. D., Muenz, L. R., Vagge, L. M., Kelly, J. F., Bello, L. R., & Michael, J. (1998). The effect of depression on return to drinking: a prospective study. *Archives of general psychiatry*, *55*(3), 259–265 |
| **Greenlee, 2010** | Greenlee, A., Karp, J. F., Dew, M. A., Houck, P., Andreescu, C., & Reynolds, C. F., 3rd (2010). Anxiety impairs depression remission in partial responders during extended treatment in late-life. *Depression and anxiety*, *27*(5), 451–456 |
| **Grillo, 2006** | Grilo, C. M., Masheb, R. M., & Wilson, G. T. (2006). Rapid response to treatment for binge eating disorder. *Journal of consulting and clinical psychology*, *74*(3), 602–613 |
| **Gudayol-Ferre, 2010** | Gudayol-Ferré, E., Herrera-Guzmán, I., Camarena, B., Cortés-Penagos, C., Herrera-Abarca, J. E., Martínez-Medina, P., Cruz, D., Hernández, S., Genis, A., Carrillo-Guerrero, M. Y., Avilés Reyes, R., & Guàrdia-Olmos, J. (2010). The role of clinical variables, neuropsychological performance and SLC6A4 and COMT gene polymorphisms on the prediction of early response to fluoxetine in major depressive disorder. *Journal of affective disorders*, *127*(1-3), 343–351 |
| **Gunning-Dixon, 2010** | Gunning-Dixon, F. M., Walton, M., Cheng, J., Acuna, J., Klimstra, S., Zimmerman, M. E., Brickman, A. M., Hoptman, M. J., Young, R. C., & Alexopoulos, G. S. (2010). MRI signal hyperintensities and treatment remission of geriatric depression. *Journal of affective disorders*, *126*(3), 395–401 |
| **Gyani, 2013** | Gyani, A., Shafran, R., Layard, R., & Clark, D. M. (2013). Enhancing recovery rates: lessons from year one of IAPT. *Behaviour research and therapy*, *51*(9), 597–606 |
| **Hahn, 2015** | Hahn, T., Kircher, T., Straube, B., Wittchen, H. U., Konrad, C., Ströhle, A., Wittmann, A., Pfleiderer, B., Reif, A., Arolt, V., & Lueken, U. (2015). Predicting treatment response to cognitive behavioral therapy in panic disorder with agoraphobia by integrating local neural information. *JAMA psychiatry*, *72*(1), 68–74 |
| **Haller, 2014** | Haller, M., Wang, F. L., Bountress, K., & Chassin, L. (2014). The Interactive Effects of Effort to Regulate Alcohol Use, Anxiety Disorders, and Affective Disorders on Long-Term Remission from Alcohol Dependence. *Addiction research & theory*, *22*(5), 371–379 |
| **Hardeveld, 2014** | Hardeveld, F., Spijker, J., Vreeburg, S. A., Graaf, R. D., Hendriks, S. M., Licht, C. M., Nolen, W. A., Penninx, B. W., & Beekman, A. T. (2014). Increased cortisol awakening response was associated with time to recurrence of major depressive disorder. *Psychoneuroendocrinology*, *50*, 62–71 |
| **Hardeveld, 2013a** | Hardeveld, F., Spijker, J., De Graaf, R., Nolen, W. A., & Beekman, A. T. (2013). Recurrence of major depressive disorder and its predictors in the general population: results from the Netherlands Mental Health Survey and Incidence Study (NEMESIS). *Psychological medicine*, *43*(1), 39–48 |
| **Hardeveld., 2013b** | Hardeveld, F., Spijker, J., De Graaf, R., Hendriks, S. M., Licht, C. M., Nolen, W. A., Penninx, B. W., & Beekman, A. T. (2013). Recurrence of major depressive disorder across different treatment settings: results from the NESDA study. *Journal of affective disorders*, *147*(1-3), 225–231 |
| **Hardeveld, 2015** | Hardeveld, F., Spijker, J., Peyrot, W. J., de Graaf, R., Hendriks, S. M., Nolen, W. A., Penninx, B. W., & Beekman, A. T. (2015). Glucocorticoid and mineralocorticoid receptor polymorphisms and recurrence of major depressive disorder. *Psychoneuroendocrinology*, *55*, 154–163 |
| **Hariman, 2020** | Hariman, K., Cheng, K. M., Lam, J., Leung, S. K., & Lui, S. S. Y. (2020). Clinical risk model to predict 28-day unplanned readmission via the accident and emergency department after discharge from acute psychiatric units for patients with psychotic spectrum disorders. *BJPsych open*, *6*(1), e13 |
| **Harkness, 2012** | Harkness, K. L., Bagby, R. M., & Kennedy, S. H. (2012). Childhood maltreatment and differential treatment response and recurrence in adult major depressive disorder. *Journal of consulting and clinical psychology*, *80*(3), 342–353 |
| **Harkness, 2014** | Harkness, K. L., Theriault, J. E., Stewart, J. G., & Bagby, R. M. (2014). Acute and chronic stress exposure predicts 1-year recurrence in adult outpatients with residual depression symptoms following response to treatment. *Depression and anxiety*, *31*(1), 1–8 |
| **Hartmann, 2010** | Hartmann, A., Orlinsky, D., Weber, S., Sandholz, A., & Zeeck, A. (2010). Session and intersession experience related to treatment outcome in bulimia nervosa. *Psychotherapy (Chicago, Ill.)*, *47*(3), 355–370 |
| **Hasin, 2002** | Hasin, D., Liu, X., Nunes, E., McCloud, S., Samet, S., & Endicott, J. (2002). Effects of major depression on remission and relapse of substance dependence. *Archives of general psychiatry*, *59*(4), 375–380 |
| **Hassan 2011** | Hassan, G. A., & Taha, G. R. (2011). Long term functioning in early onset psychosis: two years prospective follow-up study. *Behavioral and brain functions : BBF*, *7*, 28 |
| **Hatzinger, 2002** | Hatzinger, M., Hemmeter, U. M., Baumann, K., Brand, S., & Holsboer-Trachsler, E. (2002). The combined DEX-CRH test in treatment course and long-term outcome of major depression. *Journal of psychiatric research*, *36*(5), 287–297 |
| **Heffner, 2011** | Heffner, J. L., Blom, T. J., & Anthenelli, R. M. (2011). Gender differences in trauma history and symptoms as predictors of relapse to alcohol and drug use. *The American journal on addictions*, *20*(4), 307–311 |
| **Hegelstad, 2012** | Hegelstad, W. T., Larsen, T. K., Auestad, B., Evensen, J., Haahr, U., Joa, I., Johannesen, J. O., Langeveld, J., Melle, I., Opjordsmoen, S., Rossberg, J. I., Rund, B. R., Simonsen, E., Sundet, K., Vaglum, P., Friis, S., & McGlashan, T. (2012). Long-term follow-up of the TIPS early detection in psychosis study: effects on 10-year outcome. *The American journal of psychiatry*, *169*(4), 374–380 |
| **Heldt, 2009** | Heldt, E., Kipper, L., Blaya, C., Salum, G. A., Hirakata, V. N., Otto, M. W., & Manfro, G. G. (2011). Predictors of relapse in the second follow-up year post cognitive-behavior therapy for panic disorder. *Revista brasileira de psiquiatria (Sao Paulo, Brazil : 1999)*, *33*(1), 23–29 |
| **Herman, 2002** | Herman, Steve & Blumenthal, James & Babyak, Michael & Khatri, Parinda & Craighead, W & Krishnan, K & Doraiswamy, P. (2002). Exercise Therapy for Depression in Middle-Aged and Older Adults: Predictors of Early Dropout and Treatment Failure. Health psychology : official journal of the Division of Health Psychology, American Psychological Association. 21. 553-63 |
| **Herrera-Guzman, 2008** | Herrera-Guzmán, I., Gudayol-Ferré, E., Lira-Mandujano, J., Herrera-Abarca, J., Herrera-Guzmán, D., Montoya-Pérez, K., & Guardia-Olmos, J. (2008). Cognitive predictors of treatment response to bupropion and cognitive effects of bupropion in patients with major depressive disorder. *Psychiatry research*, *160*(1), 72–82 |
| **Herzog, 1999** | Herzog, D. B., Dorer, D. J., Keel, P. K., Selwyn, S. E., Ekeblad, E. R., Flores, A. T., Greenwood, D. N., Burwell, R. A., & Keller, M. B. (1999). Recovery and relapse in anorexia and bulimia nervosa: a 7.5-year follow-up study. *Journal of the American Academy of Child and Adolescent Psychiatry*, *38*(7), 829–837 |
| **Hilbert, 2020** | Hilbert, K., Kunas, S. L., Lueken, U., Kathmann, N., Fydrich, T., & Fehm, L. (2020). Predicting cognitive behavioral therapy outcome in the outpatient sector based on clinical routine data: A machine learning approach. *Behaviour research and therapy*, *124*, 103530 |
| **Hirschfeld, 1998** | Hirschfeld, R. M., Russell, J. M., Delgado, P. L., Fawcett, J., Friedman, R. A., Harrison, W. M., Koran, L. M., Miller, I. W., Thase, M. E., Howland, R. H., Connolly, M. A., & Miceli, R. J. (1998). Predictors of response to acute treatment of chronic and double depression with sertraline or imipramine. *The Journal of clinical psychiatry*, *59*(12), 669–675 |
| **Holvast , 2015** | Holvast, F., Burger, H., de Waal, M. M., van Marwijk, H. W., Comijs, H. C., & Verhaak, P. F. (2015). Loneliness is associated with poor prognosis in late-life depression: Longitudinal analysis of the Netherlands study of depression in older persons. *Journal of affective disorders*, *185*, 1–7 |
| **Hong, 2005** | Hong, C. J., Chen, T. J., Yu, Y. W., & Tsai, S. J. (2006). Response to fluoxetine and serotonin 1A receptor (C-1019G) polymorphism in Taiwan Chinese major depressive disorder. *The pharmacogenomics journal*, *6*(1), 27–33 |
| **Horstmann, 2010** | Horstmann, S., Lucae, S., Menke, A., Hennings, J. M., Ising, M., Roeske, D., Müller-Myhsok, B., Holsboer, F., & Binder, E. B. (2010). Polymorphisms in GRIK4, HTR2A, and FKBP5 show interactive effects in predicting remission to antidepressant treatment. *Neuropsychopharmacology : official publication of the American College of Neuropsychopharmacology*, *35*(3), 727–740 |
| **Hsieh M.H., 2002** | Hsieh, M. H., McQuoid, D. R., Levy, R. M., Payne, M. E., MacFall, J. R., & Steffens, D. C. (2002). Hippocampal volume and antidepressant response in geriatric depression. *International journal of geriatric psychiatry*, *17*(6), 519–525 |
| **Huang, 2014** | Huang, S. H., LePendu, P., Iyer, S. V., Tai-Seale, M., Carrell, D., & Shah, N. H. (2014). Toward personalizing treatment for depression: predicting diagnosis and severity. *Journal of the American Medical Informatics Association : JAMIA*, *21*(6), 1069–1075 |
| **Hudson, 2013** | Hudson, J. L., Lester, K. J., Lewis, C. M., Tropeano, M., Creswell, C., Collier, D. A., Cooper, P., Lyneham, H. J., Morris, T., Rapee, R. M., Roberts, S., Donald, J. A., & Eley, T. C. (2013). Predicting outcomes following cognitive behaviour therapy in child anxiety disorders: the influence of genetic, demographic and clinical information. *Journal of child psychology and psychiatry, and allied disciplines*, *54*(10), 1086–1094 |
| **Hufnagel, 2017** | Hufnagel, A., Frick, U., Ridinger, M., & Wodarz, N. (2017). Recovery from alcohol dependence: Do smoking indicators predict abstinence?. *The American journal on addictions*, *26*(4), 366–373 |
| **Hui, 2013a** | Hui, C. L., Tang, J. Y., Leung, C. M., Wong, G. H., Chang, W. C., Chan, S. K., Lee, E. H., & Chen, E. Y. (2013). A 3-year retrospective cohort study of predictors of relapse in first-episode psychosis in Hong Kong. *The Australian and New Zealand journal of psychiatry*, *47*(8), 746–753 |
| **Hui, 2013b** | Hui, C. L., Wong, G. H., Tang, J. Y., Chang, W. C., Chan, S. K., Lee, E. H., Lam, M. M., Chiu, C. P., Law, C. W., Chung, D. W., Tso, S., Pang, E. P., Chan, K. T., Wong, Y. C., Mo, F. Y., Chan, K. P., Hung, S. F., Honer, W. G., & Chen, E. Y. (2013). Predicting 1-year risk for relapse in patients who have discontinued or continued quetiapine after remission from first-episode psychosis. *Schizophrenia research*, *150*(1), 297–302 |
| **Hui, 2016** | Hui, C. L., Li, Y. K., Li, A. W., Lee, E. H., Chang, W. C., Chan, S. K., Lam, S. Y., Thornton, A. E., Sham, P., Honer, W. G., & Chen, E. Y. (2016). Visual working memory deterioration preceding relapse in psychosis. *Psychological medicine*, *46*(11), 2435–2444 |
| **Iniesta, 2016** | Iniesta, R., Malki, K., Maier, W., Rietschel, M., Mors, O., Hauser, J., Henigsberg, N., Dernovsek, M. Z., Souery, D., Stahl, D., Dobson, R., Aitchison, K. J., Farmer, A., Lewis, C. M., McGuffin, P., & Uher, R. (2016). Combining clinical variables to optimize prediction of antidepressant treatment outcomes. *Journal of psychiatric research*, *78*, 94–102 |
| **Iniesta, 2018** | Iniesta, R., Hodgson, K., Stahl, D., Malki, K., Maier, W., Rietschel, M., Mors, O., Hauser, J., Henigsberg, N., Dernovsek, M. Z., Souery, D., Dobson, R., Aitchison, K. J., Farmer, A., McGuffin, P., Lewis, C. M., & Uher, R. (2018). Antidepressant drug-specific prediction of depression treatment outcomes from genetic and clinical variables. *Scientific reports*, *8*(1), 5530 |
| **Iosifescu, 2005** | Iosifescu, D. V., Clementi-Craven, N., Fraguas, R., Papakostas, G. I., Petersen, T., Alpert, J. E., Nierenberg, A. A., & Fava, M. (2005). Cardiovascular risk factors may moderate pharmacological treatment effects in major depressive disorder. *Psychosomatic medicine*, *67*(5), 703–706 |
| **Iosifescu, 2006** | Iosifescu, D. V., Renshaw, P. F., Lyoo, I. K., Lee, H. K., Perlis, R. H., Papakostas, G. I., Nierenberg, A. A., & Fava, M. (2006). Brain white-matter hyperintensities and treatment outcome in major depressive disorder. *The British journal of psychiatry : the journal of mental science*, *188*, 180–185 |
| **Jain, 2013** | Jain, F. A., Hunter, A. M., Brooks, J. O., 3rd, & Leuchter, A. F. (2013). Predictive socioeconomic and clinical profiles of antidepressant response and remission. *Depression and anxiety*, *30*(7), 624–630 |
| **Jakubczyk, 2013** | Jakubczyk A, Klimkiewicz A, Kopera M, Krasowska A, Wrzosek M, Matsumoto H, Burmeister M, Brower KJ, Wojnar M. The CC genotype in the T102C HTR2A polymorphism predicts relapse in individuals after alcohol treatment. J Psychiatr Res. 2013 Apr;47(4):527-33 |
| **Jakubczyk, 2016** | Jakubczyk, A., Ilgen, M. A., Kopera, M., Krasowska, A., Klimkiewicz, A., Bohnert, A., Blow, F. C., Brower, K. J., & Wojnar, M. (2016). Reductions in physical pain predict lower risk of relapse following alcohol treatment. *Drug and alcohol dependence*, *158*, 167–171 |
| **Jakubovski, 2016** | Jakubovski, E., & Bloch, M. H. (2016). Anxiety Disorder-Specific Predictors of Treatment Outcome in the Coordinated Anxiety Learning and Management (CALM) Trial. *The Psychiatric quarterly*, *87*(3), 445–464 |
| **Janssen, 2007** | Janssen, J., Hulshoff Pol, H. E., Schnack, H. G., Kok, R. M., Lampe, I. K., de Leeuw, F. E., Kahn, R. S., & Heeren, T. J. (2007). Cerebral volume measurements and subcortical white matter lesions and short-term treatment response in late life depression. *International journal of geriatric psychiatry*, *22*(5), 468–474 |
| **Jarbin, 2000** | Håkan Jarbin, Rolf W Gråwe, Kjell Hansson (2000) Expressed emotion and prediction of relapse in adolescents with psychotic disorders, Nordic Journal of Psychiatry, 54:3, 201-205 |
| **Jarrett, 2000** | Jarrett, R. B., Kraft, D., Doyle, J., Foster, B. M., Eaves, G. G., & Silver, P. C. (2001). Preventing recurrent depression using cognitive therapy with and without a continuation phase: a randomized clinical trial. *Archives of general psychiatry*, *58*(4), 381–388 |
| **Jiang, 2018** | Jiang, W., Whellan, D. J., Adams, K. F., Babyak, M. A., Boyle, S. H., Wilson, J. L., Patel, C. B., Rogers, J. G., Harris, W. S., & O'Connor, C. M. (2018). Long-Chain Omega-3 Fatty Acid Supplements in Depressed Heart Failure Patients: Results of the OCEAN Trial. *JACC. Heart failure*, *6*(10), 833–843 |
| **Jin, 1998** | Jin, H., Rourke, S. B., Patterson, T. L., Taylor, M. J., & Grant, I. (1998). Predictors of relapse in long-term abstinent alcoholics. *Journal of studies on alcohol*, *59*(6), 640–646 |
| **Joel, 2014** | Joel, I., Begley, A. E., Mulsant, B. H., Lenze, E. J., Mazumdar, S., Dew, M. A., Blumberger, D., Butters, M., Reynolds, C. F., 3rd, & IRL GREY Investigative Team (2014). Dynamic prediction of treatment response in late-life depression. *The American journal of geriatric psychiatry : official journal of the American Association for Geriatric Psychiatry*, *22*(2), 167–176 |
| **Johnson, 2003** | Johnson, L., Lundström, O., Aberg-Wistedt, A., & Mathé, A. A. (2003). Social support in bipolar disorder: its relevance to remission and relapse. *Bipolar disorders*, *5*(2), 129–137 |
| **Jorge, 2008** | Jorge, R. E., Moser, D. J., Acion, L., & Robinson, R. G. (2008). Treatment of vascular depression using repetitive transcranial magnetic stimulation. *Archives of general psychiatry*, *65*(3), 268–276 |
| **Kalaydjian, 2009** | Kalaydjian, A., Swendsen, J., Chiu, W. T., Dierker, L., Degenhardt, L., Glantz, M., Merikangas, K. R., Sampson, N., & Kessler, R. (2009). Sociodemographic predictors of transitions across stages of alcohol use, disorders, and remission in the National Comorbidity Survey Replication. *Comprehensive psychiatry*, *50*(4), 299–306 |
| **Kaminga, 2018** | Kaminga, A. C., Dai, W., Liu, A., Myaba, J., Banda, R., Wen, S. W., & Pan, X. (2018). Rate of and time to symptomatic remission in first-episode psychosis in Northern Malawi: A STROBE-compliant article. *Medicine*, *97*(45), e13078 |
| **Kaneriya, 2016** | Kaneriya, S. H., Robbins-Welty, G. A., Smagula, S. F., Karp, J. F., Butters, M. A., Lenze, E. J., Mulsant, B. H., Blumberger, D., Anderson, S. J., Dew, M. A., Lotrich, F., Aizenstein, H. J., Diniz, B. S., & Reynolds, C. F., 3rd (2016). Predictors and Moderators of Remission With Aripiprazole Augmentation in Treatment-Resistant Late-Life Depression: An Analysis of the IRL-GRey Randomized Clinical Trial. *JAMA psychiatry*, *73*(4), 329–336 |
| **Kapur, 2018** | Kapur, V., Nadella, R. K., Sathur Raghuraman, B., Saraf, G., Mishra, S., Srinivasmurthy, N., Jain, S., Del Zompo, M., & Viswanath, B. (2019). Clinical factors associated with lithium treatment response in bipolar disorder patients from India. *Asian journal of psychiatry*, *39*, 165–168 |
| **Karthick, 2015** | Karthick, S., Kattimani, S., Rajkumar, R. P., Bharadwaj, B., & Sarkar, S. (2015). Long term course of bipolar I disorder in India: using retrospective life chart method. *Journal of affective disorders*, *173*, 255–260 |
| **Kato, 2000** | Kato, T., Inubushi, T., & Kato, N. (2000). Prediction of lithium response by 31P-MRS in bipolar disorder. *The international journal of neuropsychopharmacology*, *3*(1), 83–85 |
| **Katon, 2002** | Katon, W., Russo, J., Frank, E., Barrett, J., Williams, J. W., Jr, Oxman, T., Sullivan, M., & Cornell, J. (2002). Predictors of nonresponse to treatment in primary care patients with dysthymia. *General hospital psychiatry*, *24*(1), 20–27 |
| **Kautzky, 2017** | Kautzky, A., Baldinger-Melich, P., Kranz, G. S., Vanicek, T., Souery, D., Montgomery, S., Mendlewicz, J., Zohar, J., Serretti, A., Lanzenberger, R., & Kasper, S. (2017). A New Prediction Model for Evaluating Treatment-Resistant Depression. *The Journal of clinical psychiatry*, *78*(2), 215–222 |
| **Keel, 2005** | Keel, P. K., Dorer, D. J., Franko, D. L., Jackson, S. C., & Herzog, D. B. (2005). Postremission predictors of relapse in women with eating disorders. *The American journal of psychiatry*, *162*(12), 2263–2268 |
| **Kessing, 2014** | Kessing, L. V., Vradi, E., & Andersen, P. K. (2014). Starting lithium prophylaxis early v. late in bipolar disorder. *The British journal of psychiatry : the journal of mental science*, *205*(3), 214–220 |
| **Kessler, 1993** | Kessler, R. C., & Magee, W. J. (1993). Childhood adversities and adult depression: basic patterns of association in a US national survey. *Psychological medicine*, *23*(3), 679–690 |
| **Kessler, 2016** | Kessler, R. C., van Loo, H. M., Wardenaar, K. J., Bossarte, R. M., Brenner, L. A., Cai, T., Ebert, D. D., Hwang, I., Li, J., de Jonge, P., Nierenberg, A. A., Petukhova, M. V., Rosellini, A. J., Sampson, N. A., Schoevers, R. A., Wilcox, M. A., & Zaslavsky, A. M. (2016). Testing a machine-learning algorithm to predict the persistence and severity of major depressive disorder from baseline self-reports. *Molecular psychiatry*, *21*(10), 1366–1371 |
| **Khodayari-Rostamabad, 2013** | Khodayari-Rostamabad, A., Reilly, J. P., Hasey, G. M., de Bruin, H., & Maccrimmon, D. J. (2013). A machine learning approach using EEG data to predict response to SSRI treatment for major depressive disorder. *Clinical neurophysiology : official journal of the International Federation of Clinical Neurophysiology*, *124*(10), 1975–1985 |
| **Kim, 2006** | Kim, H., Lim, S. W., Kim, S., Kim, J. W., Chang, Y. H., Carroll, B. J., & Kim, D. K. (2006). Monoamine transporter gene polymorphisms and antidepressant response in koreans with late-life depression. *JAMA*, *296*(13), 1609–1618 |
| **Kim, 2009** | Kim, S. G., Kim, C. M., Choi, S. W., Jae, Y. M., Lee, H. G., Son, B. K., Kim, J. G., Choi, Y. S., Kim, H. O., Kim, S. Y., & Oslin, D. W. (2009). A micro opioid receptor gene polymorphism (A118G) and naltrexone treatment response in adherent Korean alcohol-dependent patients. *Psychopharmacology*, *201*(4), 611–618 |
| **Kim, 2013** | Kim, B., Cho, S. J., Lee, K. S., Lee, J. Y., Choe, A. Y., Lee, J. E., Choi, T. K., & Lee, S. H. (2013). Factors associated with treatment outcomes in mindfulness-based cognitive therapy for panic disorder. *Yonsei medical journal*, *54*(6), 1454–1462 |
| **Kim, 2015** | Kim, J. W., Sharma, V., & Ryan, N. D. (2015). Predicting Methylphenidate Response in ADHD Using Machine Learning Approaches. *The international journal of neuropsychopharmacology*, *18*(11), pyv052 |
| **Kim., 2020** | Kim, Y., Hersch, J., Bodell, L. P., Schebendach, J., Hildebrandt, T., Walsh, B. T., & Mayer, L. E. S. (2021). The association between leptin and weight maintenance outcome in anorexia nervosa. *The International journal of eating disorders*, *54*(4), 527–534 |
| **Kirchheiner, 2007** | Kirchheiner, J., Nickchen, K., Sasse, J., Bauer, M., Roots, I., & Brockmöller, J. (2007). A 40-basepair VNTR polymorphism in the dopamine transporter (DAT1) gene and the rapid response to antidepressant treatment. *The pharmacogenomics journal*, *7*(1), 48–55 |
| **Klein, 2018** | Klein, N. S., Holtman, G. A., Bockting, C. L. H., Heymans, M. W., & Burger, H. (2018). Development and validation of a clinical prediction tool to estimate the individual risk of depressive relapse or recurrence in individuals with recurrent depression. *Journal of psychiatric research*, *104*, 1–7 |
| **Kooji, 2008** | Kooij, J. S., Boonstra, A. M., Vermeulen, S. H., Heister, A. G., Burger, H., Buitelaar, J. K., & Franke, B. (2008). Response to methylphenidate in adults with ADHD is associated with a polymorphism in SLC6A3 (DAT1). *American journal of medical genetics. Part B, Neuropsychiatric genetics : the official publication of the International Society of Psychiatric Genetics*, *147B*(2), 201–208. |
| **Kok, 2009** | Kok, R. M., Nolen, W. A., & Heeren, T. J. (2009). Outcome of late-life depression after 3 years of sequential treatment. *Acta psychiatrica Scandinavica*, *119*(4), 274–281 |
| **Korb, 2009** | Korb, A. S., Hunter, A. M., Cook, I. A., & Leuchter, A. F. (2009). Rostral anterior cingulate cortex theta current density and response to antidepressants and placebo in major depression. *Clinical neurophysiology : official journal of the International Federation of Clinical Neurophysiology*, *120*(7), 1313–1319 |
| **Korgaonkar, 2014** | Korgaonkar, M. S., Williams, L. M., Song, Y. J., Usherwood, T., & Grieve, S. M. (2014). Diffusion tensor imaging predictors of treatment outcomes in major depressive disorder. *The British journal of psychiatry : the journal of mental science*, *205*(4), 321–328 |
| **Koutsouleris, 2017** | Koutsouleris, N., Wobrock, T., Guse, B., Langguth, B., Landgrebe, M., Eichhammer, P., Frank, E., Cordes, J., Wölwer, W., Musso, F., Winterer, G., Gaebel, W., Hajak, G., Ohmann, C., Verde, P. E., Rietschel, M., Ahmed, R., Honer, W. G., Dwyer, D., Ghaseminejad, F., … Hasan, A. (2018). Predicting Response to Repetitive Transcranial Magnetic Stimulation in Patients With Schizophrenia Using Structural Magnetic Resonance Imaging: A Multisite Machine Learning Analysis. *Schizophrenia bulletin*, *44*(5), 1021–1034 |
| **Kusalic, 1988** | Kusalic, M., & Engelsmann, F. (1998). Predictors of lithium treatment responsiveness in bipolar patients. A two-year prospective study. *Neuropsychobiology*, *37*(3), 146–149 |
| **Lambert 2010** | Lambert, M., De Marinis, T., Pfeil, J., Naber, D., & Schreiner, A. (2010). Establishing remission and good clinical functioning in schizophrenia: predictors of best outcome with long-term risperidone long-acting injectable treatment. *European psychiatry : the journal of the Association of European Psychiatrists*, *25*(4), 220–229 |
| **Law, 2016** | Law, B., Gullo, M. J., Daglish, M., Kavanagh, D. J., Feeney, G. F., Young, R. M., & Connor, J. P. (2016). Craving Mediates Stress in Predicting Lapse During Alcohol Dependence Treatment. *Alcoholism, clinical and experimental research*, *40*(5), 1058–1064 |
| **Lee, 2008** | Lee, I. S., Kim, K. J., Kang, E. H., & Yu, B. H. (2008). beta-adrenoceptor affinity as a biological predictor of treatment response to paroxetine in patients with acute panic disorder. *Journal of affective disorders*, *110*(1-2), 156–160 |
| **Leighton & Krishnadas, 2019** | Leighton, S. P., Krishnadas, R., Chung, K., Blair, A., Brown, S., Clark, S., Sowerbutts, K., Schwannauer, M., Cavanagh, J., & Gumley, A. I. (2019). Predicting one-year outcome in first episode psychosis using machine learning. *PloS one*, *14*(3), e0212846 |
| **Leighton & Upthegrove, 2019** | Leighton, S. P., Upthegrove, R., Krishnadas, R., Benros, M. E., Broome, M. R., Gkoutos, G. V., Liddle, P. F., Singh, S. P., Everard, L., Jones, P. B., Fowler, D., Sharma, V., Freemantle, N., Christensen, R. H. B., Albert, N., Nordentoft, M., Schwannauer, M., Cavanagh, J., Gumley, A. I., Birchwood, M., … Mallikarjun, P. K. (2019). Development and validation of multivariable prediction models of remission, recovery, and quality of life outcomes in people with first episode psychosis: a machine learning approach. *The Lancet. Digital health*, *1*(6), e261–e270 |
| **Liber, 2010** | Liber, J. M., van Widenfelt, B. M., Goedhart, A. W., Utens, E. M., van der Leeden, A. J., Markus, M. T., & Treffers, P. D. (2008). Parenting and parental anxiety and depression as predictors of treatment outcome for childhood anxiety disorders: has the role of fathers been underestimated?. *Journal of clinical child and adolescent psychology : the official journal for the Society of Clinical Child and Adolescent Psychology, American Psychological Association, Division 53*, *37*(4), 747–758 |
| **Licht-Strunk, 2009** | Licht-Strunk, E., Van Marwijk, H. W., Hoekstra, T., Twisk, J. W., De Haan, M., & Beekman, A. T. (2009). Outcome of depression in later life in primary care: longitudinal cohort study with three years' follow-up. *BMJ (Clinical research ed.)*, *338*, a3079 |
| **Lieberman, 2003** | Lieberman, J. A., Phillips, M., Gu, H., Stroup, S., Zhang, P., Kong, L., Ji, Z., Koch, G., & Hamer, R. M. (2003). Atypical and conventional antipsychotic drugs in treatment-naive first-episode schizophrenia: a 52-week randomized trial of clozapine vs chlorpromazine. *Neuropsychopharmacology : official publication of the American College of Neuropsychopharmacology*, *28*(5), 995–1003 |
| **Lin, 2018** | Lin, E., Kuo, P. H., Liu, Y. L., Yu, Y. W., Yang, A. C., & Tsai, S. J. (2018). A Deep Learning Approach for Predicting Antidepressant Response in Major Depression Using Clinical and Genetic Biomarkers. *Frontiers in psychiatry*, *9*, 290 |
| **Linszen, 1994** | Linszen, D. H., Dingemans, P. M., & Lenior, M. E. (1994). Cannabis abuse and the course of recent-onset schizophrenic disorders. *Archives of general psychiatry*, *51*(4), 273–279 |
| **Linszen, 1997** | Linszen, D. H., Dingemans, P. M., Nugter, M. A., Van der Does, A. J., Scholte, W. F., & Lenior, M. A. (1997). Patient attributes and expressed emotion as risk factors for psychotic relapse. *Schizophrenia bulletin*, *23*(1), 119–130 |
| **Liu, 2015** | Liu, Y., Sareen, J., Bolton, J., & Wang, J. (2015). Development and validation of a risk-prediction algorithm for the recurrence of panic disorder. *Depression and anxiety*, *32*(5), 341–348 |
| **Lock, 2006** | Lock, J., Couturier, J., Bryson, S., & Agras, S. (2006). Predictors of dropout and remission in family therapy for adolescent anorexia nervosa in a randomized clinical trial. *The International journal of eating disorders*, *39*(8), 639–647 |
| **Lock, 2008** | Lock, James & Grange, Daniel & Crosby, Ross. (2008). Exploring possible mechanisms of change in family‐based treatment for adolescent bulimia nervosa. Journal of Family Therapy. 30. 260 - 271 |
| **Lopez-Quintero, 2011** | Lopez-Quintero, C., Hasin, D. S., de Los Cobos, J. P., Pines, A., Wang, S., Grant, B. F., & Blanco, C. (2011). Probability and predictors of remission from life-time nicotine, alcohol, cannabis or cocaine dependence: results from the National Epidemiologic Survey on Alcohol and Related Conditions. *Addiction (Abingdon, England)*, *106*(3), 657–669 |
| **Lorenzo-Luaces, 2017** | Lorenzo-Luaces, L., DeRubeis, R. J., van Straten, A., & Tiemens, B. (2017). A prognostic index (PI) as a moderator of outcomes in the treatment of depression: A proof of concept combining multiple variables to inform risk-stratified stepped care models. *Journal of affective disorders*, *213*, 78–85 |
| **Lowe, 2005** | Löwe, B., Schenkel, I., Bair, M. J., & Göbel, C. (2005). Efficacy, predictors of therapy response, and safety of sertraline in routine clinical practice: prospective, open-label, non-interventional postmarketing surveillance study in 1878 patients. *Journal of affective disorders*, *87*(2-3), 271–279 |
| **Luo, 2013** | Luo, X., Zhang, S., Hu, S., Bednarski, S. R., Erdman, E., Farr, O. M., Hong, K. I., Sinha, R., Mazure, C. M., & Li, C. S. (2013). Error processing and gender-shared and -specific neural predictors of relapse in cocaine dependence. *Brain : a journal of neurology*, *136*(Pt 4), 1231–1244 |
| **Lustman, 2006** | Lustman, P. J., Clouse, R. E., Nix, B. D., Freedland, K. E., Rubin, E. H., McGill, J. B., Williams, M. M., Gelenberg, A. J., Ciechanowski, P. S., & Hirsch, I. B. (2006). Sertraline for prevention of depression recurrence in diabetes mellitus: a randomized, double-blind, placebo-controlled trial. *Archives of general psychiatry*, *63*(5), 521–529 |
| **Maarsingh, 2018** | Maarsingh, O. R., Heymans, M. W., Verhaak, P. F., Penninx, B. W. J. H., & Comijs, H. C. (2018). Development and external validation of a prediction rule for an unfavorable course of late-life depression: A multicenter cohort study. *Journal of affective disorders*, *235*, 105–113 |
| **Maciukiewicz, 2018** | Maciukiewicz, M., Marshe, V. S., Hauschild, A. C., Foster, J. A., Rotzinger, S., Kennedy, J. L., Kennedy, S. H., Müller, D. J., & Geraci, J. (2018). GWAS-based machine learning approach to predict duloxetine response in major depressive disorder. *Journal of psychiatric research*, *99*, 62–68 |
| **Malla, 2008** | Malla, A., Norman, R., Bechard-Evans, L., Schmitz, N., Manchanda, R., & Cassidy, C. (2008). Factors influencing relapse during a 2-year follow-up of first-episode psychosis in a specialized early intervention service. *Psychological medicine*, *38*(11), 1585–1593 |
| **Mancebo, 2014** | Mancebo, M. C., Boisseau, C. L., Garnaat, S. L., Eisen, J. L., Greenberg, B. D., Sibrava, N. J., Stout, R. L., & Rasmussen, S. A. (2014). Long-term course of pediatric obsessive-compulsive disorder: 3 years of prospective follow-up. *Comprehensive psychiatry*, *55*(7), 1498–1504 |
| **Manchanda, 2005** | Manchanda, R., Norman, R. M., Malla, A. K., Harricharan, R., & Northcott, S. (2005). Persistent psychoses in first episode patients. *Schizophrenia research*, *80*(1), 113–116 |
| **Mansson, 2015** | Månsson, K. N., Frick, A., Boraxbekk, C. J., Marquand, A. F., Williams, S. C., Carlbring, P., Andersson, G., & Furmark, T. (2015). Predicting long-term outcome of Internet-delivered cognitive behavior therapy for social anxiety disorder using fMRI and support vector machine learning. *Translational psychiatry*, *5*(3), e530 |
| **Marchesi , 2006a** | Marchesi, C., De Panfilis, C., Cantoni, A., Fontò, S., Giannelli, M. R., & Maggini, C. (2006). Personality disorders and response to medication treatment in panic disorder: a 1-year naturalistic study. *Progress in neuro-psychopharmacology & biological psychiatry*, *30*(7), 1240–1245 |
| **Marchesi , 2006b** | Marchesi, C., Cantoni, A., Fontò, S., Giannelli, M. R., & Maggini, C. (2006). The effect of temperament and character on response to selective serotonin reuptake inhibitors in panic disorder. *Acta psychiatrica Scandinavica*, *114*(3), 203–210 |
| **Marquenie, 2006** | Marquenie, L. A., Schadé, A., Van Balkom, A. J., Koeter, M., Frenken, S., van den Brink, W., & van Dyck, R. (2006). Comorbid phobic disorders do not influence outcome of alcohol dependence treatment. Results of a naturalistic follow-up study. *Alcohol and alcoholism (Oxford, Oxfordshire)*, *41*(2), 168–173 |
| **Marshe, 2021** | Marshe, V. S., Maciukiewicz, M., Hauschild, A. C., Islam, F., Qin, L., Tiwari, A. K., Sibille, E., Blumberger, D. M., Karp, J. F., Flint, A. J., Turecki, G., Lam, R. W., Milev, R. V., Frey, B. N., Rotzinger, S., Foster, J. A., Kennedy, S. H., Kennedy, J. L., Mulsant, B. H., Reynolds, C. F., 3rd, … Müller, D. J. (2021). Genome-wide analysis suggests the importance of vascular processes and neuroinflammation in late-life antidepressant response. *Translational psychiatry*, *11*(1), 127 |
| **Mavissakalian and Perel, 2002** | Mavissakalian, M. R., & Perel, J. M. (2002). Duration of imipramine therapy and relapse in panic disorder with agoraphobia. *Journal of clinical psychopharmacology*, *22*(3), 294–299 |
| **Mazure, 2000** | Mazure, C. M., Bruce, M. L., Maciejewski, P. K., & Jacobs, S. C. (2000). Adverse life events and cognitive-personality characteristics in the prediction of major depression and antidepressant response. *The American journal of psychiatry*, *157*(6), 896–903 |
| **McAweeney, 2005** | McAweeney, M. J., Zucker, R. A., Fitzgerald, H. E., Puttler, L. I., & Wong, M. M. (2005). Individual and partner predictors of recovery from alcohol-use disorder over a nine-year interval: findings from a community sample of alcoholic married men. *Journal of studies on alcohol*, *66*(2), 220–228 |
| **McMakin, 2012** | McMakin, D. L., Olino, T. M., Porta, G., Dietz, L. J., Emslie, G., Clarke, G., Wagner, K. D., Asarnow, J. R., Ryan, N. D., Birmaher, B., Shamseddeen, W., Mayes, T., Kennard, B., Spirito, A., Keller, M., Lynch, F. L., Dickerson, J. F., & Brent, D. A. (2012). Anhedonia predicts poorer recovery among youth with selective serotonin reuptake inhibitor treatment-resistant depression. *Journal of the American Academy of Child and Adolescent Psychiatry*, *51*(4), 404–411 |
| **Michalak, 2008** | Michalak, J., Heidenreich, T., Meibert, P., & Schulte, D. (2008). Mindfulness predicts relapse/recurrence in major depressive disorder after mindfulness-based cognitive therapy. *The Journal of nervous and mental disease*, *196*(8), 630–633 |
| **Michalak, 2011** | Michalak, J., Hölz, A., & Teismann, T. (2011). Rumination as a predictor of relapse in mindfulness-based cognitive therapy for depression. *Psychology and psychotherapy*, *84*(2), 230–236 |
| **Mocking, 2015** | Mocking, R. J., Pellikaan, C. M., Lok, A., Assies, J., Ruhé, H. G., Koeter, M. W., Visser, I., Bockting, C. L., Olff, M., & Schene, A. H. (2015). DHEAS and cortisol/DHEAS-ratio in recurrent depression: State, or trait predicting 10-year recurrence?. *Psychoneuroendocrinology*, *59*, 91–101 |
| **Mojarrad, 2014** | Mojarrad, M., Samet, J. H., Cheng, D. M., Winter, M. R., & Saitz, R. (2014). Marijuana use and achievement of abstinence from alcohol and other drugs among people with substance dependence: a prospective cohort study. *Drug and alcohol dependence*, *142*, 91–97 |
| **Mojtabai, 2003** | Mojtabai, R., Lavelle, J., Gibson, P. J., & Bromet, E. J. (2003). Atypical antipsychotics in first admission schizophrenia: medication continuation and outcomes. *Schizophrenia bulletin*, *29*(3), 519–530 |
| **Morel, 2020** | Morel, D., Yu, K. C., Liu-Ferrara, A., Caceres-Suriel, A. J., Kurtz, S. G., & Tabak, Y. P. (2020). Predicting hospital readmission in patients with mental or substance use disorders: A machine learning approach. *International journal of medical informatics*, *139*, 104136 |
| **Morimoto, 2011** | Morimoto, S. S., Gunning, F. M., Murphy, C. F., Kanellopoulos, D., Kelly, R. E., & Alexopoulos, G. S. (2011). Executive function and short-term remission of geriatric depression: the role of semantic strategy. *The American journal of geriatric psychiatry : official journal of the American Association for Geriatric Psychiatry*, *19*(2), 115–122 |
| **Mulsant, 2001** | Jeste, D. V., & Finkel, S. I. (2000). Psychosis of Alzheimer's disease and related dementias. Diagnostic criteria for a distinct syndrome. *The American journal of geriatric psychiatry : official journal of the American Association for Geriatric Psychiatry*, *8*(1), 29–34 |
| **Mumtaz, 2017** | Mumtaz, W., Xia, L., Mohd Yasin, M. A., Azhar Ali, S. S., & Malik, A. S. (2017). A wavelet-based technique to predict treatment outcome for Major Depressive Disorder. *PloS one*, *12*(2), e0171409 |
| **Munsch, 2012** | Munsch, S., Meyer, A. H., & Biedert, E. (2012). Efficacy and predictors of long-term treatment success for Cognitive-Behavioral Treatment and Behavioral Weight-Loss-Treatment in overweight individuals with binge eating disorder. *Behaviour research and therapy*, *50*(12), 775–785 |
| **Murrough, 2015** | Murrough, J. W., Burdick, K. E., Levitch, C. F., Perez, A. M., Brallier, J. W., Chang, L. C., Foulkes, A., Charney, D. S., Mathew, S. J., & Iosifescu, D. V. (2015). Neurocognitive effects of ketamine and association with antidepressant response in individuals with treatment-resistant depression: a randomized controlled trial. *Neuropsychopharmacology : official publication of the American College of Neuropsychopharmacology*, *40*(5), 1084–1090 |
| **Nemeroff, 2003** | Nemeroff, C. B., Heim, C. M., Thase, M. E., Klein, D. N., Rush, A. J., Schatzberg, A. F., Ninan, P. T., McCullough, J. P., Jr, Weiss, P. M., Dunner, D. L., Rothbaum, B. O., Kornstein, S., Keitner, G., & Keller, M. B. (2003). Differential responses to psychotherapy versus pharmacotherapy in patients with chronic forms of major depression and childhood trauma. *Proceedings of the National Academy of Sciences of the United States of America*, *100*(24), 14293–14296 |
| **Nishioka, 2013** | Nishioka, G., Yashima, H., Kiuchi, Y., Nakamura, S., Oyamada, H., Ishii, M., & Kudo, I. (2013). Prediction and structural equation model of sertraline treatment response in Japanese patients with major depressive disorder. *Human psychopharmacology*, *28*(6), 576–585 |
| **O'Keefe, 2019** | O'Keeffe, D., Hannigan, A., Doyle, R., Kinsella, A., Sheridan, A., Kelly, A., Madigan, K., Lawlor, E., & Clarke, M. (2019). The iHOPE-20 study: Relationships between and prospective predictors of remission, clinical recovery, personal recovery and resilience 20 years on from a first episode psychosis. *The Australian and New Zealand journal of psychiatry*, *53*(11), 1080–1092 |
| **O'Leary, 1996** | O'Leary, D. A., & Lee, A. S. (1996). Seven year prognosis in depression. Mortality and readmission risk in the Nottingham ECT cohort. *The British journal of psychiatry : the journal of mental science*, *169*(4), 423–429 |
| **Oudega, 2011** | Oudega, M. L., van Exel, E., Wattjes, M. P., Comijs, H. C., Scheltens, P., Barkhof, F., Eikelenboom, P., de Craen, A. J., Beekman, A. T., & Stek, M. L. (2011). White matter hyperintensities, medial temporal lobe atrophy, cortical atrophy, and response to electroconvulsive therapy in severely depressed elderly patients. *The Journal of clinical psychiatry*, *72*(1), 104–112 |
| **Owens, 2010** | Owens, D. C., Johnstone, E. C., Miller, P., Macmillan, J. F., & Crow, T. J. (2010). Duration of untreated illness and outcome in schizophrenia: test of predictions in relation to relapse risk. *The British journal of psychiatry : the journal of mental science*, *196*(4), 296–301 |
| **Papakostas, 2003a** | Papakostas, G. I., Petersen, T., Denninger, J., Sonawalla, S. B., Mahal, Y., Alpert, J. E., Nierenberg, A. A., & Fava, M. (2003). Somatic symptoms in treatment-resistant depression. *Psychiatry research*, *118*(1), 39–45 |
| **Papakostas, 2003b** | Papakostas, G. I., Petersen, T., Mischoulon, D., Hughes, M. E., Spector, A. R., Alpert, J. E., Fava, M., & Nierenberg, A. A. (2003). Functioning and interpersonal relationships as predictors of response in treatment-resistant depression. *Comprehensive psychiatry*, *44*(1), 44–50 |
| **Papakostas, 2010** | Papakostas, G. I., Chuzi, S. E., Sousa, J. L., & Fava, M. (2010). 5HT1A-mediated stimulation of cortisol release in major depression: use of non-invasive cortisol measurements to predict clinical response. *European archives of psychiatry and clinical neuroscience*, *260*(2), 175–180 |
| **Patel, 2015** | Patel, M. J., Andreescu, C., Price, J. C., Edelman, K. L., Reynolds, C. F., 3rd, & Aizenstein, H. J. (2015). Machine learning approaches for integrating clinical and imaging features in late-life depression classification and response prediction. *International journal of geriatric psychiatry*, *30*(10), 1056–1067 |
| **Paulino, 2017** | Paulino, S., Pombo, S., Ismail, F., Figueira, M. L., & Lesch, O. (2017). The role of affective temperament as a predictor of relapse in alcohol dependence. *Personality and mental health*, *11*(4), 278–289 |
| **Paulus, 2005** | Paulus, M. P., Tapert, S. F., & Schuckit, M. A. (2005). Neural activation patterns of methamphetamine-dependent subjects during decision making predict relapse. *Archives of general psychiatry*, *62*(7), 761–768 |
| **Penick, 2010** | Penick, E. C., Knop, J., Nickel, E. J., Jensen, P., Manzardo, A. M., Lykke-Mortensen, E., & Gabrielli, W. F. (2010). Do premorbid predictors of alcohol dependence also predict the failure to recover from alcoholism?. *Journal of studies on alcohol and drugs*, *71*(5), 685–694 |
| **Perlis, 2003** | Perlis, R. H., Alpert, J., Nierenberg, A. A., Mischoulon, D., Yeung, A., Rosenbaum, J. F., & Fava, M. (2003). Clinical and sociodemographic predictors of response to augmentation, or dose increase among depressed outpatients resistant to fluoxetine 20 mg/day. *Acta psychiatrica Scandinavica*, *108*(6), 432–438 |
| **Perlis, 2004** | Perlis, R. H., Iosifescu, D. V., Alpert, J., Nierenberg, A. A., Rosenbaum, J. F., & Fava, M. (2004). Effect of medical comorbidity on response to fluoxetine augmentation or dose increase in outpatients with treatment-resistant depression. *Psychosomatics*, *45*(3), 224–229 |
| **Peters, 2015** | Peters, A. T., Shesler, L. W., Sylvia, L., da Silva Magalhaes, P. V., Miklowitz, D. J., Otto, M. W., Frank, E., Berk, M., Dougherty, D. D., Nierenberg, A. A., & Deckersbach, T. (2016). Medical burden, body mass index and the outcome of psychosocial interventions for bipolar depression. *The Australian and New Zealand journal of psychiatry*, *50*(7), 667–677 |
| **Petersen, 2008** | Petersen, L., Thorup, A., Øqhlenschlaeger, J., Christensen, T. Ø., Jeppesen, P., Krarup, G., Jørrgensen, P., Mortensen, E. L., & Nordentoft, M. (2008). Predictors of remission and recovery in a first-episode schizophrenia spectrum disorder sample: 2-year follow-up of the OPUS trial. *Canadian journal of psychiatry. Revue canadienne de psychiatrie*, *53*(10), 660–670 |
| **Pfennig, 2010** | Pfennig, A., Schlattmann, P., Alda, M., Grof, P., Glenn, T., Müller-Oerlinghausen, B., Suwalska, A., Rybakowski, J., Willich, S. N., Bauer, M., & Berghöfer, A. (2010). Influence of atypical features on the quality of prophylactic effectiveness of long-term lithium treatment in bipolar disorders. *Bipolar disorders*, *12*(4), 390–396 |
| **Pinto, 2008** | Pinto, E., Reggers, J., Gorwood, P., Boni, C., Scantamburlo, G., Pitchot, W., & Ansseau, M. (2008). The short allele of the serotonin transporter promoter polymorphism influences relapse in alcohol dependence. *Alcohol and alcoholism (Oxford, Oxfordshire)*, *43*(4), 398–400 |
| **Pintor, 2009** | Pintor, L., Torres, X., Navarro, V., Martinez de Osaba, M. A., Matrai, S., & Gastó, C. (2009). Prediction of relapse in melancholic depressive patients in a 2-year follow-up study with corticotropin releasing factor test. *Progress in neuro-psychopharmacology & biological psychiatry*, *33*(3), 463–469 |
| **Poirier, 2004** | Poirier, M. F., Laqueille, X., Jalfre, V., Willard, D., Bourdel, M. C., Fermanian, J., & Olié, J. P. (2004). Clinical profile of responders to buprenorphine as a substitution treatment in heroin addicts: results of a multicenter study of 73 patients. *Progress in neuro-psychopharmacology & biological psychiatry*, *28*(2), 267–272 |
| **Potkin, 2009** | Potkin, S. G., Weiden, P. J., Loebel, A. D., Warrington, L. E., Watsky, E. J., & Siu, C. O. (2009). Remission in schizophrenia: 196-week, double-blind treatment with ziprasidone vs. haloperidol. *The international journal of neuropsychopharmacology*, *12*(9), 1233–1248 |
| **Potter, 2004** | Potter, G. G., Kittinger, J. D., Wagner, H. R., Steffens, D. C., & Krishnan, K. R. (2004). Prefrontal neuropsychological predictors of treatment remission in late-life depression. *Neuropsychopharmacology : official publication of the American College of Neuropsychopharmacology*, *29*(12), 2266–2271 |
| **Powell, 2013** | Powell, T. R., Schalkwyk, L. C., Heffernan, A. L., Breen, G., Lawrence, T., Price, T., Farmer, A. E., Aitchison, K. J., Craig, I. W., Danese, A., Lewis, C., McGuffin, P., Uher, R., Tansey, K. E., & D'Souza, U. M. (2013). Tumor necrosis factor and its targets in the inflammatory cytokine pathway are identified as putative transcriptomic biomarkers for escitalopram response. *European neuropsychopharmacology : the journal of the European College of Neuropsychopharmacology*, *23*(9), 1105–1114 |
| **Pu, 2013** | Pu, M., Zhang, Z., Xu, Z., Shi, Y., Geng, L., Yuan, Y., Zhang, X., & Reynolds, G. P. (2013). Influence of genetic polymorphisms in the glutamatergic and GABAergic systems and their interactions with environmental stressors on antidepressant response. *Pharmacogenomics*, *14*(3), 277–288 |
| **Rando, 2011** | Rando, K., Hong, K. I., Bhagwagar, Z., Li, C. S., Bergquist, K., Guarnaccia, J., & Sinha, R. (2011). Association of frontal and posterior cortical gray matter volume with time to alcohol relapse: a prospective study. *The American journal of psychiatry*, *168*(2), 183–192 |
| **Raskin, 2008** | Raskin, J., Xu, J. Y., & Kajdasz, D. K. (2008). Time to response for duloxetine 60 mg once daily versus placebo in elderly patients with major depressive disorder. *International psychogeriatrics*, *20*(2), 309–327 |
| **Redlich, 2016** | Redlich, R., Opel, N., Grotegerd, D., Dohm, K., Zaremba, D., Bürger, C., Münker, S., Mühlmann, L., Wahl, P., Heindel, W., Arolt, V., Alferink, J., Zwanzger, P., Zavorotnyy, M., Kugel, H., & Dannlowski, U. (2016). Prediction of Individual Response to Electroconvulsive Therapy via Machine Learning on Structural Magnetic Resonance Imaging Data. *JAMA psychiatry*, *73*(6), 557–564 |
| **Renwick, 2015** | Renwick, L., Lyne, J., Donoghue, B. O., Owens, L., Doyle, R., Hill, M., McCarthy, E., Pilling, M., O'Callaghan, E., & Clarke, M. (2015). Prodromal symptoms and remission following first episode psychosis. *Schizophrenia research*, *168*(1-2), 30–36 |
| **Rhebergen, 2015** | Rhebergen, D., Huisman, A., Bouckaert, F., Kho, K., Kok, R., Sienaert, P., Spaans, H. P., & Stek, M. (2015). Older age is associated with rapid remission of depression after electroconvulsive therapy: a latent class growth analysis. *The American journal of geriatric psychiatry : official journal of the American Association for Geriatric Psychiatry*, *23*(3), 274–282 |
| **Ribeiz, 2013** | Ribeiz, S. R., Duran, F., Oliveira, M. C., Bezerra, D., Castro, C. C., Steffens, D. C., Busatto Filho, G., & Bottino, C. M. (2013). Structural brain changes as biomarkers and outcome predictors in patients with late-life depression: a cross-sectional and prospective study. *PloS one*, *8*(11), e80049 |
| **Richard et al, 2005** | Richard, M., Bauer, S. and Kordy, H. (2005), Relapse in anorexia and bulimia nervosa—a 2.5-year follow-up study. Eur. Eat. Disorders Rev., 13: 180-190 |
| **Richieri, 2011** | Richieri, R., Boyer, L., Farisse, J., Colavolpe, C., Mundler, O., Lancon, C., & Guedj, E. (2011). Predictive value of brain perfusion SPECT for rTMS response in pharmacoresistant depression. *European journal of nuclear medicine and molecular imaging*, *38*(9), 1715–1722 |
| **Rickels, 2013** | Rickels, K., Etemad, B., Rynn, M. A., Lohoff, F. W., Mandos, L. A., & Gallop, R. (2013). Remission of generalized anxiety disorder after 6 months of open-label treatment with venlafaxine XR. *Psychotherapy and psychosomatics*, *82*(6), 363–371 |
| **Robinson, 1999** | Robinson, D., Woerner, M. G., Alvir, J. M., Bilder, R., Goldman, R., Geisler, S., Koreen, A., Sheitman, B., Chakos, M., Mayerhoff, D., & Lieberman, J. A. (1999). Predictors of relapse following response from a first episode of schizophrenia or schizoaffective disorder. *Archives of general psychiatry*, *56*(3), 241–247 |
| **Rossi, 2009** | Rossi, A., Bagalà, A., Del Curatolo, V., Scapati, F., Bernareggi, M.M. and Giustra, M.G. (2009), Remission in schizophrenia: one-year Italian prospective study of risperidone long-acting injectable (RLAI) in patients with schizophrenia or schizoaffective disorder. Hum. Psychopharmacol. Clin. Exp., 24: 574-583 |
| **Rudy, 2014** | Rudy, B. M., Lewin, A. B., Geffken, G. R., Murphy, T. K., & Storch, E. A. (2014). Predictors of treatment response to intensive cognitive-behavioral therapy for pediatric obsessive-compulsive disorder. *Psychiatry research*, *220*(1-2), 433–440 |
| **Saghafi, 2007** | Saghafi, R., Brown, C., Butters, M. A., Cyranowski, J., Dew, M. A., Frank, E., Gildengers, A., Karp, J. F., Lenze, E. J., Lotrich, F., Martire, L., Mazumdar, S., Miller, M. D., Mulsant, B. H., Weber, E., Whyte, E., Morse, J., Stack, J., Houck, P. R., Bensasi, S., … Reynolds, C. F., 3rd (2007). Predicting 6-week treatment response to escitalopram pharmacotherapy in late-life major depressive disorder. *International journal of geriatric psychiatry*, *22*(11), 1141–1146 |
| **Sakado, 1999** | Sakado, K., Sato, T., Uehara, T., Sakado, M., & Someya, T. (1999). Perceived parenting pattern and response to antidepressants in patients with major depression. *Journal of affective disorders*, *52*(1-3), 59–66 |
| **Sala, 2014** | Sala, R., Strober, M. A., Axelson, D. A., Gill, M. K., Castro-Fornieles, J., Goldstein, T. R., Goldstein, B. I., Ha, W., Liao, F., Iyengar, S., Yen, S., Hower, H., Hunt, J., Dickstein, D. P., Ryan, N. D., Keller, M. B., & Birmaher, B. (2014). Effects of comorbid anxiety disorders on the longitudinal course of pediatric bipolar disorders. *Journal of the American Academy of Child and Adolescent Psychiatry*, *53*(1), 72–81 |
| **Sander, 2006** | Sander, W., & Jux, M. (2006). Psychological distress in alcohol-dependent patients. Evaluating inpatient treatment with the symptom checklist (SCL-90-R). *European addiction research*, *12*(2), 61–66 |
| **Saravanan, 2010** | Saravanan, B., Jacob, K. S., Johnson, S., Prince, M., Bhugra, D., & David, A. S. (2010). Outcome of first-episode schizophrenia in India: longitudinal study of effect of insight and psychopathology. *The British journal of psychiatry : the journal of mental science*, *196*(6), 454–459 |
| **Schlegl, 2014** | Schlegl, S., Quadflieg, N., Löwe, B., Cuntz, U., & Voderholzer, U. (2014). Specialized inpatient treatment of adult anorexia nervosa: effectiveness and clinical significance of changes. *BMC psychiatry*, *14*, 258 |
| **Schlegl, 2016** | Schlegl, S., Diedrich, A., Neumayr, C., Fumi, M., Naab, S., & Voderholzer, U. (2016). Inpatient Treatment for Adolescents with Anorexia Nervosa: Clinical Significance and Predictors of Treatment Outcome. *European eating disorders review : the journal of the Eating Disorders Association*, *24*(3), 214–222 |
| **Schmidt, 2007** | Schmidt, L. G., & Smolka, M. N. (2007). Results from two pharmacotherapy trials show alcoholic smokers were more severely alcohol dependent but less prone to relapse than alcoholic non-smokers. *Alcohol and alcoholism (Oxford, Oxfordshire)*, *42*(3), 241–246 |
| **Schneekloth, 2012** | Schneekloth, T. D., Biernacka, J. M., Hall-Flavin, D. K., Karpyak, V. M., Frye, M. A., Loukianova, L. L., Stevens, S. R., Drews, M. S., Geske, J. R., & Mrazek, D. A. (2012). Alcohol craving as a predictor of relapse. *The American journal on addictions*, *21 Suppl 1*, S20–S26 |
| **Sebastian, 2019** | Sebastian, M. R., Wiemann, C. M., & Hergenroeder, A. C. (2019). Rate of weight gain as a predictor of readmission in adolescents with eating disorders. *International journal of adolescent medicine and health*, *33*(4), 10.1515/ijamh-2018-0228 |
| **Sellman, 1997** | Sellman, J. D., Mulder, R. T., Sullivan, P. F., & Joyce, P. R. (1997). Low persistence predicts relapse in alcohol dependence following treatment. *Journal of studies on alcohol*, *58*(3), 257–263 |
| **Seo, 2013** | Seo, D., Lacadie, C. M., Tuit, K., Hong, K. I., Constable, R. T., & Sinha, R. (2013). Disrupted ventromedial prefrontal function, alcohol craving, and subsequent relapse risk. *JAMA psychiatry*, *70*(7), 727–739 |
| **Seripa, 2015** | Seripa, D., Pilotto, A., Paroni, G., Fontana, A., D'Onofrio, G., Gravina, C., Urbano, M., Cascavilla, L., Paris, F., Panza, F., Padovani, A., & Pilotto, A. (2015). Role of the serotonin transporter gene locus in the response to SSRI treatment of major depressive disorder in late life. *Journal of psychopharmacology (Oxford, England)*, *29*(5), 623–633 |
| **Shamseddeen, 2011** | Shamseddeen, W., Asarnow, J. R., Clarke, G., Vitiello, B., Wagner, K. D., Birmaher, B., Keller, M. B., Emslie, G., Iyengar, S., Ryan, N. D., McCracken, J. T., Porta, G., Mayes, T., & Brent, D. A. (2011). Impact of physical and sexual abuse on treatment response in the Treatment of Resistant Depression in Adolescent Study (TORDIA). *Journal of the American Academy of Child and Adolescent Psychiatry*, *50*(3), 293–301 |
| **Shamseddeen 2012** | Shamseddeen, W., Clarke, G., Keller, M. B., Wagner, K. D., Birmaher, B., Emslie, G. J., Ryan, N., Asarnow, J. R., Porta, G., & Brent, D. A. (2012). Adjunctive sleep medications and depression outcome in the treatment of serotonin-selective reuptake inhibitor resistant depression in adolescents study. *Journal of child and adolescent psychopharmacology*, *22*(1), 29–36 |
| **Sheline, 2010** | Sheline, Y. I., Pieper, C. F., Barch, D. M., Welsh-Bohmer, K., McKinstry, R. C., MacFall, J. R., D'Angelo, G., Garcia, K. S., Gersing, K., Wilkins, C., Taylor, W., Steffens, D. C., Krishnan, R. R., & Doraiswamy, P. M. (2010). Support for the vascular depression hypothesis in late-life depression: results of a 2-site, prospective, antidepressant treatment trial. *Archives of general psychiatry*, *67*(3), 277–285 |
| **Sherbourne, 1995** | Sherbourne, C.D., Hays, R.D., & Wells, K.B. (1995). Personal and psychosocial risk factors for physical and mental health outcomes and course of depression among depressed patients. *Journal of consulting and clinical psychology, 63 3*, 345-55 . |
| **Shi, 2017** | Shi, Y., Li, M., Song, C., Xu, Q., Huo, R., Shen, L., Xing, Q., Cui, D., Li, W., Zhao, J., He, L., & Qin, S. (2017). Combined study of genetic and epigenetic biomarker risperidone treatment efficacy in Chinese Han schizophrenia patients. *Translational psychiatry*, *7*(7), e1170 |
| **Shiroma, 2014** | Shiroma, P. R., Drews, M. S., Geske, J. R., & Mrazek, D. A. (2014). SLC6A4 polymorphisms and age of onset in late-life depression on treatment outcomes with citalopram: a Sequenced Treatment Alternatives to Relieve Depression (STAR*D) report. *The American journal of geriatric psychiatry : official journal of the American Association for Geriatric Psychiatry*, *22*(11), 1140–1148 |
| **Simonsen, 2010** | Simonsen, E., Friis, S., Opjordsmoen, S., Mortensen, E. L., Haahr, U., Melle, I., Joa, I., Johannessen, J. O., Larsen, T. K., Røssberg, J. I., Rund, B. R., Vaglum, P., & McGlashan, T. H. (2010). Early identification of non-remission in first-episode psychosis in a two-year outcome study. *Acta psychiatrica Scandinavica*, *122*(5), 375–383 |
| **Simonsen, 2017** | Simonsen, C., Faerden, A., Romm, K. L., Berg, A. O., Bjella, T., Sundet, K., Ueland, T., Andreassen, O., & Melle, I. (2017). Early clinical recovery in first-episode psychosis: Symptomatic remission and its correlates at 1-year follow-up. *Psychiatry research*, *254*, 118–125 |
| **Simpson, 1997** | Simpson, H. B., Nee, J. C., & Endicott, J. (1997). First-episode major depression. Few sex differences in course. *Archives of general psychiatry*, *54*(7), 633–639 |
| **Singh, 2004** | Singh, S. P., Burns, T., Amin, S., Jones, P. B., & Harrison, G. (2004). Acute and transient psychotic disorders: precursors, epidemiology, course and outcome. *The British journal of psychiatry : the journal of mental science*, *185*, 452–459 |
| **Sinha, 2011** | Sinha, R., Fox, H. C., Hong, K. I., Hansen, J., Tuit, K., & Kreek, M. J. (2011). Effects of adrenal sensitivity, stress- and cue-induced craving, and anxiety on subsequent alcohol relapse and treatment outcomes. *Archives of general psychiatry*, *68*(9), 942–952 |
| **Sivaprakash, 2000** | Sivaprakash, B., Chandrasekaran, R., & Sahai, A. (2000). Predictors of response to electro-convulsive therapy in major depression. *Indian journal of psychiatry*, *42*(2), 148–155 |
| **Slaap, 1996** | Slaap, B. R., van Vliet, I. M., Westenberg, H. G., & Den Boer, J. A. (1996). Responders and non-responders to drug treatment in social phobia: differences at baseline and prediction of response. *Journal of affective disorders*, *39*(1), 13–19 |
| **Smagula, 2016** | Smagula, S. F., Wallace, M. L., Anderson, S. J., Karp, J. F., Lenze, E. J., Mulsant, B. H., Butters, M. A., Blumberger, D. M., Diniz, B. S., Lotrich, F. E., Dew, M. A., & Reynolds, C. F., 3rd (2016). Combining moderators to identify clinical profiles of patients who will, and will not, benefit from aripiprazole augmentation for treatment resistant late-life major depressive disorder. *Journal of psychiatric research*, *81*, 112–118 |
| **Smith, 2014** | Smith, N., Hill, R., Marshall, J., Keaney, F., & Wanigaratne, S. (2014). Sleep related beliefs and their association with alcohol relapse following residential alcohol detoxification treatment. *Behavioural and cognitive psychotherapy*, *42*(5), 593–604 |
| **Sneed, 2007** | Sneed, J. R., Keilp, J. G., Brickman, A. M., & Roose, S. P. (2008). The specificity of neuropsychological impairment in predicting antidepressant non-response in the very old depressed. *International journal of geriatric psychiatry*, *23*(3), 319–323 |
| **Sorbara, 2002** | Sorbara, F., Liraud, F., Assens, F., Abalan, F., & Verdoux, H. (2003). Substance use and the course of early psychosis: a 2-year follow-up of first-admitted subjects. *European psychiatry : the journal of the Association of European Psychiatrists*, *18*(3), 133–136 |
| **Spaans, 2013** | Spaans, H. P., Verwijk, E., Comijs, H. C., Kok, R. M., Sienaert, P., Bouckaert, F., Fannes, K., Vandepoel, K., Scherder, E. J., Stek, M. L., & Kho, K. H. (2013). Efficacy and cognitive side effects after brief pulse and ultrabrief pulse right unilateral electroconvulsive therapy for major depression: a randomized, double-blind, controlled study. *The Journal of clinical psychiatry*, *74*(11), e1029–e1036 |
| **Spashett, 2014** | Spashett, R., Fernie, G., Reid, I. C., & Cameron, I. M. (2014). MADRS symptom subtypes in ECT-treated depressed patients: relationship to response and subsequent ECT. *The journal of ECT*, *30*(3), 227–231 |
| **Spinhoven et al., 2016** | Spinhoven, P., Drost, J., de Rooij, M., van Hemert, A. M., & Penninx, B. W. (2016). Is Experiential Avoidance a Mediating, Moderating, Independent, Overlapping, or Proxy Risk Factor in the Onset, Relapse and Maintenance of Depressive Disorders?. *Cognitive therapy and research*, *40*, 150–163 |
| **Steketee, 1999** | Steketee, G., Eisen, J., Dyck, I., Warshaw, M., & Rasmussen, S. (1999). Predictors of course in obsessive-compulsive disorder. *Psychiatry research*, *89*(3), 229–238 |
| **Strakowski, 2007** | Strakowski, S. M., DelBello, M. P., Fleck, D. E., Adler, C. M., Anthenelli, R. M., Keck, P. E., Jr, Arnold, L. M., & Amicone, J. (2007). Effects of co-occurring cannabis use disorders on the course of bipolar disorder after a first hospitalization for mania. *Archives of general psychiatry*, *64*(1), 57–64 |
| **Strakowski, 2005** | Strakowski, S. M., DelBello, M. P., Fleck, D. E., Adler, C. M., Anthenelli, R. M., Keck, P. E., Jr, Arnold, L. M., & Amicone, J. (2005). Effects of co-occurring alcohol abuse on the course of bipolar disorder following a first hospitalization for mania. *Archives of general psychiatry*, *62*(8), 851–858 |
| **Strober, 1997** | Strober, M., Freeman, R. and Morrell, W. (1997), The long-term course of severe anorexia nervosa in adolescents: Survival analysis of recovery, relapse, and outcome predictors over 10–15 years in a prospective study. Int. J. Eat. Disord., 22: 339-360 |
| **Suija, 2011** | Suija, K., Aluoja, A., Kalda, R., & Maaroos, H. I. (2011). Factors associated with recurrent depression: a prospective study in family practice. *Family practice*, *28*(1), 22–28 |
| **Sullivan, 2003** | Sullivan, M. D., Katon, W. J., Russo, J. E., Frank, E., Barrett, J. E., Oxman, T. E., & Williams, J. W., Jr (2003). Patient beliefs predict response to paroxetine among primary care patients with dysthymia and minor depression. *The Journal of the American Board of Family Practice*, *16*(1), 22–31 |
| **Suter, 2011** | Suter, M., Strik, W., & Moggi, F. (2011). Depressive symptoms as a predictor of alcohol relapse after residential treatment programs for alcohol use disorder. *Journal of substance abuse treatment*, *41*(3), 225–232 |
| **Tang, 2014** | Tang, J. Y., Chang, W. C., Hui, C. L., Wong, G. H., Chan, S. K., Lee, E. H., Yeung, W. S., Wong, C. K., Tang, W. N., Chan, W. F., Pang, E. P., Tso, S., Ng, R. M., Hung, S. F., Dunn, E. L., Sham, P. C., & Chen, E. Y. (2014). Prospective relationship between duration of untreated psychosis and 13-year clinical outcome: a first-episode psychosis study. *Schizophrenia research*, *153*(1-3), 1–8 |
| **Taylor., 2011** | Taylor, W. D., McQuoid, D. R., Ashley-Koch, A., MacFall, J. R., Bridgers, J., Krishnan, R. R., & Steffens, D. C. (2011). BDNF Val66Met genotype and 6-month remission rates in late-life depression. *The pharmacogenomics journal*, *11*(2), 146–154 |
| **Taylor, 2008** | Taylor, W. D., Kuchibhatla, M., Payne, M. E., Macfall, J. R., Sheline, Y. I., Krishnan, K. R., & Doraiswamy, P. M. (2008). Frontal white matter anisotropy and antidepressant remission in late-life depression. *PloS one*, *3*(9), e3267 |
| **Ten Doesschate, 2014** | Ten Doesschate, F., van Eijndhoven, P., Tendolkar, I., van Wingen, G. A., & van Waarde, J. A. (2014). Pre-treatment amygdala volume predicts electroconvulsive therapy response. *Frontiers in psychiatry*, *5*, 169 |
| **Terra, 2008** | Terra, M. B., Barros, H. M., Stein, A. T., Figueira, I., Athayde, L. D., Ott, D. R., de Azambuja, R.deC., & da Silveira, D. X. (2008). Predictors of relapse in 300 Brazilian alcoholic patients: a 6-month follow-up study. *Substance use & misuse*, *43*(3-4), 403–411 |
| **Thirlwall, 2017** | Thirlwall, K., Cooper, P., & Creswell, C. (2017). Guided parent-delivered cognitive behavioral therapy for childhood anxiety: Predictors of treatment response. *Journal of anxiety disorders*, *45*, 43–48 |
| **Trim, 2013** | Trim, R. S., Schuckit, M. A., & Smith, T. L. (2013). Predictors of initial and sustained remission from alcohol use disorders: findings from the 30-year follow-up of the San Diego Prospective Study. *Alcoholism, clinical and experimental research*, *37*(8), 1424–1431 |
| **Trivedi, 2001** | Trivedi, M. H., Rush, A. J., Pan, J. Y., & Carmody, T. J. (2001). Which depressed patients respond to nefazodone and when?. *The Journal of clinical psychiatry*, *62*(3), 158–163 |
| **Trivedi, 2006** | Trivedi, M. H., Rush, A. J., Wisniewski, S. R., Nierenberg, A. A., Warden, D., Ritz, L., Norquist, G., Howland, R. H., Lebowitz, B., McGrath, P. J., Shores-Wilson, K., Biggs, M. M., Balasubramani, G. K., Fava, M., & STAR*D Study Team (2006). Evaluation of outcomes with citalopram for depression using measurement-based care in STAR*D: implications for clinical practice. *The American journal of psychiatry*, *163*(1), 28–40 |
| **Trucco, 2007** | Trucco, E. M., Connery, H. S., Griffin, M. L., & Greenfield, S. F. (2007). The relationship of self-esteem and self-efficacy to treatment outcomes of alcohol-dependent men and women. *The American journal on addictions*, *16*(2), 85–92 |
| **Tuithof, 2014** | Tuithof, M., ten Have, M., van den Brink, W., Vollebergh, W., & de Graaf, R. (2014). Alcohol consumption and symptoms as predictors for relapse of DSM-5 alcohol use disorder. *Drug and alcohol dependence*, *140*, 85–91 |
| **Tulloch, 2016** | Tulloch, A. D., David, A. S., & Thornicroft, G. (2016). Exploring the predictors of early readmission to psychiatric hospital. *Epidemiology and psychiatric sciences*, *25*(2), 181–193 |
| **Turkcapar, 2005** | Turkcapar, H., Kose, S., Ince, A., & Myrick, H. (2005). Beliefs as a predictor of relapse in alcohol-dependent Turkish men. *Journal of studies on alcohol*, *66*(6), 848–851 |
| **Turkington, 2009** | Turkington, A., Mulholland, C. C., Rushe, T. M., Anderson, R., McCaul, R., Barrett, S. L., Barr, R. S., & Cooper, S. J. (2009). Impact of persistent substance misuse on 1-year outcome in first-episode psychosis. *The British journal of psychiatry : the journal of mental science*, *195*(3), 242–248 |
| **Ucok, 2006** | Uçok, A., Polat, A., Cakir, S., & Genç, A. (2006). One year outcome in first episode schizophrenia. Predictors of relapse. *European archives of psychiatry and clinical neuroscience*, *256*(1), 37–43 |
| **Ucok, 2011** | Üçok, A., Serbest, S., & Kandemir, P. E. (2011). Remission after first-episode schizophrenia: results of a long-term follow-up. *Psychiatry research*, *189*(1), 33–37 |
| **Uher, 2010** | Uher, R., Huezo-Diaz, P., Perroud, N., Smith, R., Rietschel, M., Mors, O., Hauser, J., Maier, W., Kozel, D., Henigsberg, N., Barreto, M., Placentino, A., Dernovsek, M. Z., Schulze, T. G., Kalember, P., Zobel, A., Czerski, P. M., Larsen, E. R., Souery, D., Giovannini, C., … Craig, I. (2009). Genetic predictors of response to antidepressants in the GENDEP project. *The pharmacogenomics journal*, *9*(4), 225–233 |
| **Usta, 2019** | Usta, Miraç & Karabekiroglu, Koray & Şahin, Berkan & Aydın, Muazzez & Bozkurt, Abdullah & Karaosman, Tolga & Aral, Armagan & Çobanoğlu Osmanlı, Cansu & Duman Kurt, Ayşegül & Kesim, Neriman & Şahin, İrem & Urer, Emre. (2018). Use of machine learning methods in prediction of short-term outcome in autism spectrum disorders. |
| **Van Breda, 2018** | van Breda, W., Bremer, V., Becker, D., Hoogendoorn, M., Funk, B., Ruwaard, J., & Riper, H. (2018). Predicting therapy success for treatment as usual and blended treatment in the domain of depression. *Internet interventions*, *12*, 100–104 |
| **Van loo, 2015** | van Loo, H. M., Aggen, S. H., Gardner, C. O., & Kendler, K. S. (2015). Multiple risk factors predict recurrence of major depressive disorder in women. *Journal of affective disorders*, *180*, 52–61 |
| **Van loo, 2018** | van Loo, H. M., Aggen, S. H., Gardner, C. O., & Kendler, K. S. (2018). Sex similarities and differences in risk factors for recurrence of major depression. *Psychological medicine*, *48*(10), 1685–1693 |
| **Vaz, 2014** | Vaz, A. R., Conceição, E., & Machado, P. P. (2014). Early response as a predictor of success in guided self-help treatment for bulimic disorders. *European eating disorders review : the journal of the Eating Disorders Association*, *22*(1), 59–65 |
| **Verdoux, 2001** | Verdoux, H., Liraud, F., Bergey, C., Assens, F., Abalan, F., & van Os, J. (2001). Is the association between duration of untreated psychosis and outcome confounded? A two year follow-up study of first-admitted patients. *Schizophrenia research*, *49*(3), 231–241 |
| **Verma, 2012** | Verma, S., Subramaniam, M., Abdin, E., Poon, L. Y., & Chong, S. A. (2012). Symptomatic and functional remission in patients with first-episode psychosis. *Acta psychiatrica Scandinavica*, *126*(4), 282–289 |
| **Vielva, 2001** | Vielva, I. and Iraurgi, I. (2001), Cognitive and behavioural factors as predictors of abstinence following treatment for alcohol dependence. Addiction, 96: 297-303. |
| **Vigerland, 2017** | Vigerland, S., Serlachius, E., Thulin, U., Andersson, G., Larsson, J. O., & Ljótsson, B. (2017). Long-term outcomes and predictors of internet-delivered cognitive behavioral therapy for childhood anxiety disorders. *Behaviour research and therapy*, *90*, 67–75 |
| **Vitiello, 2011** | Vitiello, B., Emslie, G., Clarke, G., Wagner, K. D., Asarnow, J. R., Keller, M. B., Birmaher, B., Ryan, N. D., Kennard, B., Mayes, T. L., DeBar, L., Lynch, F., Dickerson, J., Strober, M., Suddath, R., McCracken, J. T., Spirito, A., Onorato, M., Zelazny, J., Porta, G., … Brent, D. A. (2011). Long-term outcome of adolescent depression initially resistant to selective serotonin reuptake inhibitor treatment: a follow-up study of the TORDIA sample. *The Journal of clinical psychiatry*, *72*(3), 388–396 |
| **Vito Agosti, 2013** | Agosti V. (2013). Predictors of alcohol dependence relapse during recurrence of major depression. *Journal of addictive diseases*, *32*(1), 79–84 |
| **Volonteri, 2010** | Volonteri, L. S., Cerveri, G., De Gaspari, I. F., Baldi, M. L., Rolandi, M. L., Papa, P., Mauri, M. C., & Mencacci, C. (2010). Long-acting injectable risperidone and metabolic ratio: a possible index of clinical outcome in treatment-resistant schizophrenic patients. *Psychopharmacology*, *210*(4), 489–497 |
| **Wade, 2006** | Wade, D., Harrigan, S., Edwards, J., Burgess, P. M., Whelan, G., & McGorry, P. D. (2006). Substance misuse in first-episode psychosis: 15-month prospective follow-up study. *The British journal of psychiatry : the journal of mental science*, *189*, 229–234 |
| **Wade, 2017** | Wade, Benjamin & Sui, Jing & Hellemann, Gerhard & Leaver, Amber & Espinoza, Randall & Woods, Roger & Abbott, Christopher & Joshi, Shantanu & Narr, Katherine. (2017). Inter and intra-hemispheric structural imaging markers predict depression relapse after electroconvulsive therapy: A multisite study. Translational Psychiatry |
| **Wagner, 2004** | Wagner, T., Krampe, H., Stawicki, S., Reinhold, J., Jahn, H., Mahlke, K., Barth, U., Sieg, S., Maul, O., Galwas, C., Aust, C., Kröner-Herwig, B., Brunner, E., Poser, W., Henn, F., Rüther, E., & Ehrenreich, H. (2004). Substantial decrease of psychiatric comorbidity in chronic alcoholics upon integrated outpatient treatment - results of a prospective study. *Journal of psychiatric research*, *38*(6), 619–635 |
| **Wagner, 2012** | Wagner, G., Penelo, E., Wanner, C., Gwinner, P., Trofaier, M. L., Imgart, H., Waldherr, K., Wöber-Bingöl, C., & Karwautz, A. F. (2013). Internet-delivered cognitive-behavioural therapy v. conventional guided self-help for bulimia nervosa: long-term evaluation of a randomised controlled trial. *The British journal of psychiatry : the journal of mental science*, *202*, 135–141 |
| **Wales, 2015** | Wales, J., Brewin, N., Cashmore, R., Haycraft, E., Baggott, J., Cooper, A., & Arcelus, J. (2016). Predictors of Positive Treatment Outcome in People With Anorexia Nervosa Treated in a Specialized Inpatient Unit: The Role of Early Response to Treatment. *European eating disorders review : the journal of the Eating Disorders Association*, *24*(5), 417–424 |
| **Wang, 2018** | Wang, J., Fan, Y., Dong, Y., Ma, M., Dong, Y., Niu, Y., Jiang, Y., Wang, H., Wang, Z., Wu, L., Sun, H., & Cui, C. (2018). Combining gray matter volume in the cuneus and the cuneus-prefrontal connectivity may predict early relapse in abstinent alcohol-dependent patients. *PloS one*, *13*(5), e0196860 |
| **Wang-Pattern, 2014** | Wang, J. L., Patten, S., Sareen, J., Bolton, J., Schmitz, N., & MacQueen, G. (2014). Development and validation of a prediction algorithm for use by health professionals in prediction of recurrence of major depression. *Depression and anxiety*, *31*(5), 451–457 |
| **Wang, 2004** | Wang J. (2004). A longitudinal population-based study of treated and untreated major depression. *Medical care*, *42*(6), 543–550 |
| **Wang, 2018** | Wang, P., Lv, Q., Mao, Y., Zhang, C., Bao, C., Sun, H., Chen, H., Yi, Z., Cai, W., & Fang, Y. (2018). HTR1A/1B DNA methylation may predict escitalopram treatment response in depressed Chinese Han patients. *Journal of affective disorders*, *228*, 222–228 |
| **Wang, 2018b** | Wang, P., Zhang, C., Lv, Q., Bao, C., Sun, H., Ma, G., Fang, Y., Yi, Z., & Cai, W. (2018). Association of DNA methylation in BDNF with escitalopram treatment response in depressed Chinese Han patients. *European journal of clinical pharmacology*, *74*(8), 1011–1020 |
| **Weinberger, 2015** | Weinberger, A. H., Platt, J., Jiang, B., & Goodwin, R. D. (2015). Cigarette Smoking and Risk of Alcohol Use Relapse Among Adults in Recovery from Alcohol Use Disorders. *Alcoholism, clinical and experimental research*, *39*(10), 1989–1996 |
| **Weinberger, 2016** | Weinberger, A. H., Platt, J., & Goodwin, R. D. (2016). Is cannabis use associated with an increased risk of onset and persistence of alcohol use disorders? A three-year prospective study among adults in the United States. *Drug and alcohol dependence*, *161*, 363–367 |
| **Wergeland, 2016** | Wergeland, G. J., Fjermestad, K. W., Marin, C. E., Bjelland, I., Haugland, B. S., Silverman, W. K., Öst, L. G., Bjaastad, J. F., Oeding, K., Havik, O. E., & Heiervang, E. R. (2016). Predictors of treatment outcome in an effectiveness trial of cognitive behavioral therapy for children with anxiety disorders. *Behaviour research and therapy*, *76*, 1–12 |
| **Wiersma, 1998** | Wiersma, D., Nienhuis, F. J., Slooff, C. J., & Giel, R. (1998). Natural course of schizophrenic disorders: a 15-year followup of a Dutch incidence cohort. *Schizophrenia bulletin*, *24*(1), 75–85 |
| **Wigman, 2014** | Wigman, J. T., van Os, J., Abidi, L., Huibers, M. J., Roelofs, J., Arntz, A., Kelleher, I., & Peeters, F. P. (2014). Subclinical psychotic experiences and bipolar spectrum features in depression: association with outcome of psychotherapy. *Psychological medicine*, *44*(2), 325–336 |
| **Wild, 2016** | Wild, B., Friederich, H. C., Zipfel, S., Resmark, G., Giel, K., Teufel, M., Schellberg, D., Löwe, B., de Zwaan, M., Zeeck, A., Herpertz, S., Burgmer, M., von Wietersheim, J., Tagay, S., Dinkel, A., & Herzog, W. (2016). Predictors of outcomes in outpatients with anorexia nervosa - Results from the ANTOP study. *Psychiatry research*, *244*, 45–50 |
| **Wilkinson, 2009** | Wilkinson, P., Dubicka, B., Kelvin, R., Roberts, C., & Goodyer, I. (2009). Treated depression in adolescents: predictors of outcome at 28 weeks. *The British journal of psychiatry : the journal of mental science*, *194*(4), 334–341 |
| **Williams, 2015** | Williams, L. M., Korgaonkar, M. S., Song, Y. C., Paton, R., Eagles, S., Goldstein-Piekarski, A., Grieve, S. M., Harris, A. W., Usherwood, T., & Etkin, A. (2015). Amygdala Reactivity to Emotional Faces in the Prediction of General and Medication-Specific Responses to Antidepressant Treatment in the Randomized iSPOT-D Trial. *Neuropsychopharmacology : official publication of the American College of Neuropsychopharmacology*, *40*(10), 2398–2408 |
| **Williams, 2014** | Williams, J. M., Crane, C., Barnhofer, T., Brennan, K., Duggan, D. S., Fennell, M. J., Hackmann, A., Krusche, A., Muse, K., Von Rohr, I. R., Shah, D., Crane, R. S., Eames, C., Jones, M., Radford, S., Silverton, S., Sun, Y., Weatherley-Jones, E., Whitaker, C. J., Russell, D., … Russell, I. T. (2014). Mindfulness-based cognitive therapy for preventing relapse in recurrent depression: a randomized dismantling trial. *Journal of consulting and clinical psychology*, *82*(2), 275–286 |
| **Wilson, 2003** | Wilson, K. C., Mottram, P. G., Ashworth, L., & Abou-Saleh, M. T. (2003). Older community residents with depression: long-term treatment with sertraline. Randomised, double-blind, placebo-controlled study. *The British journal of psychiatry : the journal of mental science*, *182*, 492–497 |
| **Wojnar, 2008** | Wojnar, M., Ilgen, M. A., Jakubczyk, A., Wnorowska, A., Klimkiewicz, A., & Brower, K. J. (2008). Impulsive suicide attempts predict post-treatment relapse in alcohol-dependent patients. *Drug and alcohol dependence*, *97*(3), 268–275 |
| **Wojnar, 2009** | Wojnar, M., Brower, K.J., Strobbe, S., Ilgen, M., Matsumoto, H., Nowosad, I., Sliwerska, E. and Burmeister, M. (2009), Association Between Val66Met Brain-Derived Neurotrophic Factor (BDNF) Gene Polymorphism and Post-Treatment Relapse in Alcohol Dependence. Alcoholism: Clinical and Experimental Research, 33: 693-702 |
| **Wolmer, 2008** | Wölwer, W., Brinkmeyer, J., Riesbeck, M., Freimüller, L., Klimke, A., Wagner, M., Möller, H. J., Klingberg, S., Gaebel, W., & German Study Group on First Episode Schizophrenia (2008). Neuropsychological impairments predict the clinical course in schizophrenia. *European archives of psychiatry and clinical neuroscience*, *258 Suppl 5*, 28–34 |
| **Wong, 2006** | Wong, J. O., Leung, S. P., Mak, T., Ng, R. M., Chan, K. T., Hon-Kee Cheung, H., Choi, W. K., Lai, J., & Wai-Kiu Tsang, A. (2006). Plasma clozapine levels and clinical response in treatment-refractory Chinese schizophrenic patients. *Progress in neuro-psychopharmacology & biological psychiatry*, *30*(2), 251–264 |
| **Woo, 2013** | Woo, Y.S., Bahk, W.-M., Jeong, J.-H., Lee, S.-H., Sung, H.-M., Pae, C.-U., Koo, B.-H. and Kim, W. (2013), Tianeptine for SSRI non-response. Psychiatry Clin Neurosci, 67: 219-227 |
| **Yackobovitch-Gavan, 2009** | Yackobovitch-Gavan, M., Golan, M., Valevski, A., Kreitler, S., Bachar, E., Lieblich, A., Mitrani, E., Weizman, A., & Stein, D. (2009). An integrative quantitative model of factors influencing the course of anorexia nervosa over time. *The International journal of eating disorders*, *42*(4), 306–317 |
| **Yan, 2004** | Yan, L. J., Hammen, C., Cohen, A. N., Daley, S. E., & Henry, R. M. (2004). Expressed emotion versus relationship quality variables in the prediction of recurrence in bipolar patients. *Journal of affective disorders*, *83*(2-3), 199–206 |
| **Yatham, 2009** | Yatham, L. N., Kauer-Sant'Anna, M., Bond, D. J., Lam, R. W., & Torres, I. (2009). Course and outcome after the first manic episode in patients with bipolar disorder: prospective 12-month data from the Systematic Treatment Optimization Program For Early Mania project. *Canadian journal of psychiatry. Revue canadienne de psychiatrie*, *54*(2), 105–112 |
| **Yazici, 1999** | Yazici, O., Kora, K., Uçok, A., Tunali, D., & Turan, N. (1999). Predictors of lithium prophylaxis in bipolar patients. *Journal of affective disorders*, *55*(2-3), 133–142 |
| **Yen 2016** | Yen, S, Stout, R, Hower, H, Killam, MA, Weinstock, LM, Topor, DR, Dickstein, DP, Hunt, JI, Gill, MK, Goldstein, TR, Goldstein, BI, Ryan, ND, Strober, M, Sala, R, Axelson, DA, Birmaher, B, Keller, MB. The influence of comorbid disorders on the episodicity of bipolar disorder in youth |
[truncated: 163,001 more chars]
